# Supplementary material for: AAV induces hepatic necroptosis and carcinoma in diabetic and obese mice dependent on Pebp1 pathway
Source: EMBO Mol Med. 2023 Jun 5;15(7):e17230. doi: 10.15252/emmm.202217230 (PMC10331584; doi:10.15252/emmm.202217230)
Supplement: Supplementary file 1 — Expanded View Figures PDF [file EMMM-15-e17230-s009.pdf]

Expanded View Figures

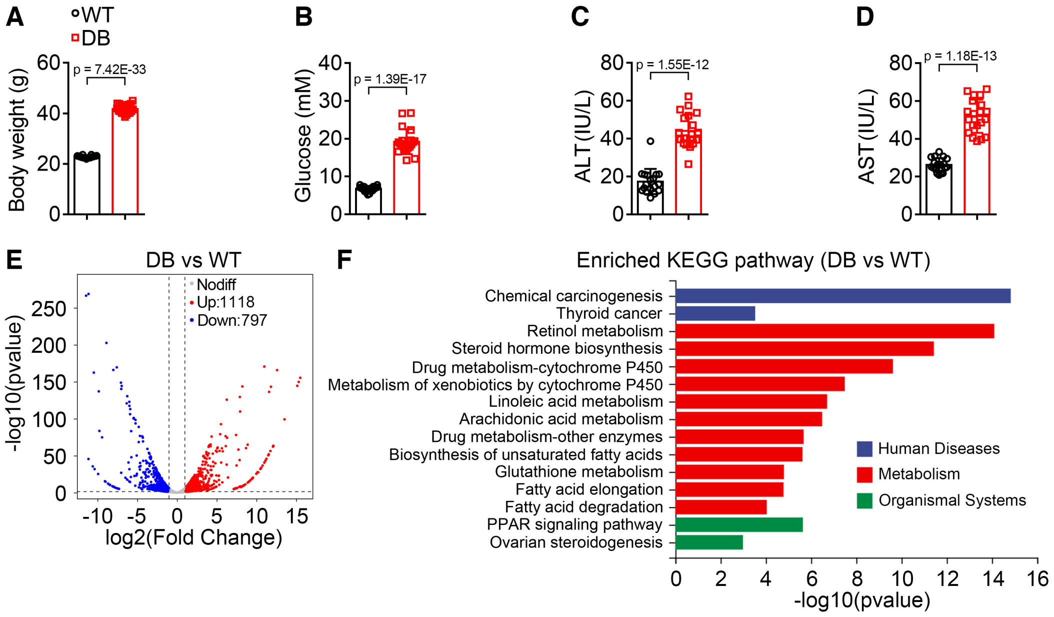

Figure EV1. Body weight, blood glucose levels, and serum ALT and AST activities, hepatic transcriptomic analysis of wild type and *db/db* mice.

A, B Body weight (A) and blood glucose levels (B) were significantly increased in *db/db* mice. *n* = 18–20 for each group.

C, D Serum alanine aminotransferase (ALT) (C) and aspartate aminotransferase (AST) (D) activities were significantly elevated in *db/db* mice. *n* = 18–20 for each group.

E Scatter plot comparing the differentially expressed genes detected by RNA-seq in liver of wild-type and *db/db* mice.

F The top 15 significantly enriched KEGG pathways of the differentially expressed genes in (E).

Data information: In (A–D), data are presented as mean ± SD. Student's *t*-test.

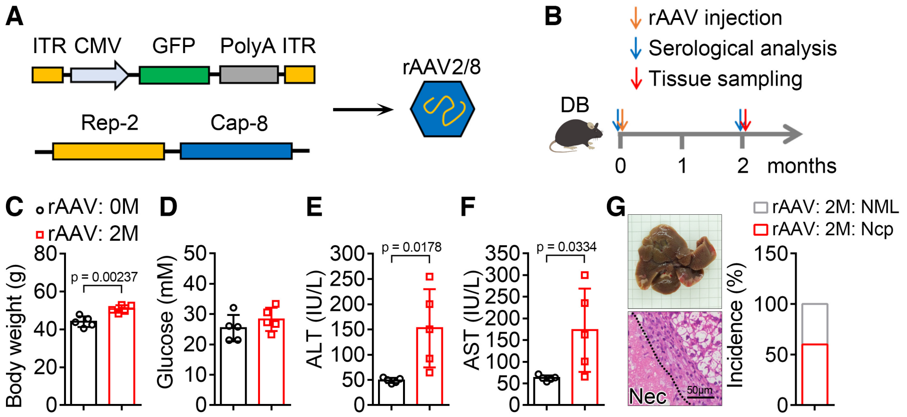

Figure EV2. Injection of rAAV serotype 2/8 can also lead to liver injury and necroptosis in *db/db* mice.

A, B Schematic of rAAV serotype 2/8 (rAAV2/8) package (A) and the experimental design for the effect of rAAV2/8 injection on liver function and hepatic necroptosis (B). ITR, AAV2 ITR.

C–F Two months after a single injection of rAAV2/8, *db/db* mice showed similar blood glucose levels (D), and increased body weight (C), serum ALT (E) and AST (F) activities compared with those before injection of rAAV2/8. *n* = 5.

G Liver images, H&E staining of liver sections and the incidence of hepatic necroptosis (Ncp) for mice in (C). NML, Normal.

Data information: In (C–F), data are presented as mean ± SD. Student's *t*-test.

© 2023 The Authors

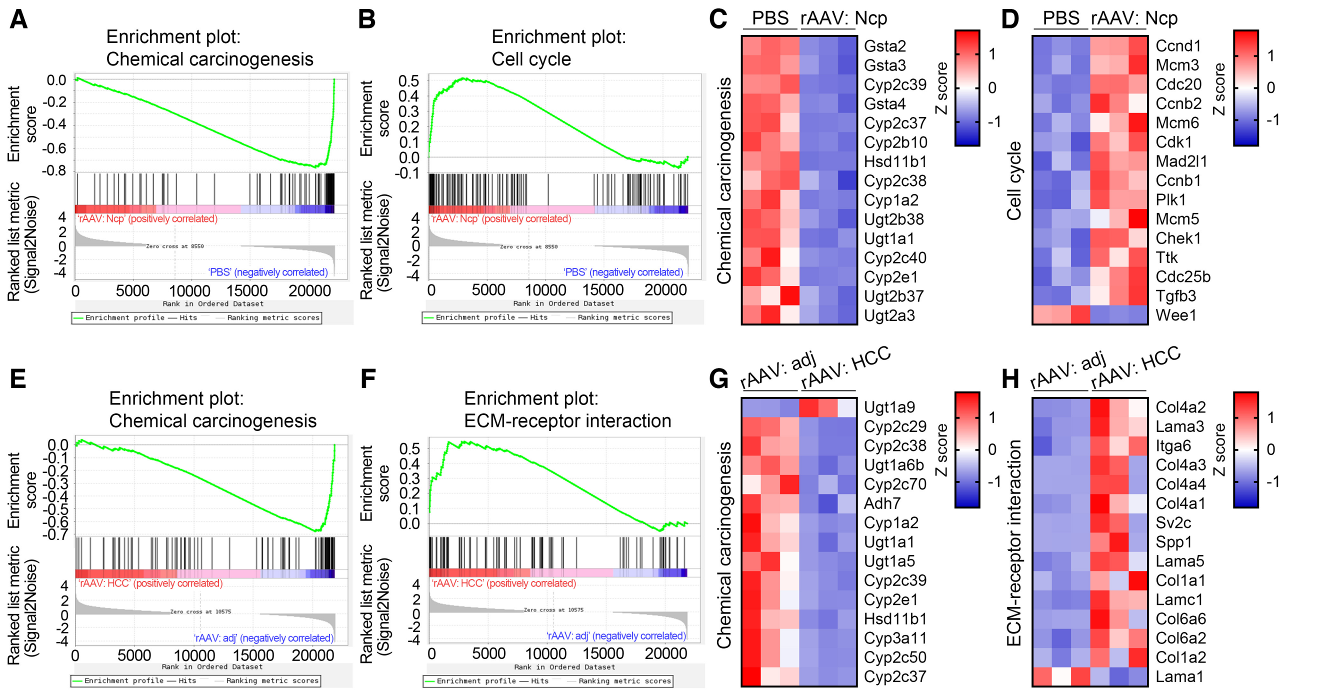

Figure EV3. GSEA analysis and heatmaps of the top 15 differentially expressed genes in the indicated enriched KEGG pathways.

A, B GSEA analysis of the indicated enriched KEGG pathways of the differentially expressed genes in normal or necroptotic area from the liver of mice after a single injection of PBS or rAAV for 2 months. Ncp, necroptosis.

C, D Heatmaps of the top 15 differentially expressed genes ranked by adjusted *P* value in the indicated KEGG pathways in (A) and (B).

E, F GSEA analysis of the indicated enriched KEGG pathways of the differentially expressed genes in adjacent or HCC area from the liver of mice after a single injection of rAAV for 6 months. adj, adjacent.

G, H Heatmaps of the top 15 differentially expressed genes ranked by adjusted *P* value in the indicated KEGG pathways in (E) and (F).

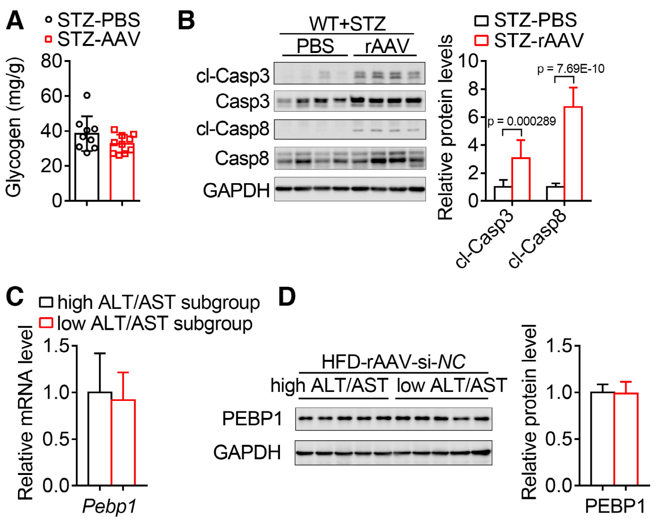

Figure EV4. Effect of rAAV injection on hepatic glycogen content and apoptosis in hyperglycemic mice and on endogenous Pebp1 level in liver of hyperglycemic and obese mice.

A Two months after a single injection of rAAV, hyperglycemic mice induced by streptozotocin showed similar glycogen content compared with those mice injected with PBS. n = 9–10.

B The hepatic protein levels of cleaved Caspase 3 (cl-Casp3) and cleaved Caspase 8 (cl-Casp8) in the livers of mice in (A) were measured to monitor apoptosis. n = 9–10.

C The hepatic mRNA level of *Pebp1* were similar in the high ALT & AST subgroup and low ALT & AST subgroup from HFD-induced hyperglycemic and obese mice injected with rAAV-si-*NC* as shown in Fig 6D and E. n = 5 biological replicates.

D The hepatic protein level of Pebp1 from mice in (C).

Data information: In (A–D), data are presented as mean ± SD. Student's t-test.

© 2023 The Authors

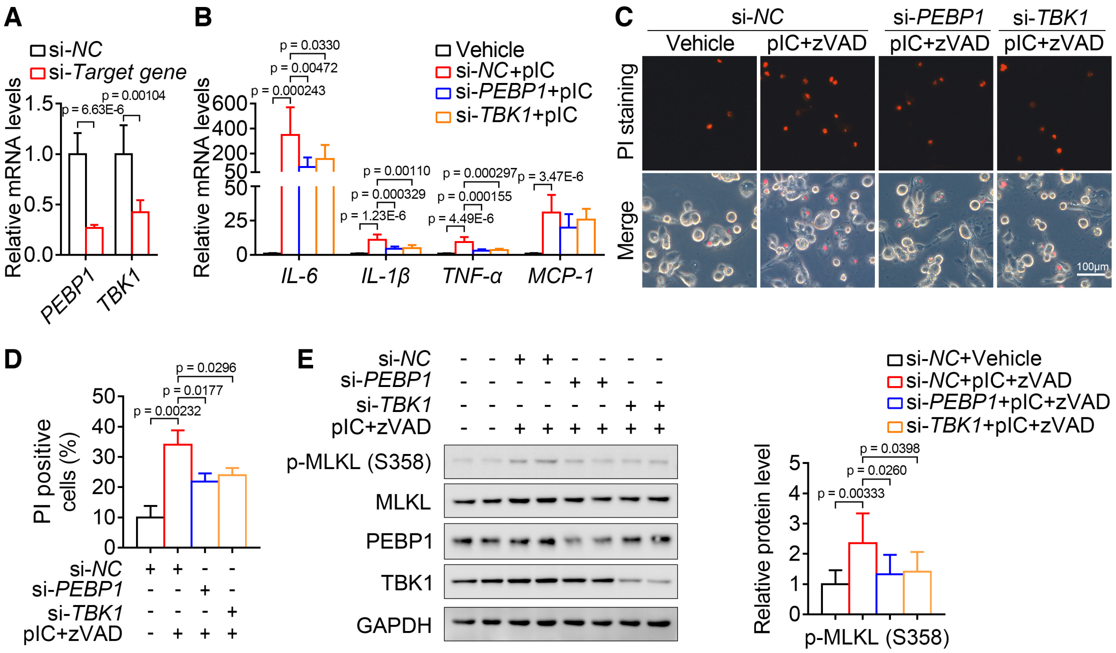

Figure EV5. Suppression of PEBP1 signaling alleviates inflammation and necroptosis in human macrophages.

A The effect of si-*PEBP1* and si-*TBK1* in THP-1 human macrophages. $n = 6$ biological replicates.

B Knockdown of PEBP1 and TBK1 markedly blocked the increase of some key inflammatory factors in THP-1 macrophages treated with poly(I:C). $n = 9$ biological replicates.

C–E Knockdown of PEBP1 and TBK1 markedly blocked the loss of cell membrane integrity (C-D, $n = 3$ in triplicate) and the increase of p-MLKL level (E, $n = 8$ biological replicates) of THP-1 macrophages induced by poly(I:C) and z-VAD-fmk.

Data information: In (A, B, D, E), data are presented as mean ± SD. Student's *t*-test.
