# Supplementary material for: AAV induces hepatic necroptosis and carcinoma in diabetic and obese mice dependent on Pebp1 pathway
Source: EMBO Mol Med. 2023 Jun 5;15(7):e17230. doi: 10.15252/emmm.202217230 (PMC10331584; doi:10.15252/emmm.202217230)
Supplement: Supplementary file 2 — PDF+ [file EMMM-15-e17230-s003.pdf]

Article

SOURCE DATA TRANSPARENT PROCESS OPEN ACCESS

EMBO Molecular Medicine

AAV induces hepatic necroptosis and carcinoma in diabetic and obese mice dependent on Pebp1 pathway

Yalan Cheng[1,†], Zhentong Zhang[1,†], Peidong Gao[1], Hejin Lai[1], Wuling Zhong[1], Ning Feng[1], Yale Yang[1], Huimin Yu[1], Yali Zhang[1], Yumo Han[2], Jieya Dong[1], Zhishui He[1], Rui Huang[1] & Qiwei Zhai[1,2,*]

Abstract

Obesity and diabetes are risk factors for hepatocellular carcinoma (HCC); however, the underlying mechanisms are yet to be elucidated. Adeno-associated virus (AAV) frequently infects humans and has been widely used in gene therapy, but the risk of AAV infection such as HCC should be further evaluated. Here, we show that recombinant AAV injection caused liver injury, hepatic necroptosis, and HCC in *db/db* or high-fat diet-induced hyperglycemic and obese mice, but not in mice with only hyperglycemia or obesity. Prednisone administration or knockdown of Pebp1, highly expressed in *db/db* mice, alleviated hepatic injury and necroptosis induced by recombinant AAV in mice with diabetes and obesity. Inhibition of Pebp1 pathway also attenuated inflammation and necroptosis *in vitro*. Our findings show that AAV infection is a critical risk factor for HCC in patients with diabetes and obesity, and AAV gene therapy for these patients should be carefully evaluated. Both prednisone treatment and targeting Pebp1 pathway are promising strategies to alleviate inflammation and necroptosis that occurred in AAV gene therapy or related diseases.

Keywords diabetes; HCC; necroptosis; obesity; rAAV
Subject Categories Cancer; Digestive System; Genetics, Gene Therapy & Genetic Disease
DOI 10.15252/emmm.202217230 | Received 30 November 2022 | Revised 8 May 2023 | Accepted 9 May 2023 | Published online 5 June 2023
EMBO Mol Med (2023) 15: e17230

Introduction

Both diabetes and obesity are considered as important risk factors for tumorigenesis, including hepatocellular carcinoma (HCC; Marengo *et al*, 2016; Mantovani & Targher, 2017; Lega & Lipscombe, 2020; Plaz Torres *et al*, 2022). A case–control study showed that a history of diabetes could explain around 8% of cases of liver cancer in the population (La Vecchia *et al*, 1997). Similarly, a history of diabetes at baseline is highly associated with HCC without hepatitis B or C virus infections (Koh *et al*, 2013). A study in 362,552 Swedish men showed that obese men have a significantly increased risk of all cancers (Samanic *et al*, 2006). Moreover, a European cohort study also provided evidence of an association between obesity, particularly abdominal obesity, and the risk of HCC (Schlesinger *et al*, 2013). A national population-based cohort study in Korean showed general obesity and central obesity are associated with an increased risk of HCC (Hwang *et al*, 2021). However, the underlying mechanisms for the high risk of HCC in patients with diabetes and obesity need further investigation.

It has been reported that the estimated total of infection-attributable cancer is 17.8% of the global cancer burden, and HBV and HCV account for 4.9% (Parkin, 2006). HBV and HCV are the leading risk factors for HCC globally (El-Serag, 2012; Teng *et al*, 2021). Around 54.4% of all liver cancer in the world is attributable to HBV (Parkin, 2006). A prospective study showed that persons with anti-HCV positivity and anti-HBV negativity have a 20-fold increased risk of developing HCC (Sun *et al*, 2003). Moreover, a follow-up study showed over 100-fold increased risk of HCC in HBV or HCV carriers with both obesity and diabetes (Chen *et al*, 2008), and overweight is associated with an increased risk of HCC occurrence in patients with HCV (N'Kontchou *et al*, 2006). However, whether other viral infections may also contribute to the risk of HCC in patients with diabetes and obesity is yet to be elucidated.

Adeno-associated virus (AAV) infection is around 30–80% in human populations (Erles *et al*, 1999; Halbert *et al*, 2006; Calcedo *et al*, 2009). However, no specific diseases have yet been associated with natural infection in most population studies, and AAV is usually regarded as nonpathogenic (Russell & Kay, 1999; Vasileva & Jessberger, 2005). Moreover, AAV vectors have broad tropism, low immunogenicity, rarely integrate into the host chromosome and can

1 CAS Key Laboratory of Nutrition, Metabolism and Food Safety, Shanghai Institute of Nutrition and Health, University of Chinese Academy of Sciences, Chinese Academy of Sciences, Shanghai, China
2 School of Life Science and Technology, ShanghaiTech University, Shanghai, China
*Corresponding author. Tel: +86-021-54920903; E-mail: qwzhai@sibs.ac.cn
[†]These authors contributed equally to this work

© 2023 The Authors. Published under the terms of the CC BY 4.0 license.

result in long-term expression of the transgene (Russell & Kay, 1999; Li & Samulski, 2020). Therefore, AAVs are considered as promising vectors with the excellent safety profile for gene therapy applications, and even approved for clinical applications (Nathwani *et al*, 2014; Keeler & Flotte, 2019; Li & Samulski, 2020). Meanwhile, it has been reported that gene therapy with recombinant AAV (rAAV) frequently leads to impaired serum alanine aminotransferase (ALT) and/or aspartate aminotransferase (AST) activities (Nathwani *et al*, 2014; George *et al*, 2017; Mendell *et al*, 2017; Ozelo *et al*, 2022), and high dose of AAV even led to liver dysfunction and death of four patients (Philippidis, 2020, 2021). Newly, a Phase Ib trial of AAV gene therapy was placed on hold after the death of a young male participant (Philippidis, 2022). However, the underlying mechanisms leading to liver dysfunction and death are still unclear. Recently, a participant in a Phase 3 trial of AAV gene therapy developed hepatocellular carcinoma, although whether AAV contributed to this cancer's development needs further investigation (Kaiser, 2020). The safety of AAV was further challenged by the findings of AAV vector integration sites in mouse HCC after AAV injection (Donsante *et al*, 2007). Furthermore, a long-term study of AAV gene therapy in dogs identified 1,741 unique AAV integration events in genomic DNA (Nguyen *et al*, 2021). It has also been reported that clonal integration of AAV in 11 of 193 HCCs, suggesting AAV is associated with oncogenic insertional mutagenesis in human HCC (Nault *et al*, 2015). Therefore, an association between AAV and HCC in the setting of therapeutic gene delivery has remained uncertain, and the mechanisms of AAV-correlated development of HCC are still largely unknown (Valdmanis *et al*, 2012; Chandler *et al*, 2015). Moreover, whether rAAV injection in diabetic and obese models will lead to the development of HCC is still unclear.

In this study, we investigated the effect of rAAV injection in hyperglycemic and/or obese mice, and the potential protective role of prednisone. Liver injury, hepatic necroptosis, and HCC were monitored in those mice. Moreover, Pebp1 was screened, and we investigated its role in inflammation and necroptosis *in vivo* and *in vitro*. Our data demonstrate that rAAV induces prednisone-protectable liver injury, hepatic necroptosis, and HCC in diabetic and obese mice via Pebp1 pathway.

Results

Injection of rAAV induces liver injury, hepatic necroptosis, and carcinoma in *db/db* mice

To investigate whether rAAV injection will affect liver function and lead to the development of HCC in mice with diabetes and obesity, rAAV serotype 2/9 was constructed and injected into wild-type and *db/db* male mice at the age of 9 weeks through tail vein (Fig 1A and B). The increased body weight, blood glucose levels, and serum ALT and AST activities in the diabetic and obese *db/db* mice were confirmed (Fig EV1A–D). Two months after a single injection of rAAV, wild-type mice showed similar body weight, blood glucose levels, serum ALT and AST activities with the mice injected with PBS (Fig 1C–F). Injection of rAAV also had no noticeable effect on the hepatic morphology and histomorphology analyzed by H&E staining in wild-type mice (Fig 1G). Meanwhile, *db/db* mice injected with rAAV showed similar body weight and blood glucose levels, but markedly increased serum ALT and AST activities compared with those injected with PBS (Fig 1H–K). Moreover, rAAV injection induced hepatic necrosis and death in *db/db* mice, and the incidence of necrosis and death was around 58 and 17%, respectively (Fig 1L).

To investigate whether the necrosis is necroptosis, phosphorylation of MLKL as a specific marker of necroptosis was detected (Pasparakis & Vandenabeele, 2015). The p-MLKL level in necrotic area was dramatically increased compared with that in normal area (Fig 1M), which indicated that necroptosis was induced in livers of

Figure 1. Tail vein injection of rAAV induces liver injury, hepatic necroptosis, and carcinoma in *db/db* but not wild-type mice.

- A, B Schematic of rAAV serotype 2/9 (rAAV2/9) package for expressing si-*NC* and ZsGreen (A) and the experimental design for the effect of rAAV injection on liver function and the potential development of liver cancer (B). ITR, AAV2 ITR. Except indicated, rAAV2/9 expressing si-*NC* and ZsGreen were used.
- C–F Two months after a single tail vein injection of rAAV, wild-type mice showed similar body weight (C), blood glucose levels (D), serum alanine aminotransferase (ALT) (E) and aspartate aminotransferase (AST) activities (F) with the mice injected with PBS. $n = 8–10$ for each group.
- G Liver images, H&E staining of liver sections and the incidence of hepatic necrosis (Nec) for mice in (C). NML, Normal.
- H, I Two months after a single injection of rAAV, *db/db* mice showed similar body weight (H) and blood glucose levels (I) with the mice injected with PBS. $n = 10$ for each group.
- J, K Serum ALT (J) and AST (K) of mice in (H).
- L Liver images, H&E staining of liver sections for mice in (H) and the incidence of death and hepatic necrosis of *db/db* mice after a single injection of PBS or rAAV for 2 months. $n = 10–12$ for each group.
- M The hepatic p-MLKL protein level in normal and necrotic area from mice injected with PBS or rAAV in (H). Ncp, Necroptosis. $n = 7–8$ biological replicates.
- N The hepatic mRNA levels of *Il-6*, *Il-1β*, *Tnf-α* and *Mcp-1* involved in necroptosis at normal or necroptotic area from mice injected with PBS or rAAV in (H). $n = 7–8$ biological replicates.
- O Scatter plots comparing the differentially expressed hepatic genes of mice in (H).
- P The top 15 significantly enriched KEGG pathways of the differentially expressed genes in (O).
- Q, R Six months after a single injection of rAAV, *db/db* mice showed decreased body weight (Q) and similar blood glucose levels (R) compared to the mice injected with PBS. $n = 8–12$.
- S, T Serum ALT (S) and AST (T) activities of mice in (Q). $n = 8–10$.
- U Liver images, H&E staining, and ki67 immunohistochemistry of liver sections for mice in (Q) and the incidence of death and HCC of *db/db* mice after a single injection of PBS or rAAV for 6 months. $n = 8–13$.
- V Scatter plots comparing the differentially expressed hepatic genes of mice in (Q). adj, adjacent.
- W The top 15 significantly enriched KEGG pathways of the differentially expressed genes in (V).

Data information: In (C–F, H–K, M, N, Q–T), data are presented as mean ± SD. Student's *t*-test.
Source data are available online for this figure.

© 2023 The Authors

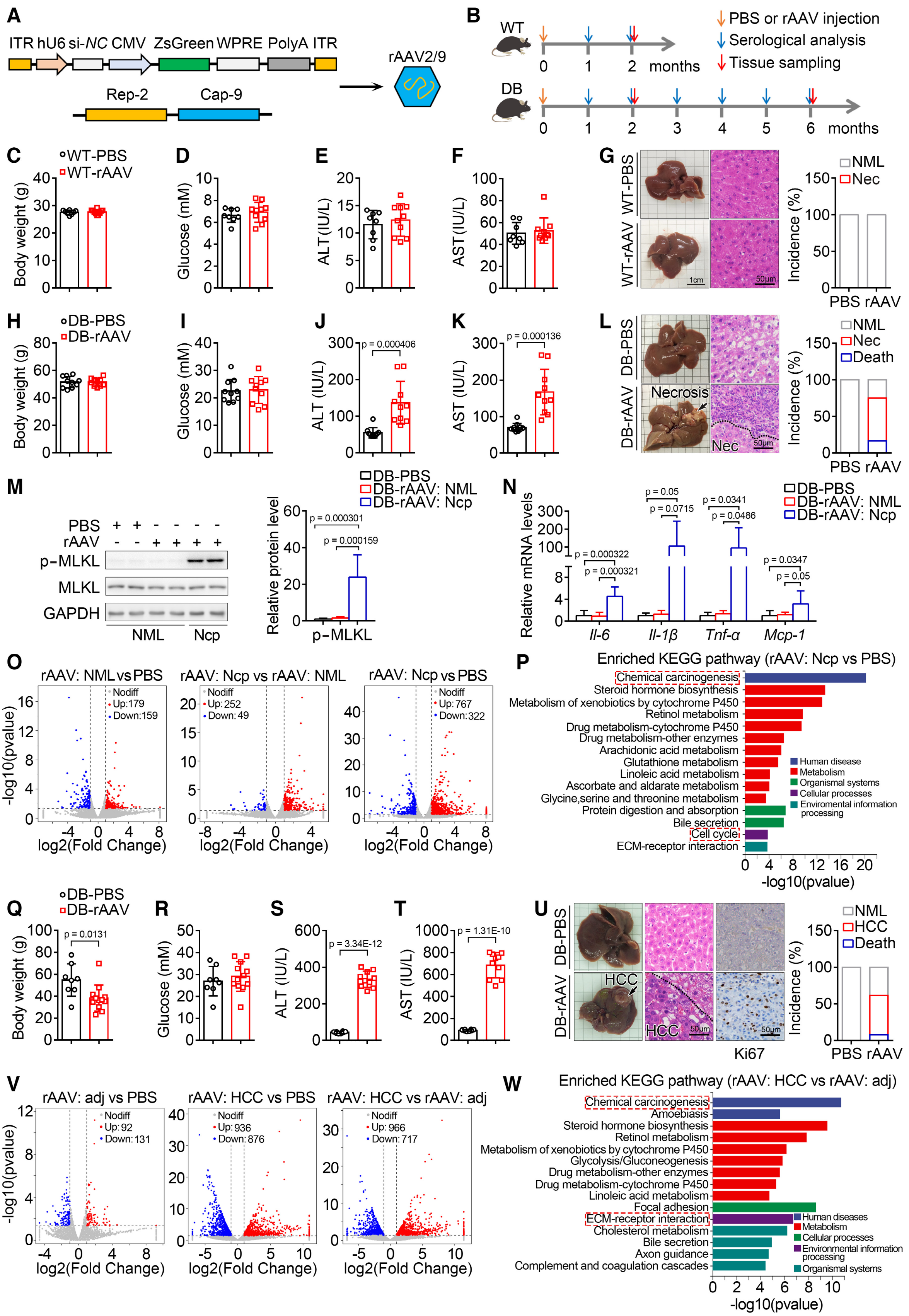
A
ITR hU6 si-NC CMV ZsGreen WPRE PolyA ITR
Rep-2 Cap-9
rAAV2/9
B
PBS or rAAV injection
Serological analysis
Tissue sampling
WT
DB
months
Necrosis
Nec
HCC
Ki67
p-MLKL
MLKL
GAPDH
Enriched KEGG pathway (rAAV: Ncp vs PBS)
Enriched KEGG pathway (rAAV: HCC vs rAAV: adj)
log2(Fold Change)
-log10(pvalue)

Figure 1.

db/db mice after a single injection of rAAV for 2 months. Necroptosis is usually accompanied with inflammation (Newton & Manning, 2016); therefore, the hepatic mRNA levels of some key inflammatory factors were monitored. As expected, the mRNA levels of *Il-6*, *Il-1β*, *Tnf-α*, and *Mcp-1* were significantly increased in necroptotic area compared with those in normal area (Fig 1N). To further investigate the effects of other serotypes of rAAV, rAAV serotype 2/8 was constructed and injected into *db/db* mice in the same manner. Two months after a single injection of rAAV serotype 2/8, liver injury and hepatic necroptosis were also observed in *db/db* mice (Fig EV2A–G). These data demonstrate that rAAV injection can lead to liver injury and necroptosis in *db/db* mice but not in wild-type mice.

We next analyzed gene expression profiles of normal and necroptotic areas from livers of *db/db* mice after a single injection of PBS or rAAV for 2 months. In this and the following experiments, rAAV serotype 2/9 were used. The scatter plots of differentially expressed genes were shown in Fig 1O. The top 15 significantly enriched KEGG pathways of the differentially expressed genes between normal and necroptotic areas from mice injected with PBS and rAAV, respectively, were shown in Fig 1P. GSEA analysis of the enriched chemical carcinogenesis and cell cycle pathways related to carcinogenesis were shown in Fig EV3A and B, and the top 15 differentially expressed genes in these two enriched KEGG pathways were shown in Fig EV3C and D. To further investigate whether rAAV injection will lead to the development of HCC in *db/db* mice, the mice were analyzed after a single injection of rAAV or PBS for 6 months. The *db/db* mice injected with rAAV showed decreased body weight, similar blood glucose levels, and markedly increased serum ALT and AST activities compared with those injected with PBS (Fig 1Q–T). Furthermore, as expected, obvious liver tumors could be observed in around 54% of *db/db* mice after a single injection of rAAV for 6 months and around 8% of these mice died (Fig 1U). The hepatic carcinoma was confirmed by H&E staining and ki67 immunohistochemistry of liver sections (Fig 1U). Moreover, we analyzed gene expression profiles of HCC and adjacent areas from livers of *db/db* mice after a single injection of rAAV for 6 months. The scatter plots of differentially expressed genes were shown in Fig 1V. The top 15 significantly enriched KEGG pathways of the differentially expressed genes between HCC and adjacent areas were shown in Fig 1W. GSEA analysis of the enriched chemical carcinogenesis and ECM-receptor interaction pathways related to carcinogenesis were shown in Fig EV3E and F, and the top 15 differentially expressed genes in these two enriched KEGG pathways were shown in Fig EV3G and H.

Taken together, these data demonstrate that injection of rAAV induces liver injury, necroptosis, and development of HCC in *db/db* mice but not in wild-type mice.

Injection of rAAV induces liver injury, but not hepatic necroptosis, in mice with only hyperglycemia or obesity

To investigate whether rAAV injection induces liver injury, hepatic necroptosis, and carcinoma in mice with only hyperglycemia, wild-type mice were consecutively injected with 40 mg/kg/day streptozotocin intraperitoneally for 5 days to induce hyperglycemia. The schematic of the experimental design was shown in Fig 2A. Injection with streptozotocin led to a decrease in body weight and an obvious increase in blood glucose levels (Fig 2B and C). After a single injection of rAAV for 2 months, hyperglycemic mice induced by streptozotocin showed similar body weight, blood glucose levels, and hepatic glycogen content, but increased liver weight, serum ALT and AST activities, and apoptotic liver cells compared with those injected with PBS (Figs 2D–H, and EV4A and B). Hepatic morphological and histomorphological analysis showed that rAAV injection did not cause necroptosis in livers of streptozotocin-induced hyperglycemic mice (Fig 2I). Moreover, there was no significant change of p-MLKL level in livers of streptozotocin-induced hyperglycemic mice after rAAV injection (Fig 2J).

To investigate whether rAAV injection induces liver injury, hepatic necroptosis and carcinoma in mice with obesity and euglycemia, resistance to HFD-induced hyperglycemia (RHH) mice were fed with high-fat diet (HFD) to induce obesity. The schematic of the experimental design was shown in Fig 2K. Resistance to HFD-induced hyperglycemia mice fed with HFD for 12 weeks showed significantly increased body weight and fat mass but normal blood glucose levels (Fig 2L–N). Two months after a single injection of rAAV, obese but euglycemic RHH mice showed similar body weight, liver weight and blood glucose levels, and significantly increased serum ALT and AST activities compared with those injected with PBS (Fig 2O–S). Hepatic morphological and histomorphological analysis showed that rAAV injection did not cause necroptosis in livers of the obese but euglycemic mice (Fig 2T). Moreover, there was no significant change of p-MLKL level in livers of the obese but euglycemic mice after rAAV injection (Fig 2U).

Taken together, neither hyperglycemia nor obesity alone is sufficient for rAAV-induced hepatic necroptosis in mice.

Injection of rAAV induces liver injury, hepatic necroptosis, and carcinoma in HFD-induced hyperglycemic and obese mice

To further investigate the contribution of hyperglycemia and obesity to the liver injury, hepatic necroptosis, and carcinoma induced by rAAV injection, wild-type mice were fed with HFD to induce hyperglycemia and obesity. The schematic of the experimental design was shown in Fig 3A. Body weight, fat mass, and blood glucose levels were significantly increased in mice fed with HFD for 12 weeks (Fig 3B–D). Two months after a single injection of rAAV, hyperglycemic and obese mice induced by HFD showed similar body weight, liver weight, and blood glucose levels, but significantly increased serum ALT and AST activities compared with those injected with PBS (Fig 3E–I). Hepatic morphological and histomorphological analysis showed that rAAV injection led to hepatic necroptosis in hyperglycemic and obese mice induced by HFD (Fig 3J). Moreover, the protein level of p-MLKL and the mRNA levels of some key inflammatory factors in necroptotic area were dramatically increased compared with those in normal area (Fig 3K and L).

To further investigate whether rAAV injection will lead to the development of HCC in hyperglycemic and obese mice induced by HFD, the mice were analyzed after a single injection of rAAV for 6 months. The mice injected with rAAV showed similar body weight and blood glucose levels, decreased liver weight, and increased serum ALT and AST activities compared with those injected with PBS (Fig 3M–Q). Furthermore, as expected, obvious liver tumors could be observed in around 38% of the hyperglycemic

 © 2023 The Authors

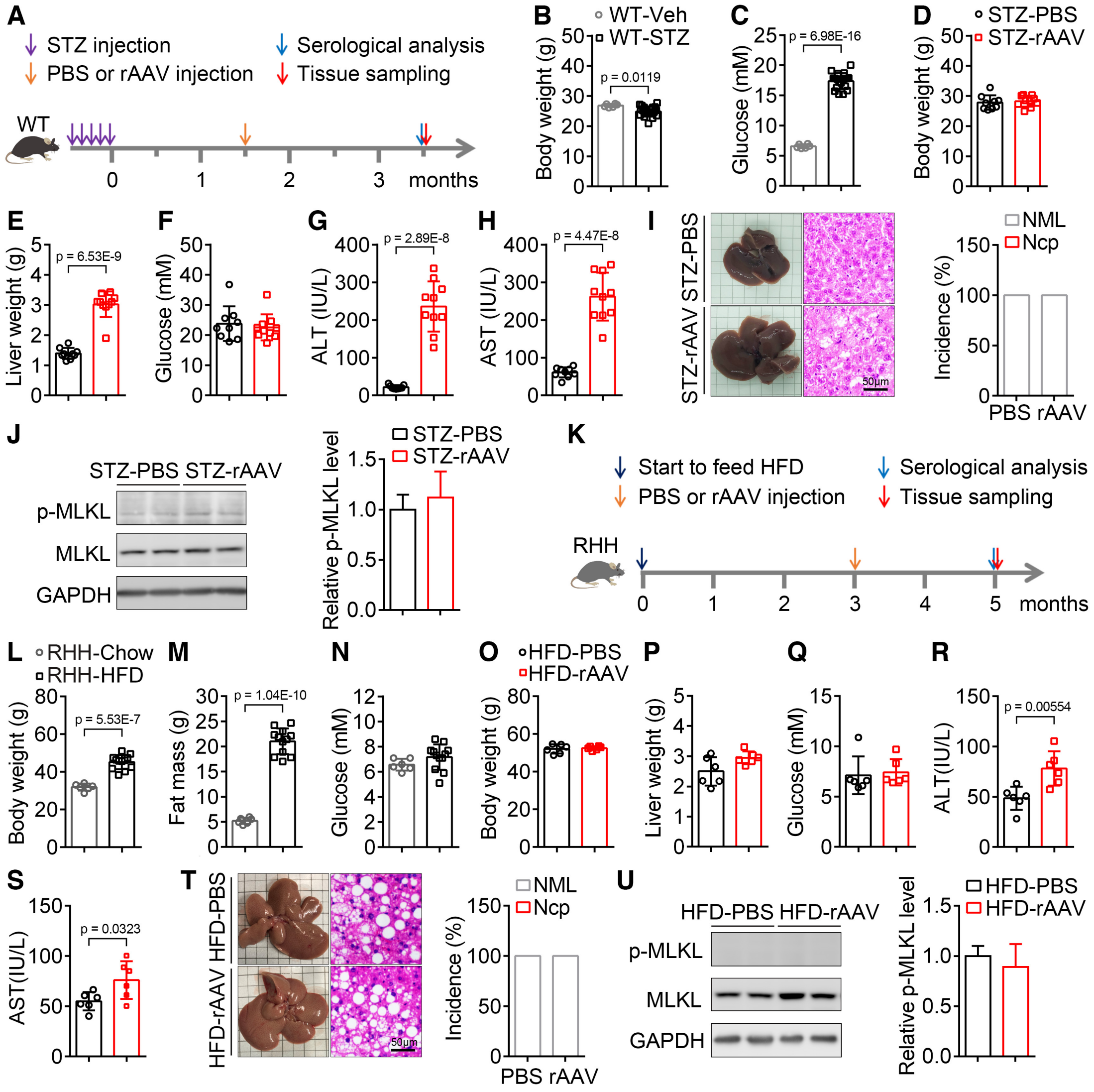

Figure 2. Tail vein injection of rAAV induces liver injury, but not hepatic necroptosis, in mice with only hyperglycemia or obesity.

A Schematic of the hyperglycemic mouse model construction by streptozotocin (STZ) and the experimental design for the contribution of hyperglycemia to the liver injury and hepatic necroptosis induced by rAAV injection.

B, C Body weight (B) and blood glucose levels (C) were measured 6 weeks later, after injection once a day for 5 consecutive days with vehicle (Veh) or streptozotocin (STZ). n = 6–19 for each group.

D–F Two months after a single injection of rAAV, hyperglycemic mice induced by streptozotocin showed similar body weight (D), blood glucose levels (F) and increased liver weight (E) compared with those mice injected with PBS. n = 9–10.

G, H Serum ALT (G) and AST (H) activities of mice in (D).

I Liver images, H&E staining of liver sections and the incidence of hepatic necroptosis for mice in (D).

J The hepatic p-MLKL level of mice in (D).

K Schematic of the obese mouse model construction induced by high-fat diet (HFD) and the experimental design for the contribution of obesity to the liver injury and hepatic necroptosis induced by rAAV injection.

L–N Body weight (L), fat mass (M) and blood glucose levels (N) of RHH (Resistance to HFD-induced Hyperglycemia) mice fed with HFD for 15 weeks. n = 6–12.

O–Q Body weight (O), liver weight (P) and blood glucose levels (Q) of obese and euglycemic mice in (L) after a single injection of PBS or rAAV for 2 months. n = 6.

R, S Serum ALT (R) and AST (S) activities of mice in (O).

T Liver images, H&E staining of liver sections and incidence of hepatic necroptosis for mice in (O).

U The hepatic p-MLKL level of mice in (O).

Data information: In (B–H, J, L–S, U), data are presented as mean ± SD. Student's t-test.
Source data are available online for this figure.

and obese mice after a single injection of rAAV for 6 months (Fig 3R). The hepatic carcinoma was further confirmed by H&E staining and ki67 immunohistochemistry of liver sections (Fig 3R).

Taken together, rAAV injection induces liver injury, hepatic necroptosis, and carcinoma in mice with both hyperglycemia and obesity.

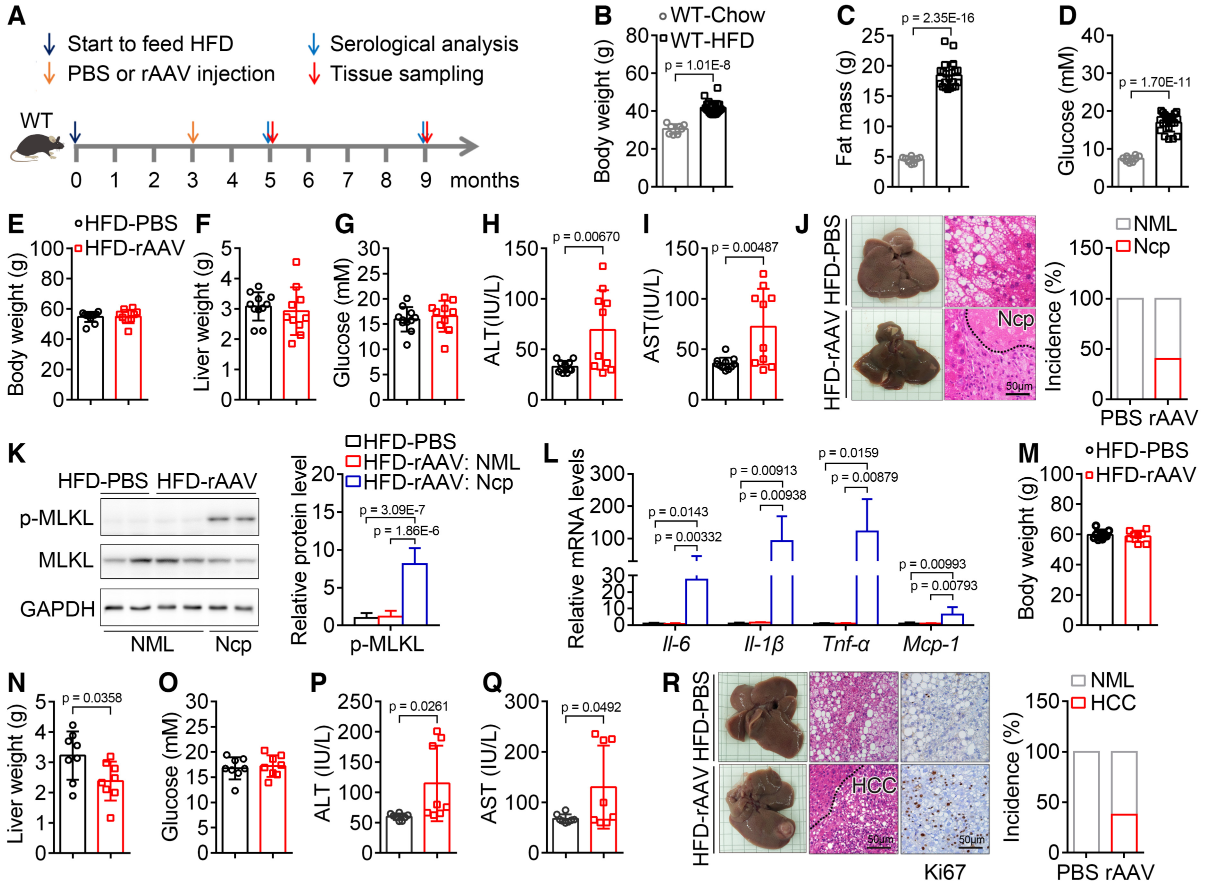

Figure 3. Tail vein injection of rAAV induces liver injury, hepatic necroptosis, and carcinoma in mice with both hyperglycemia and obesity.

A Schematic of the hyperglycemic and obese mouse model construction and the experimental design for the contribution of hyperglycemia and obesity to the liver injury, hepatic necroptosis and carcinoma induced by rAAV.

B–D Body weight (B), fat mass (C), and blood glucose levels (D) of mice fed with chow or high-fat diet (HFD) for 15 weeks. n = 8–21.

E–G Two months after a single injection of PBS or rAAV, body weight (E), liver weight (F), and blood glucose levels (G) of hyperglycemic and obese mice in (B) were not significantly changed. n = 10–11 for each group.

H, I Serum ALT (H) and AST (I) activities of mice in (E).

J Liver images, H&E staining of liver sections and the incidence of hepatic necroptosis (Ncp) for mice in (E). NML, Normal.

K The hepatic p-MLKL protein level in normal or necrotic area from mice injected with PBS or rAAV in (E).

L The hepatic mRNA levels of *Il-6*, *Il-1β*, *Tnf-α* and *Mcp-1* involved in necroptosis at normal or necroptotic area from mice in (E).

M–O Body weight (M), liver weight (N) and blood glucose levels (O) of hyperglycemic and obese mice after a single injection of PBS or rAAV for 6 months. n = 8.

P, Q Serum ALT (P) and AST (Q) activities of mice in (M).

R Liver images, H&E staining and ki67 immunostaining of liver sections and HCC incidence of mice in (M).

Data information: In (B–I, K–Q), data are presented as mean ± SD. Student's t-test.
Source data are available online for this figure.

Oral administration of prednisone significantly alleviates rAAV-induced liver injury and hepatic necroptosis in *db/db* mice

It has been reported that asymptomatic increase in serum AST and/or ALT activities induced by gene therapy with AAV vector can be resolved with prednisone treatment (George *et al*, 2017; Mendell *et al*, 2017). To investigate whether prednisone can also attenuate liver injury, hepatic necroptosis, and carcinoma induced by rAAV injection in mice with hyperglycemia and obesity, we first investigated whether prednisone can alleviate inflammation and necroptosis *in vitro*. It has been reported that Poly(I:C) has been used to mimic virus infection in cell model to induce inflammation (Liu *et al*, 2019). As shown in Fig 4A, some inflammatory factors were dramatically induced by poly(I:C) in primary macrophages, which was markedly alleviated by prednisone. Then, we directly analyzed the effect of prednisone on necroptosis in a cell model induced by poly(I:C) and z-VAD-fmk (Pearson *et al*, 2017). Necroptosis induced by poly(I:C) and z-VAD-fmk in primary macrophages monitored by propidium iodide staining and p-MLKL level was also significantly inhibited by prednisone (Fig 4B and C). Furthermore, we fed *db/db* mice with chow diet containing prednisone for 2 months after a single injection of rAAV (Fig 4D). As shown in Fig 4E–I, prednisone treatment had no significant effect on body weight, liver weight, and blood glucose levels, but markedly decreased serum ALT and

 © 2023 The Authors

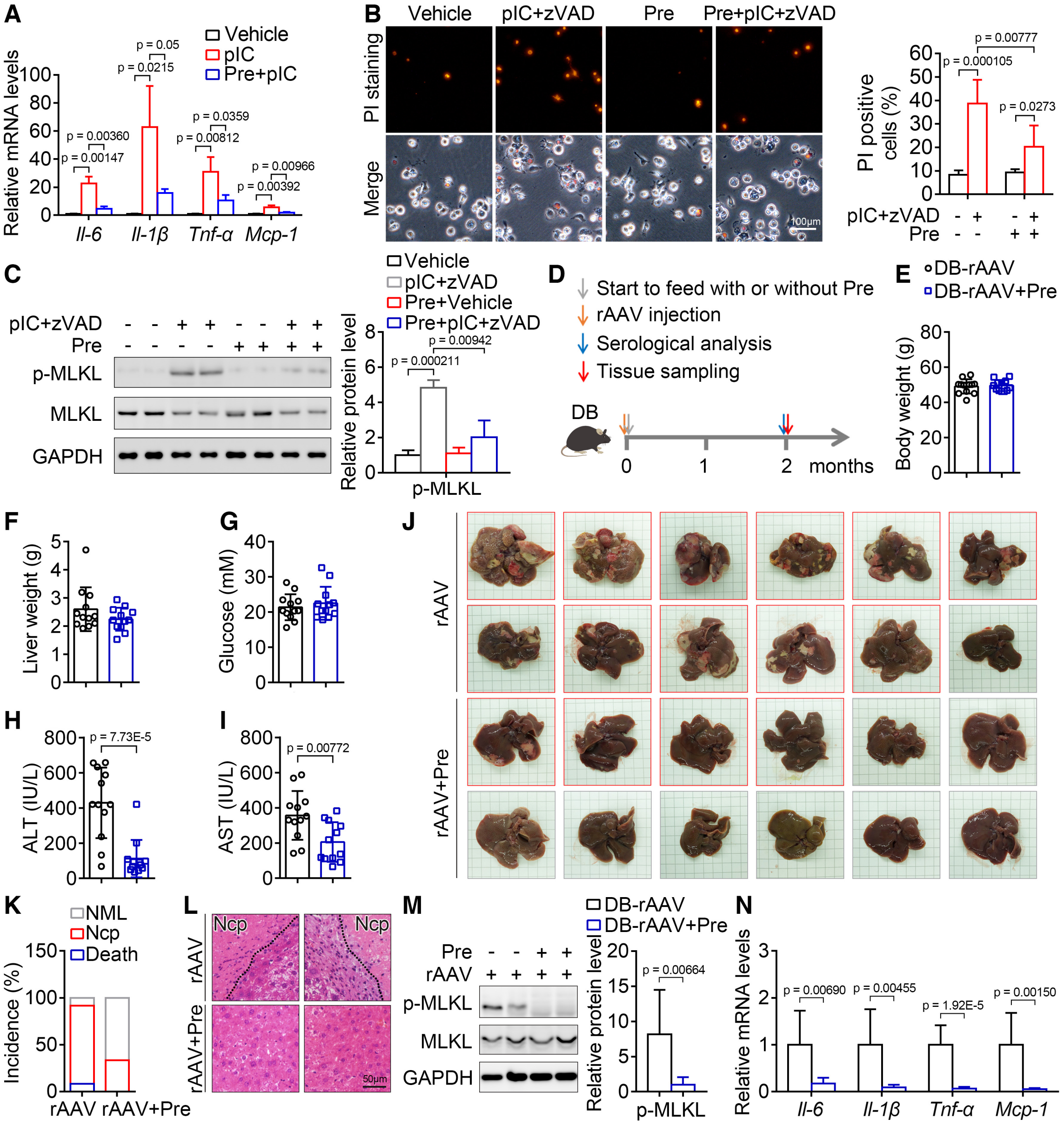

Figure 4. Prednisone alleviates inflammation and necroptosis *in vitro* and attenuates liver injury and hepatic necroptosis in *db/db* mice injected with rAAV.

A Prednisone treatment markedly blocked the increase of some key inflammatory factors in mouse macrophages treated with poly(I:C) (pIC). n = 3 in duplicate.

B, C Prednisone treatment markedly blocked the loss of cell membrane integrity monitored by PI staining (B, n = 5–6 in duplicate or triplicate.) and the increase in p-MLKL level (C, n = 3 in duplicate.) of macrophages induced by poly(I:C) and z-VAD-fmk (zVAD). Scar bar, 100 μm.

D Schematic of the experimental design for the effect of prednisone on liver injury and hepatic neccroptosis induced by rAAV.

E–G Two months after a single injection of rAAV, the *db/db* mice treated with or without prednisone showed similar body weight (E), liver weight (F) and blood glucose levels (G). n = 12.

H, I Serum ALT (H) and AST (I) activities of mice in (E).

J Representative liver images for mice in (E).

K The incidence of death and hepatic necroptosis (Ncp) for mice in (E). NML, Normal.

L Representative H&E staining of liver sections for mice in (E).

M The hepatic p-MLKL level of mice in (E).

N The indicated hepatic mRNA levels of mice in (E).

Data information: In (A–C, E–I, M, N), Data are presented as mean ± SD. Student's t-test.
Source data are available online for this figure.

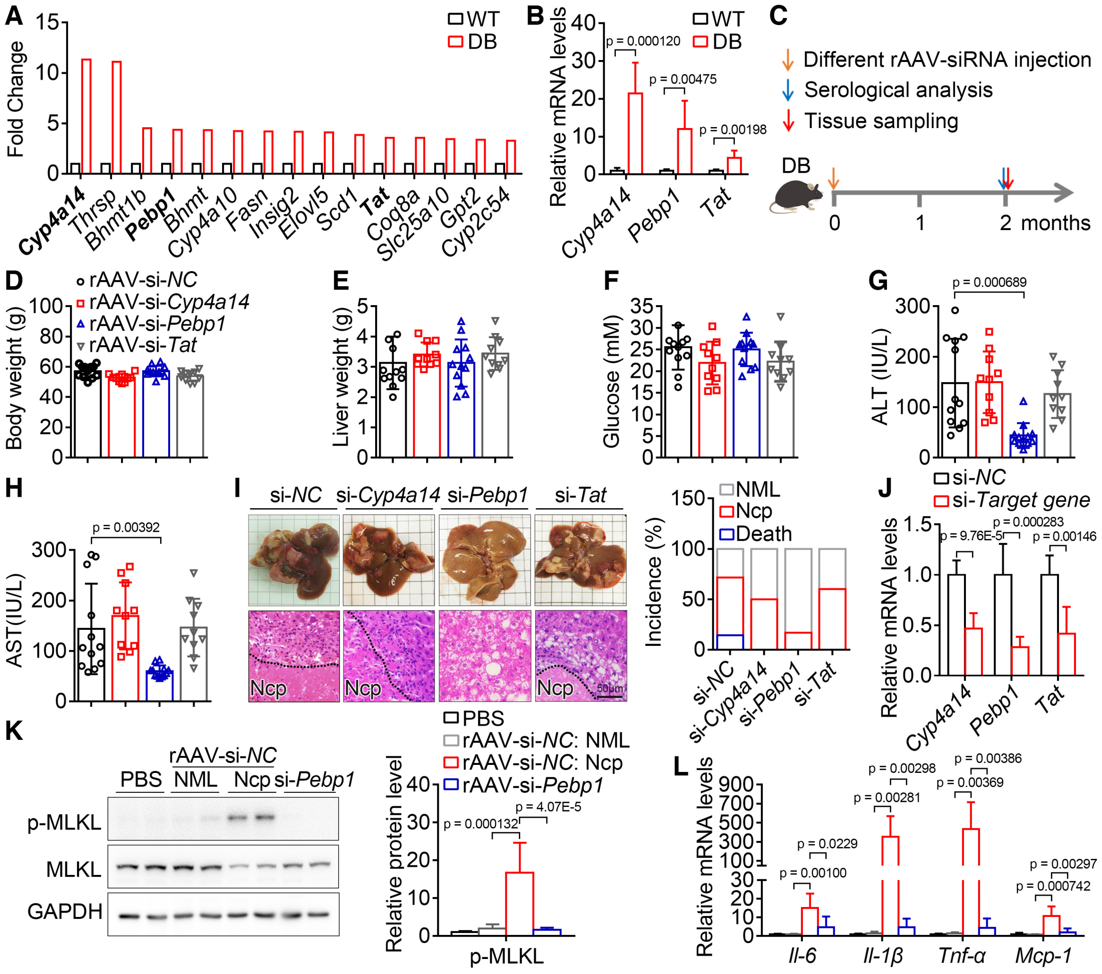

Figure 5. Downregulation of Pebp1 prevents rAAV-induced liver injury and hepatic necroptosis in *db/db* mice.

- A The top 15 hepatic genes upregulated in 9-week-old *db/db* mice compared with wild-type mice. $n = 1$, liver samples from three mice were mixed together in equal amounts for RNA extraction.
- B Relative mRNA levels of *Cyp4a14*, *Pebp1* and *Tat* in livers of wild type and *db/db* mice. $n = 6$ for each group.
- C Schematic of the experimental design to study the role of candidate genes in liver injury and hepatic necroptosis induced by rAAV.
- D–F Body weight (D), liver weight (E), and blood glucose levels (F) of *db/db* mice after a single injection of rAAV-si-*NC/Cyp4a14/Pebp1/Tat* for 2 months. $n = 10–12$. rAAV-si-*NC* was packaged as indicated in Fig 1A and mentioned as rAAV in Figs 1–4.
- G, H Serum ALT (G) and AST (H) activities of mice in (D).
- I Liver images, H&E staining of liver sections and the incidence of death and hepatic necroptosis (Ncp) of mice in (D). $n = 10–14$, NML, Normal.
- J The indicated hepatic mRNA levels of mice in (D).
- K The hepatic p-MLKL level of the indicated mice in (D).
- L The indicated hepatic mRNA levels of mice in (K).

Data information: In (B, D–H, J–L), Data are presented as mean ± SD. Student's *t*-test.
Source data are available online for this figure.

AST activities in *db/db* mice after a single injection of rAAV for 2 months. Moreover, prednisone administration dramatically inhibited hepatic necroptosis induced by rAAV injection when analyzed by morphological analysis, and the incidence of hepatic necroptosis was decreased from 83 to 33%; meanwhile, the death ratio was significantly decreased (Fig 4J and K). The effect of prednisone on hepatic necroptosis was further confirmed by H&E staining and immunoblot analysis of p-MLKL level (Fig 4L and M). In addition, some inflammatory factors increased in necroptotic area were dramatically decreased by prednisone treatment (Fig 4N).

These data demonstrate that prednisone treatment protects *db/db* mice injected with rAAV from liver injury and hepatic necroptosis, and prednisone is likely to alleviate liver injury through blocking hepatic necroptosis.

Pebp1 mediates rAAV-induced liver injury and hepatic necroptosis in *db/db* mice

To further investigate why hyperglycemic and obese mice are sensitive to rAAV injection, the gene expression profile of liver samples

 © 2023 The Authors

from wild-type and *db/db* mice was analyzed. The scatter plot of differentially expressed genes was shown in Fig EV1E. The top 15 significantly enriched KEGG pathways of the differentially expressed genes were shown in Fig EV1F, and the top 15 genes upregulated in *db/db* mice were shown in Fig 5A. Among the top 15 genes upregulated in *db/db* mice, Cyp4a14 plays an important role in the development and progression of NAFLD (Zhang *et al*, 2017), Pebp1 is an inflammatory and immune system modulator (Lai *et al*, 2017; Gabriela-Freitas *et al*, 2019), and Tat plays an important suppressive role in the development and progression of HCC (Fu *et al*, 2010). However, the effects of these three genes involved in different signaling pathways on diabetes or necroptosis are still largely unknown. The increase in *Cyp4a14*, *Pebp1*, and *Tat* were validated by qPCR and selected for further investigation (Fig 5B). The rAAV expressing siRNAs targeting these genes were injected via tail vein into *db/db* mice, and the schematic of the experimental design was shown in Fig 5C. Two months after a single injection of rAAV expressing the siRNAs, each group of *db/db* mice showed similar body weight, liver weight, and blood glucose levels (Fig 5D–F). Serum ALT and AST activities were significantly decreased in *db/db* mice injected with rAAV-si-*Pebp1* compared with those injected with rAAV-si-*NC*, rAAV-si-*Cyp4a14*, or rAAV-si-*Tat* (Fig 5G and H). Similarly, hepatic morphological and histomorphological analysis showed that downregulation of Pebp1 markedly alleviated rAAV-induced hepatic necroptosis from around 57% to around 17% in *db/db* mice, and the death ratio was significantly decreased (Fig 5I). The effect of these siRNAs was confirmed by qPCR (Fig 5J). Moreover, the levels of p-MLKL and some inflammatory factors in necroptotic area were dramatically decreased by downregulation of Pebp1 (Fig 5K and L).

These data demonstrate that knockdown of Pebp1 prevents liver injury and hepatic necroptosis induced by rAAV injection in *db/db* mice.

Pebp1 mediates rAAV-induced liver injury and hepatic necroptosis in HFD-induced hyperglycemic and obese mice

To further confirm the role of Pebp1 in the development of liver injury and hepatic necroptosis after rAAV injection, HFD-induced hyperglycemic and obese mice were injected with rAAV-si-*NC* or rAAV-si-*Pebp1* (Fig 6A). As expected, after a single injection of rAAV for 2 months, all the mice showed similar body weight and blood glucose levels, while serum ALT and AST activities were significantly decreased in the mice injected with rAAV-si-*Pebp1* compared with those injected with rAAV-si-*NC* (Fig 6B–E). The same mice with high ALT activity had high AST activity (Fig 6D and E), and 4 of the 5 mice with high ALT and AST activities showed hepatic necroptosis (Fig 6F). Both Pebp1 mRNA and protein levels were similar in the high ALT & AST subgroup and low ALT & AST subgroup (Fig EV4C and D). Hepatic morphological and histomorphological analysis showed that downregulation of Pebp1 significantly alleviates rAAV-induced hepatic necroptosis in HFD-induced hyperglycemic and obese mice (Fig 6F). In addition, the levels of p-MLKL and some inflammatory factors increased in necroptotic area

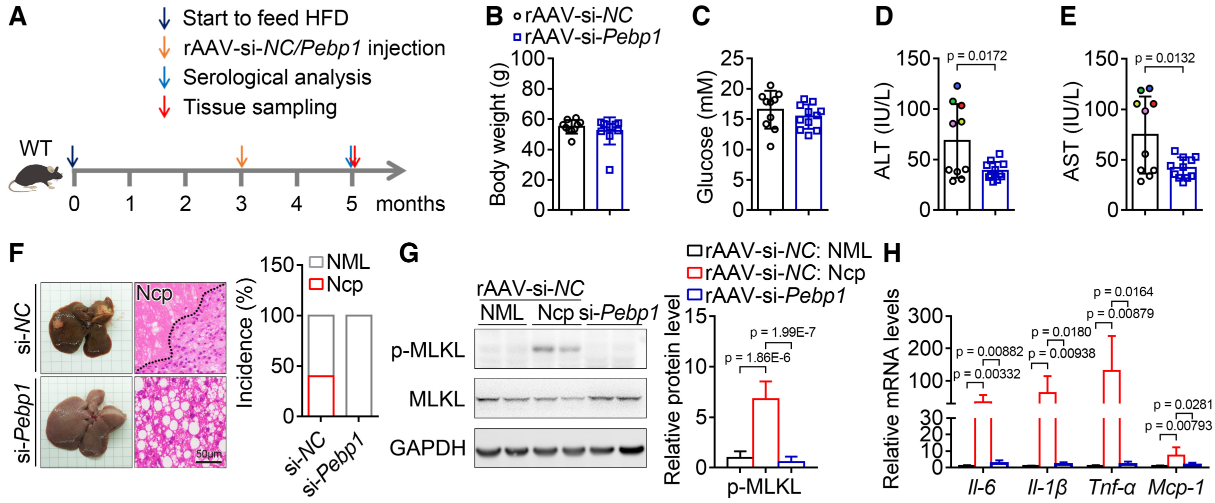

Figure 6. Downregulation of Pebp1 prevents rAAV-induced liver injury and hepatic necroptosis in HFD-induced hyperglycemic and obese mice.

A Schematic of the experimental design for the role of Pebp1 in liver injury and hepatic necroptosis induced by rAAV.

B, C Two months after a single injection of rAAV-si-*Pebp1*, HFD-induced hyperglycemic and obese mice showed similar body weight (B) and blood glucose levels (C) with the mice injected with rAAV-si-*NC*. $n = 10$–11.

D, E Serum ALT (D) and AST (E) activities from mice in (B). The colored dots indicate the mice with high ALT or AST activity, and the same color dot in (D) and (E) indicates the same mouse.

F Liver images and H&E staining of liver sections and the incidence of hepatic necroptosis (Ncp) for mice in (B). NML, Normal.

G The hepatic p-MLKL level of mice in (B).

H The indicated hepatic mRNA levels of mice in (B).

Data information: The experiments for (B), (F) and Fig 3E and J were performed simultaneously, and the rAAV-si-*NC* group and the HFD-rAAV group are the same group. In (B–E, G, H), data are presented as mean ± SD. Student's *t*-test.

Source data are available online for this figure.

© 2023 The Authors

were dramatically decreased by downregulation of Pebp1 (Fig 6G and H).

The observations in *db/db* mice and HFD-induced diabetic and obese mice show that Pebp1 mediates rAAV-induced liver injury and hepatic necroptosis in hyperglycemic and obese mice.

Inhibition of Pebp1 pathway can attenuate inflammation and necroptosis

To investigate the role of Pebp1 in necroptosis pathway, we first analyzed the role of Pebp1 in inflammation. Poly(I:C) was used to mimic virus infection in cell model to induce inflammation as described previously (Liu *et al*, 2019). As shown in Fig 7A, the downregulation of *Pebp1* in mouse primary macrophages by siRNA was confirmed by qPCR, and knockdown of Pebp1 almost completely inhibited the increase of some inflammatory factors induced by poly(I:C) (Fig 7B). Then, we directly analyzed the role of Pebp1 in a necroptosis cell model induced by poly(I:C) and zVAD-fmk. Necroptosis of macrophages monitored by propidium iodide staining and p-MLKL level was significantly alleviated by downregulation of Pebp1 (Fig 7C and D). It has been reported that Pebp1 is essential for Tbk1 activation (Lai *et al*, 2017). As shown in Fig 7E, knockdown of Pebp1 suppressed the elevation of p-Tbk1 induced by poly(I:C). Knockdown of Tbk1 alleviated the increase of some inflammatory factors induced by poly(I:C) (Fig 7F and G). Similarly, necroptosis of macrophages was significantly attenuated by downregulation of Tbk1 (Fig 7H and I). In addition, the role of PEBP1 and TBK1 in necroptosis pathway can also be observed in THP-1 human macrophages (Fig EV5).

To investigate whether Tbk1 mediates rAAV-induced liver injury and hepatic necroptosis, *db/db* mice were injected with rAAV-si-*NC* or rAAV-si-*Tbk1* (Fig 7J). As expected, after a single injection of rAAV for 11 weeks, all the *db/db* mice showed similar body weight, liver weight, and blood glucose levels, while serum ALT and AST activities were significantly decreased in the mice injected with rAAV-si-*Tbk1* compared with those injected with rAAV-si-*NC* (Fig 7K–O). Hepatic morphological and histomorphological analysis showed that downregulation of Tbk1 significantly alleviates rAAV-induced liver necroptosis in *db/db* mice (Fig 7P). The downregulation of *Tbk1* by siRNA was confirmed by qPCR (Fig 7Q). In addition, the levels of p-MLKL and some inflammatory factors increased in necroptotic area were dramatically decreased by downregulation of Tbk1 (Fig 7R and S).

These evidences demonstrate that inhibition of Pebp1 pathway can also attenuate inflammation and necroptosis both *in vitro* and *in vivo*.

Discussion

In this study, we found that rAAV injection induces liver injury, hepatic necroptosis, and carcinoma in diabetic and obese mice, but neither in normal mice nor in mice with only hyperglycemia or obesity. Similarly, it has been reported that diabetes and obesity are considered as important risk factors for HCC (Gallagher & LeRoith, 2015; Lega & Lipscombe, 2020). Moreover, AAV infection is around 30–80% in human population (Erles *et al*, 1999; Halbert *et al*, 2006; Calcedo *et al*, 2009). Combined with our observations, these findings demonstrate that AAV infection is most likely a vital cause leading to HCC in patients with diabetes and obesity. Chronic infection with HBV together with exposure to aflatoxin B1 and infection with HCV together with alcohol use are considered as the dominant risk factors for HCC (Llovet *et al*, 2003). Our findings suggest that AAV infection together with diabetes and obesity is an additional key risk factor for HCC. Early detection of AAV infection and serum ALT/AST activities in patients with diabetes and obesity is highly encouraged to evaluate the risk of HCC, and it is also highly recommended to prevent HCC by keeping serum ALT/AST activities within normal levels in patients with diabetes and obesity infected with AAV.

Here, we show that rAAV injection leads to liver injury, hepatic necroptosis, and carcinoma in diabetic and obese mice, suggesting that AAV gene therapy should be limited to patients without hyperglycemia and obesity. Similarly, tumorigenesis after rAAV injection

Figure 7. Downregulation of Pebp1 signaling alleviates inflammation and necroptosis *in vitro* and attenuates liver injury and hepatic necroptosis in *db/db* mice injected with rAAV. ▶

A The effect of si-*Pebp1* in primary mouse macrophages. $n = 3$.
B Knockdown of Pebp1 markedly blocked the increase of some key inflammatory factors in mouse macrophages treated with poly(I:C). $n = 6$.
C, D Knockdown of Pebp1 markedly blocked the loss of cell membrane integrity (C, $n = 6$ in duplicate or triplicate.) and the increase of p-MLKL level (D, $n = 3$) of mouse macrophages induced by poly(I:C) and z-VAD-fmk.
E Knockdown of Pebp1 attenuated the increase of p-Tbk1 level induced by poly(I:C). $n = 3$ biological replicates.
F The effect of si-*Tbk1* in mouse macrophages. $n = 3$.
G Knockdown of Tbk1 alleviated the increase of some key inflammatory factors in mouse macrophages treated with poly(I:C). $n = 3$.
H, I Knockdown of Tbk1 markedly blocked the loss of cell membrane integrity (H, $n = 8$) and the increase of p-MLKL level (I, $n = 4$) of mouse macrophages induced by poly(I:C) and z-VAD-fmk.
J Schematic of the experimental design for the role of Tbk1 in liver injury and hepatic necroptosis induced by rAAV.
K–M After a single injection of rAAV-si-*Tbk1* for 11 weeks, the *db/db* mice showed similar body weight (K), liver weight (L) and blood glucose levels (M) with the mice injected with rAAV-si-*NC*. $n = 11$.
N, O Serum ALT (N) and AST (O) activities of mice in (K).
P Liver images and H&E staining of liver sections and the incidence of hepatic necroptosis for mice in (K).
Q Relative mRNA level of *Tbk1* in the livers of mice in (K).
R The hepatic p-MLKL level of mice in (K).
S The indicated hepatic mRNA levels of mice in (K).

Data information: In (A–I, K–O, Q–S), data are presented as mean ± SD. Student's *t*-test.
Source data are available online for this figure.

© 2023 The Authors

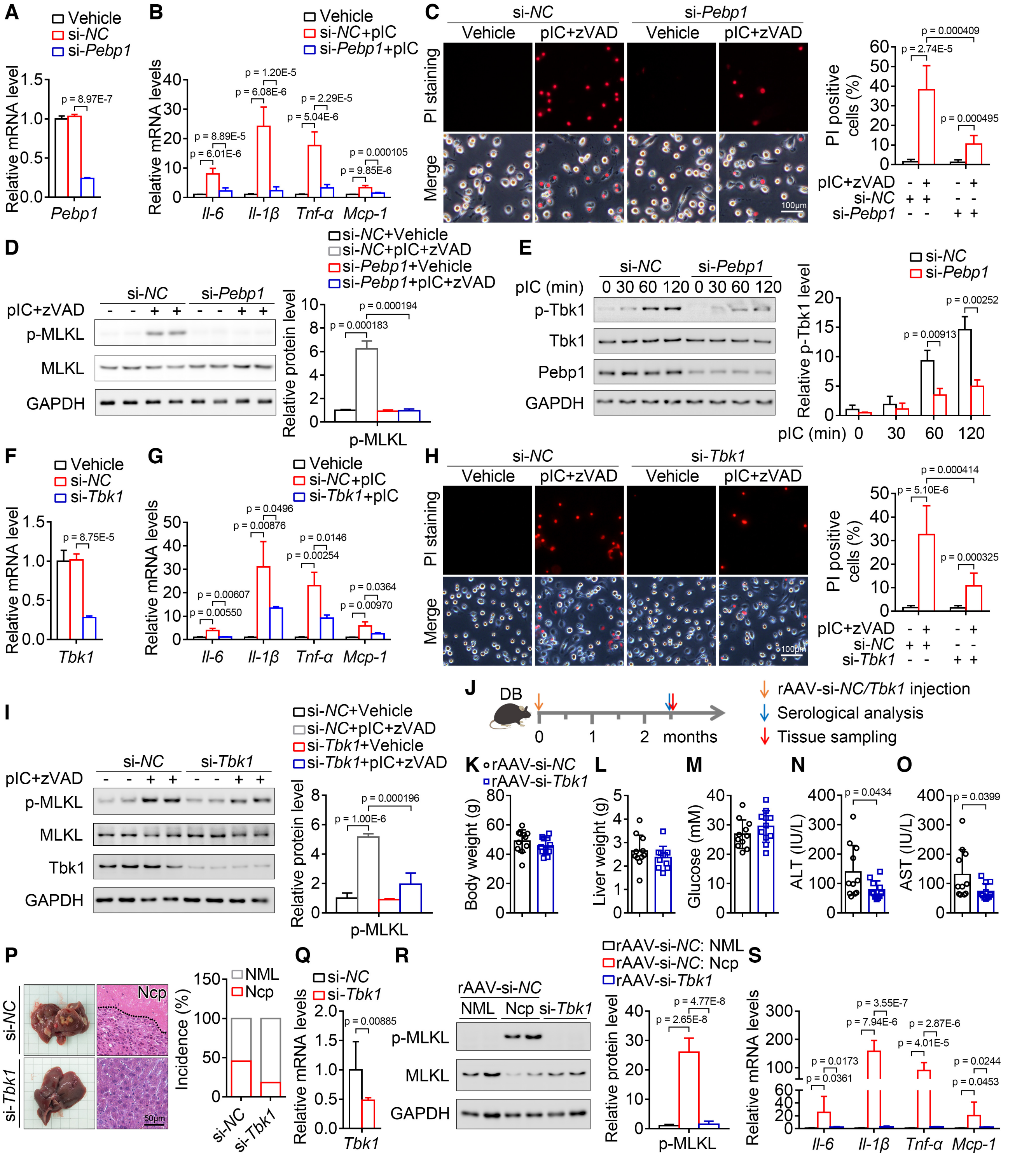

Figure 7.

in mice with mucopolysaccharidosis type VII disease or with ornithine transcarbamylase deficiency was observed (Donsante *et al*, 2001; Bell *et al*, 2006), which also suggest that AAV gene therapy needs to be applied in patients without some specific diseases. In our study, we found that the death of mice with severe liver injury and hepatic necroptosis after rAAV injection for 4–6 weeks in *db/db* mice. Similarly, it has been reported that high dose of rAAV led to liver dysfunction and death of four patients with heavier weight and evidence of pre-existing hepatobiliary disease (Philippidis, 2020, 2021; Shieh *et al*, 2020; Wilson & Flotte, 2020). Since a

lot of clinical trials are ongoing (Wang *et al*, 2019), and clinical applications even have been approved, it has been recommended that patients with liver or kidney dysfunction needs to be excluded as an eligibility criteria for gene therapy (Miesbach *et al*, 2019). According to our findings, whether AAV gene therapy is applicable to patients with hyperglycemia and obesity should be carefully considered.

AAV gene therapy frequently lead to impaired serum AST and/or ALT activities, and which can be resolved with prednisone treatment (Nathwani *et al*, 2014; George *et al*, 2017; Mendell *et al*, 2017). Consistently, we also observed that the prednisone treatment attenuated the increase of AST and ALT activities induced by rAAV injection in *db/db* mice. Moreover, we found that prednisone also alleviated hepatic necroptosis induced by rAAV in *db/db* mice. It has been reported that glucocorticoids are crucial to various physiological processes including inflammation, and prednisone is a synthetic glucocorticoid routinely used in the clinic today for their anti-inflammatory and immunosuppressive effects (Spies *et al*, 2011; Cain & Cidlowski, 2017). Meanwhile, inflammatory factors can induce necroptosis (Newton & Manning, 2016). Combined with our observations, prednisone might block necroptosis by inhibiting inflammation. All these findings show that inhibiting inflammation and necroptosis by prednisone is a promising way to prevent liver dysfunction and subsequent related diseases during AAV gene therapy.

It has been reported that Pebp1 is involved in antiviral innate immunity, and Pebp1 deficiency or knockdown of Pebp1 attenuates inflammatory response (Gu *et al*, 2016; Lai *et al*, 2017). Consistently, we also observed that knockdown of Pebp1 alleviated inflammation both *in vitro* and *in vivo*. Furthermore, we found that knockdown of Pebp1 prevents necroptosis both *in vitro* and *in vivo*, and the downregulation of Pebp1 is likely to block necroptosis through inhibition of inflammation. Pebp1 is essential for Tbk1 activation (Lai *et al*, 2017), and we also found that knockdown of Tbk1 attenuated inflammation and necroptosis both *in vitro* and *in vivo*. These findings provide strong evidence that Pebp1 pathway is essential to necroptosis and targeting Pebp1 pathway is a potential promising strategy to alleviate necroptosis and related diseases.

Taken together, our findings provide strong evidence to consider AAV infection as a critical risk factor for HCC, especially for patients with diabetes and obesity. It should be carefully weighed whether AAV gene therapy is applicable to patients with hyperglycemia and obesity, and treatment with prednisone to alleviate inflammation and necroptosis is a promising strategy to attenuate the side effects during AAV gene therapy. We also identified Pebp1 as a key component in necroptotic pathway and a potential drug target to alleviate necroptosis and related diseases including the side effects of AAV gene therapy.

Materials and Methods

Animals

All animals were maintained and used in accordance with the guidelines of the Institutional Animal Care and Use Committee of Shanghai Institute of Nutrition and Health, Chinese Academy of Sciences (ethics committee approval no. SIBS-2017-ZQW-1, SINH-2021-ZQW-1, SINH-2022-ZQW-1). Male wild-type C57BL/6J, *db/db* and RHH mice were purchased from the Shanghai Laboratory Animals Center (SLAC). The animals were housed in individual cages with free access to a regular chow diet and water in a room at 22 ± 1°C on a 12-h light/dark cycle. The animals presented a healthy status, and male mice were used for all experiments.

Recombinant adeno-associated virus (rAAV) construction and injection

For rAAV2/9 construction, a random sequence TTCTCCGAAC GTGTCACGTAA without any predicted target gene, and the sequence CAGCCTTCCACTATGATATCCTCAA, GAGACAACTC AAGACTCCCATCTTA, CCCAGAATTTGAGAATGACGTGGAA and AGACTATACTAACGAGTTA targeting Cyp4a14, Pebp1, Tat and Tbk1 were synthesized and cloned into pHBAAV-U6-ZsGreen (Hanbio) to obtain pAAV-U6-si-*NC*-ZsGreen, pAAV-U6-si-*Cyp4a14*-ZsGreen, pAAV-U6-si-*Pebp1*-ZsGreen, pAAV-U6-si-*Tat*-ZsGreen and pAAV-U6-si-*Tbk1*-ZsGreen. These constructed plasmids were co-transfected with pAAV-RC (Hanbio) and pHelper (Hanbio) respectively into HEK293T cells to generate rAAV-si-*NC*, rAAV-si-*Cyp4a14*, rAAV-si-*Pebp1*, rAAV-si-*Tat* and rAAV-si-*Tbk1*. Except indicated, rAAV-si-*NC* was mentioned as rAAV and used to study the effect of rAAV. For rAAV2/8 construction, the pAAV-GFP plasmid (Cell Biolabs) was co-transfected with packaging plasmid pHelper and pAAV-8 (Cell Biolabs) into HEK293T cells to generate rAAV2/8. The virus was concentrated by density gradient centrifugation with iodixanol (Sigma, 1343517). Titers of the virus were measured by qPCR with vector-specific primers GTGCACTGTGTTTGCTGACG and GAAAGGAGCTGACAGGTGGT. The viruses were diluted with PBS and administered at a dose of 1×10^{11} vg/mouse through tail vein injection with a volume of 200 µl.

Blood and serum analysis

Blood glucose levels were measured at the indicated time points using tail or heart blood using the FreeStyle blood glucose monitoring system (TheraSense). Serum aspartate transaminase and alanine transaminase were determined using enzymatic assay kits (ShenSuo Unf Medical Diagnostics Articles Co.).

Histological analysis of liver

Liver tissues were excised and fixed with 4% paraformaldehyde in PBS for 24 h and then dehydrated in PBS containing 30% sucrose for at least 16 h. Following paraffin embedding, the tissue sections were stained with hematoxylin and eosin (H&E). Immunohistochemistry was performed as described previously with modifications (Wu *et al*, 2011). Briefly, sections were deparaffinized with xylene and rehydrated. Heat-induced epitope retrieval was performed by placing slides immersed in sodium citrate buffer (pH 6.0) at 100°C for 20 min followed by endogenous peroxidase blocking with 3% H_2O_2 for 30 min. Blocking was performed with 2% (w/v) BSA, and then the sections were incubated with Ki67 antibody (CST, 9449, 1:400) at 4°C overnight. Subsequently, the sections were incubated with Horseradish peroxidase (HRP)-conjugated goat anti-mouse antibody for 2 h at room temperature, and then stained by diaminobenzidine tetrahydrochloride (Gene

 © 2023 The Authors

Tech, GK500710). The sections were counterstained with hematoxylin.

Immunoblotting

Equal volume of cell or liver lysates at the same protein concentrations with a total amount of 20–40 μg protein were separated by SDS–PAGE, transferred to polyvinylidene fluoride (PVDF) membranes (Merck Millipore), blocked and detected with antibodies against mouse p-MLKL (S345; Abcam, Ab196436, 1:1,000), mouse MLKL (CST, 37705, 1:1,000), human p-MLKL (S358; Abcam, Ab187091, 1:1,000), human MLKL (Abcam, Ab184718, 1:1,000), PEBP1 (Santa Cruz Biotechnology, sc-376925, 1:2,000), p-TBK1 (CST, 5483, 1:1,000), TBK1 (CST, 38066, 1:1,000), cleaved Caspase-3 (CST, 9661, 1:1,000), Caspase-3 (CST, 9662, 1:1,000), cleaved Caspase-8 (CST, 9429, 1:1,000), Caspase-8 (CST, 4927, 1:1,000), and GAPDH (CST, 2118, 1:5,000). The immune complexes were detected using a horseradish peroxidase-conjugated secondary antibody and visualized with a chemiluminescence reagent (Thermo). Protein quantification was analyzed by PhotoShop, and normalized to GAPDH.

RNA isolation and quantitative PCR

Total RNA extracted with TRIzol reagent was reverse transcribed using M-MLV Reverse Transcriptase (Promega) with random hexamer primers. Quantitative PCR was performed using FastStart Universal SYBR Green Master (Roche) on an ABI Prism 7900 Sequence Detection System. The primers GTGCCGCCTGGAGAAA CCT and TGAAGTCGCAGGAGACAACC were used to detect mouse *Gapdh*; CCCAACTGGTACATCAGCAC and TCTGCTCATTCA CGAAAAGG for mouse *Il-1β*; CCCACTCTGACCCCTTTACT and TTTGAGTCCTTGATGGTGGT for mouse *Tnf-α*; CGGAGAGGAGA CTTCACAGA and CCAGTTTGGTAGCATCCATC for mouse *Il-6*; TTAAAAACCTGGATCGGAACCAA and GCATTAGCTTCAGATTTA CGGGT for mouse *Mcp-1*; TGAATTGCTGCCAGATCCCAC and GTTCAGTGGCTGGTCAGAGTT for mouse *Cyp4a14*; ATAGACCCAC-CAGCATTTCGT and GTAAACCAGCCAGACATAGCG for mouse *Pebp1*; ACCTTCAATCCCATCCGA and TCCCGACTGGATAGGTAG for mouse *Tat*; TATCTTTGTCACGAGCCGGG and AACCAGTTCAA CCAGCCACC for mouse *Tbk1*. The gene expression levels were normalized to mouse *Gapdh*. The primers GCGAGAAGATGACCCAGA TCAT and GCTCAGGAGGAGCAATGATCTT were used to detect human *ACTB*; AGATGATAAGCCCACTCTACAGC and CTTTAAGT GAGTAGGAGAGGTGAG for human *IL-1β*; AGATGATCTGACTGCCT GGG and CTGCTGCACTTTGGAGTGAT for human *TNF-α*; GGT ACATCCTCGACGGCATCT and GTGCCTCTTTGCTGCTTTCAC for human *IL-6*; CAGCCAGATGCAATCAATGCC and TGGAATCCT GAACCCACTTCT for human *MCP-1*; CTACACCTTGGTCCTGACAGA and GAGCCCACATAATCGGAGAGG for human *PEBP1*; TTGCAG TCTTTCTCGGGGTC and ACTGGTCAAAACCCCAACACT for human *TBK1*. The gene expression levels were normalized to human *ACTB*.

Streptozotocin-induced hyperglycemia mouse model

Male wild-type mice were injected intraperitoneally with 40 mg/kg/day STZ (Sigma-Aldrich) or an equivalent volume of vehicle for 5 consecutive days at the age of 9-week-old as described previously (Cheng *et al*, 2019). The mice with a blood glucose level > 13.8 mM were used for the following experiments.

Measurement of hepatic glycogen content

Hepatic glycogen content was measured using a glycogen content assay kit (Sangon Biotech).

HFD-induced obesity mouse model

Five-week-old wild-type mice were randomly assigned and fed with either normal chow diet containing 10 kcal% fat or high-fat diet containing 40 kcal% fat and 40 kcal% sucrose (Research Diets D12327). Fat mass was measured in conscious animals by a minispec mq serial NMR spectrometer (Bruker). After fed with high-fat diet for 12 weeks, the mice with a fat mass percentage > 30% and a blood glucose level > 13.8 mM were considered both hyperglycemia and obesity, and used for the following experiments. Injection with PBS, AAV-si-NC or AAV-si-*Pebp1* was performed simultaneously in these hyperglycemic and obese mice. Five-week-old RHH mice were randomly assigned and fed with either normal chow containing 10 kcal% fat or high-fat diet containing 60 kcal% fat (Research Diets). After fed for 12 weeks, the mice with a fat mass percentage > 30% were considered obesity.

Oral administration of prednisone

The chow diet was ground and mixed with the powder of prednisone at the indicated concentrations, and then was pelleted for the following studies. The *db/db* mice at the age of 9 weeks were injected with AAV at a dose of 1×10^{11} vg/mouse and randomly assigned to feed a chow diet without or with 4.3 mg/kg/day prednisone for 2 months. The liver function of the mice was monitored by measurement of serum aspartate transaminase and alanine transaminase.

High-throughput RNA-Sequencing (RNA-Seq)

Liver samples were used to extract total RNA for the following RNA-Seq. Completed libraries were generated and sequenced with 150-bp paired-end reads on Illumina HiSeq 4000. The top 15 hepatic genes upregulated in 9-week-old *db/db* mice compared with wild-type mice ranked by adjusted *P*-value with fold change > 3 and rpm > 100 in wild-type mice were selected for the following analysis. Genes having adjusted *P*-value ≤ 0.05 and abs (log2 (fold change)) ≥ 1 were considered as differentially expressed. The differentially expressed genes were further analyzed by KEGG (Kyoto Encyclopedia of Genes and Genomes) and GSEA (Gene Set Enrichment Analysis).

Mouse peritoneal macrophage isolation, culture, and treatment

Mouse peritoneal macrophages were obtained from male wild-type mice at the age of 6–8 weeks as described previously (Zhao *et al*, 2017). Briefly, mice were intraperitoneally injected with 2–3 ml 5% dehydrated thioglycolate medium (BBL) in PBS. After 3–4 days, the mice were killed, and 3–4 ml of RPMI 1640 medium was injected intraperitoneally. The mice were gently rubbed on the abdomen to

© 2023 The Authors

make the medium flow well in the abdomen, and then an incision was made on the abdomen, and the liquid was aspirated from the abdomen with a 5-ml syringe. After precipitation by centrifugation, washing and counting, peritoneal macrophages were cultured in RPMI 1640 containing 10% FBS at 37°C with 5% CO_2. Mouse peritoneal macrophages were treated by 20 μg/ml poly(I:C) (Sigma) with or without 20 μM z-VAD-fmk (MCE) for 8 h, then used for RNA isolation, propidium iodide staining or immunoblotting. For treatment with prednisone, cells were treated with 40 μM prednisone (MCE) for 1 h, then 20 μg/ml poly(I:C) with or without 20 μM z-VAD-fmk were added and incubated for 3 h. Subsequently, additional 40 μM prednisone was supplemented to compensate its degradation. The cells were harvested for immunoblotting after treatment with poly(I:C) and z-VAD-fmk for 6 h, and used for RNA isolation and propidium iodide staining after treatment with poly(I:C) with or without z-VAD-fmk for 8 h.

Human THP-1 cell culture and treatment

THP-1 human monocytes were obtained from Cell Bank of Chinese Academy of Sciences and were tested about monthly for mycoplasma contamination. THP-1 monocytes were cultured in RPMI 1640 containing 10% FBS and 50 μM β-mercaptoethanol at 37°C with 5% CO_2, and treated with 100 ng/ml phorbol 12-myristate 13-acetate (PMA; Sigma) for 24 h to induce the differentiation to macrophages. After culture in RPMI 1640 containing 10% FBS for another 24 h, the THP-1 macrophages were transfected with the indicated siRNAs. After transfected with siRNAs for 48 h, the cells were treated with 40 μg/ml poly(I:C) for 8 h to isolate RNA or treated with 40 μg/ml poly(I:C) and 40 μM z-VAD-fmk for 24 h for propidium iodide staining or immunoblotting.

Transfection of siRNAs

Mouse peritoneal macrophages were transfected with si-*NC* (sense: 5′-UUCUCCGAACGUGUCACGUAAdTdT-3′, antisense: 5′-UUACGUGACACGUUCGGAGAAdTdT-3′) or si-*Pebp1* (sense: 5′-GAGACAACUCAAGACUCCCAUCUUAdTdT-3′, antisense: 5′-UAAGAUGGGAGUCUUGAGUUGUCUCdTdT-3′) or si-*Tbk1* (sense: 5′-AGACUAUACUAACGAGUUAdTdT-3′, antisense: 5′-UAACUCGUUAGUAUAGUAUdTdT-3′) Human THP-1 cells were transfected with si-*NC* (sense: 5′-UUCUCCGAACGUGUCACGUAAdTdT-3′, antisense: 5′-UUACGUGACACGUUCGGAGAAdTdT-3′) or si-*PEBP1* (sense: 5′-UGGUCAACAUGAAGGGUAAdTdT-3′, antisense: 5′-UUACCCUUCAUGUUGACCAdTdT-3′) or si-*TBK1* (sense: 5′-CCUCUGAAUACCAUAGGAUdTdT-3′, antisense: 5′-AUCCUAUGGUAUUCAGAGGdTdT-3′). All the siRNAs were transfected at a dose of 50 nM using Lipofectamine 3000 (Thermo Fisher Scientific). After transfection for 36 h for mouse macrophages or 48 h for human macrophages, cells were treated as indicated and harvested for RNA isolation. After transfection for 48 h, cells were treated as indicated for propidium iodide staining and immunoblotting.

Propidium iodide staining

Cells were stained with 5 μg/ml propidium iodide (Beyotime Biotech) in PBS for 5–10 min and visualized under a fluorescent microscope.

The paper explained

Problem

Obesity and diabetes are important risk factors for tumorigenesis, including liver cancer. Adeno-associated virus (AAV), widely used for gene therapy, is found in around 30–80% of the population. However, whether environmental or therapeutical AAV infection is a critical risk factor for liver cancer in individuals with obesity and diabetes is yet to be elucidated.

Results

rAAV injection leads to liver injury, hepatic necroptosis, and liver cancer in hyperglycemic and obese mice, but not in hyperglycemic and slim mice or euglycemic and obese mice. Prednisone administration markedly alleviated liver injury and hepatic necroptosis in hyperglycemic and obese mice. Inhibition of Pebp1/Tbk1 signaling also attenuated liver injury, hepatic necroptosis, and subsequent liver cancer caused by rAAV injection in hyperglycemic and obese mice.

Impact

Environmental AAV infection or AAV gene therapy for individuals with hyperglycemia and obesity should be carefully evaluated. Both prednisone treatment and targeting Pebp1/Tbk1 signaling are promising strategies to prevent or treat AAV-induced liver injury, hepatic necroptosis, and related diseases including liver cancer.

Statistics

Animals were randomized into different treatment groups. No mice were excluded from all the experiments. Propidium iodide positive cells were counted blindly.

Data are expressed as mean ± SD of at least three independent experiments. Statistical significance was assessed by two-tailed unpaired Student's *t*-test. Differences were considered statistically significant at $P < 0.05$.

Data availability

The RNA-sequencing data generated and/or analyzed during this study are available in the National Center for Biotechnology Information Sequence Read Archive repository (accession number PRJNA762256 (https://www.ncbi.nlm.nih.gov/bioproject/PRJNA762256), PRJNA867031 (https://www.ncbi.nlm.nih.gov/bioproject/PRJNA867031)).

Expanded View for this article is available online.

Acknowledgements

This work was supported by National Key R&D Program of China (2018YFA0800603), National Natural Science Foundation of China (32200969, 31630037, 91740103, 91940306), Strategic Priority Research Program of the Chinese Academy of Sciences (XDB19030103), Frontier Science of Chinese Academy of Sciences Key Research Projects (QYZDJ-SSW-SMC022), Shanghai Leading Talents Program, Shanghai Post-doctoral Excellence Program and China Postdoctoral Science Foundation Grant (2019M661658). We would like to thank Yifan Bu, Zhonghui Weng and Aimin Huang from the Institutional Center for Shared Technologies and Facilities of SINH, CAS for technical assistance.

© 2023 The Authors

Author contributions

Yalan Cheng: Conceptualization; data curation; formal analysis; funding acquisition; validation; investigation; visualization; methodology; writing – original draft; project administration; writing – review and editing. **Zhentong Zhang:** Data curation; formal analysis; validation; investigation; visualization; methodology; project administration; writing – review and editing. **Peidong Gao:** Formal analysis; investigation; writing – review and editing. **Hejin Lai:** Software; formal analysis; writing – review and editing. **Wuling Zhong:** Investigation; writing – review and editing. **Ning Feng:** Investigation; methodology; writing – review and editing. **Yale Yang:** Investigation; writing – review and editing. **Huimin Yu:** Investigation; writing – review and editing. **Yali Zhang:** Investigation; methodology; writing – review and editing. **Yumo Han:** Investigation; methodology; writing – review and editing. **Jieya Dong:** Investigation; methodology; writing – review and editing. **Zhishui He:** Formal analysis; investigation; writing – review and editing. **Rui Huang:** Investigation; methodology; writing – review and editing. **Qiwei Zhai:** Conceptualization; resources; formal analysis; supervision; funding acquisition; investigation; visualization; writing – review and editing.

Disclosure and competing interests statement

The authors declare that they have no conflict of interest.

References

Bell P, Moscioni AD, McCarter RJ, Wu D, Gao GP, Hoang A, Sanmiguel JC, Sun X, Wivel NA, Raper SE *et al* (2006) Analysis of tumors arising in male B6C3F1 mice with and without AAV vector delivery to liver. *Mol Ther* 14: 34 – 44

Cain DW, Cidlowski JA (2017) Immune regulation by glucocorticoids. *Nat Rev Immunol* 17: 233 – 247

Calcedo R, Vandenberghe LH, Gao G, Lin J, Wilson JM (2009) Worldwide epidemiology of neutralizing antibodies to adeno-associated viruses. *J Infect Dis* 199: 381 – 390

Chandler RJ, LaFave MC, Varshney GK, Trivedi NS, Carrillo-Carrasco N, Senac JS, Wu W, Hoffmann V, Elkahloun AG, Burgess SM *et al* (2015) Vector design influences hepatic genotoxicity after adeno-associated virus gene therapy. *J Clin Invest* 125: 870 – 880

Chen CL, Yang HI, Yang WS, Liu CJ, Chen PJ, You SL, Wang LY, Sun CA, Lu SN, Chen DS *et al* (2008) Metabolic factors and risk of hepatocellular carcinoma by chronic hepatitis B/C infection: a follow-up study in Taiwan. *Gastroenterology* 135: 111 – 121

Cheng Y, Liu J, Luan Y, Liu Z, Lai H, Zhong W, Yang Y, Yu H, Feng N, Wang H *et al* (2019) Sarm1 gene deficiency attenuates diabetic peripheral neuropathy in mice. *Diabetes* 68: 2120 – 2130

Donsante A, Vogler C, Muzyczka N, Crawford JM, Barker J, Flotte T, Campbell-Thompson M, Daly T, Sands MS (2001) Observed incidence of tumorigenesis in long-term rodent studies of rAAV vectors. *Gene Ther* 8: 1343 – 1346

Donsante A, Miller DG, Li Y, Vogler C, Brunt EM, Russell DW, Sands MS (2007) AAV vector integration sites in mouse hepatocellular carcinoma. *Science* 317: 477

El-Serag HB (2012) Epidemiology of viral hepatitis and hepatocellular carcinoma. *Gastroenterology* 142: 1264 – 1273

Erles K, Sebokova P, Schlehofer JR (1999) Update on the prevalence of serum antibodies (IgG and IgM) to adeno-associated virus (AAV). *J Med Virol* 59: 406 – 411

Fu L, Dong SS, Xie YW, Tai LS, Chen L, Kong KL, Man K, Xie D, Li Y, Cheng Y *et al* (2010) Down-regulation of tyrosine aminotransferase at a frequently deleted region 16q22 contributes to the pathogenesis of hepatocellular carcinoma. *Hepatology* 51: 1624 – 1634

Gabriela-Freitas M, Pinheiro J, Raquel-Cunha A, Cardoso-Carneiro D, Martinho O (2019) RKIP as an inflammatory and immune system modulator: implications in cancer. *Biomolecules* 9: 769

Gallagher EJ, LeRoith D (2015) Obesity and diabetes: the increased risk of cancer and cancer-related mortality. *Physiol Rev* 95: 727 – 748

George LA, Sullivan SK, Giermasz A, Rasko JEJ, Samelson-Jones BJ, Ducore J, Cuker A, Sullivan LM, Majumdar S, Teitel J *et al* (2017) Hemophilia B gene therapy with a High-specific-activity factor IX variant. *N Engl J Med* 377: 2215 – 2227

Gu M, Liu Z, Lai R, Liu S, Lin W, Ouyang C, Ye S, Huang H, Wang X (2016) RKIP and TBK1 form a positive feedback loop to promote type I interferon production in innate immunity. *EMBO J* 35: 2553 – 2565

Halbert CL, Miller AD, McNamara S, Emerson J, Gibson RL, Ramsey B, Aitken ML (2006) Prevalence of neutralizing antibodies against adeno-associated virus (AAV) types 2, 5, and 6 in cystic fibrosis and normal populations: implications for gene therapy using AAV vectors. *Hum Gene Ther* 17: 440 – 447

Hwang S, Park YM, Han KD, Yun JS, Ko SH, Ahn YB, Han JH (2021) Associations of general obesity and central obesity with the risk of hepatocellular carcinoma in a Korean population: a national population-based cohort study. *Int J Cancer* 148: 1144 – 1154

Kaiser J (2020) How safe is a popular gene therapy vector? *Science* 367: 131

Keeler AM, Flotte TR (2019) Recombinant adeno-associated virus gene therapy in light of Luxturna (and Zolgensma and Glybera): where are we, and how did we get here? *Annu Rev Virol* 6: 601 – 621

Koh WP, Wang R, Jin A, Yu MC, Yuan JM (2013) Diabetes mellitus and risk of hepatocellular carcinoma: findings from the Singapore Chinese health study. *Br J Cancer* 108: 1182 – 1188

La Vecchia C, Negri E, Decarli A, Franceschi S (1997) Diabetes mellitus and the risk of primary liver cancer. *Int J Cancer* 73: 204 – 207

Lai R, Gu M, Jiang W, Lin W, Xu P, Liu Z, Huang H, An H, Wang X (2017) Raf kinase inhibitor protein preferentially promotes TLR3-triggered signaling and inflammation. *J Immunol* 198: 4086 – 4095

Lega IC, Lipscombe LL (2020) Review: diabetes, obesity, and cancer-pathophysiology and clinical implications. *Endocr Rev* 41: 33 – 52

Li C, Samulski RJ (2020) Engineering adeno-associated virus vectors for gene therapy. *Nat Rev Genet* 21: 255 – 272

Liu CX, Li X, Nan F, Jiang S, Gao X, Guo SK, Xue W, Cui Y, Dong K, Ding H *et al* (2019) Structure and degradation of circular RNAs regulate PKR activation in innate immunity. *Cell* 177: 865 – 880

Llovet JM, Burroughs A, Bruix J (2003) Hepatocellular carcinoma. *Lancet* 362: 1907 – 1917

Mantovani A, Targher G (2017) Type 2 diabetes mellitus and risk of hepatocellular carcinoma: spotlight on nonalcoholic fatty liver disease. *Ann Transl Med* 5: 270

Marengo A, Rosso C, Bugianesi E (2016) Liver cancer: connections with obesity, fatty liver, and cirrhosis. *Annu Rev Med* 67: 103 – 117

Mendell JR, Al-Zaidy S, Shell R, Arnold WD, Rodino-Klapac LR, Prior TW, Lowes L, Alfano L, Berry K, Church K *et al* (2017) Single-dose gene-replacement therapy for spinal muscular atrophy. *N Engl J Med* 377: 1713 – 1722

Miesbach W, O'Mahony B, Key NS, Makris M (2019) How to discuss gene therapy for haemophilia? A patient and physician perspective. *Haemophilia* 25: 545 – 557

Nathwani AC, Reiss UM, Tuddenham EG, Rosales C, Chowdary P, McIntosh J, Della Peruta M, Lheriteau E, Patel N, Raj D *et al* (2014) Long-term safety and efficacy of factor IX gene therapy in hemophilia B. *N Engl J Med* 371: 1994 – 2004

© 2023 The Authors

Nault JC, Datta S, Imbeaud S, Franconi A, Mallet M, Couchy G, Letouze E, Pilati C, Verret B, Blanc JF *et al* (2015) Recurrent AAV2-related insertional mutagenesis in human hepatocellular carcinomas. *Nat Genet* 47: 1187 – 1193

Newton K, Manning G (2016) Necroptosis and inflammation. *Annu Rev Biochem* 85: 743 – 763

Nguyen GN, Everett JK, Kafle S, Roche AM, Raymond HE, Leiby J, Wood C, Assenmacher CA, Merricks EP, Long CT *et al* (2021) A long-term study of AAV gene therapy in dogs with hemophilia a identifies clonal expansions of transduced liver cells. *Nat Biotechnol* 39: 47 – 55

N'Kontchou G, Paries J, Htar MT, Ganne-Carrie N, Costentin L, Grando-Lemaire V, Trinchet JC, Beaugrand M (2006) Risk factors for hepatocellular carcinoma in patients with alcoholic or viral C cirrhosis. *Clin Gastroenterol Hepatol* 4: 1062 – 1068

Ozelo MC, Mahlangu J, Pasi KJ, Giermasz A, Leavitt AD, Laffan M, Symington E, Quon DV, Wang JD, Peerlinck K *et al* (2022) Valoctocogene Roxaparvovec gene therapy for hemophilia A. *N Engl J Med* 386: 1013 – 1025

Parkin DM (2006) The global health burden of infection-associated cancers in the year 2002. *Int J Cancer* 118: 3030 – 3044

Pasparakis M, Vandenabeele P (2015) Necroptosis and its role in inflammation. *Nature* 517: 311 – 320

Pearson JS, Giogha C, Muhlen S, Nachbur U, Pham CL, Zhang Y, Hildebrand JM, Oates CV, Lung TW, Ingle D *et al* (2017) EspL is a bacterial cysteine protease effector that cleaves RHIM proteins to block necroptosis and inflammation. *Nat Microbiol* 2: 16258

Philippidis A (2020) After third death, Audentes' AT132 remains on clinical hold. *Hum Gene Ther* 31: 908 – 910

Philippidis A (2021) Fourth boy dies in clinical trial of Astellas' AT132. *Hum Gene Ther* 32: 1008 – 1010

Philippidis A (2022) After patient death, FDA places hold on Pfizer Duchenne muscular dystrophy gene therapy trial. *Hum Gene Ther* 33: 111 – 115

Plaz Torres MC, Jaffe A, Perry R, Marabotto E, Strazzabosco M, Giannini EG (2022) Diabetes medications and risk of HCC. *Hepatology* 76: 1880 – 1897

Russell DW, Kay MA (1999) Adeno-associated virus vectors and hematology. *Blood* 94: 864 – 874

Samanic C, Chow WH, Gridley G, Jarvholm B, Fraumeni JF Jr (2006) Relation of body mass index to cancer risk in 362,552 Swedish men. *Cancer Causes Control* 17: 901 – 909

Schlesinger S, Aleksandrova K, Pischon T, Fedirko V, Jenab M, Trepo E, Boffetta P, Dahm C, Overvad K, Tjønneland A *et al* (2013) Abdominal obesity, weight gain during adulthood and risk of liver and biliary tract cancer in a European cohort. *Int J Cancer* 132: 645 – 657

Shieh PB, Bonnemann CG, Muller-Felber W, Blaschek A, Dowling JJ, Kuntz NL, Seferian AM (2020) Re: "moving forward after two deaths in a gene therapy trial of Myotubular myopathy" by Wilson and Flotte. *Hum Gene Ther* 31: 787

Spies CM, Cutolo M, Straub RH, Burmester GR, Buttgereit F (2011) Prednisone chronotherapy. *Clin Exp Rheumatol* 29: S42 – S45

Sun CA, Wu DM, Lin CC, Lu SN, You SL, Wang LY, Wu MH, Chen CJ (2003) Incidence and cofactors of hepatitis C virus-related hepatocellular carcinoma: a prospective study of 12,008 men in Taiwan. *Am J Epidemiol* 157: 674 – 682

Teng W, Liu YC, Jeng WJ, Su CW (2021) Tertiary prevention of HCC in chronic hepatitis B or C infected patients. *Cancer* 13: 1729

Valdmanis PN, Lisowski L, Kay MA (2012) rAAV-mediated tumorigenesis: still unresolved after an AAV assault. *Mol Ther* 20: 2014 – 2017

Vasileva A, Jessberger R (2005) Precise hit: adeno-associated virus in gene targeting. *Nat Rev Microbiol* 3: 837 – 847

Wang D, Tai PWL, Gao GP (2019) Adeno-associated virus vector as a platform for gene therapy delivery. *Nat Rev Drug Discov* 18: 358 – 378

Wilson JM, Flotte TR (2020) Moving forward after two deaths in a gene therapy trial of Myotubular myopathy. *Hum Gene Ther* 31: 695 – 696

Wu J, Zhang F, Yan M, Wu D, Yu Q, Zhang Y, Zhou B, McBurney MW, Zhai Q (2011) WldS enhances insulin transcription and secretion via a SIRT1-dependent pathway and improves glucose homeostasis. *Diabetes* 60: 3197 – 3207

Zhang X, Li S, Zhou Y, Su W, Ruan X, Wang B, Zheng F, Warner M, Gustafsson JA, Guan Y (2017) Ablation of cytochrome P450 omega-hydroxylase 4A14 gene attenuates hepatic steatosis and fibrosis. *Proc Natl Acad Sci USA* 114: 3181 – 3185

Zhao Q, Yu X, Zhang H, Liu Y, Zhang X, Wu X, Xie Q, Li M, Ying H, Zhang H (2017) RIPK3 mediates necroptosis during embryonic development and postnatal inflammation in Fadd-deficient mice. *Cell Rep* 19: 798 – 808

License: This is an open access article under the terms of the Creative Commons Attribution License, which permits use, distribution and reproduction in any medium, provided the original work is properly cited.

© 2023 The Authors

Expanded View Figures

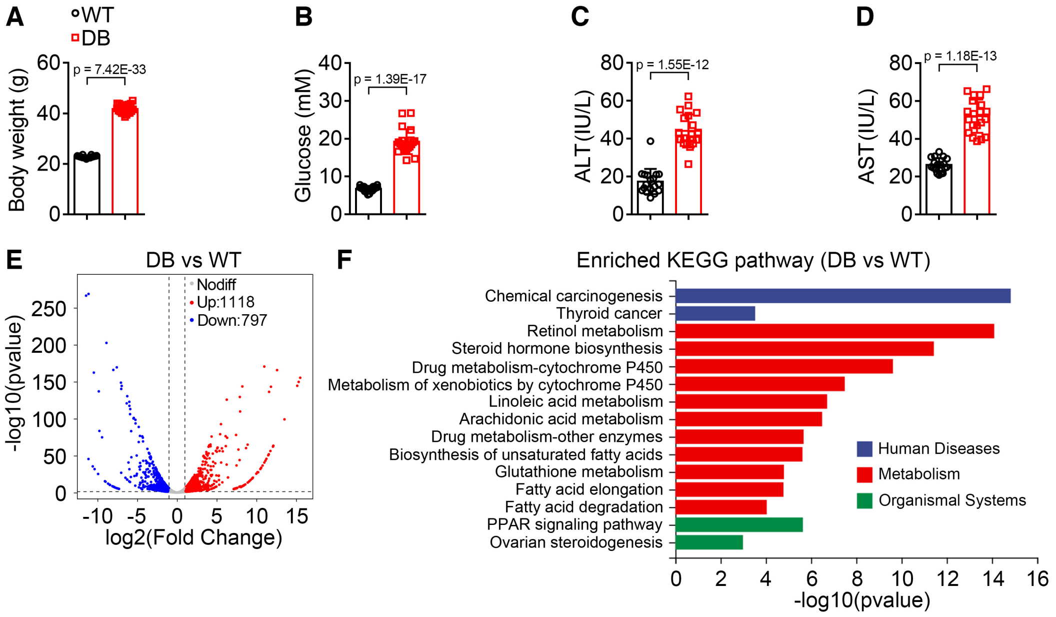

Figure EV1. Body weight, blood glucose levels, and serum ALT and AST activities, hepatic transcriptomic analysis of wild type and *db/db* mice.

A, B Body weight (A) and blood glucose levels (B) were significantly increased in *db/db* mice. $n = 18$–20 for each group.
C, D Serum alanine aminotransferase (ALT) (C) and aspartate aminotransferase (AST) (D) activities were significantly elevated in *db/db* mice. $n = 18$–20 for each group.
E Scatter plot comparing the differentially expressed genes detected by RNA-seq in liver of wild-type and *db/db* mice.
F The top 15 significantly enriched KEGG pathways of the differentially expressed genes in (E).

Data information: In (A–D), data are presented as mean ± SD. Student's *t*-test.

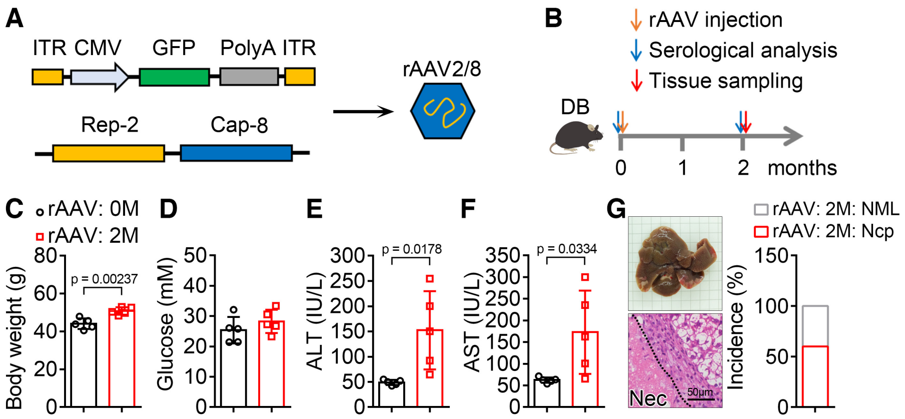

Figure EV2. Injection of rAAV serotype 2/8 can also lead to liver injury and necroptosis in *db/db* mice.

A, B Schematic of rAAV serotype 2/8 (rAAV2/8) package (A) and the experimental design for the effect of rAAV2/8 injection on liver function and hepatic necroptosis (B). ITR, AAV2 ITR.
C–F Two months after a single injection of rAAV2/8, *db/db* mice showed similar blood glucose levels (D), and increased body weight (C), serum ALT (E) and AST (F) activities compared with those before injection of rAAV2/8. $n = 5$.
G Liver images, H&E staining of liver sections and the incidence of hepatic necroptosis (Ncp) for mice in (C). NML, Normal.

Data information: In (C–F), data are presented as mean ± SD. Student's *t*-test.

© 2023 The Authors

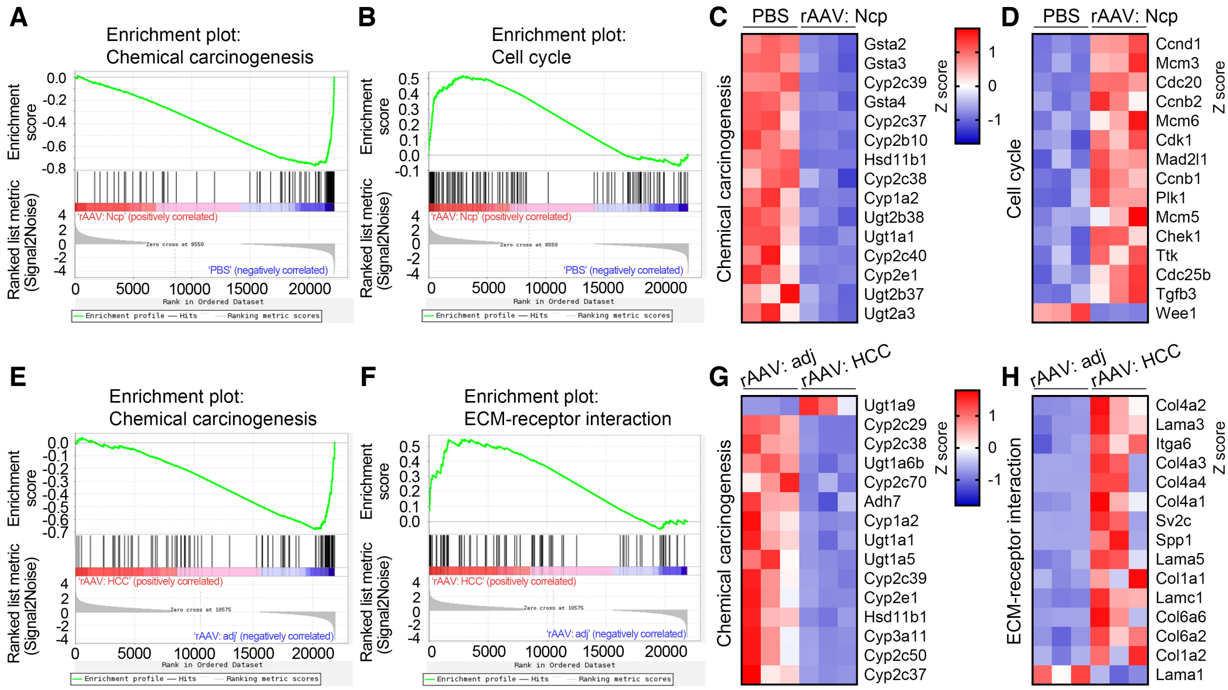

Figure EV3. GSEA analysis and heatmaps of the top 15 differentially expressed genes in the indicated enriched KEGG pathways.

A, B GSEA analysis of the indicated enriched KEGG pathways of the differentially expressed genes in normal or necroptotic area from the liver of mice after a single injection of PBS or rAAV for 2 months. Ncp, necroptosis.

C, D Heatmaps of the top 15 differentially expressed genes ranked by adjusted *P* value in the indicated KEGG pathways in (A) and (B).

E, F GSEA analysis of the indicated enriched KEGG pathways of the differentially expressed genes in adjacent or HCC area from the liver of mice after a single injection of rAAV for 6 months. adj, adjacent.

G, H Heatmaps of the top 15 differentially expressed genes ranked by adjusted *P* value in the indicated KEGG pathways in (E) and (F).

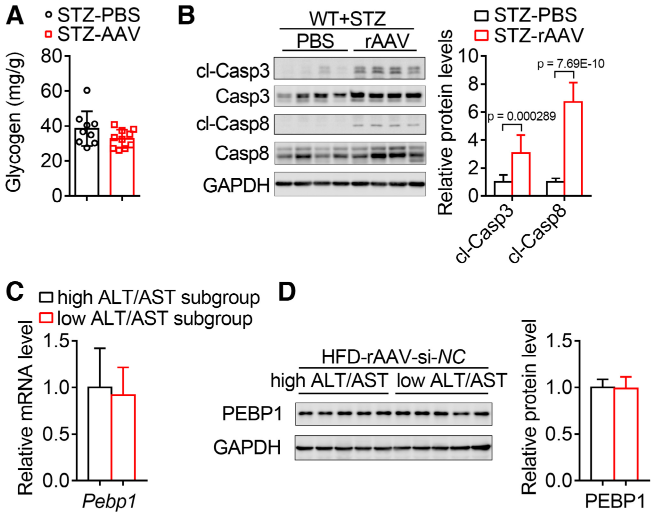

Figure EV4. Effect of rAAV injection on hepatic glycogen content and apoptosis in hyperglycemic mice and on endogenous Pebp1 level in liver of hyperglycemic and obese mice.

A Two months after a single injection of rAAV, hyperglycemic mice induced by streptozotocin showed similar glycogen content compared with those mice injected with PBS. $n = 9–10$.

B The hepatic protein levels of cleaved Caspase 3 (cl-Casp3) and cleaved Caspase 8 (cl-Casp8) in the livers of mice in (A) were measured to monitor apoptosis. $n = 9–10$.

C The hepatic mRNA level of *Pebp1* were similar in the high ALT & AST subgroup and low ALT & AST subgroup from HFD-induced hyperglycemic and obese mice injected with rAAV-si-*NC* as shown in Fig 6D and E. $n = 5$ biological replicates.

D The hepatic protein level of Pebp1 from mice in (C).

Data information: In (A–D), data are presented as mean ± SD. Student's *t*-test.

 © 2023 The Authors

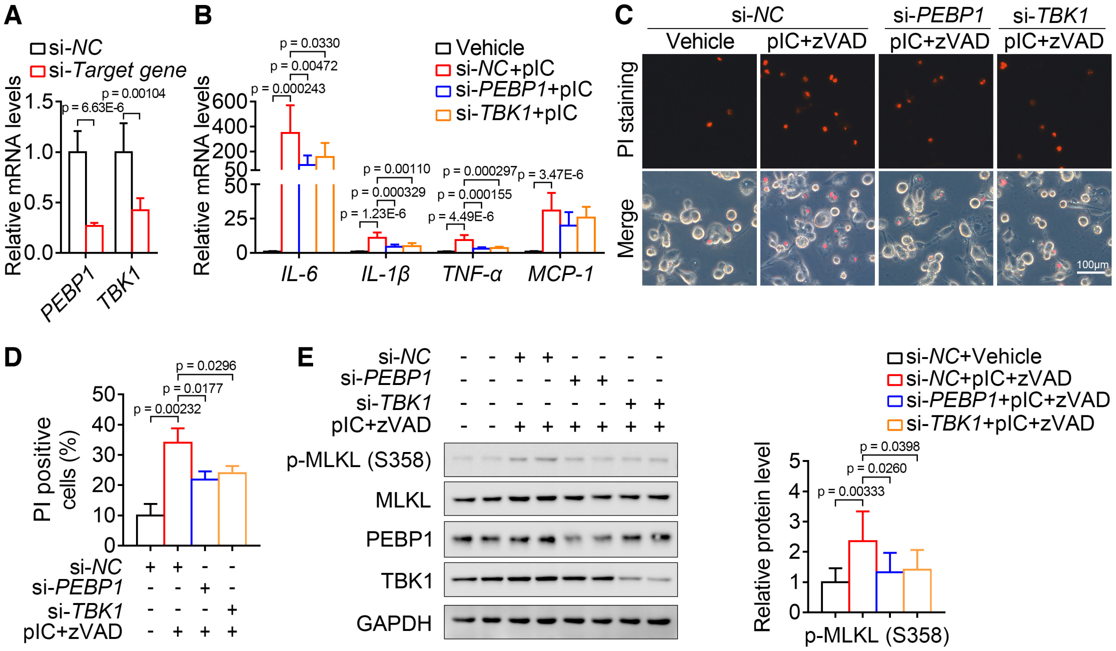

Figure EV5. Suppression of PEBP1 signaling alleviates inflammation and necroptosis in human macrophages.

A The effect of si-*PEBP1* and si-*TBK1* in THP-1 human macrophages. $n = 6$ biological replicates.

B Knockdown of PEBP1 and TBK1 markedly blocked the increase of some key inflammatory factors in THP-1 macrophages treated with poly(I:C). $n = 9$ biological replicates.

C–E Knockdown of PEBP1 and TBK1 markedly blocked the loss of cell membrane integrity (C-D, $n = 3$ in triplicate) and the increase of p-MLKL level (E, $n = 8$ biological replicates) of THP-1 macrophages induced by poly(I:C) and z-VAD-fmk.

Data information: In (A, B, D, E), data are presented as mean ± SD. Student's *t*-test.
