# Supplementary figures and images for: AAV induces hepatic necroptosis and carcinoma in diabetic and obese mice dependent on Pebp1 pathway (part 1 of 2)
Source: EMBO Mol Med. 2023 Jun 5;15(7):e17230. doi: 10.15252/emmm.202217230 (PMC10331584; doi:10.15252/emmm.202217230)

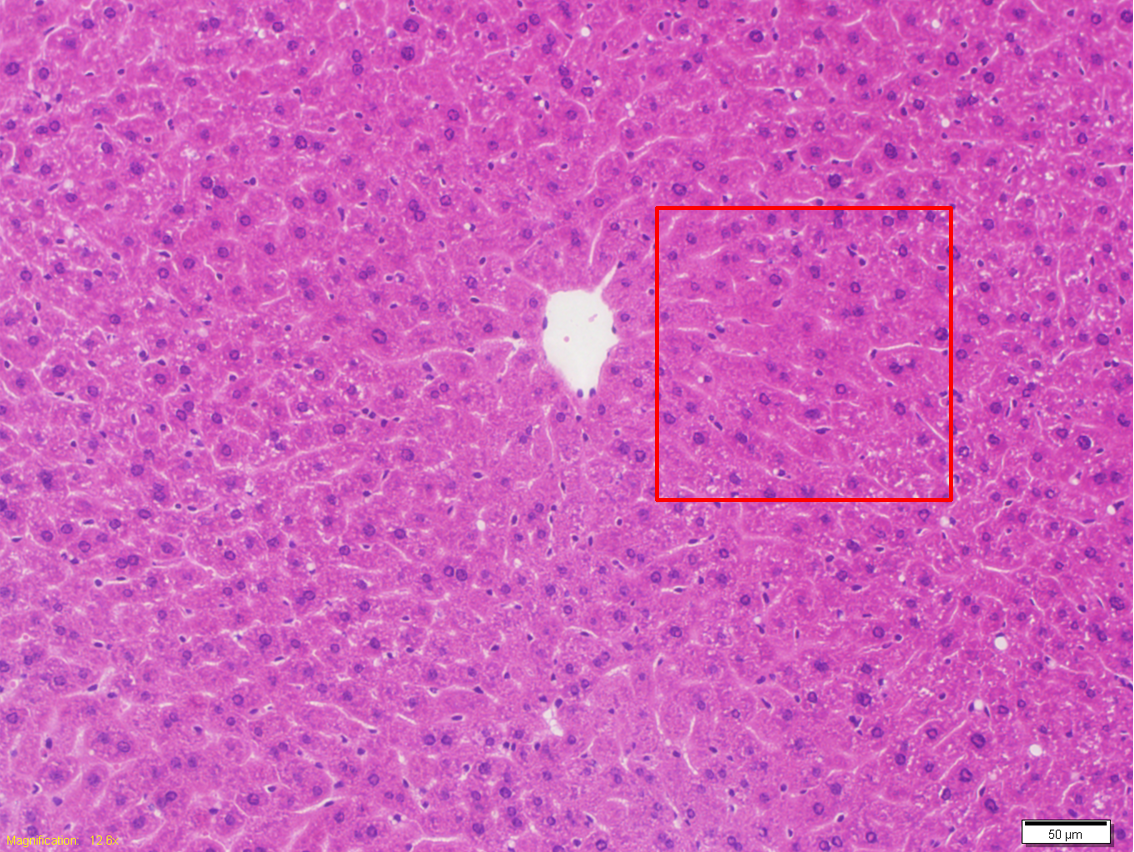

Supplement: Supplementary file 3 — Source Data for Figure 1 [file EMMM-15-e17230-s007.zip › Source Data for Figure 1/Figure 1/1G/H&E for WT-PBS.tif]

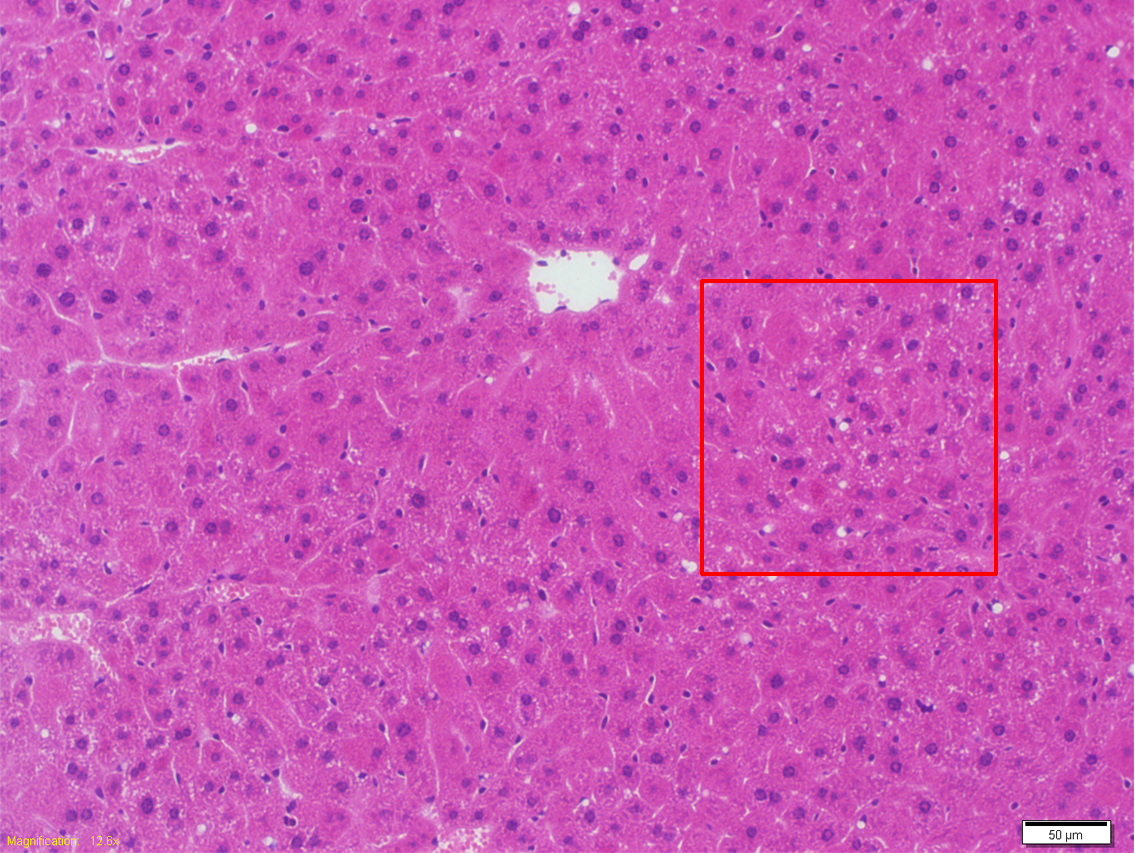

Supplement: Supplementary file 3 — Source Data for Figure 1 [file EMMM-15-e17230-s007.zip › Source Data for Figure 1/Figure 1/1G/H&E for WT-rAAV.tif]

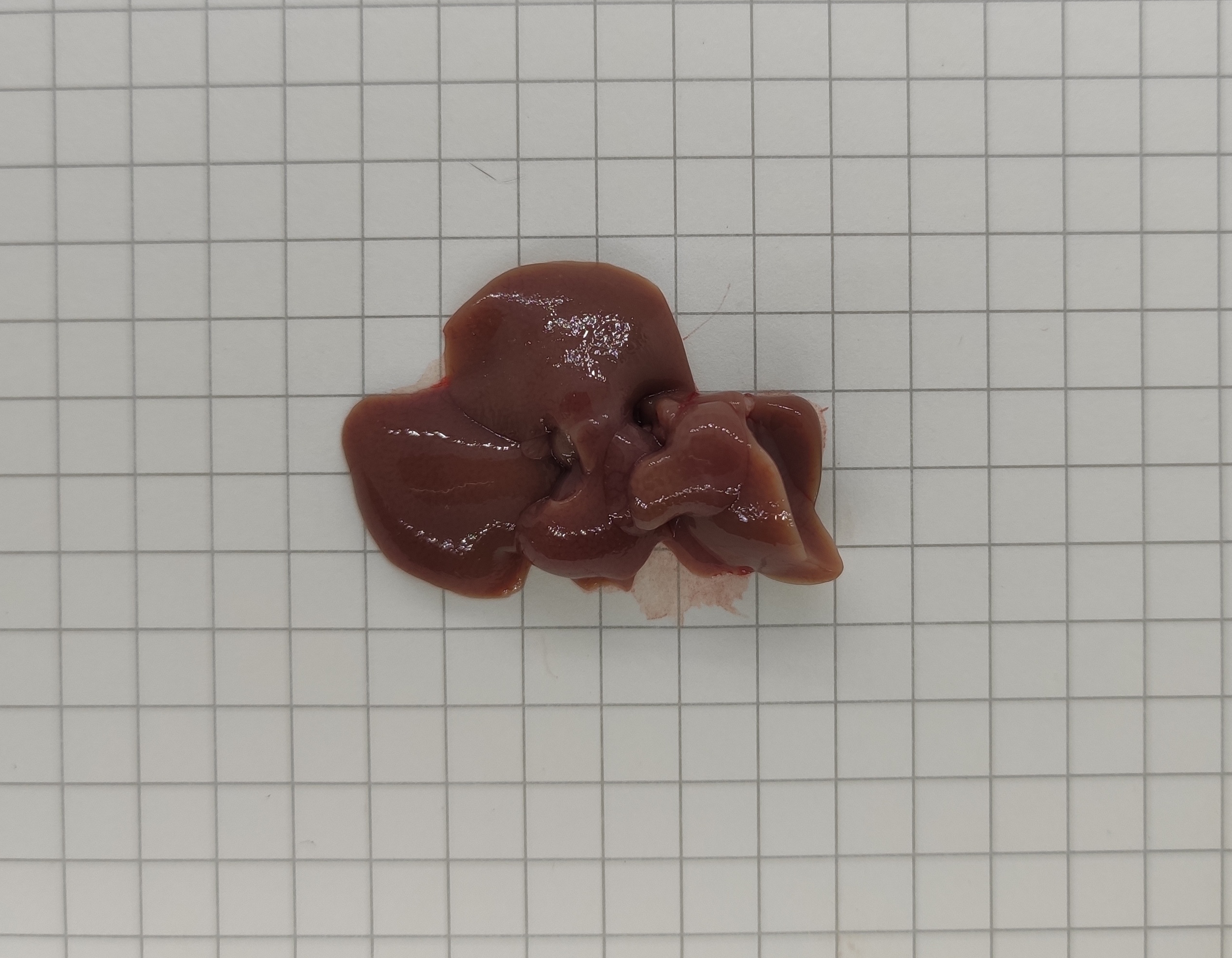

Supplement: Supplementary file 3 — Source Data for Figure 1 [file EMMM-15-e17230-s007.zip › Source Data for Figure 1/Figure 1/1G/WT-PBS-Liver.tif]

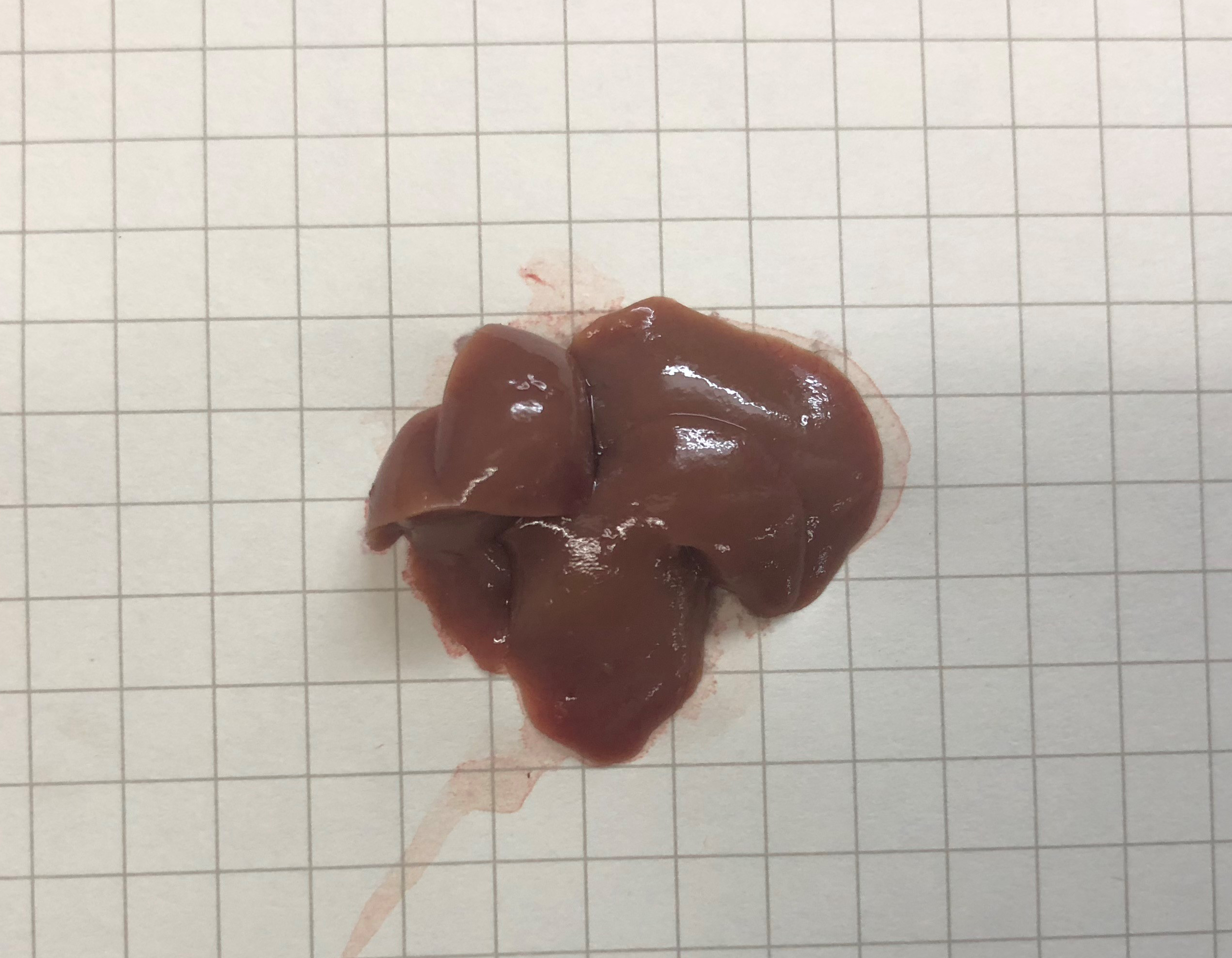

Supplement: Supplementary file 3 — Source Data for Figure 1 [file EMMM-15-e17230-s007.zip › Source Data for Figure 1/Figure 1/1G/WT-rAAV-Liver.tif]

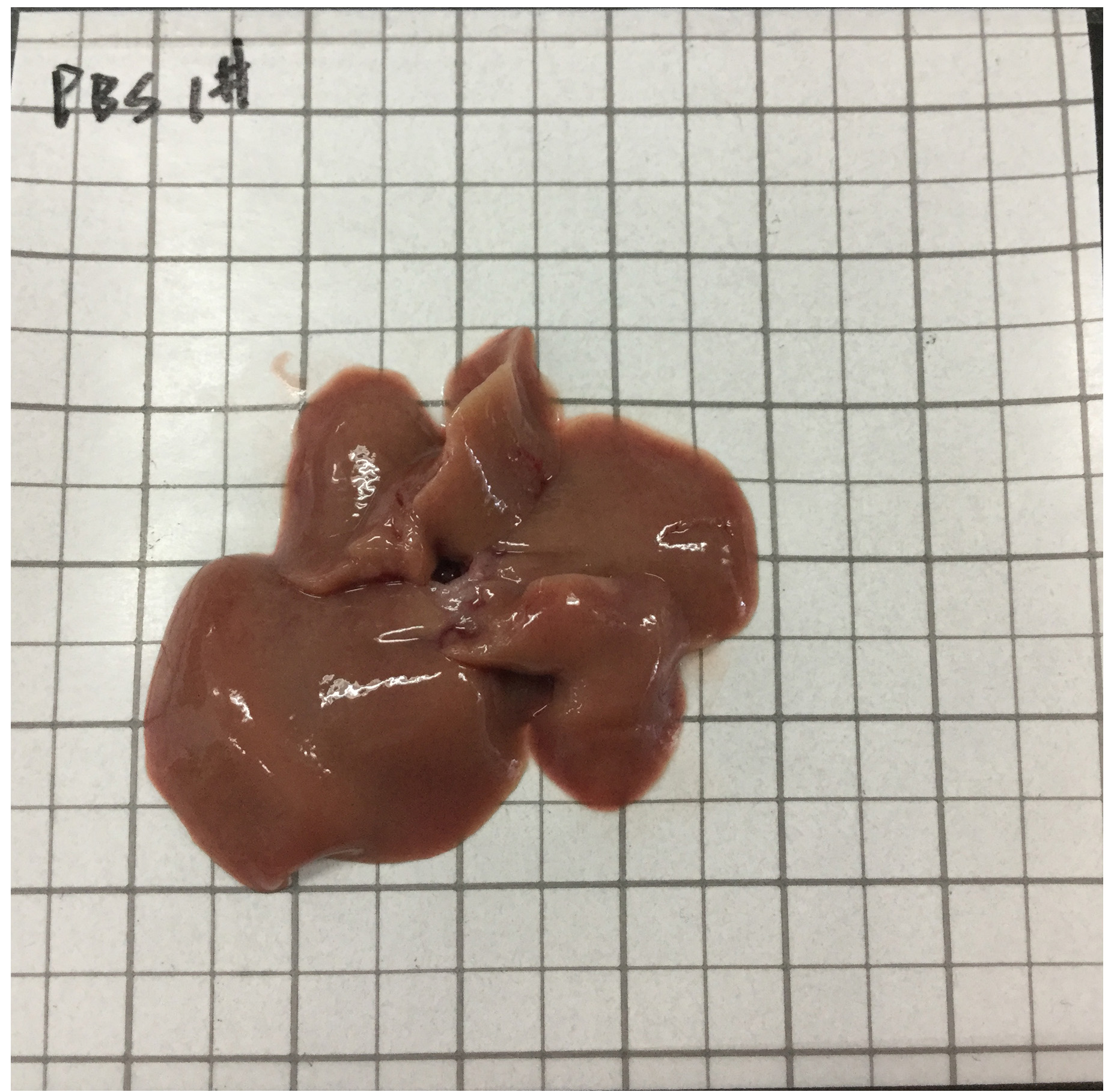

Supplement: Supplementary file 3 — Source Data for Figure 1 [file EMMM-15-e17230-s007.zip › Source Data for Figure 1/Figure 1/1L/DB-PBS-Liver.tif]

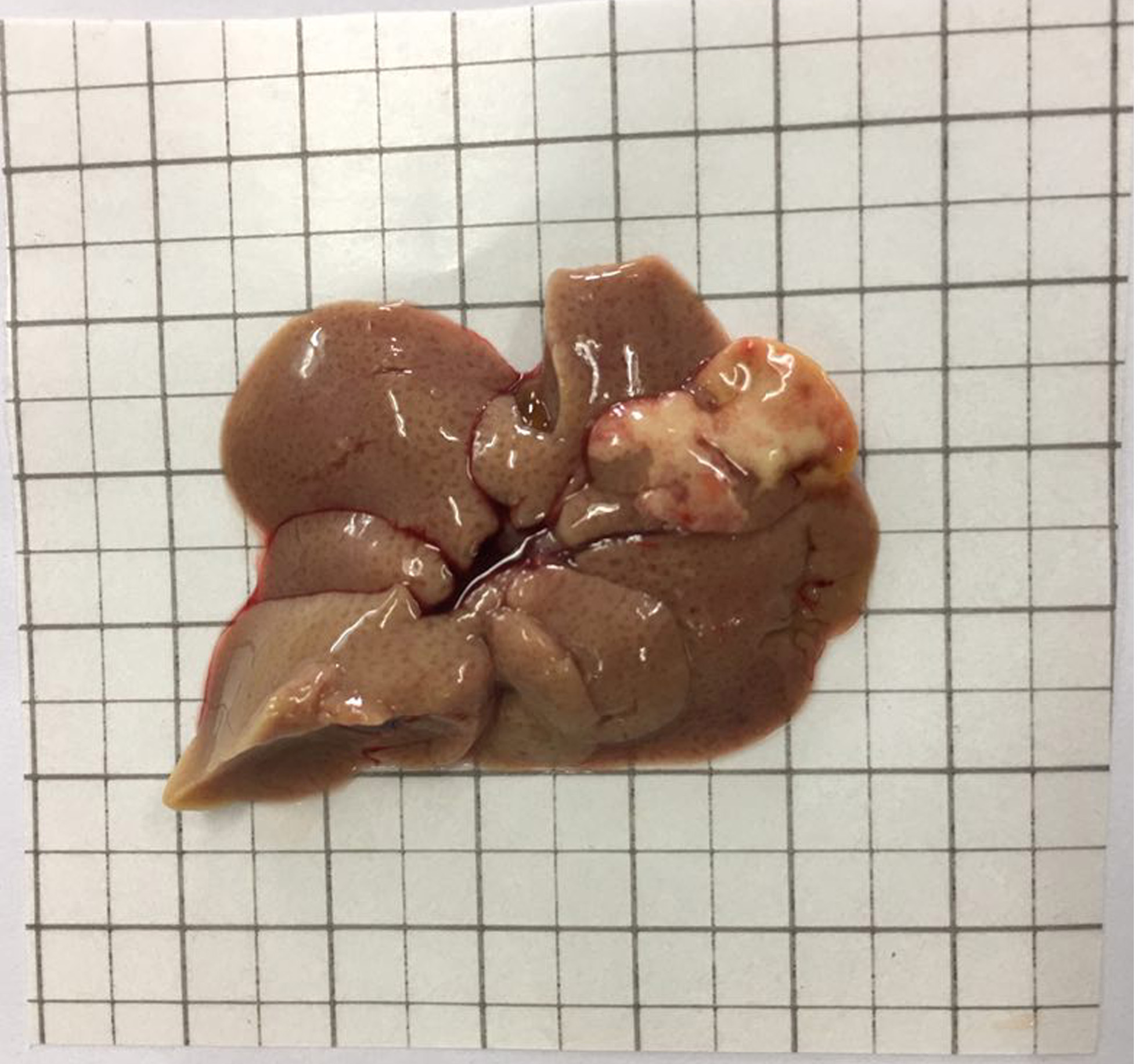

Supplement: Supplementary file 3 — Source Data for Figure 1 [file EMMM-15-e17230-s007.zip › Source Data for Figure 1/Figure 1/1L/DB-rAAV-Liver.tif]

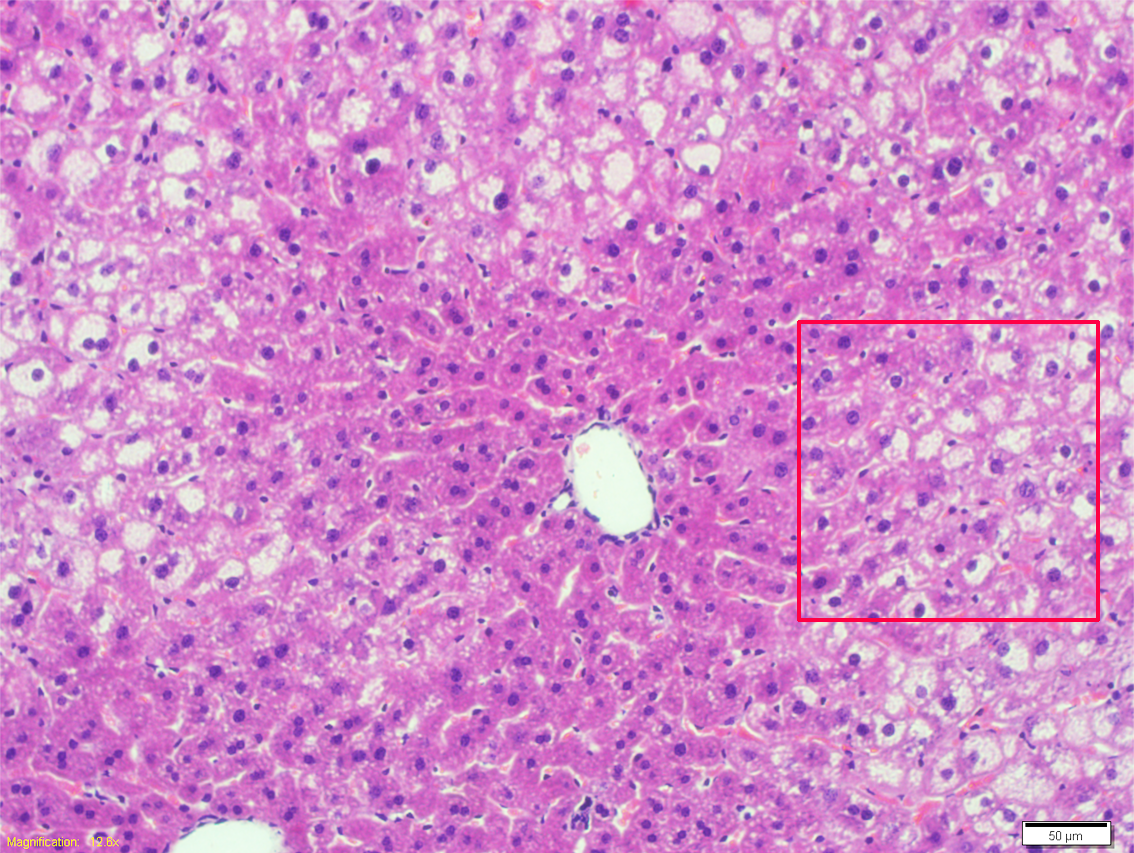

Supplement: Supplementary file 3 — Source Data for Figure 1 [file EMMM-15-e17230-s007.zip › Source Data for Figure 1/Figure 1/1L/H&E for DB-PBS liver.tif]

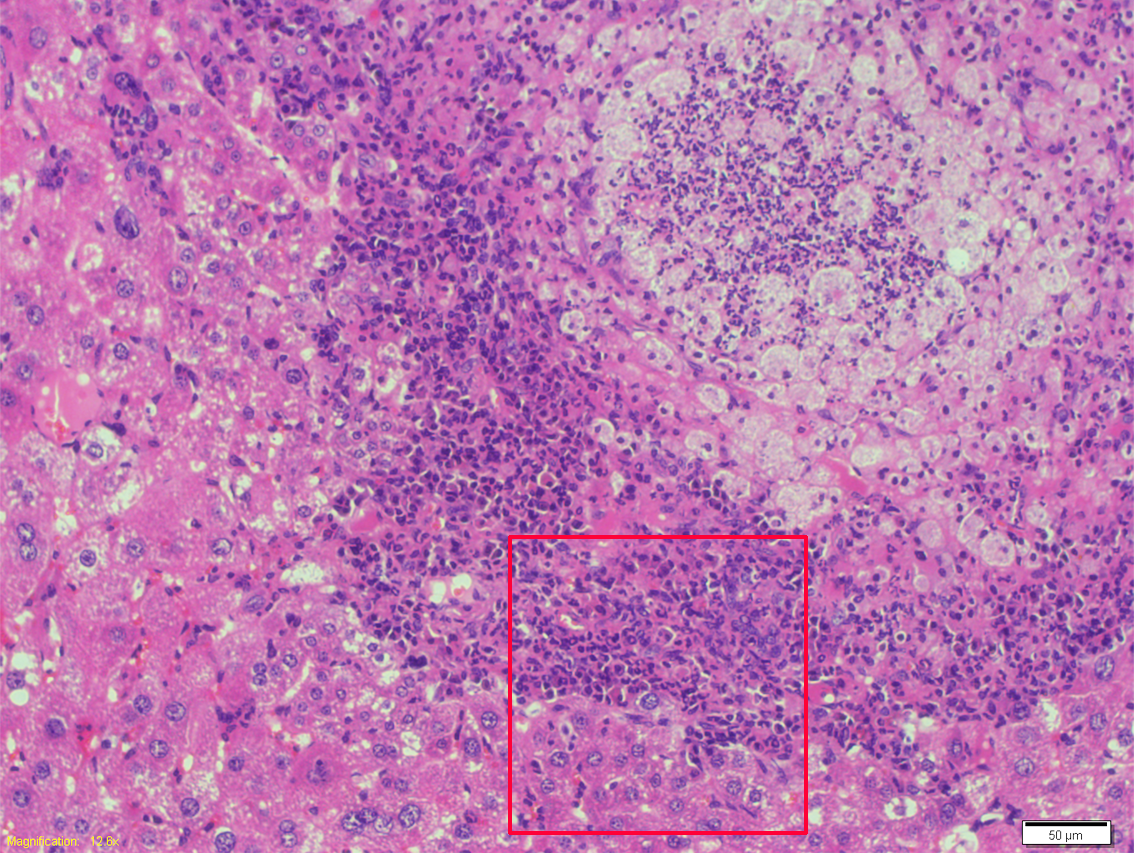

Supplement: Supplementary file 3 — Source Data for Figure 1 [file EMMM-15-e17230-s007.zip › Source Data for Figure 1/Figure 1/1L/H&E for DB-rAAV liver.tif]

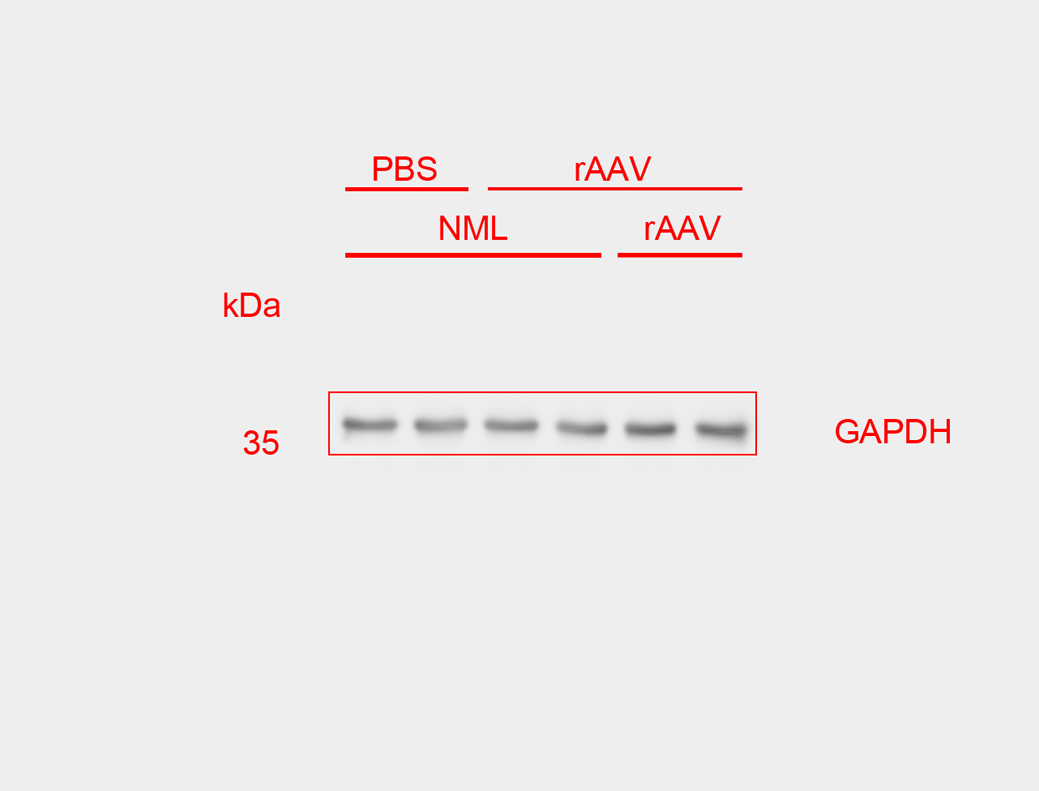

Supplement: Supplementary file 3 — Source Data for Figure 1 [file EMMM-15-e17230-s007.zip › Source Data for Figure 1/Figure 1/1M/Western/GAPDH.tif]

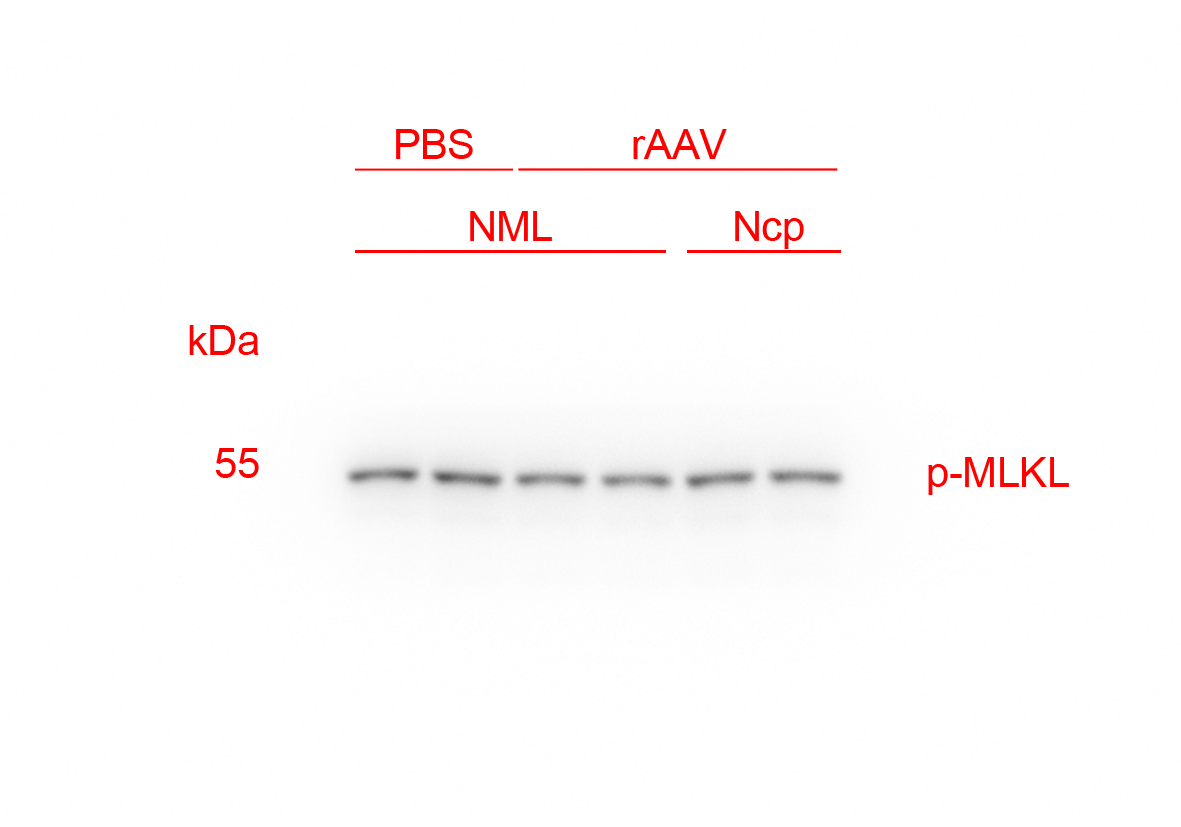

Supplement: Supplementary file 3 — Source Data for Figure 1 [file EMMM-15-e17230-s007.zip › Source Data for Figure 1/Figure 1/1M/Western/MLKL.tif]

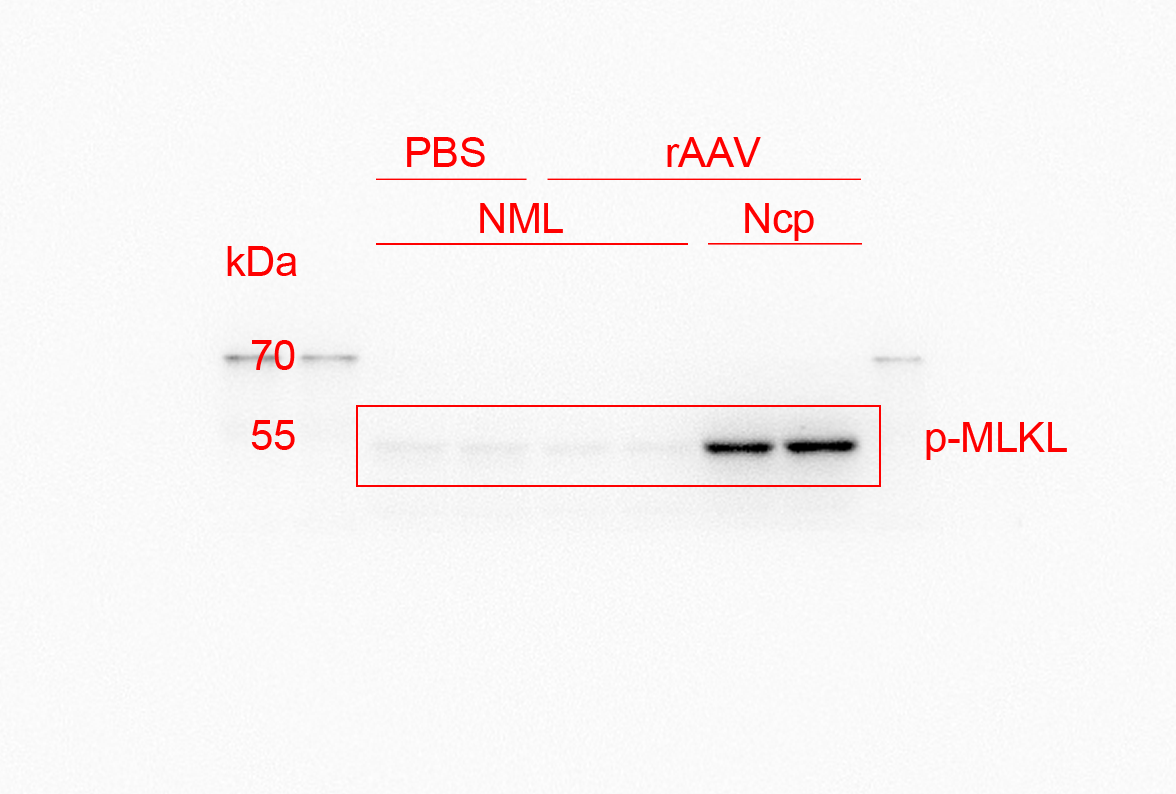

Supplement: Supplementary file 3 — Source Data for Figure 1 [file EMMM-15-e17230-s007.zip › Source Data for Figure 1/Figure 1/1M/Western/p-MLKL.tif]

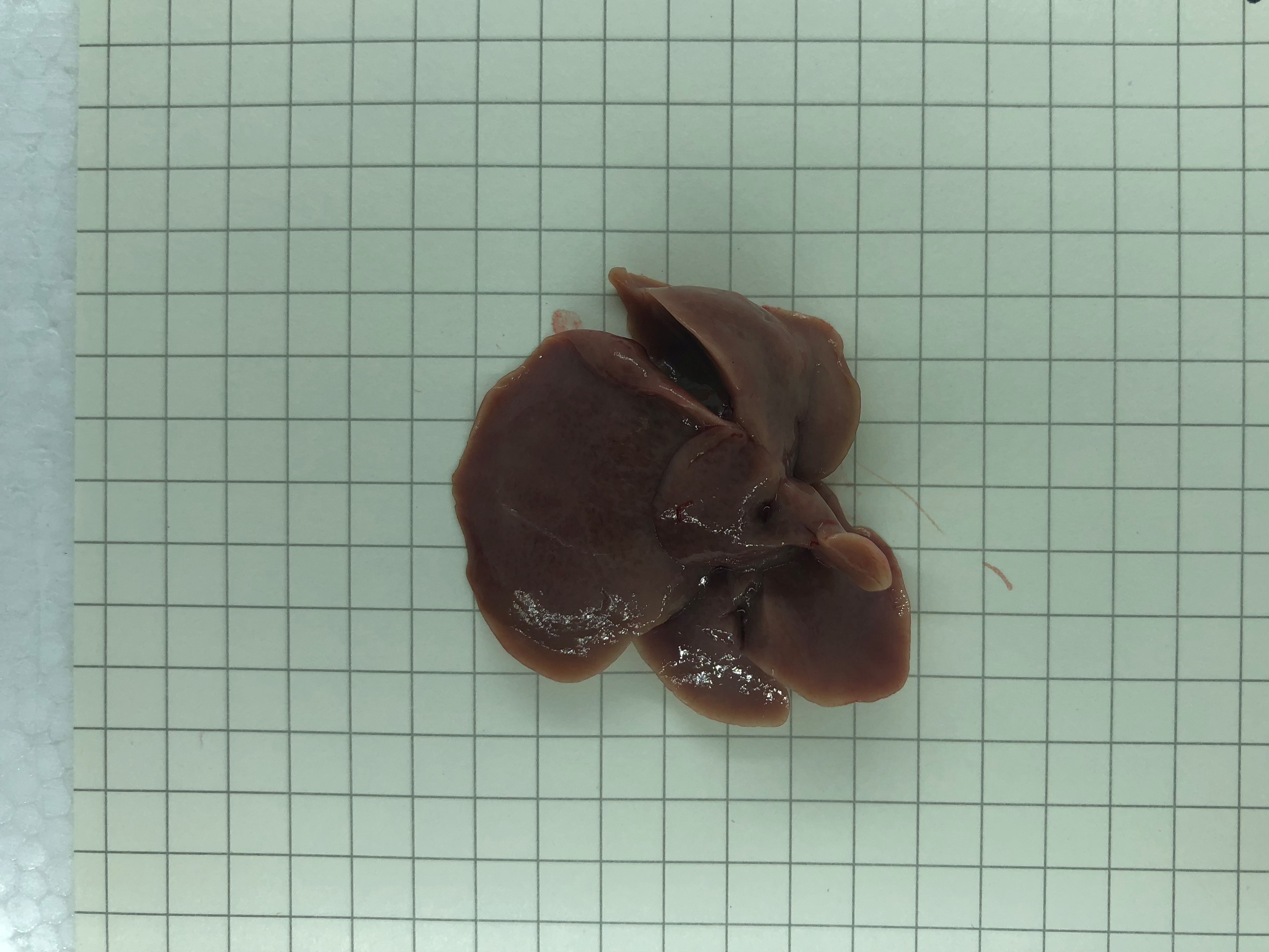

Supplement: Supplementary file 3 — Source Data for Figure 1 [file EMMM-15-e17230-s007.zip › Source Data for Figure 1/Figure 1/1U/DB-PBS-6M.tif]

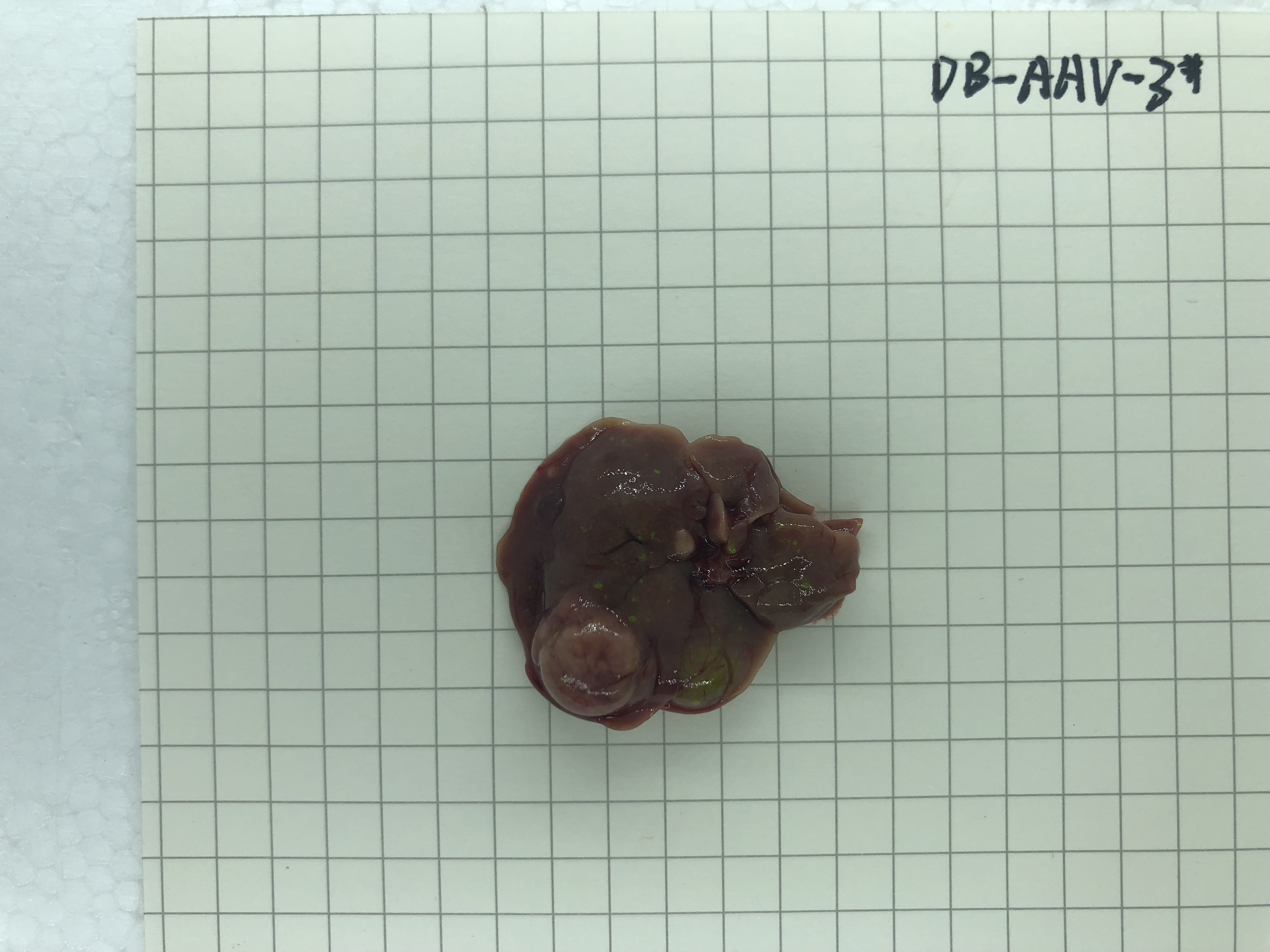

Supplement: Supplementary file 3 — Source Data for Figure 1 [file EMMM-15-e17230-s007.zip › Source Data for Figure 1/Figure 1/1U/DB-rAAV-6M.tif]

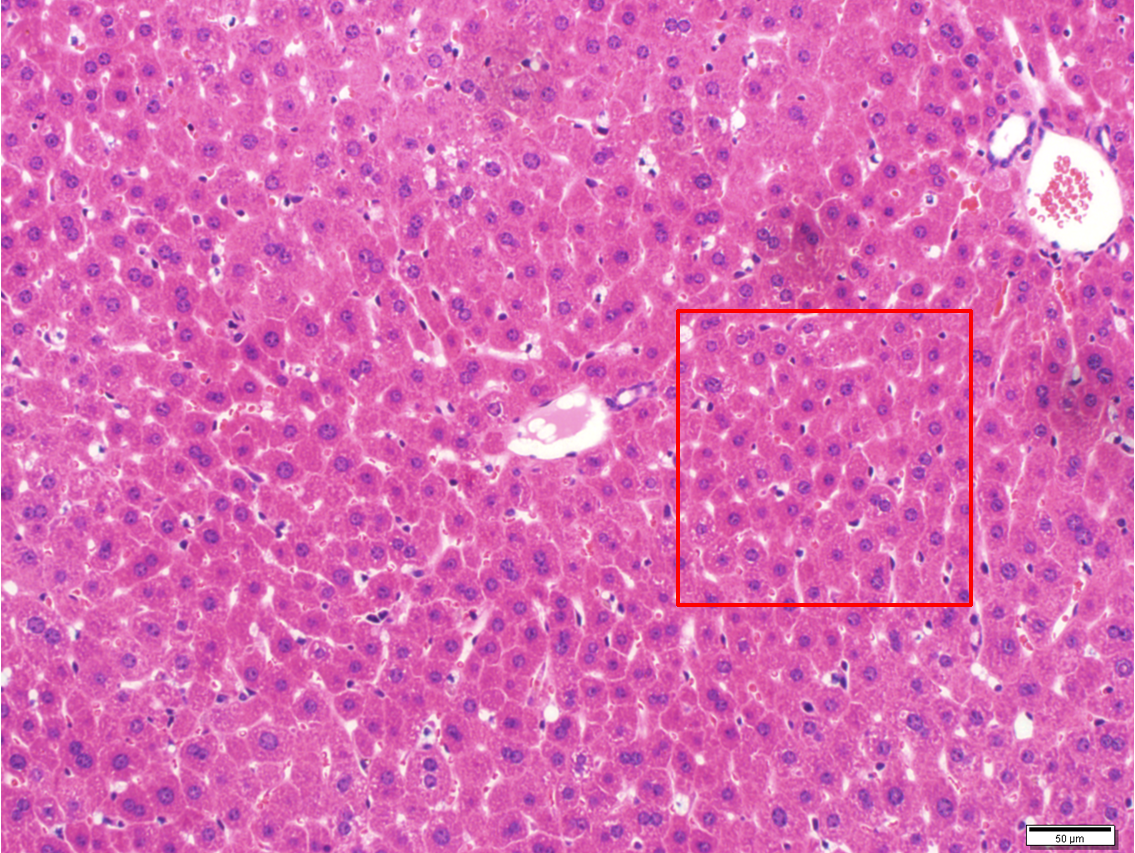

Supplement: Supplementary file 3 — Source Data for Figure 1 [file EMMM-15-e17230-s007.zip › Source Data for Figure 1/Figure 1/1U/H&E for DB-PBS-6M.tif]

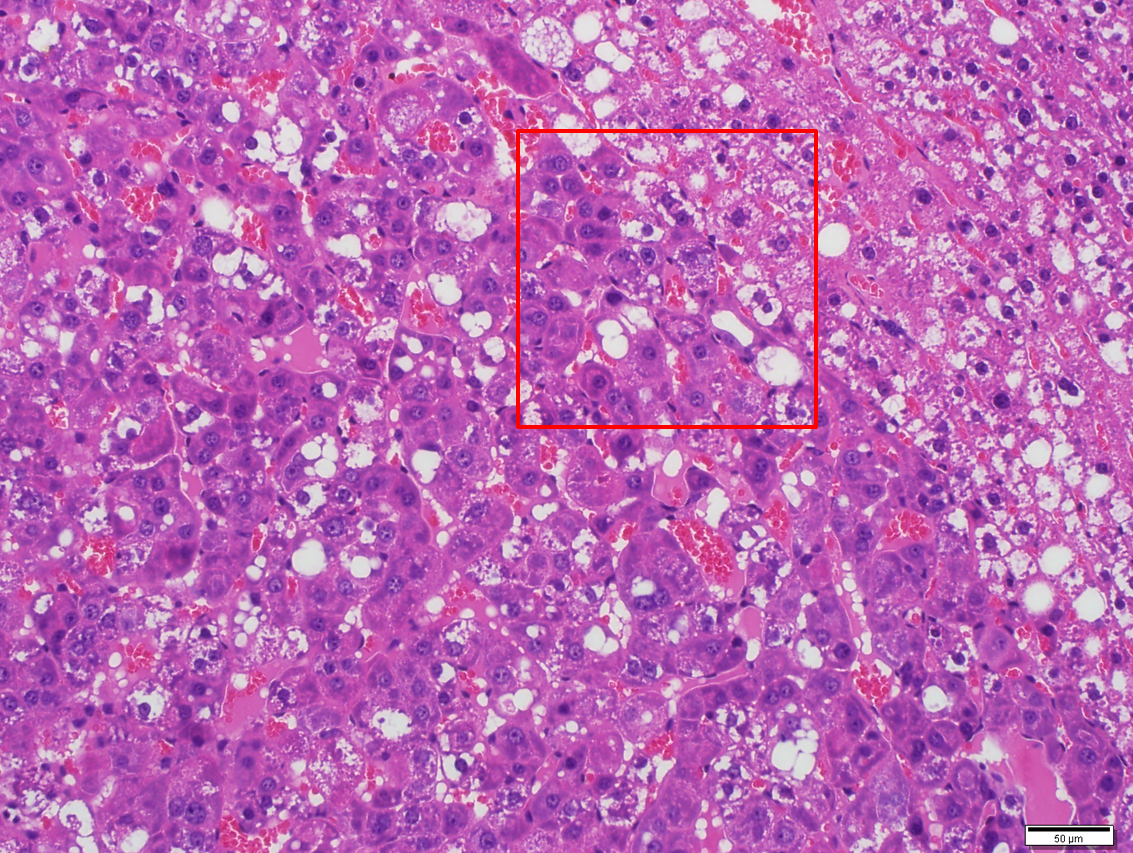

Supplement: Supplementary file 3 — Source Data for Figure 1 [file EMMM-15-e17230-s007.zip › Source Data for Figure 1/Figure 1/1U/H&E for DB-rAAV-6M.tif]

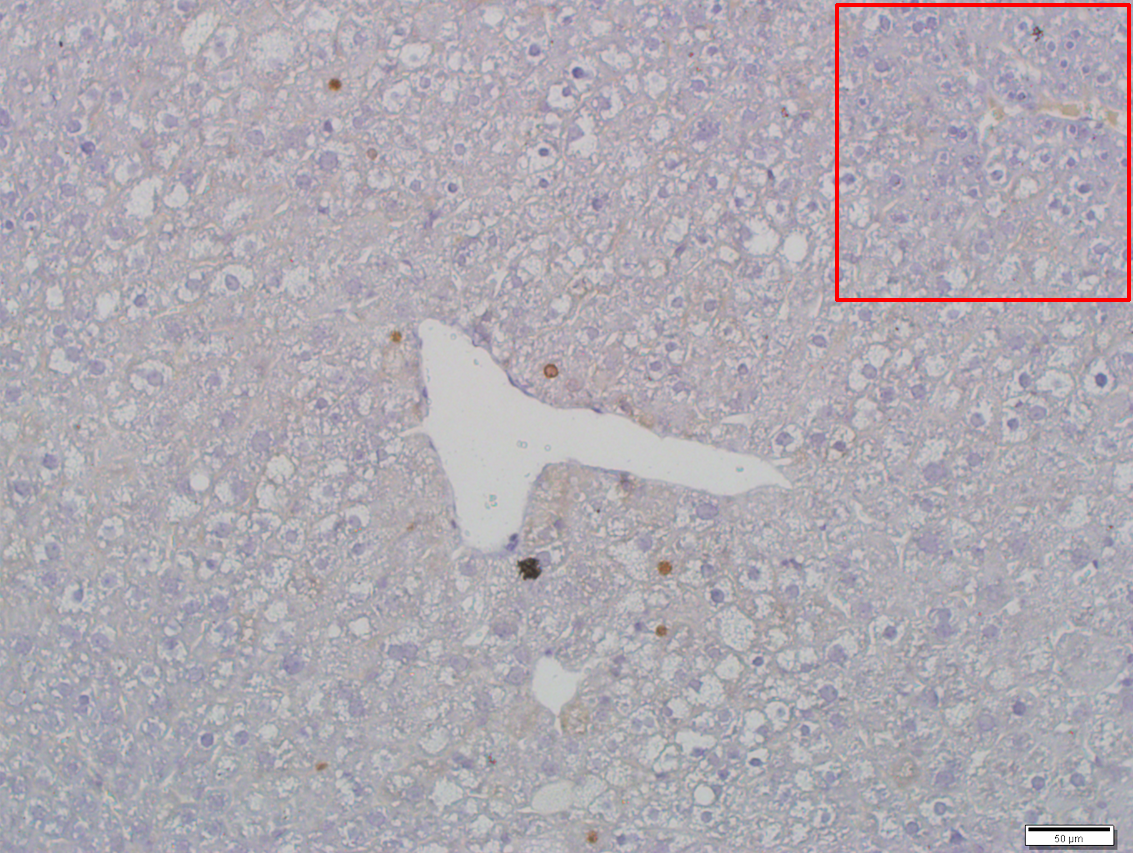

Supplement: Supplementary file 3 — Source Data for Figure 1 [file EMMM-15-e17230-s007.zip › Source Data for Figure 1/Figure 1/1U/IHC-ki67 for DB-PBS-6M.tif]

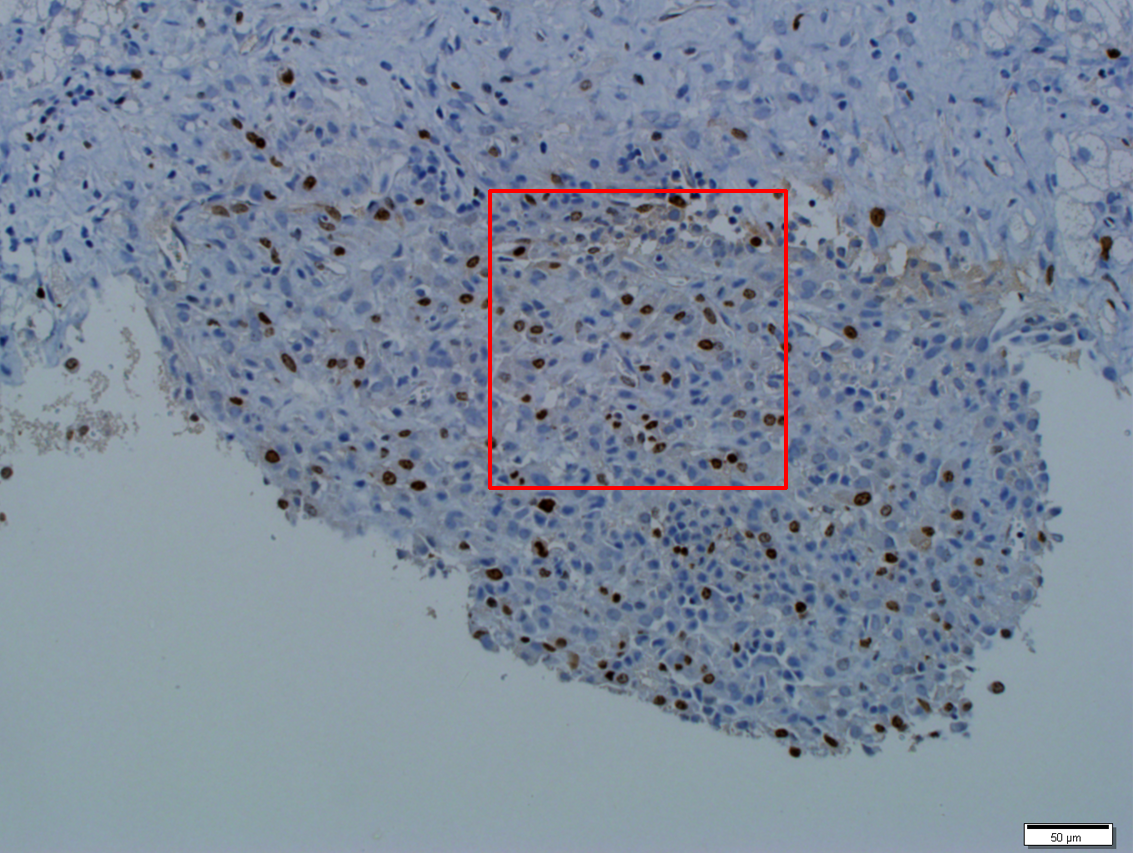

Supplement: Supplementary file 3 — Source Data for Figure 1 [file EMMM-15-e17230-s007.zip › Source Data for Figure 1/Figure 1/1U/IHC-ki67 for DB-rAAV-6M(Tumor).tif]

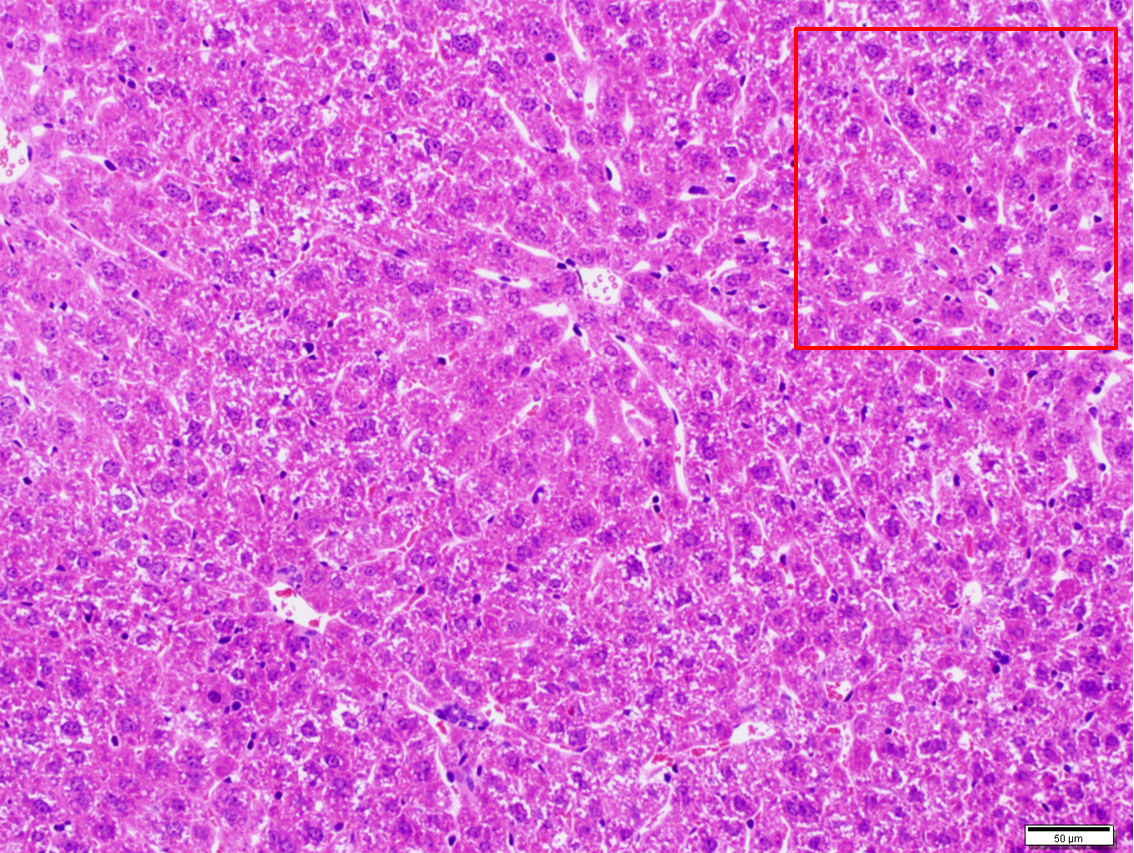

Supplement: Supplementary file 4 — Source Data for Figure 2 [file EMMM-15-e17230-s005.zip › Figure 2/2I/H&E for WT-STZ-PBS.tif]

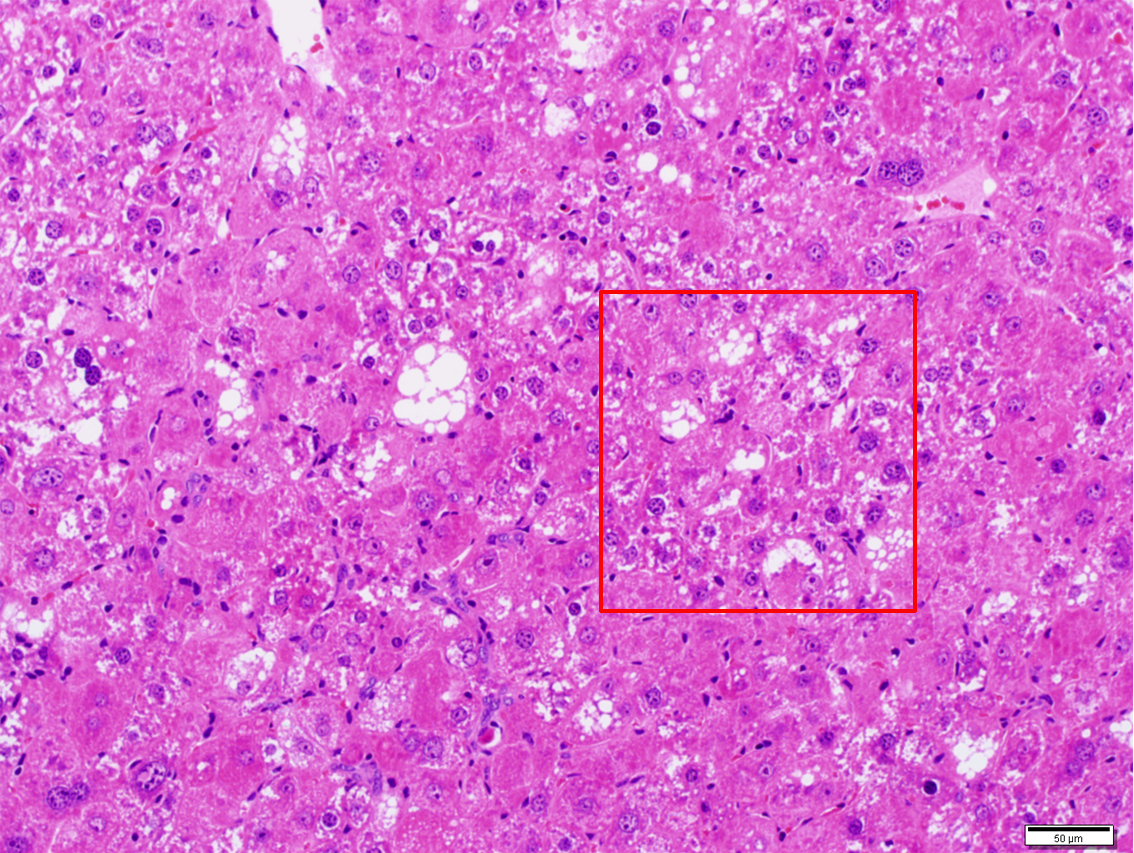

Supplement: Supplementary file 4 — Source Data for Figure 2 [file EMMM-15-e17230-s005.zip › Figure 2/2I/H&E for WT-STZ-rAAV.tif]

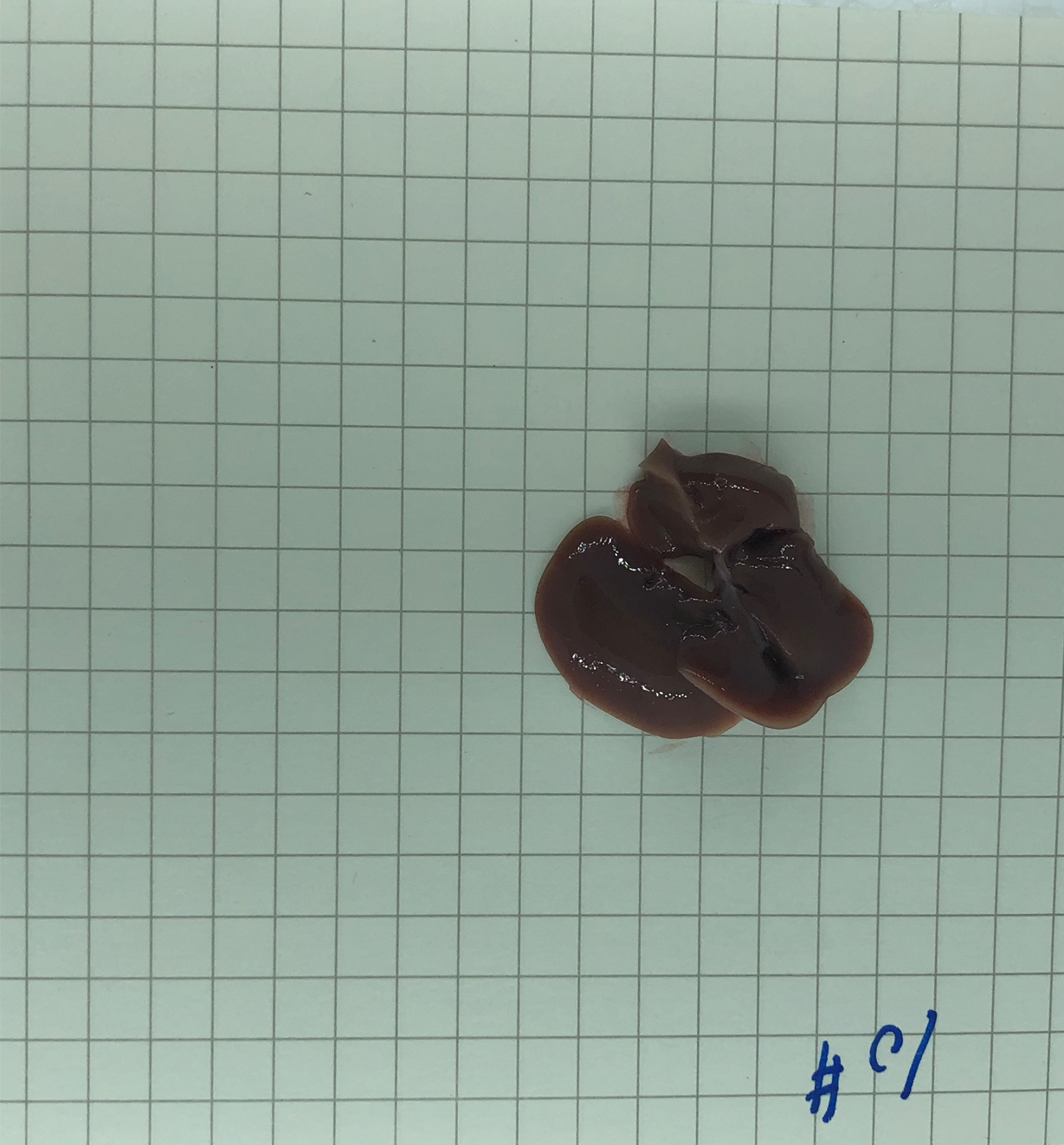

Supplement: Supplementary file 4 — Source Data for Figure 2 [file EMMM-15-e17230-s005.zip › Figure 2/2I/WT-STZ-PBS-liver.tif]

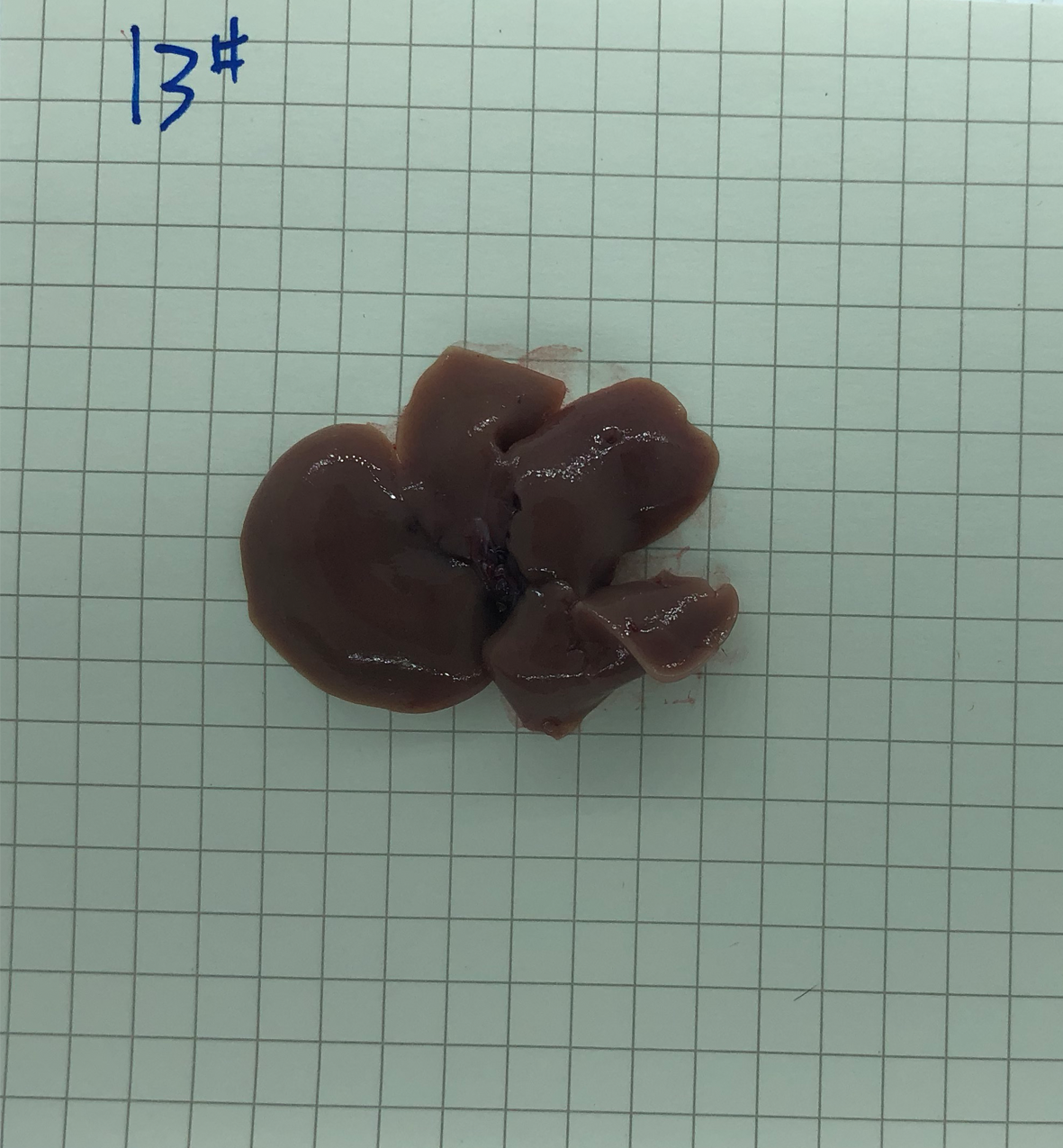

Supplement: Supplementary file 4 — Source Data for Figure 2 [file EMMM-15-e17230-s005.zip › Figure 2/2I/WT-STZ-rAAV-liver.tif]

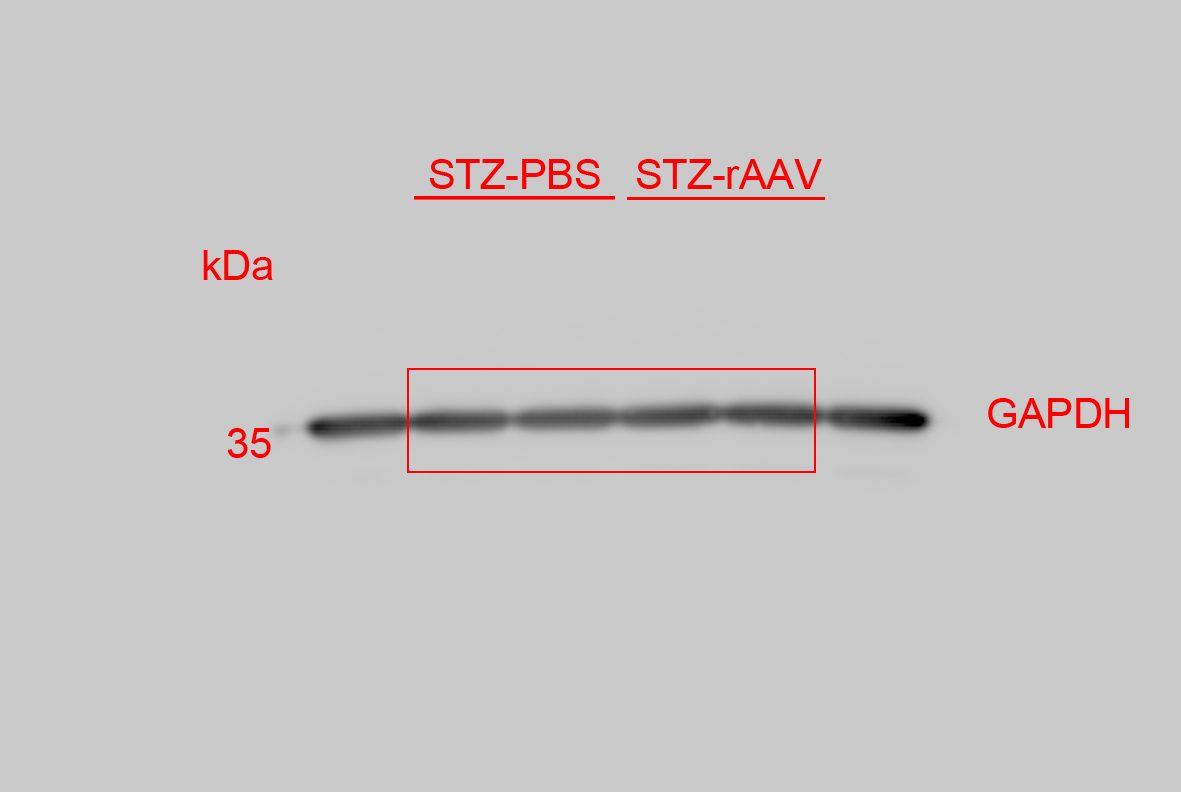

Supplement: Supplementary file 4 — Source Data for Figure 2 [file EMMM-15-e17230-s005.zip › Figure 2/2J/Western/GAPDH-(WT+STZ).tif]

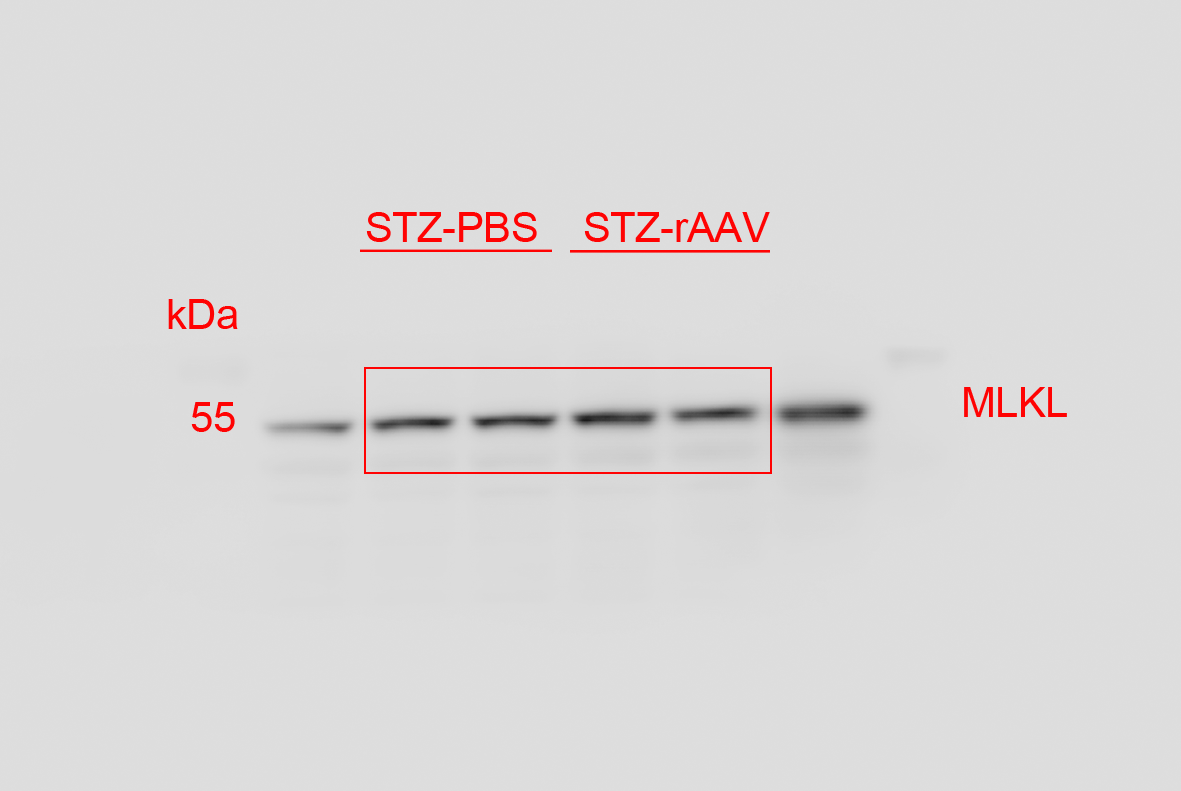

Supplement: Supplementary file 4 — Source Data for Figure 2 [file EMMM-15-e17230-s005.zip › Figure 2/2J/Western/MLKL-(WT+STZ).tif]

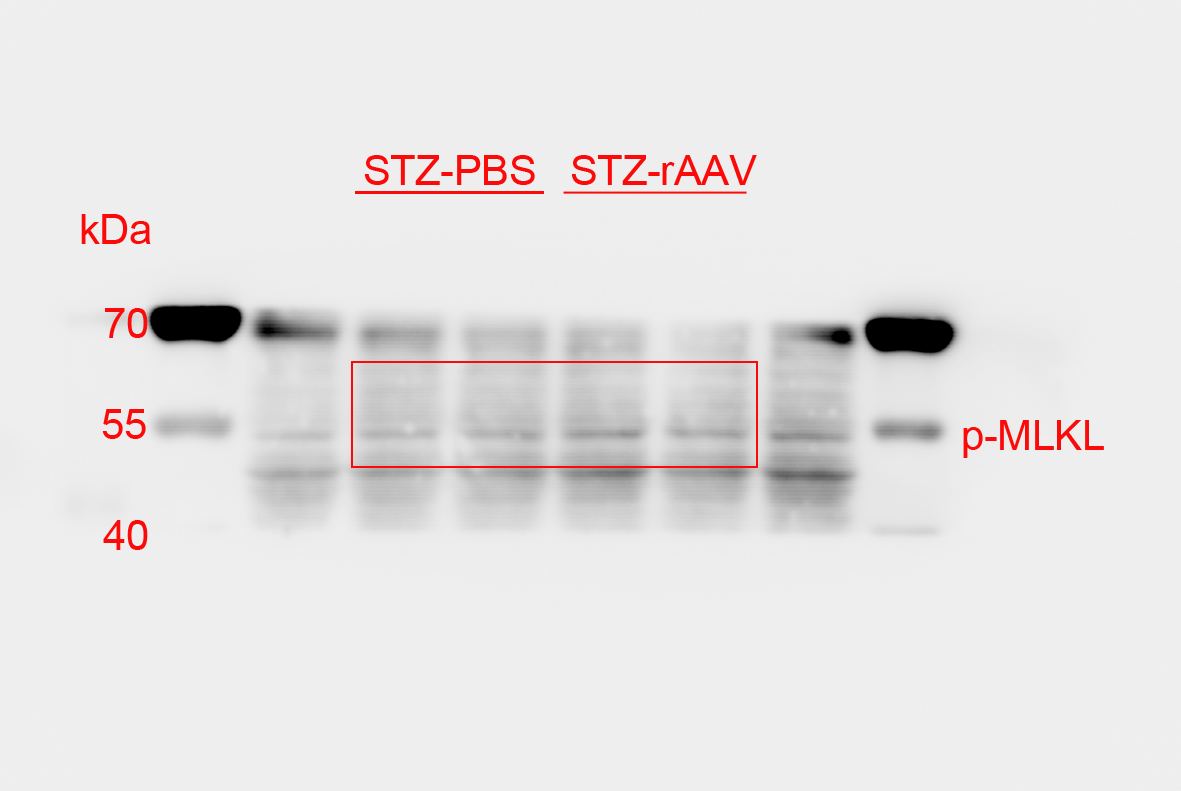

Supplement: Supplementary file 4 — Source Data for Figure 2 [file EMMM-15-e17230-s005.zip › Figure 2/2J/Western/p-MLKL-(WT+STZ).tif]

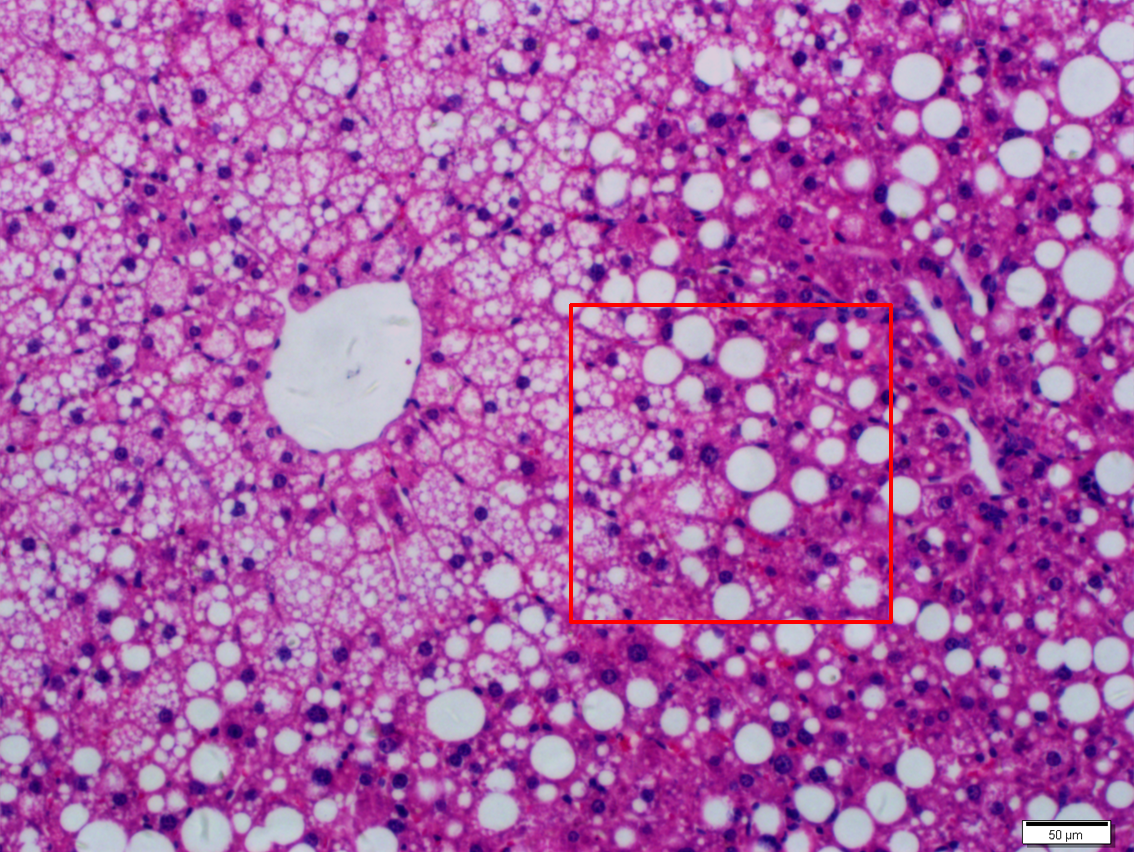

Supplement: Supplementary file 4 — Source Data for Figure 2 [file EMMM-15-e17230-s005.zip › Figure 2/2T/H&E for HFD(Obesity)-PBS.tif]

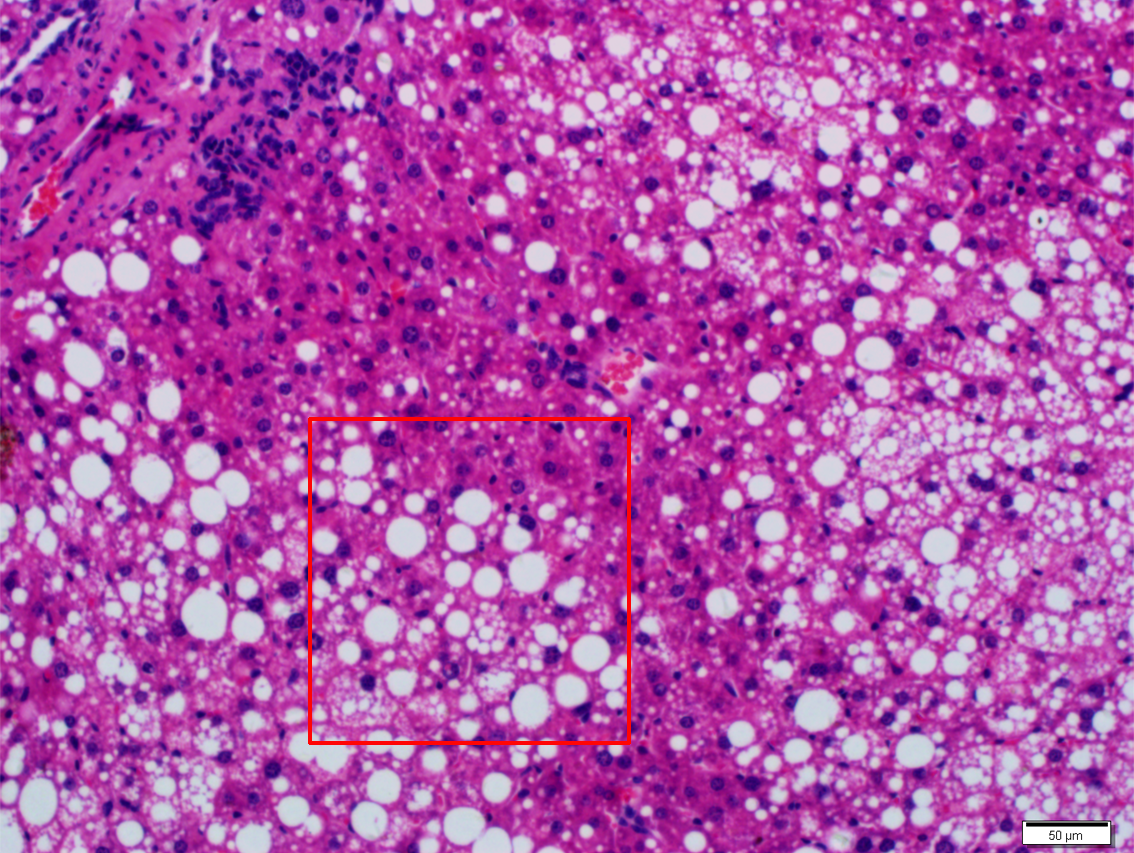

Supplement: Supplementary file 4 — Source Data for Figure 2 [file EMMM-15-e17230-s005.zip › Figure 2/2T/H&E for HFD(Obesity)-rAAV.tif]

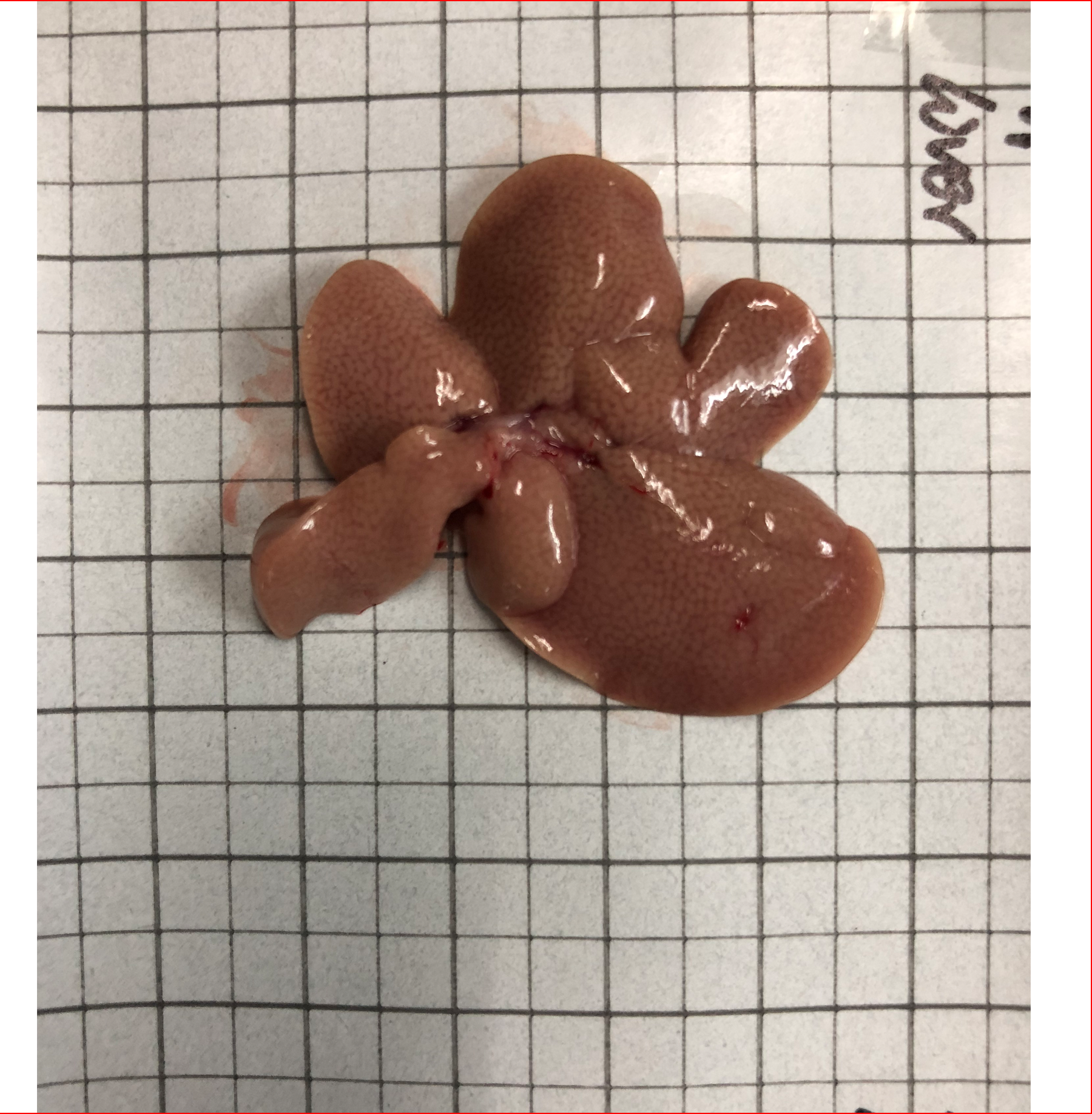

Supplement: Supplementary file 4 — Source Data for Figure 2 [file EMMM-15-e17230-s005.zip › Figure 2/2T/HFD(Obesity)-PBS-Liver.tif]

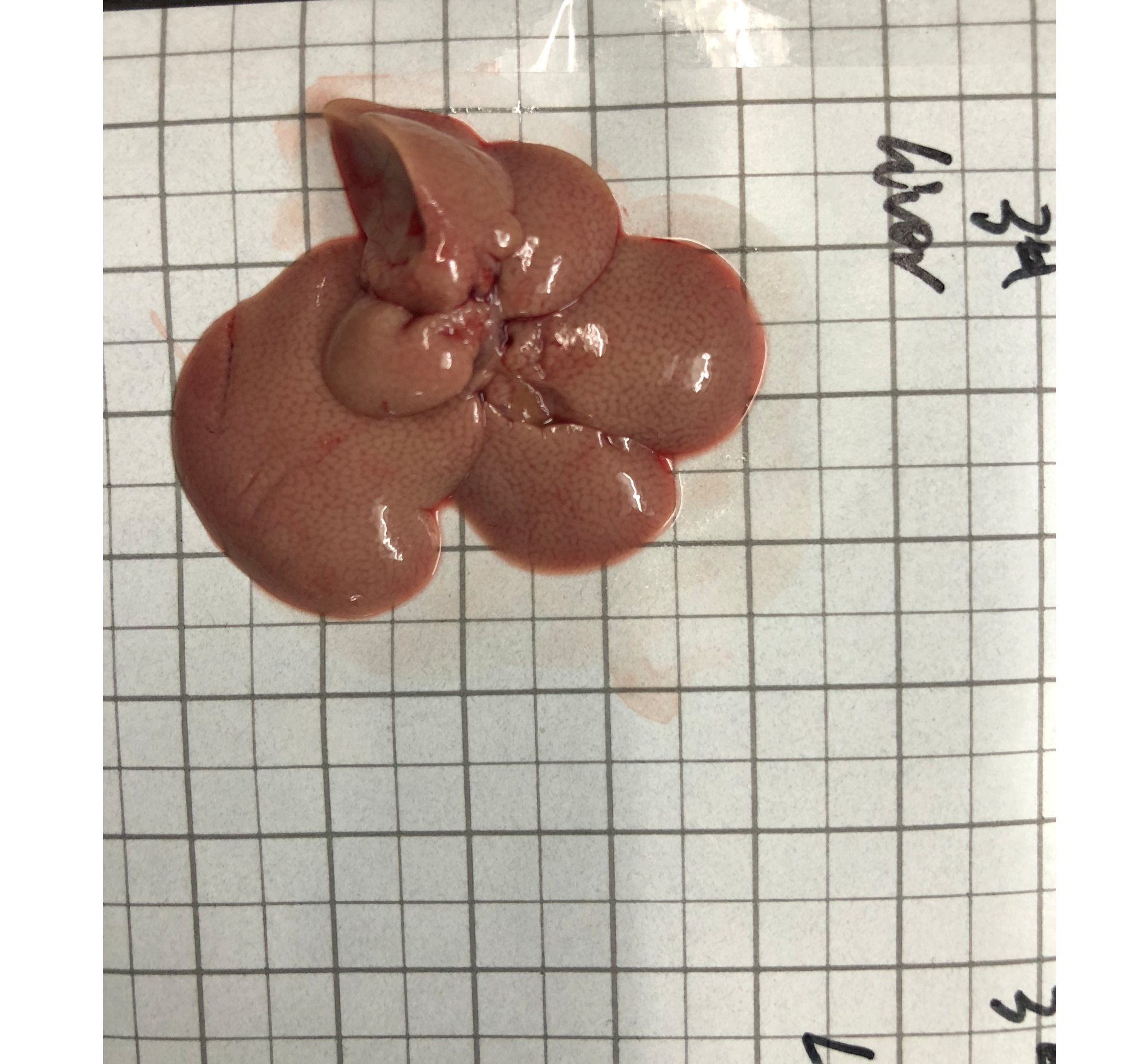

Supplement: Supplementary file 4 — Source Data for Figure 2 [file EMMM-15-e17230-s005.zip › Figure 2/2T/HFD(Obesity)-rAAV-Liver.tif]

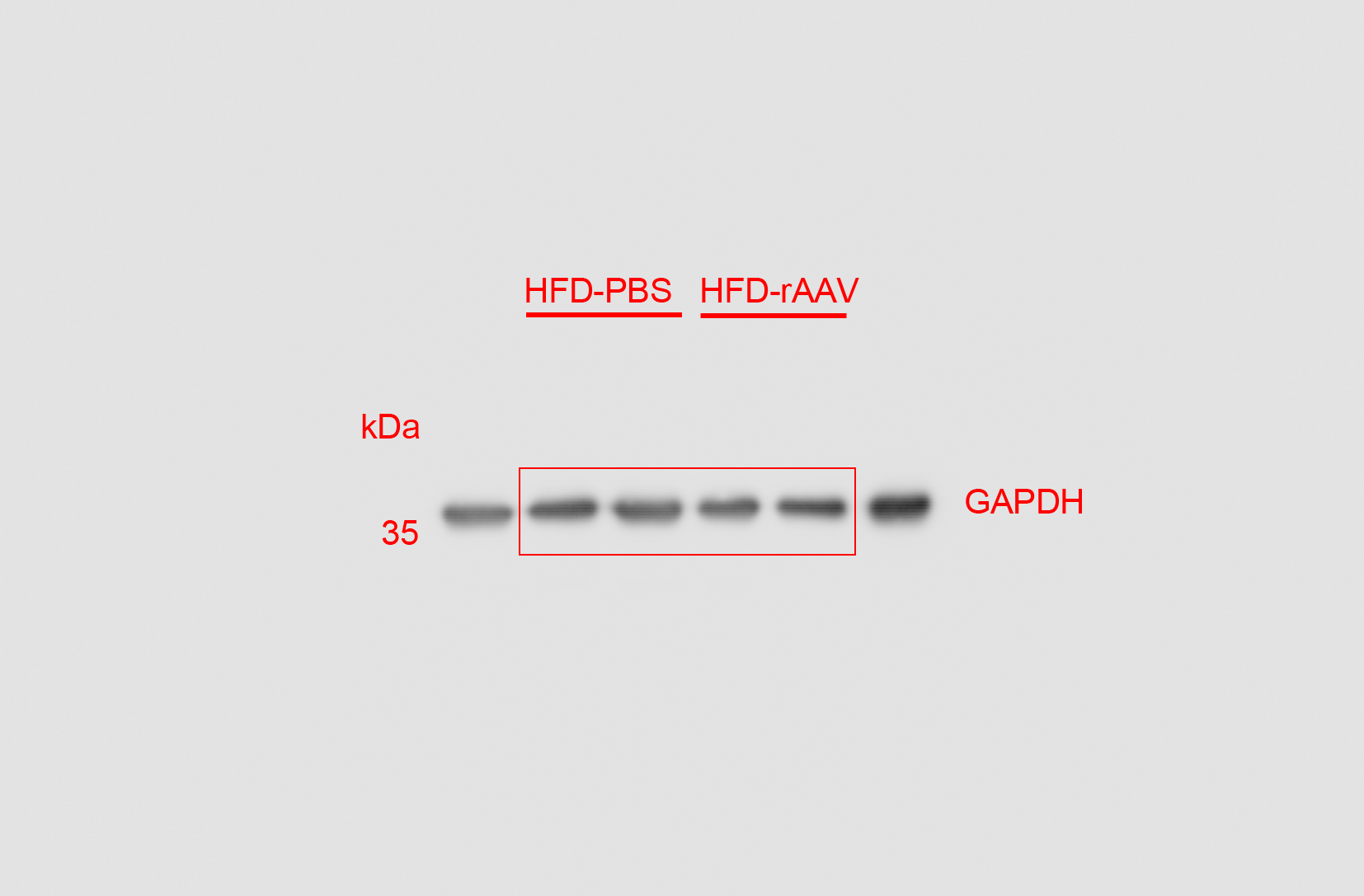

Supplement: Supplementary file 4 — Source Data for Figure 2 [file EMMM-15-e17230-s005.zip › Figure 2/2U/Western/GAPDH(HFD-Obesity).tif]

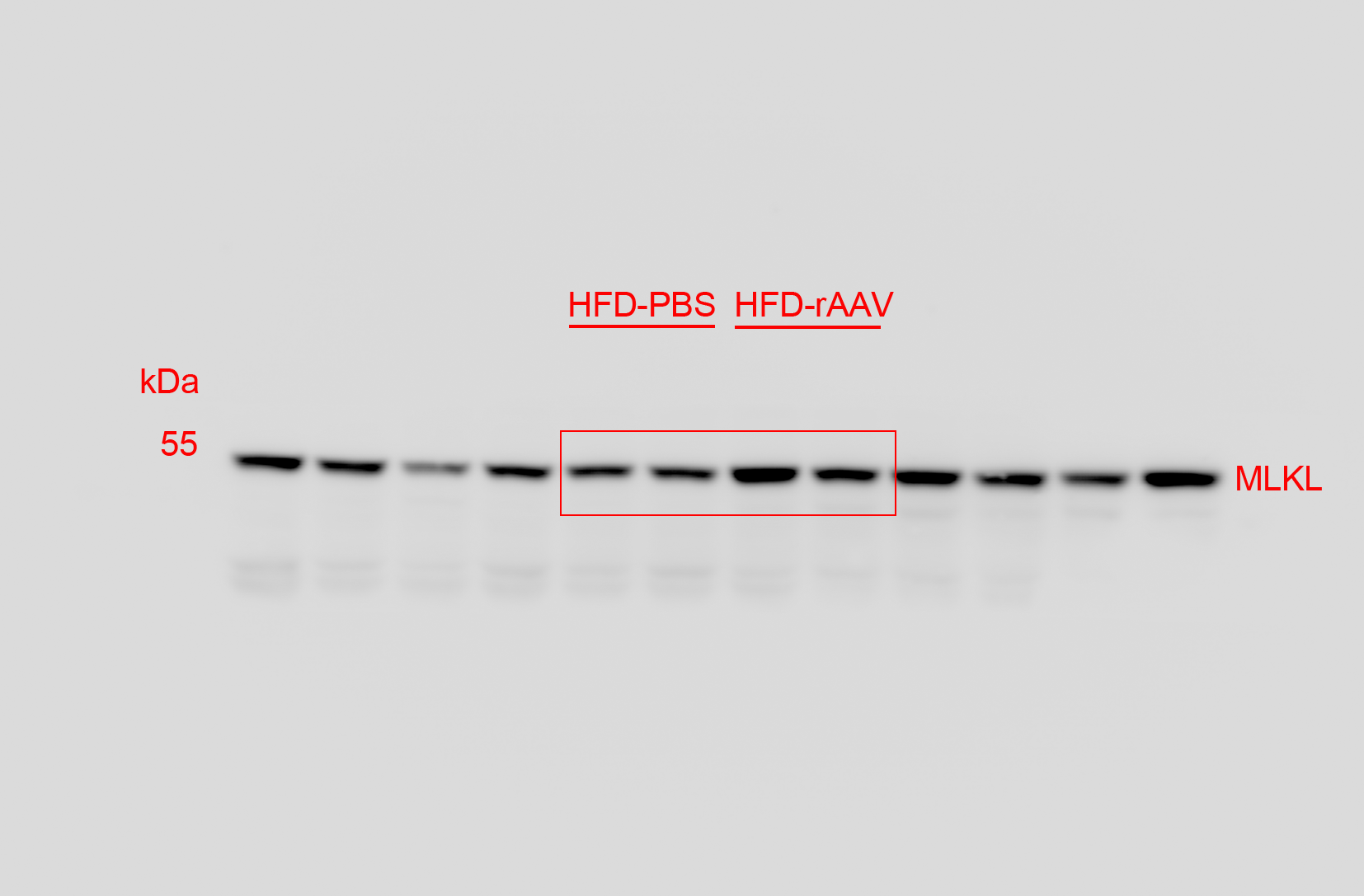

Supplement: Supplementary file 4 — Source Data for Figure 2 [file EMMM-15-e17230-s005.zip › Figure 2/2U/Western/MLKL(HFD-Obesity).tif]

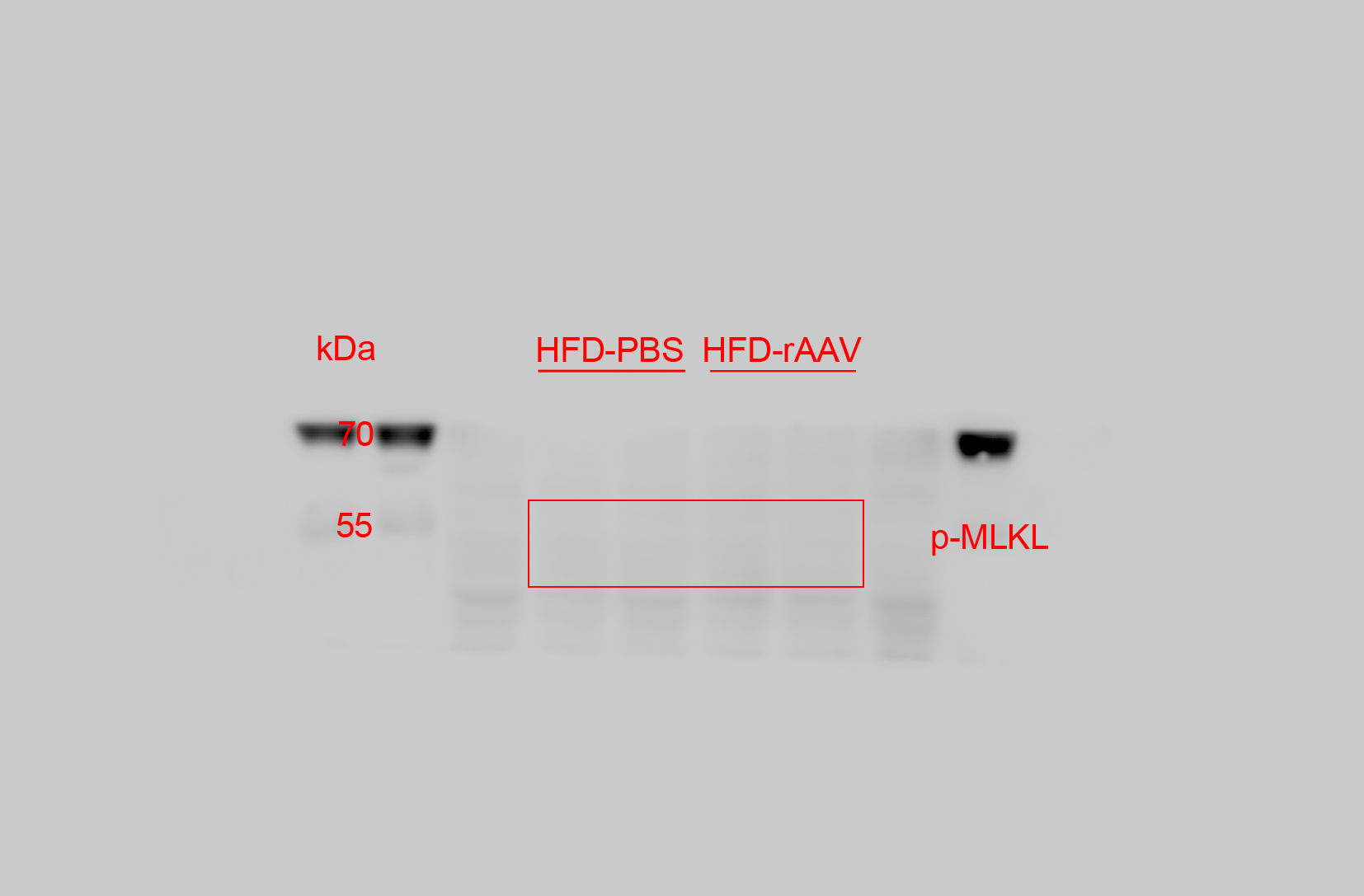

Supplement: Supplementary file 4 — Source Data for Figure 2 [file EMMM-15-e17230-s005.zip › Figure 2/2U/Western/p-MLKL(HFD-Obesity).tif]

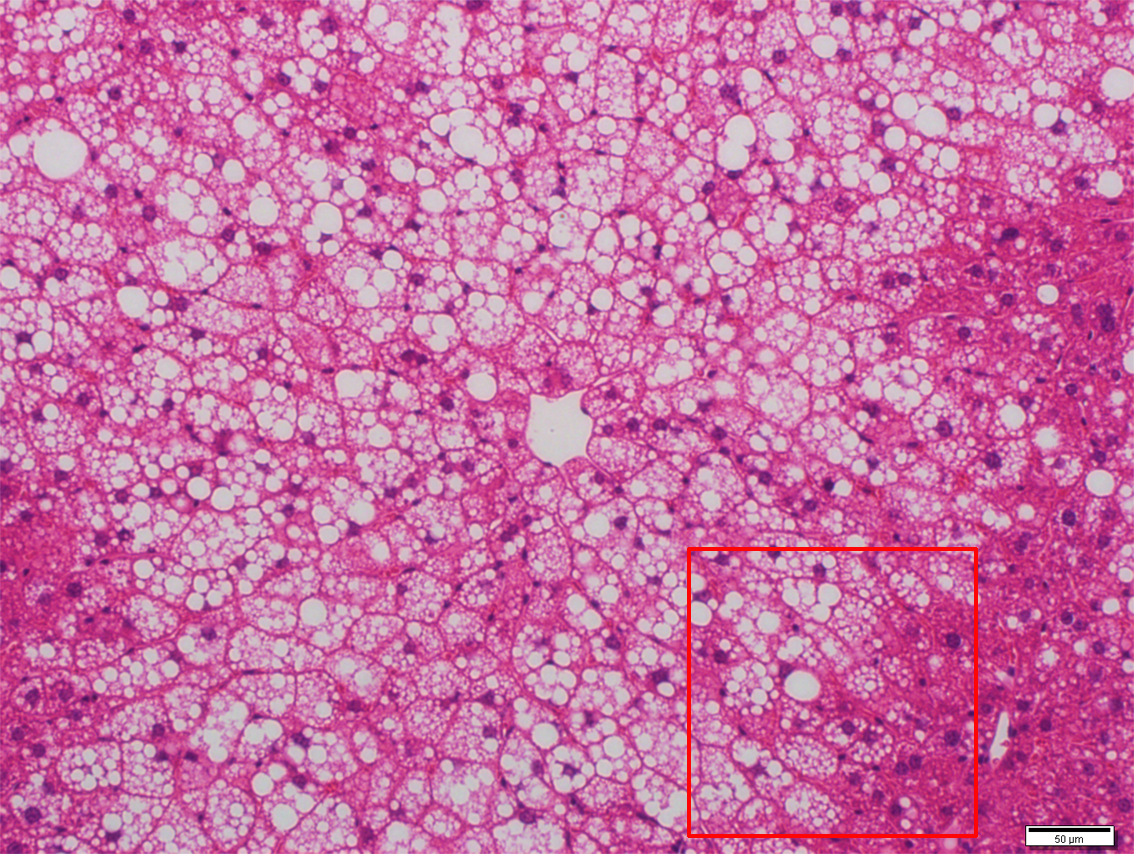

Supplement: Supplementary file 5 — Source Data for Figure 3 [file EMMM-15-e17230-s004.zip › Figure 3/3J/H&E for HFD(Diabetes&Obesity)-PBS-2M.tif]

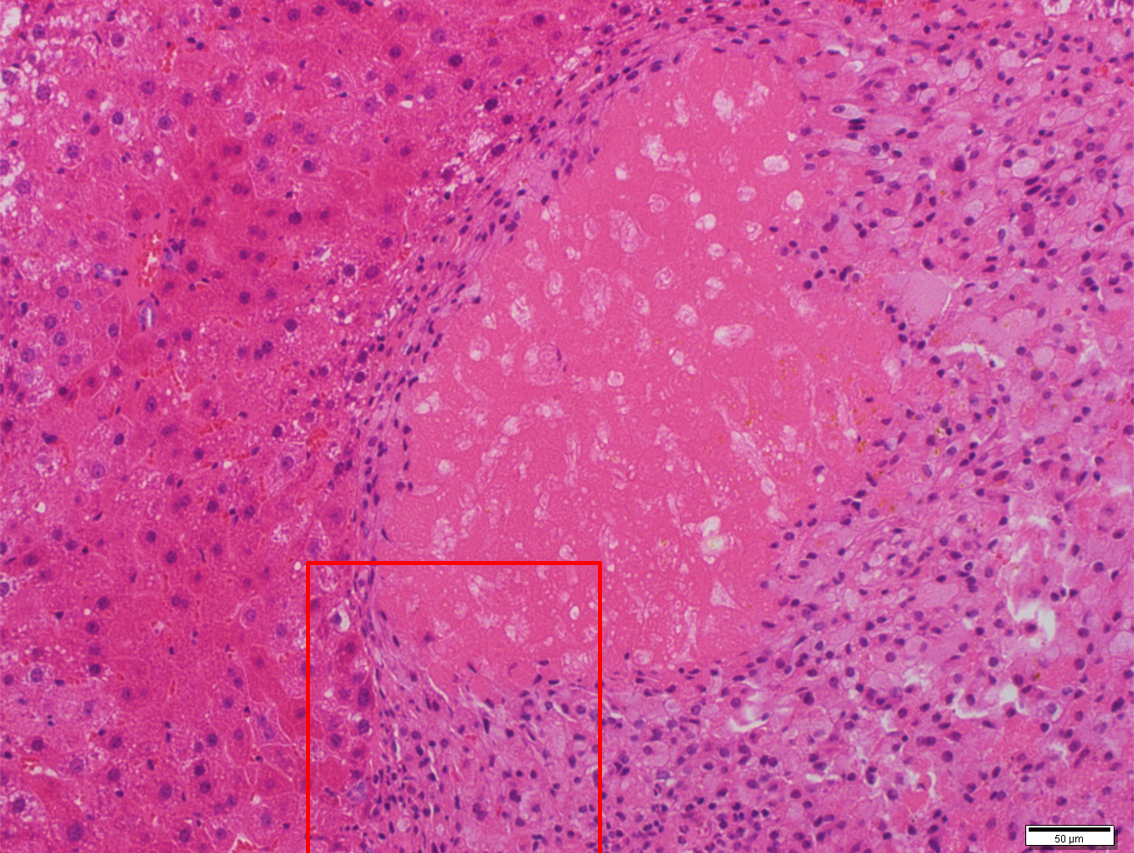

Supplement: Supplementary file 5 — Source Data for Figure 3 [file EMMM-15-e17230-s004.zip › Figure 3/3J/H&E for HFD(Diabetes&Obesity)-rAAV-2M.tif]

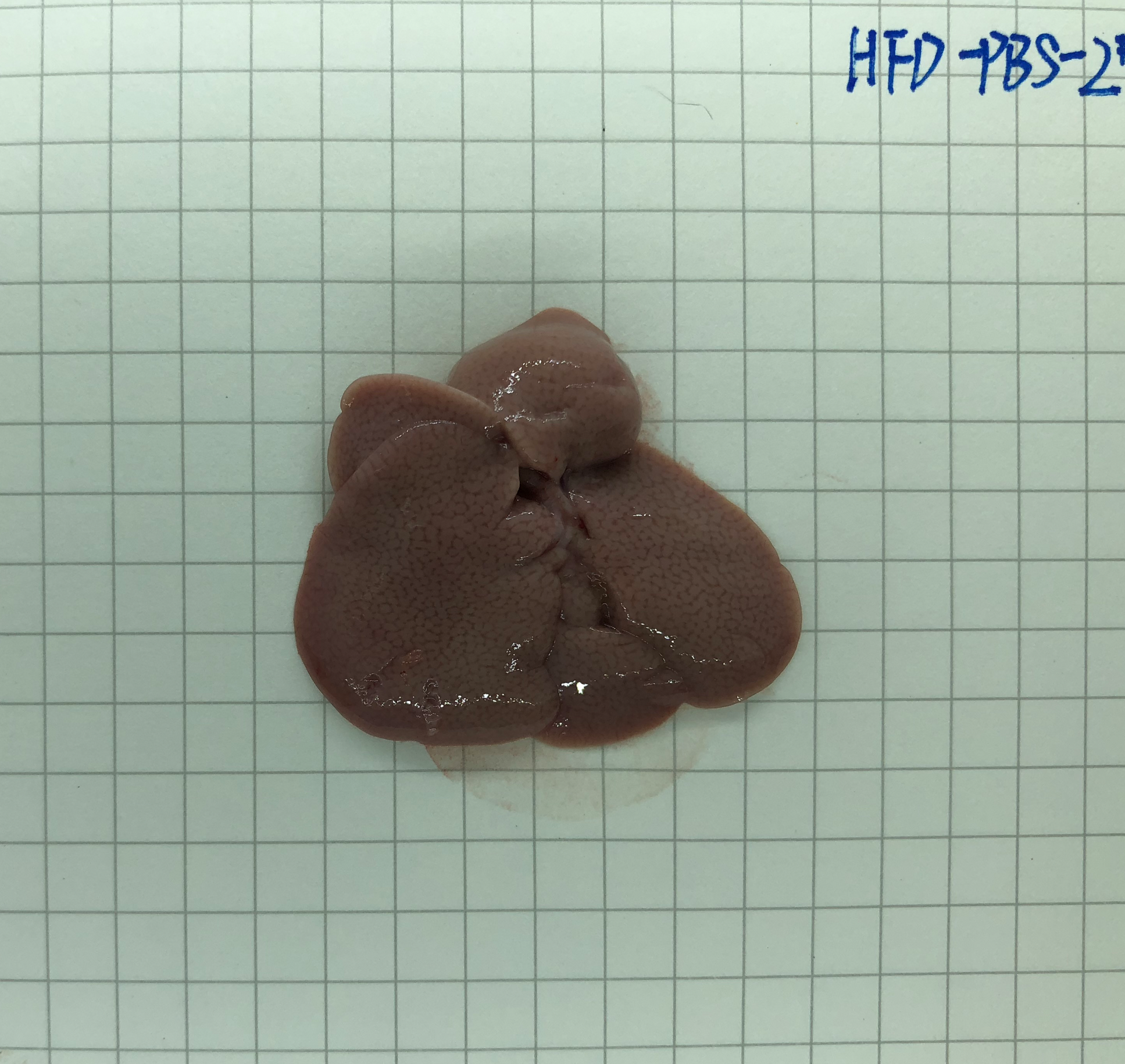

Supplement: Supplementary file 5 — Source Data for Figure 3 [file EMMM-15-e17230-s004.zip › Figure 3/3J/HFD(Diabetes&Obesity)-PBS-2M-Liver.tif]

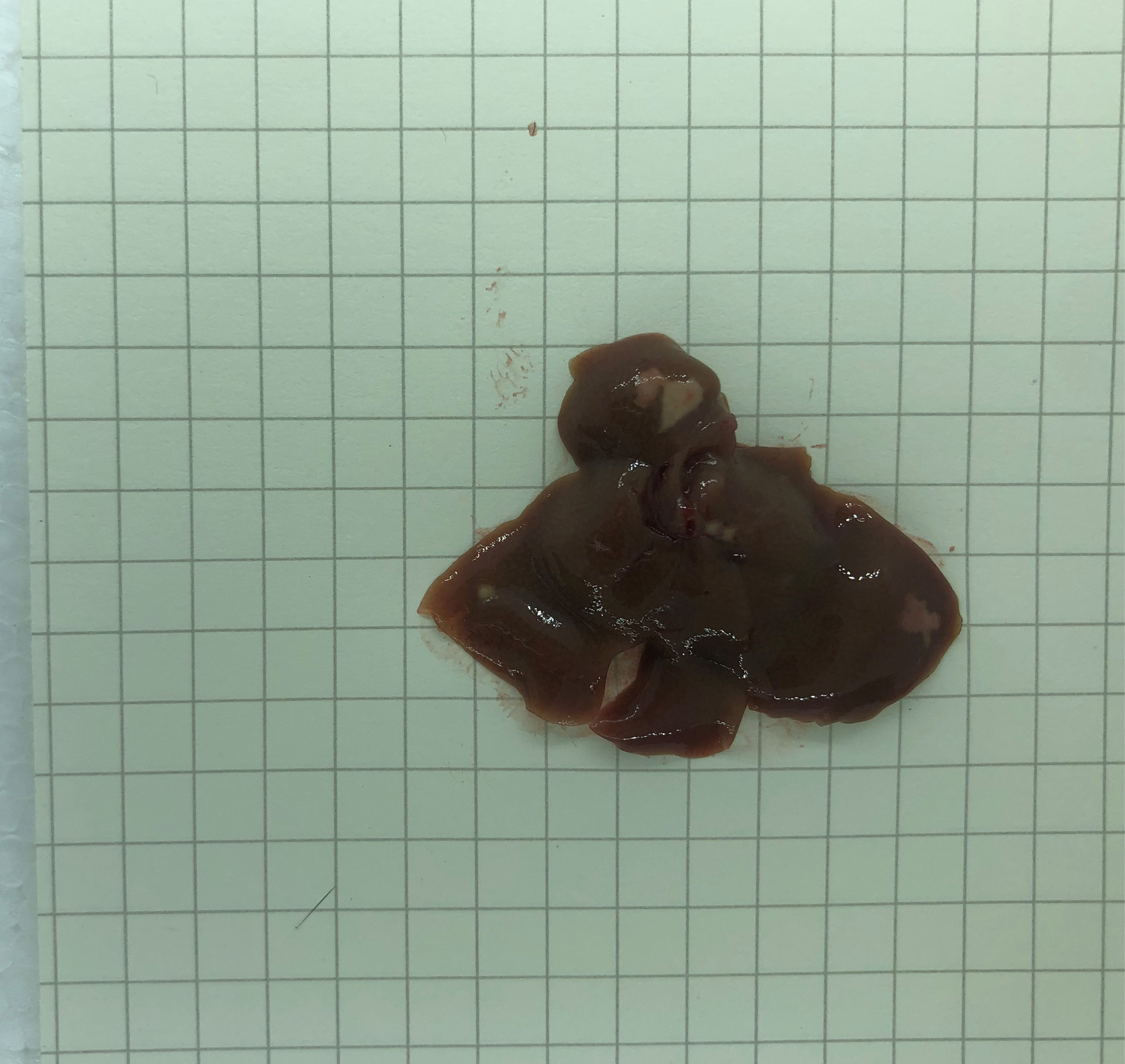

Supplement: Supplementary file 5 — Source Data for Figure 3 [file EMMM-15-e17230-s004.zip › Figure 3/3J/HFD(Diabetes&Obesity)-rAAV-2M-Liver.tif]

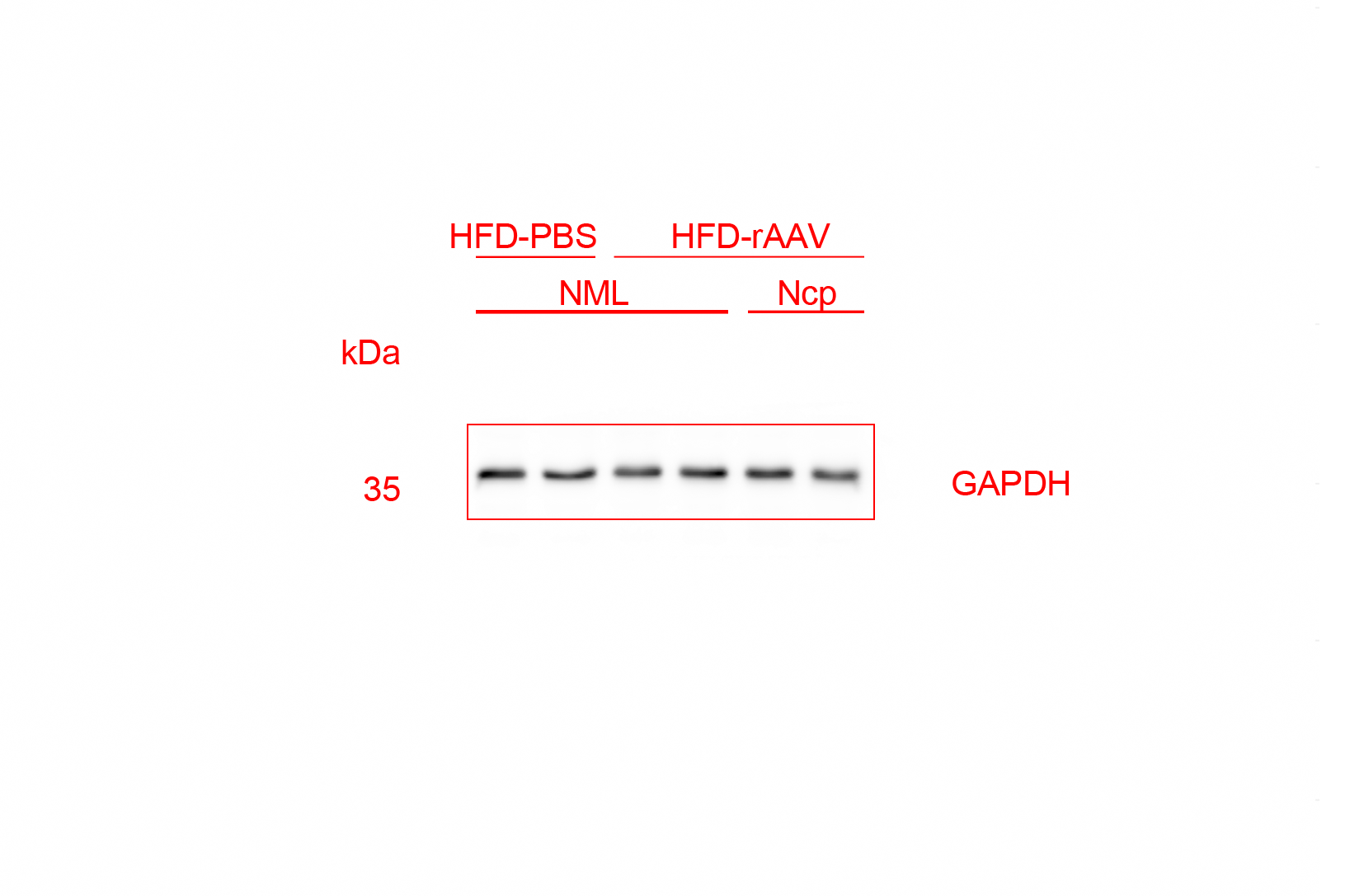

Supplement: Supplementary file 5 — Source Data for Figure 3 [file EMMM-15-e17230-s004.zip › Figure 3/3K/Western/GAPDH(HFD-Diabetes&Obesity).tif]

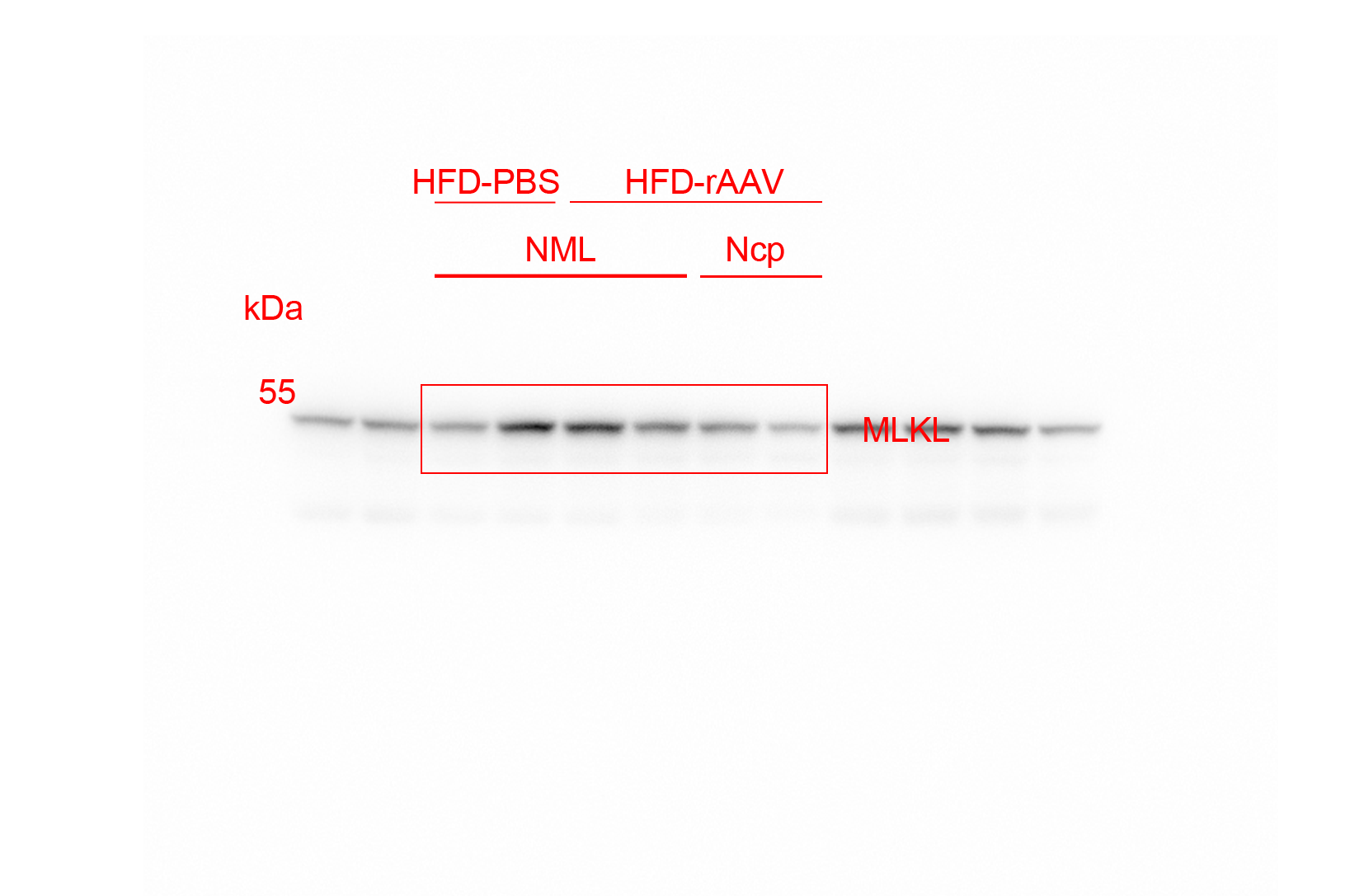

Supplement: Supplementary file 5 — Source Data for Figure 3 [file EMMM-15-e17230-s004.zip › Figure 3/3K/Western/MLKL(HFD-Diabetes&Obesity).tif]

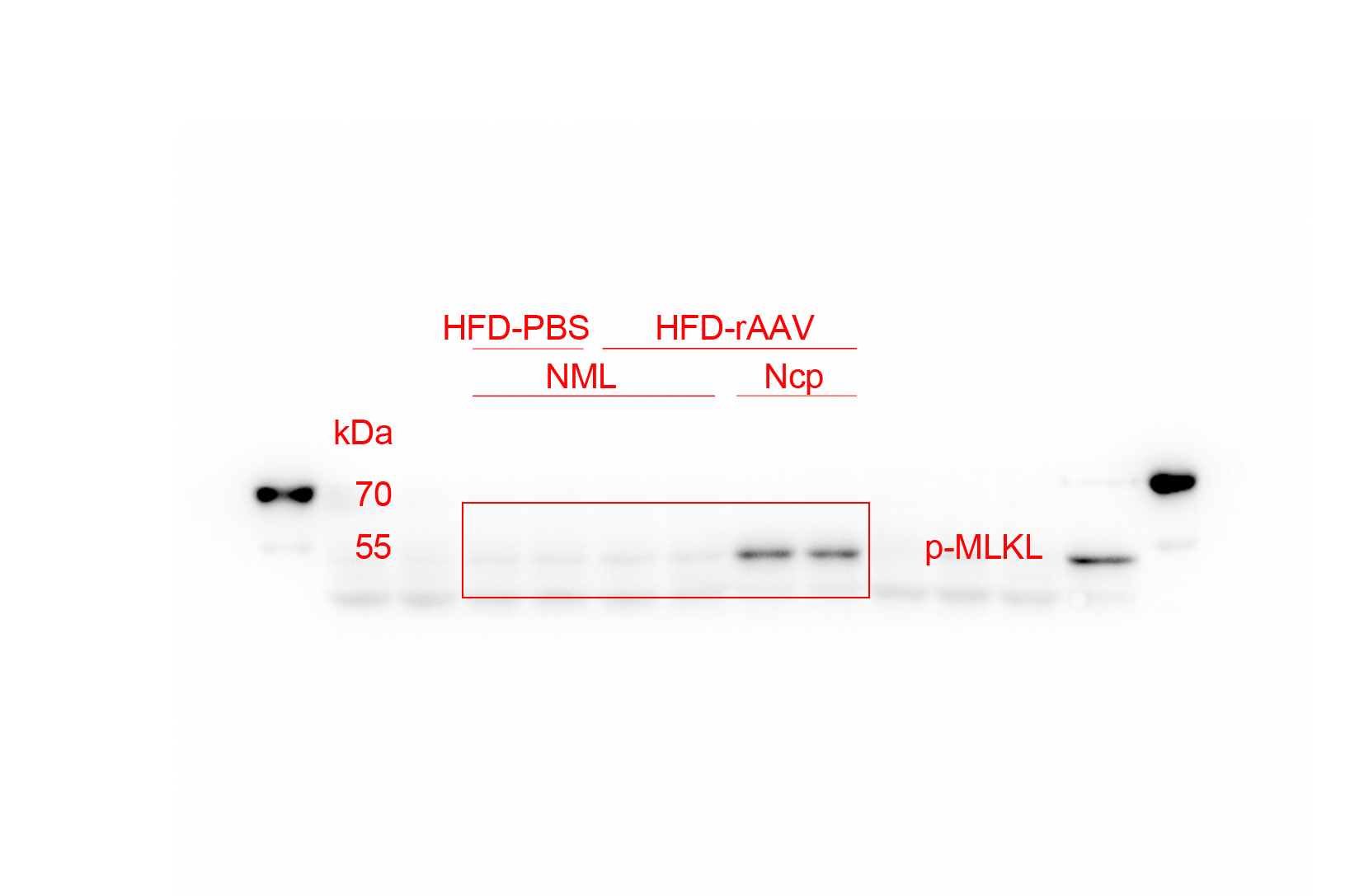

Supplement: Supplementary file 5 — Source Data for Figure 3 [file EMMM-15-e17230-s004.zip › Figure 3/3K/Western/p-MLKL(HFD-Diabetes&Obesity).tif]

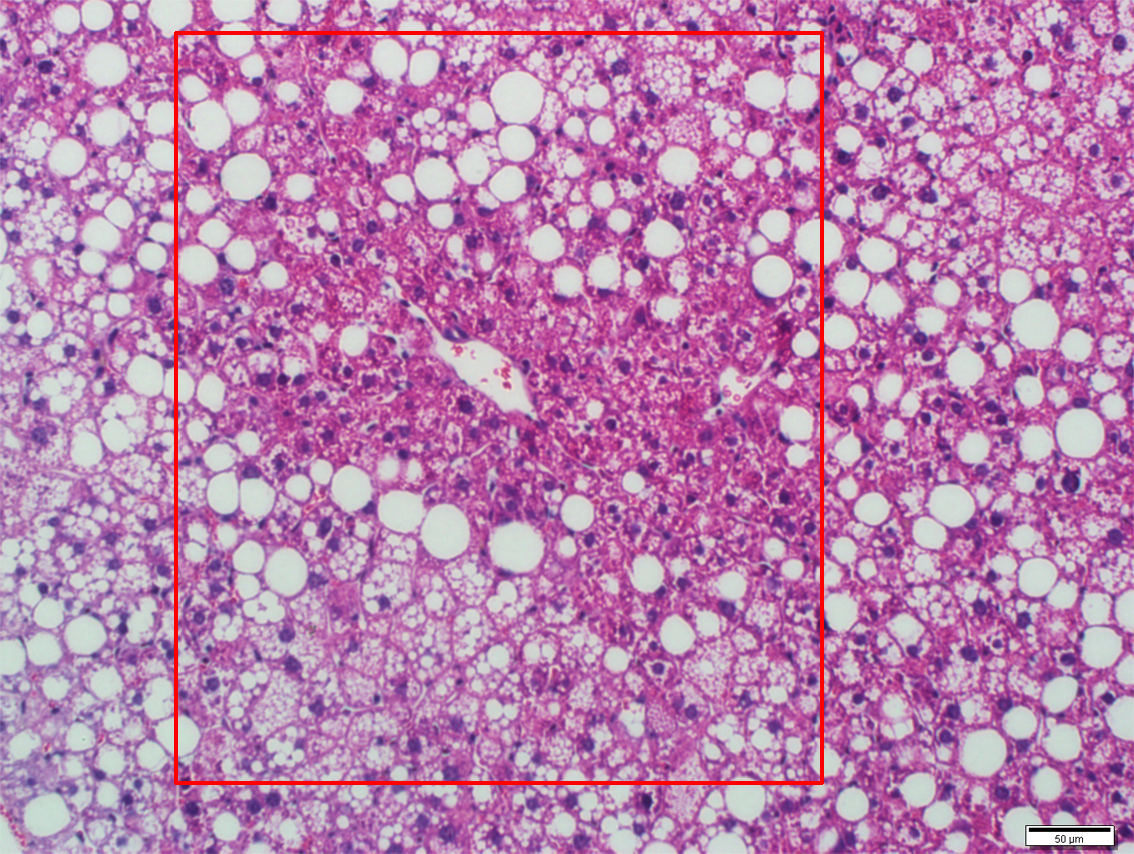

Supplement: Supplementary file 5 — Source Data for Figure 3 [file EMMM-15-e17230-s004.zip › Figure 3/3R/H&E for HFD(Diabetes&Obesity)-PBS-6M.tif]

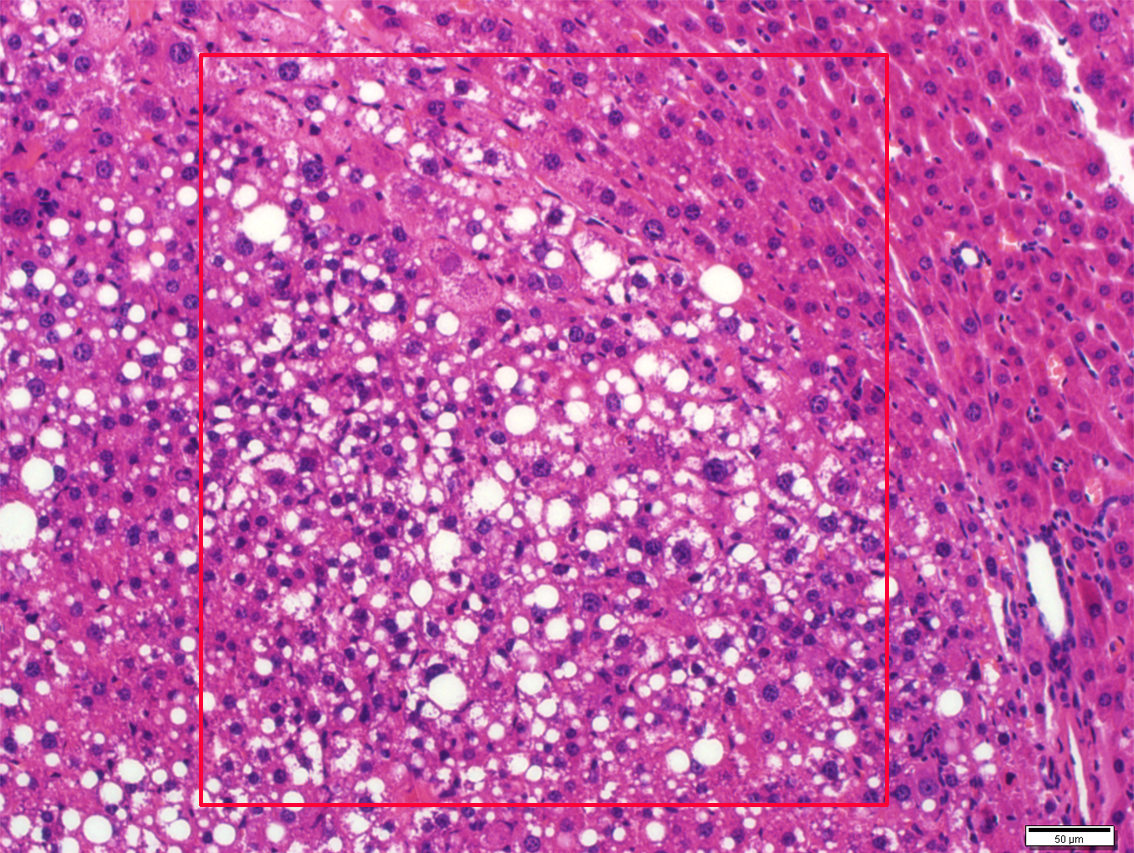

Supplement: Supplementary file 5 — Source Data for Figure 3 [file EMMM-15-e17230-s004.zip › Figure 3/3R/H&E for HFD(Diabetes&Obesity)-rAAV-6M.tif]

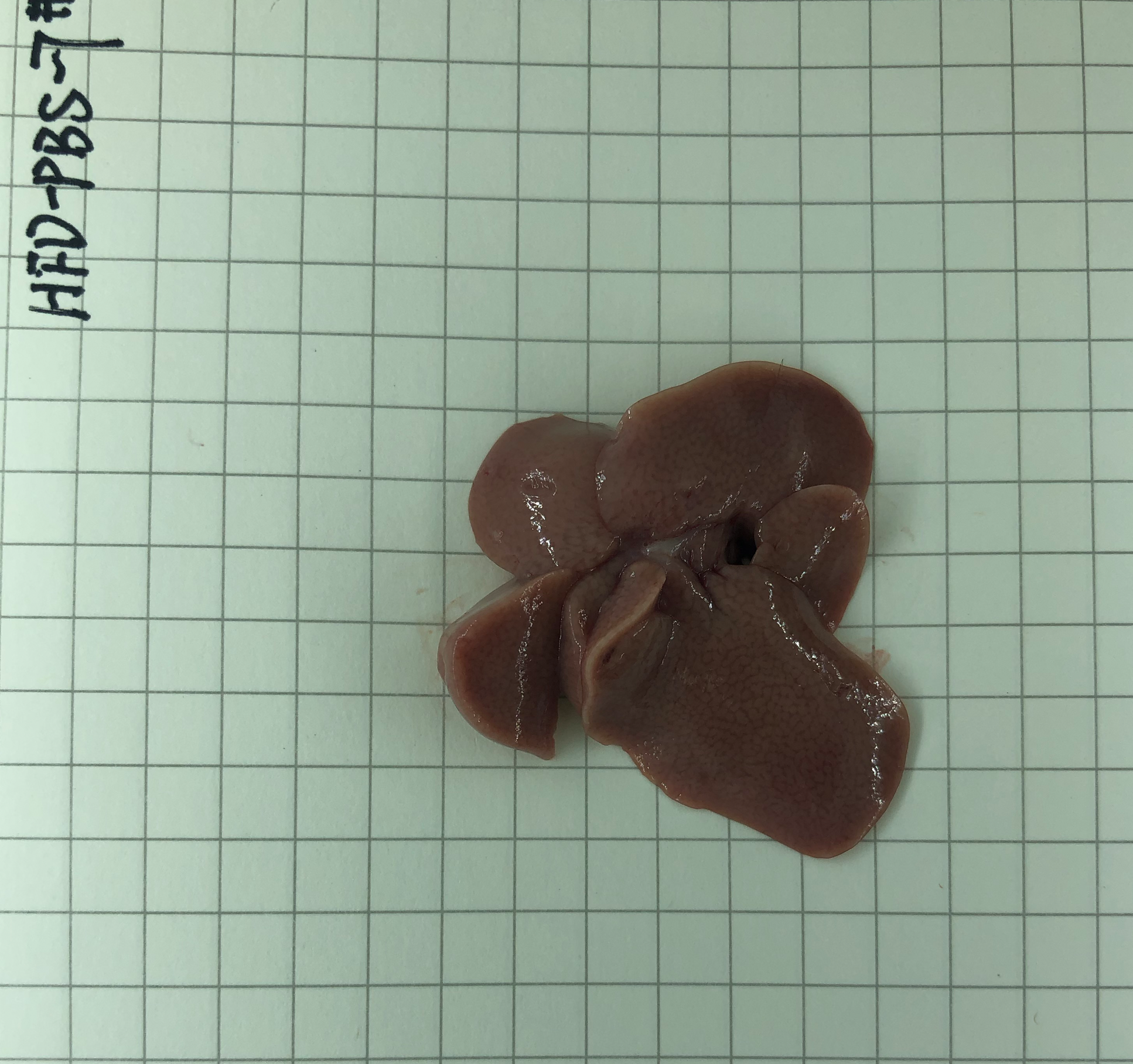

Supplement: Supplementary file 5 — Source Data for Figure 3 [file EMMM-15-e17230-s004.zip › Figure 3/3R/HFD(Diabetes&Obesity)-PBS-6M-Liver.tif]

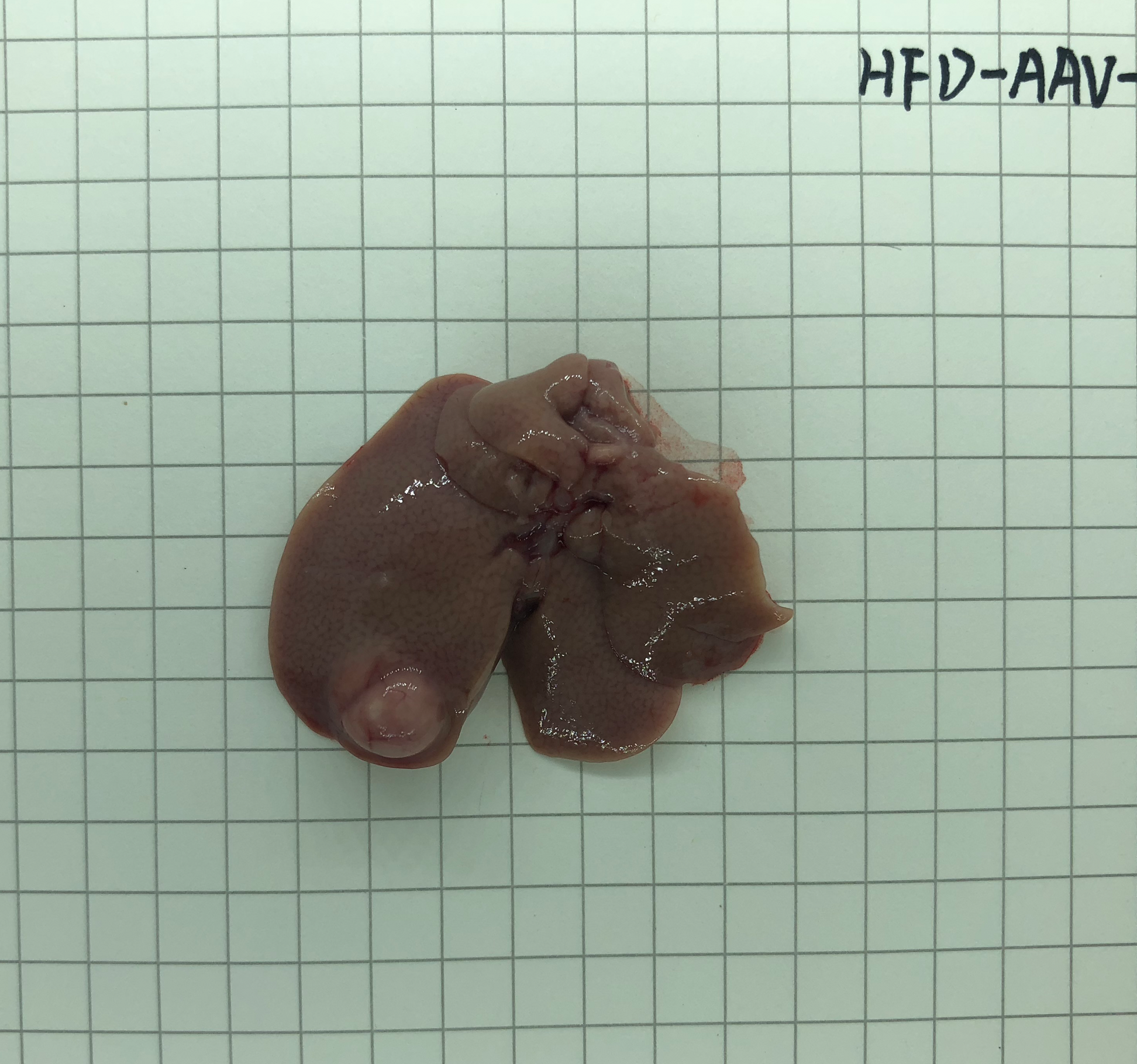

Supplement: Supplementary file 5 — Source Data for Figure 3 [file EMMM-15-e17230-s004.zip › Figure 3/3R/HFD(Diabetes&Obesity)-rAAV-6M-Liver.tif]

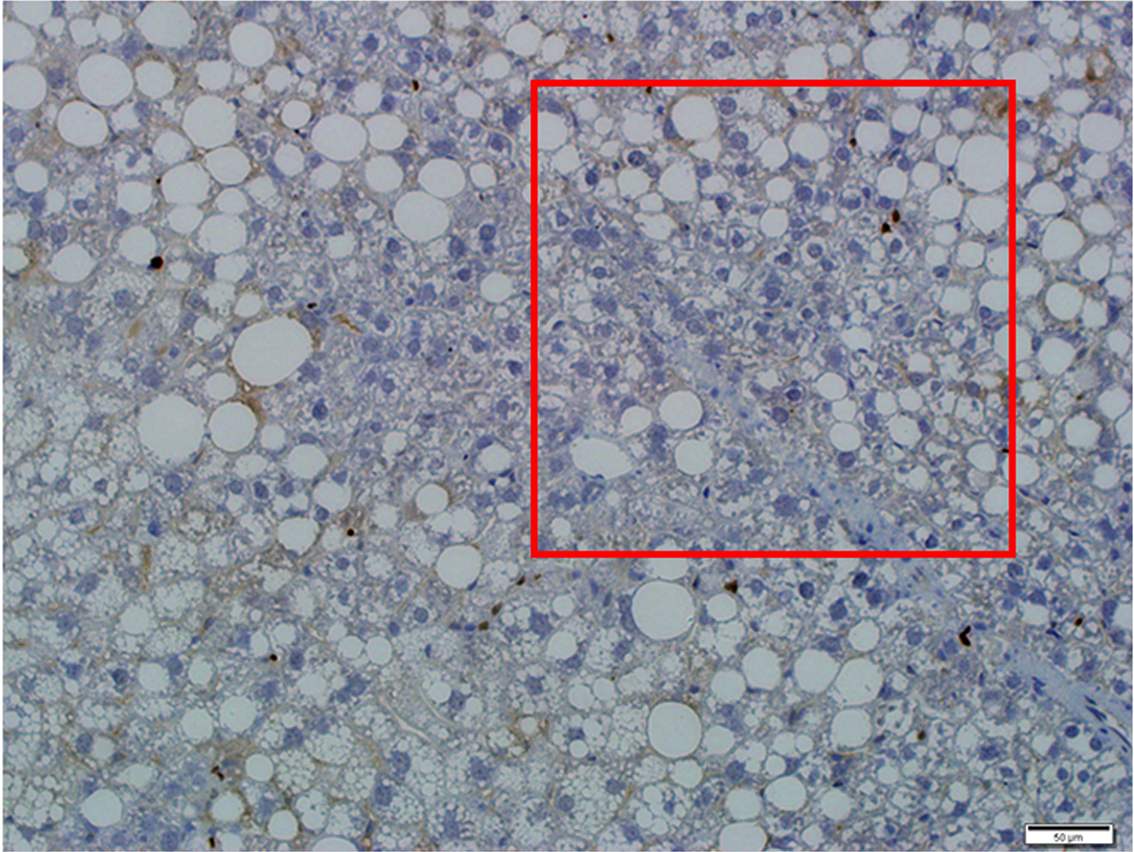

Supplement: Supplementary file 5 — Source Data for Figure 3 [file EMMM-15-e17230-s004.zip › Figure 3/3R/Ki67 staining for HFD(Diabetes&Obesity)-PBS-6M.tif]

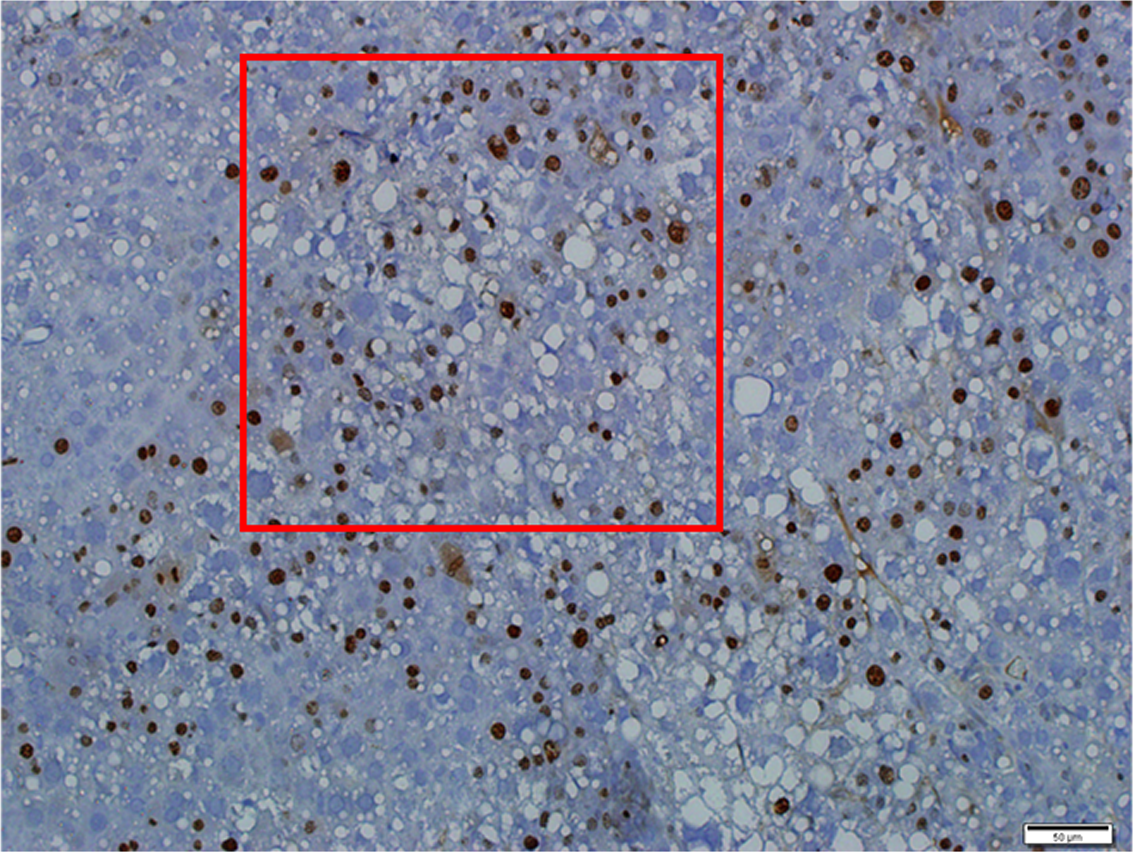

Supplement: Supplementary file 5 — Source Data for Figure 3 [file EMMM-15-e17230-s004.zip › Figure 3/3R/Ki67 staining for HFD(Diabetes&Obesity)-rAAV-6M.tif]

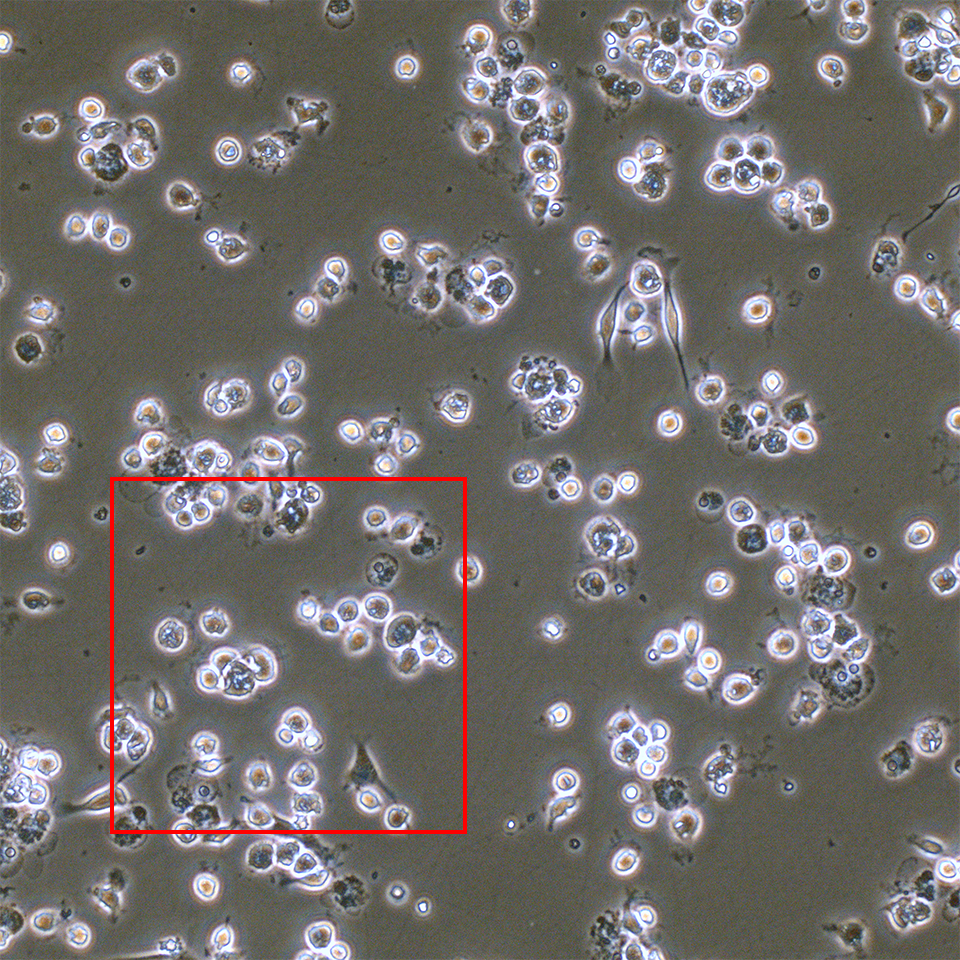

Supplement: Supplementary file 6 — Source Data for Figure 4 [file EMMM-15-e17230-s010.zip › Figure 4/4B/BF for Pre+pIC+zVAD.tif]

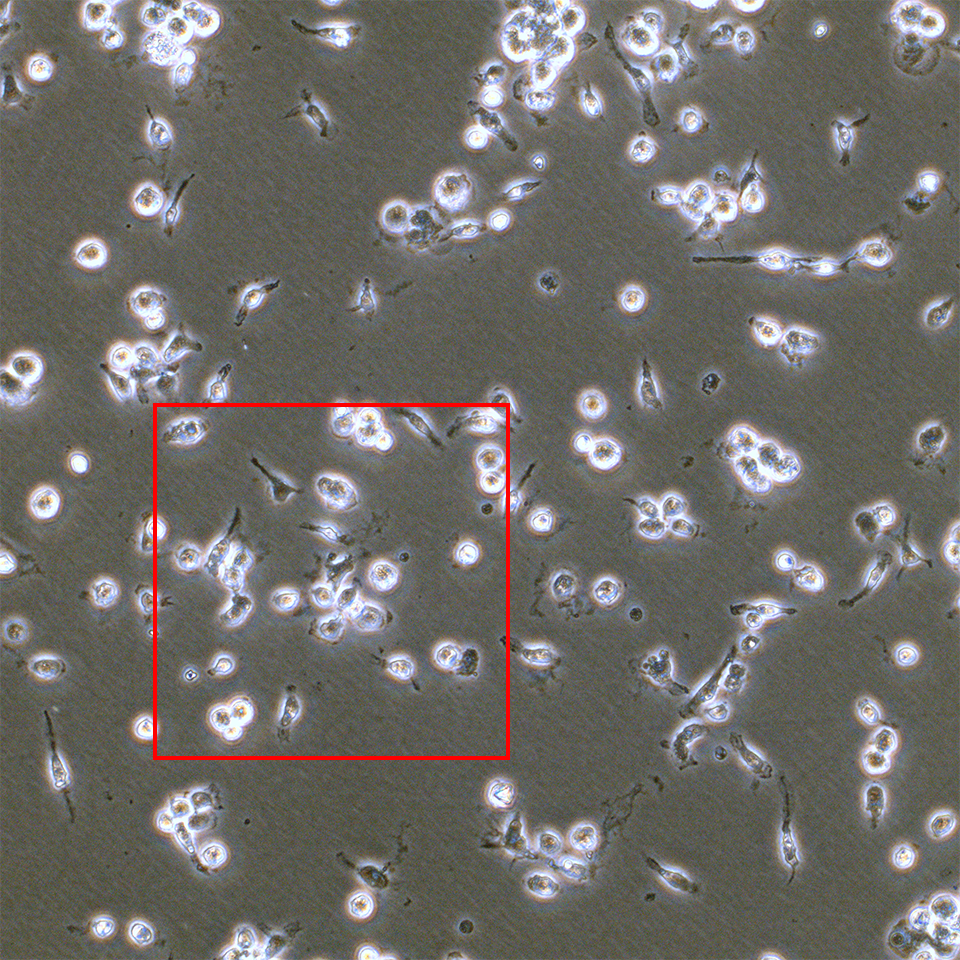

Supplement: Supplementary file 6 — Source Data for Figure 4 [file EMMM-15-e17230-s010.zip › Figure 4/4B/BF for Pre.tif]

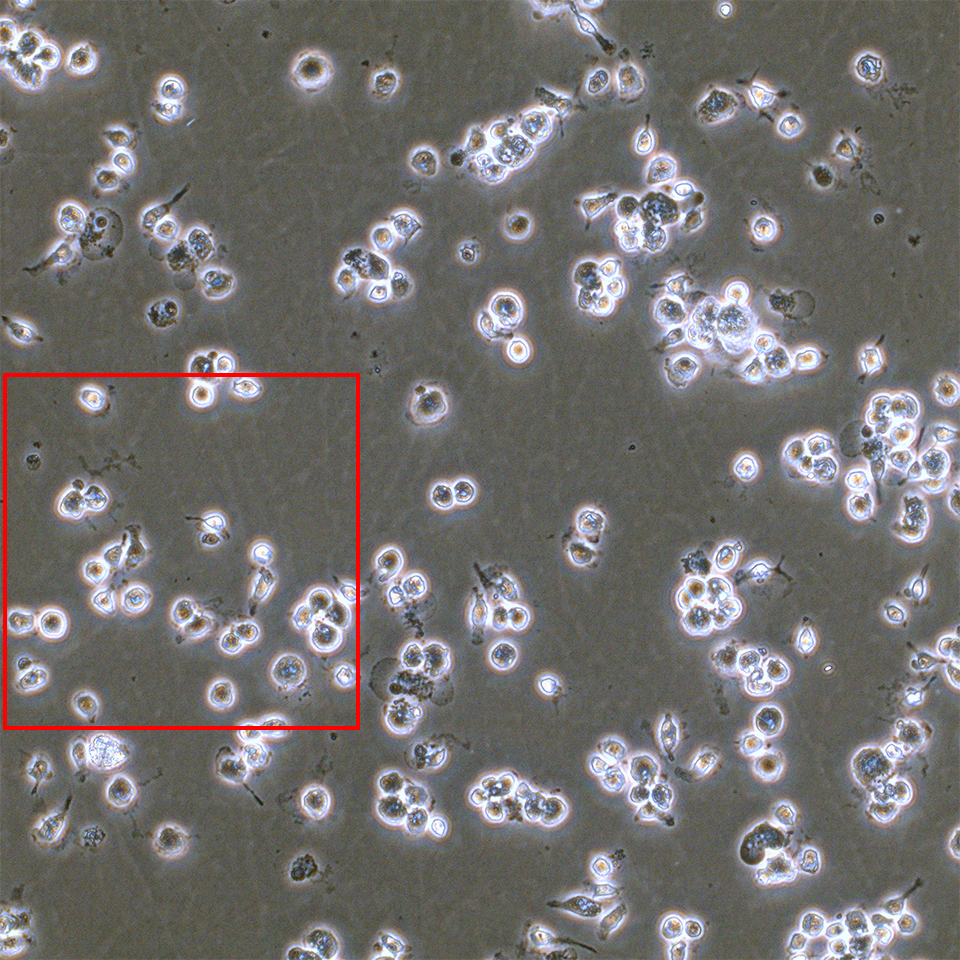

Supplement: Supplementary file 6 — Source Data for Figure 4 [file EMMM-15-e17230-s010.zip › Figure 4/4B/BF for Vehicle.tif]

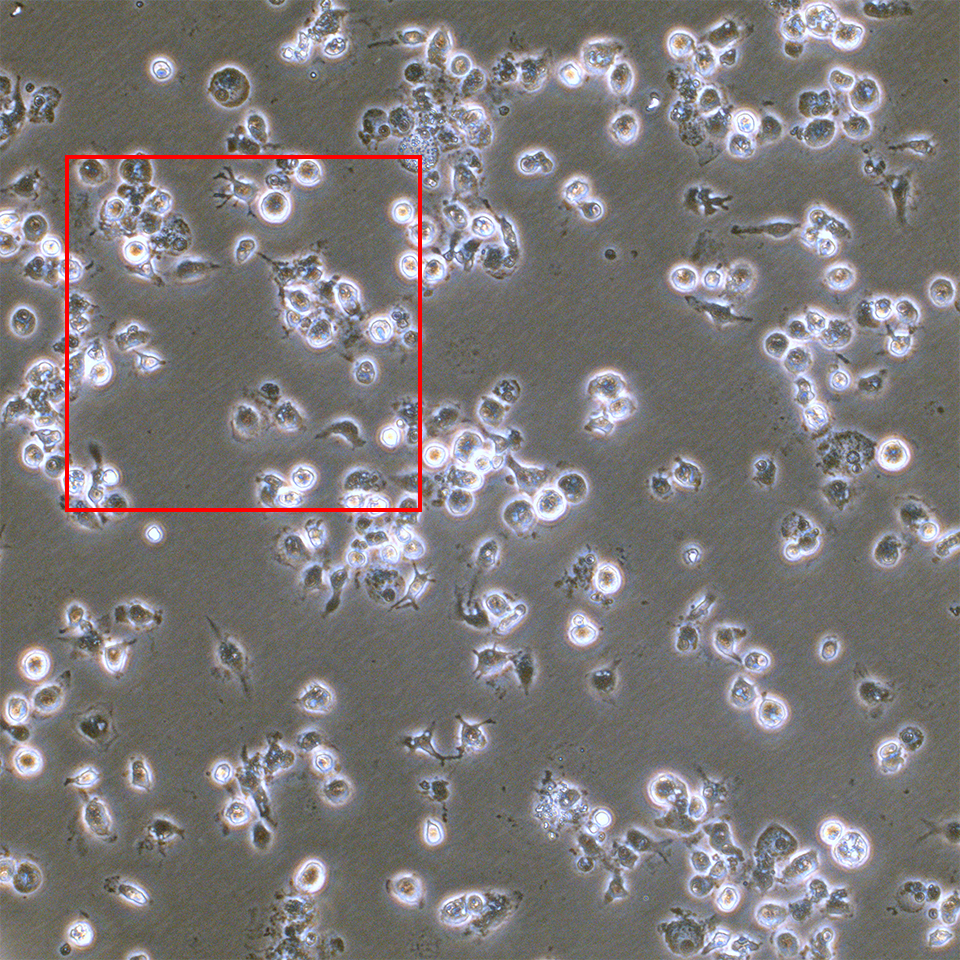

Supplement: Supplementary file 6 — Source Data for Figure 4 [file EMMM-15-e17230-s010.zip › Figure 4/4B/BF for pIC+zVAD.tif]

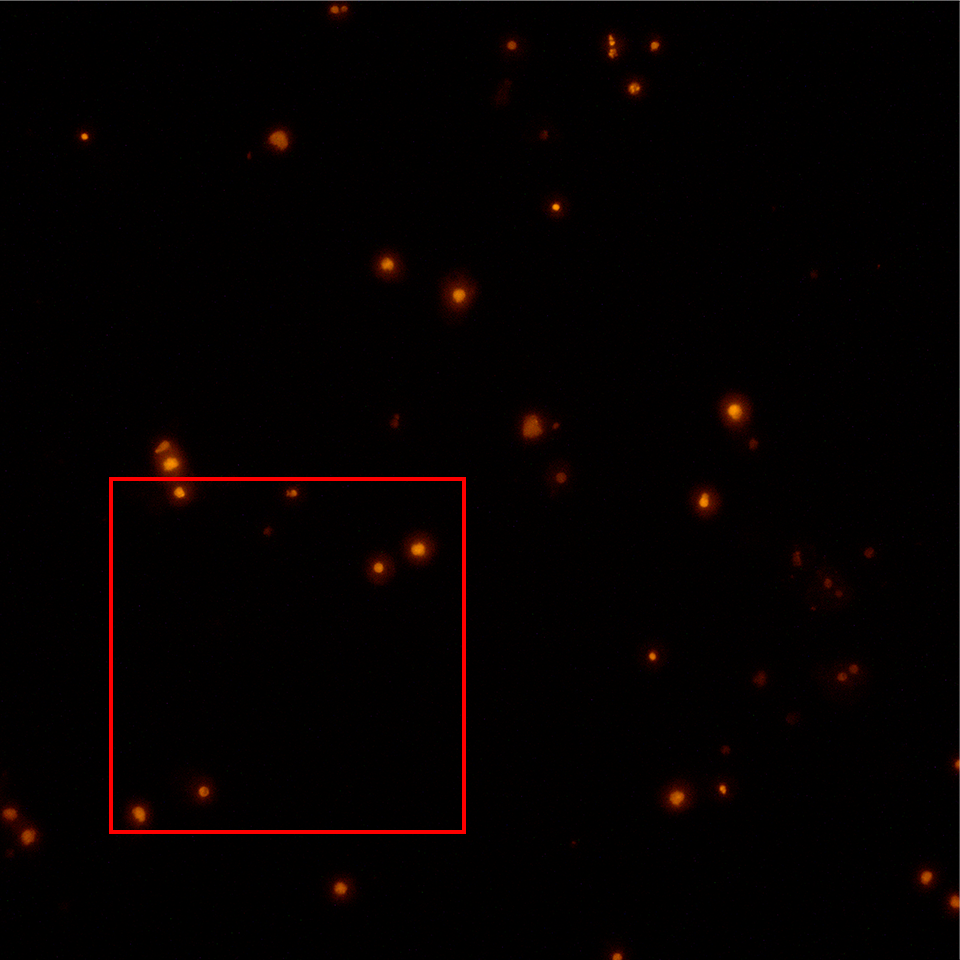

Supplement: Supplementary file 6 — Source Data for Figure 4 [file EMMM-15-e17230-s010.zip › Figure 4/4B/PI staining for Pre+pIC+zVAD.tif]

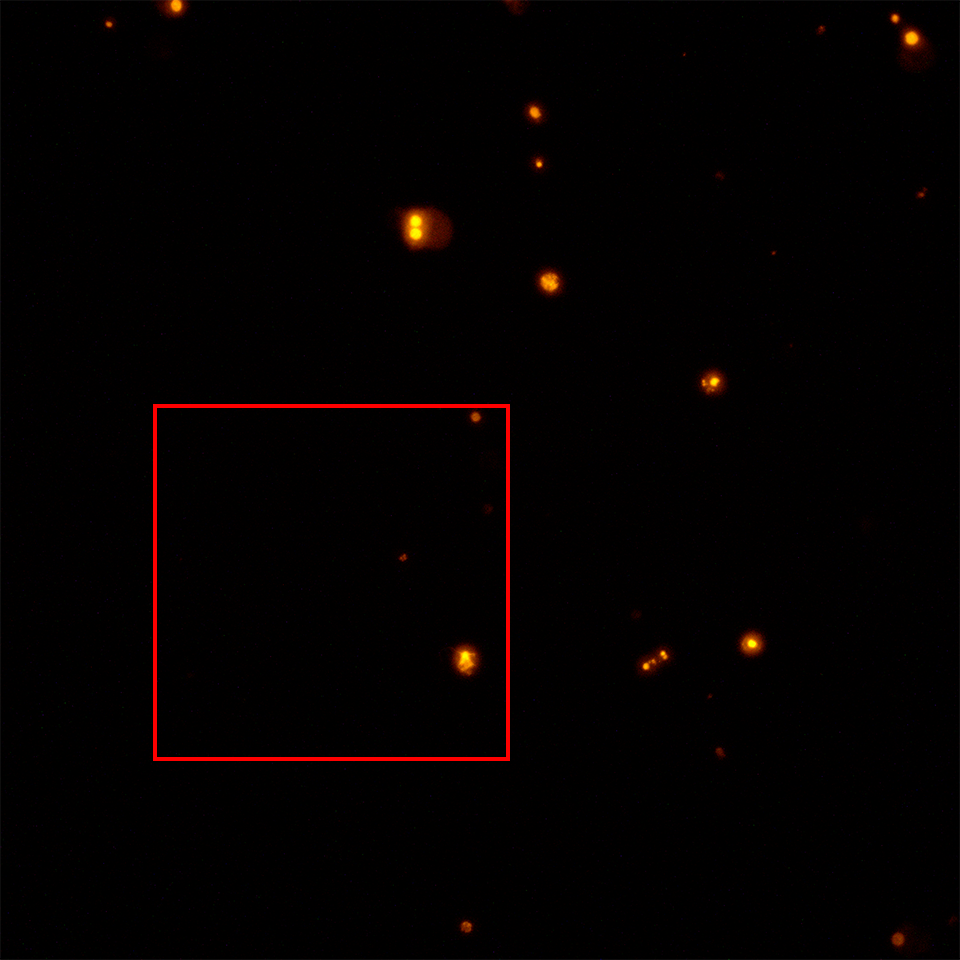

Supplement: Supplementary file 6 — Source Data for Figure 4 [file EMMM-15-e17230-s010.zip › Figure 4/4B/PI staining for Pre.tif]

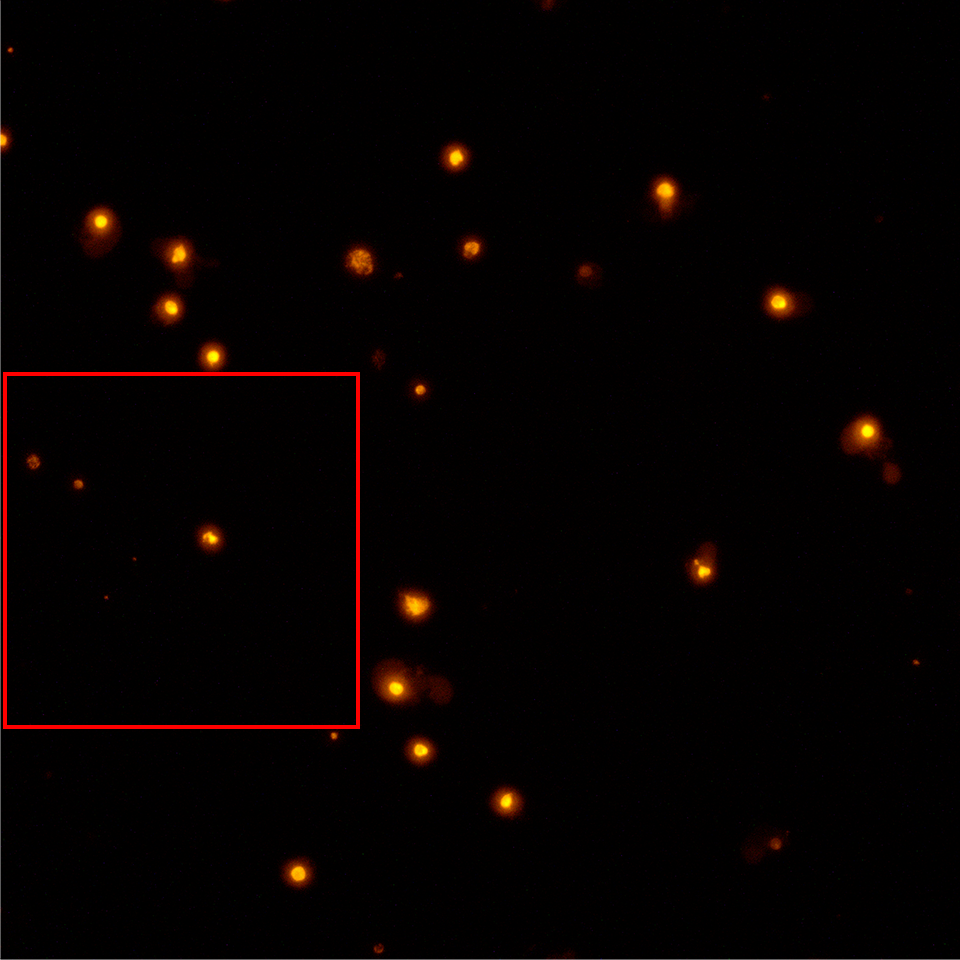

Supplement: Supplementary file 6 — Source Data for Figure 4 [file EMMM-15-e17230-s010.zip › Figure 4/4B/PI staining for Vehicle.tif]

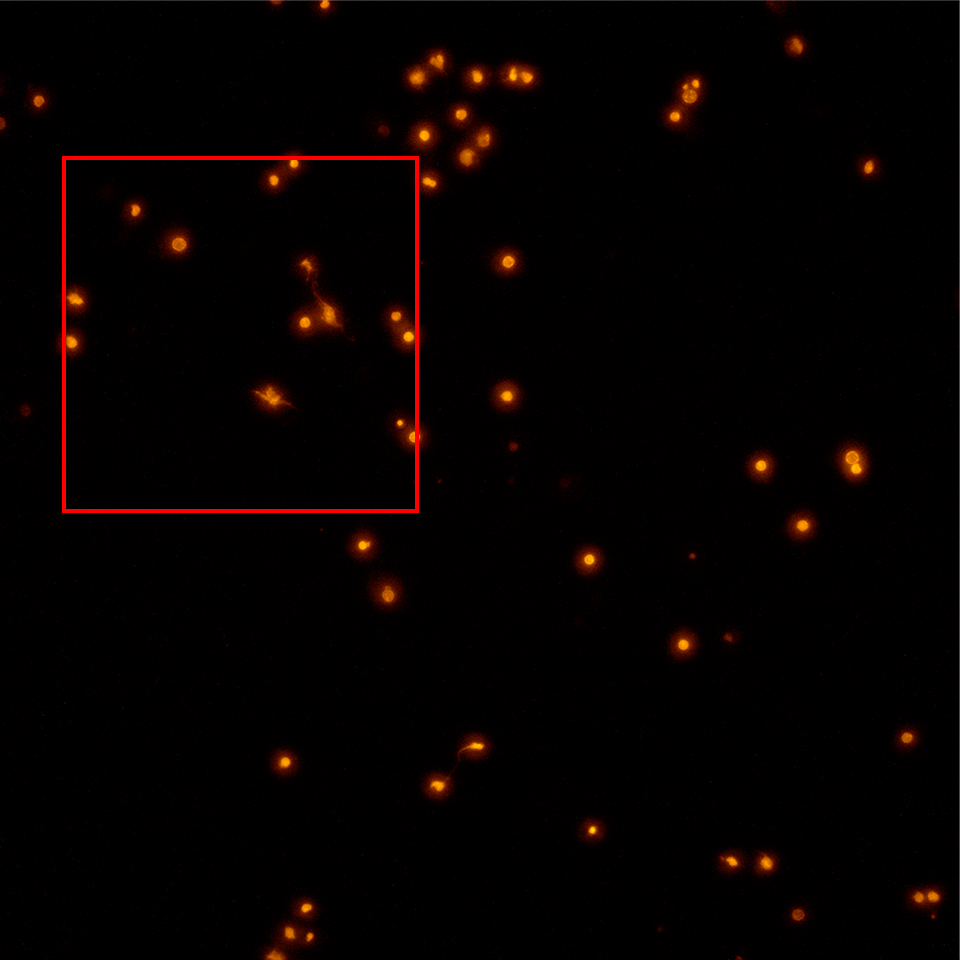

Supplement: Supplementary file 6 — Source Data for Figure 4 [file EMMM-15-e17230-s010.zip › Figure 4/4B/PI staining for pIC+zVAD.tif]

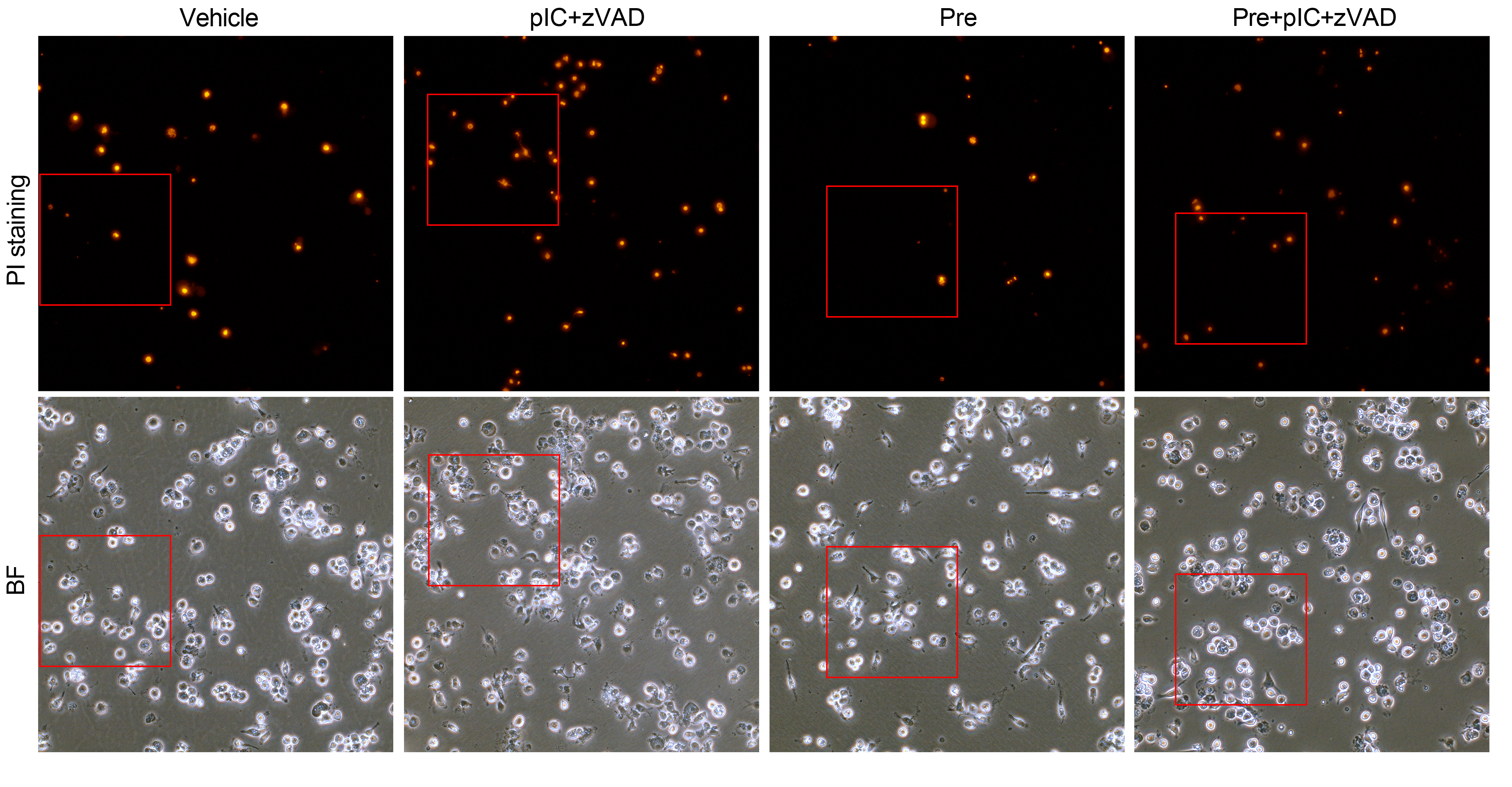

Supplement: Supplementary file 6 — Source Data for Figure 4 [file EMMM-15-e17230-s010.zip › Figure 4/4B/PI staining.tif]

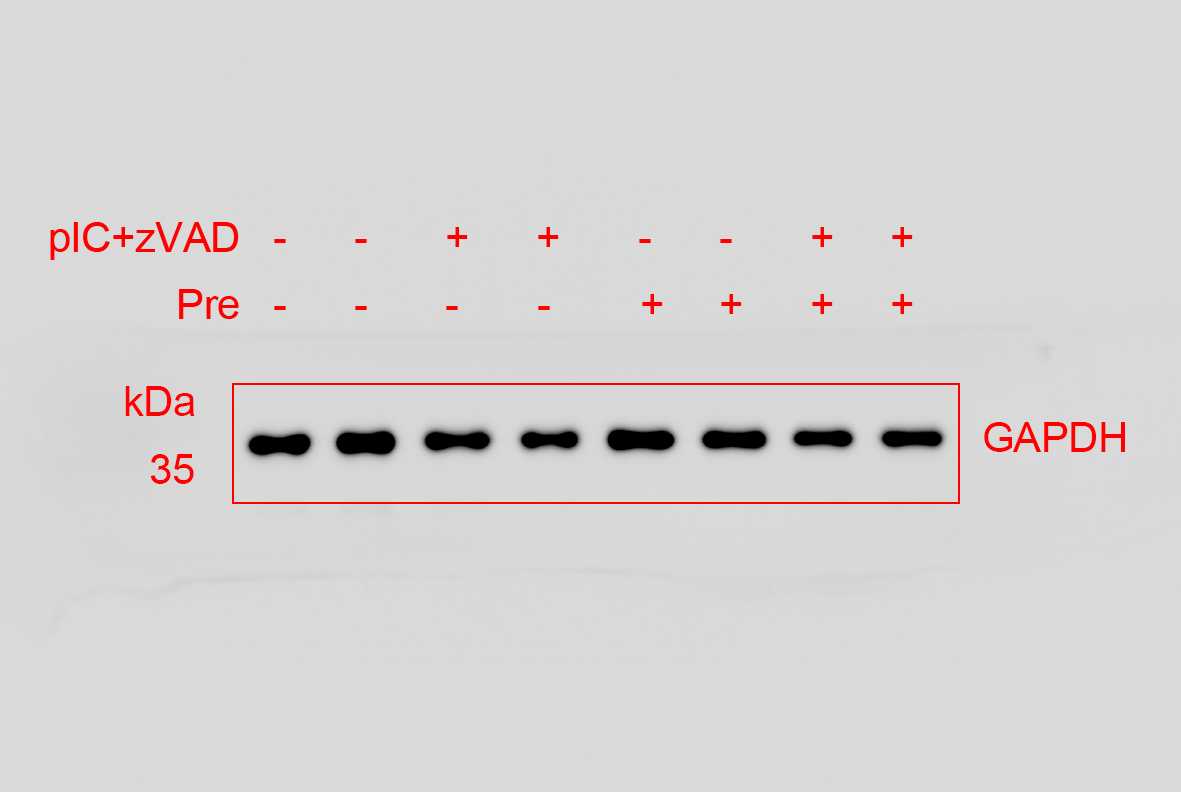

Supplement: Supplementary file 6 — Source Data for Figure 4 [file EMMM-15-e17230-s010.zip › Figure 4/4C/Western/GAPDH.tif]

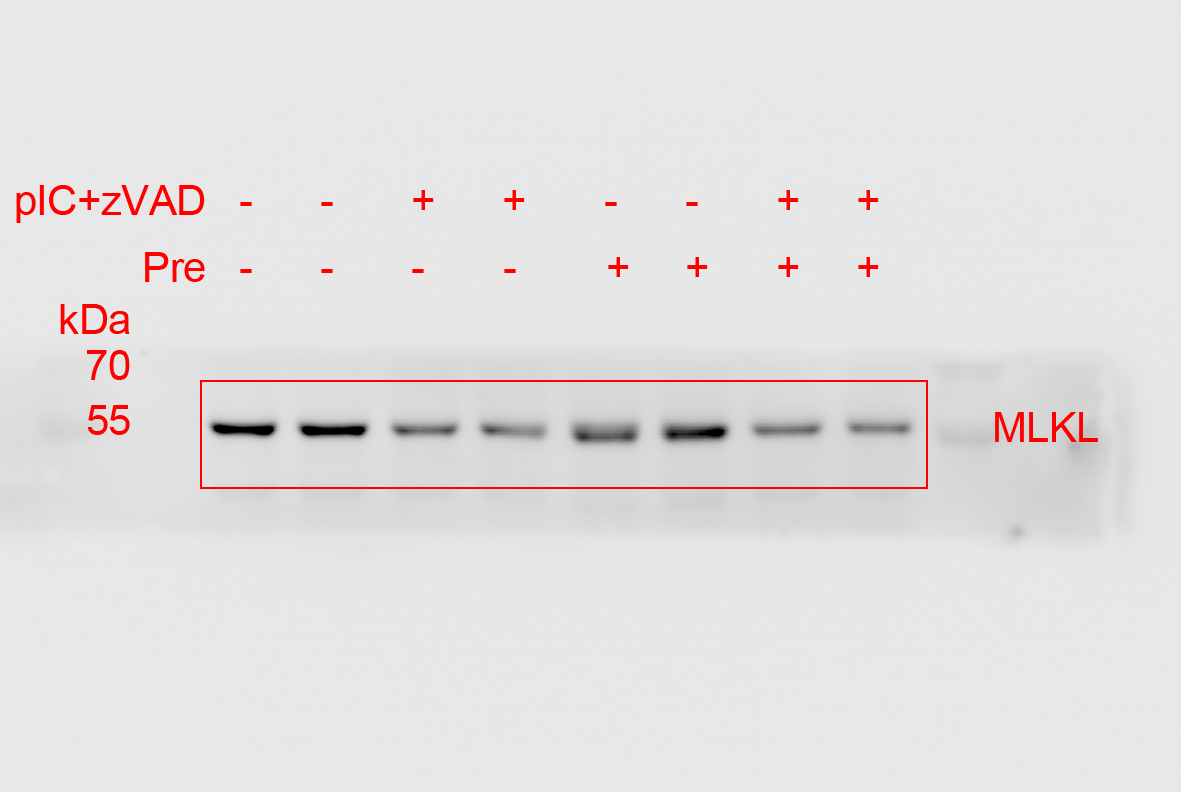

Supplement: Supplementary file 6 — Source Data for Figure 4 [file EMMM-15-e17230-s010.zip › Figure 4/4C/Western/MLKL.tif]

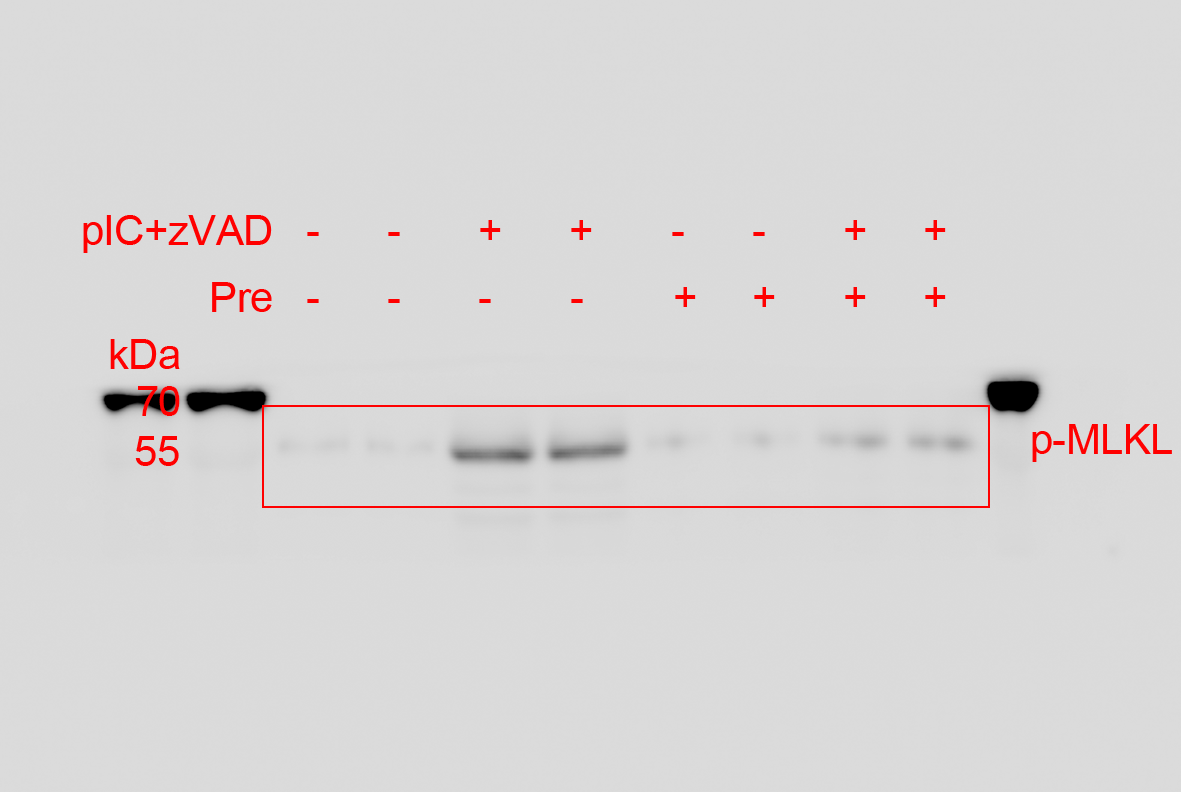

Supplement: Supplementary file 6 — Source Data for Figure 4 [file EMMM-15-e17230-s010.zip › Figure 4/4C/Western/p-MLKL.tif]

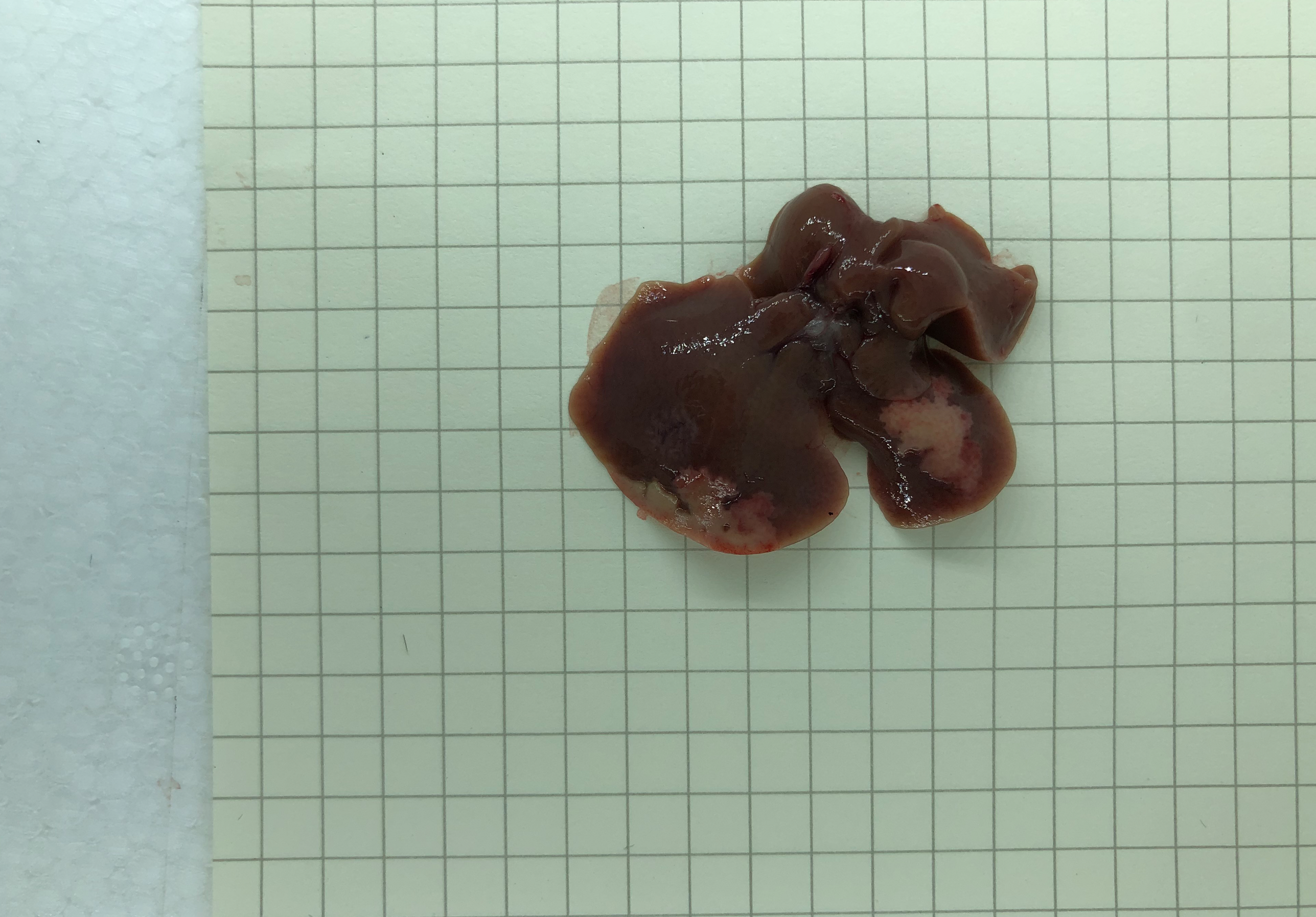

Supplement: Supplementary file 6 — Source Data for Figure 4 [file EMMM-15-e17230-s010.zip › Figure 4/4J/Liver image(DB-rAAV+Prednisone-1#).tif]

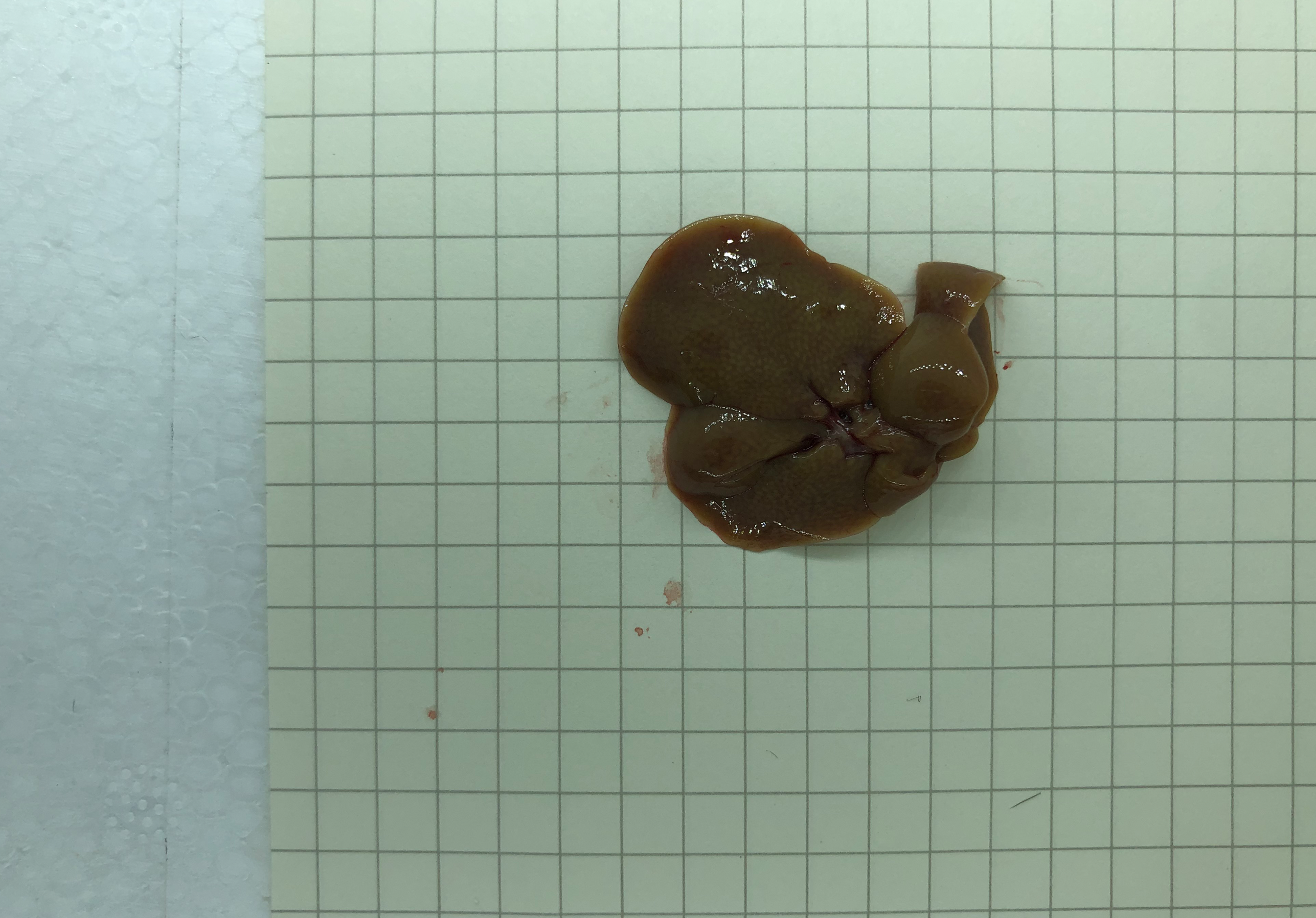

Supplement: Supplementary file 6 — Source Data for Figure 4 [file EMMM-15-e17230-s010.zip › Figure 4/4J/Liver image(DB-rAAV+Prednisone-10#).tif]

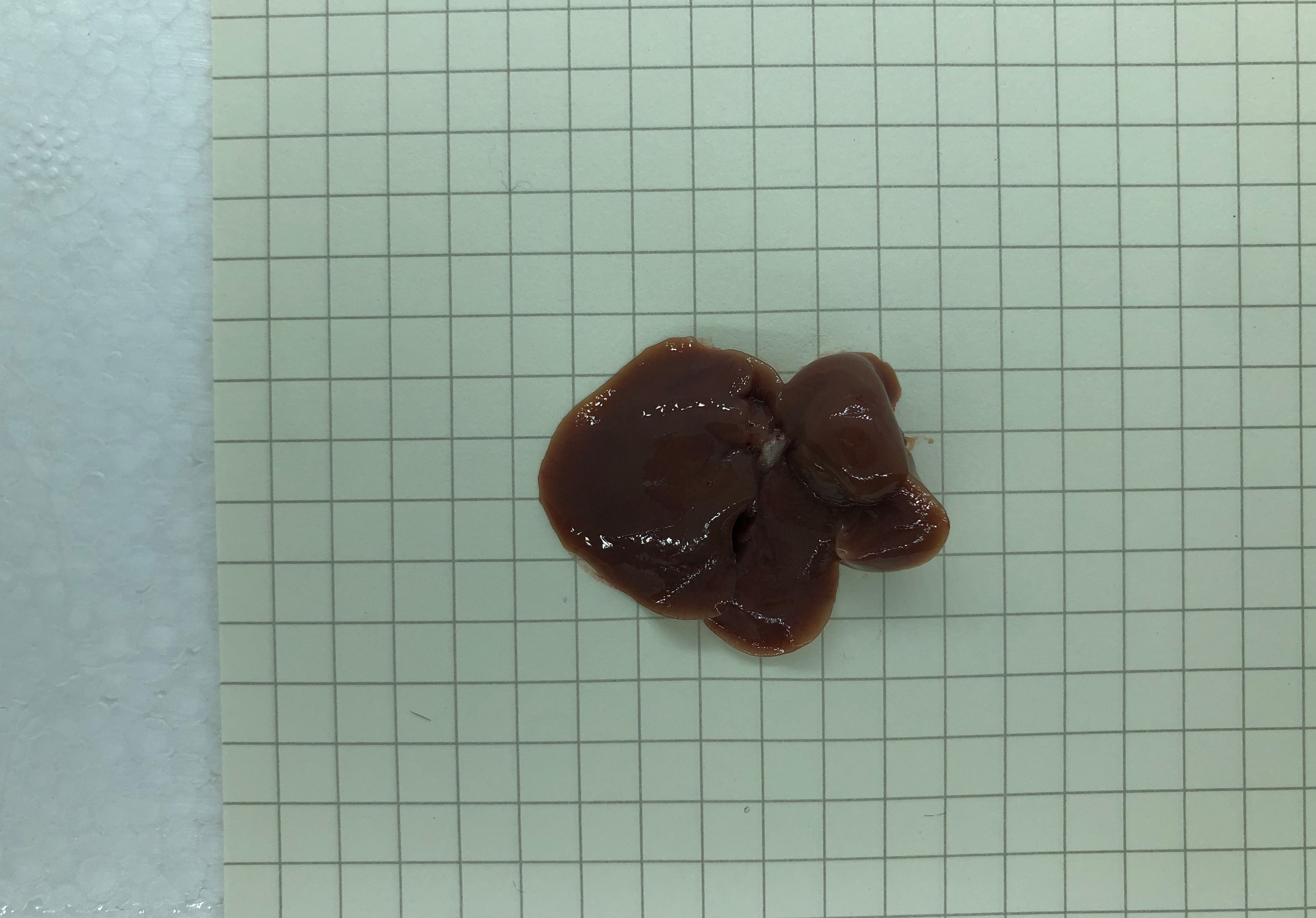

Supplement: Supplementary file 6 — Source Data for Figure 4 [file EMMM-15-e17230-s010.zip › Figure 4/4J/Liver image(DB-rAAV+Prednisone-11#).tif]

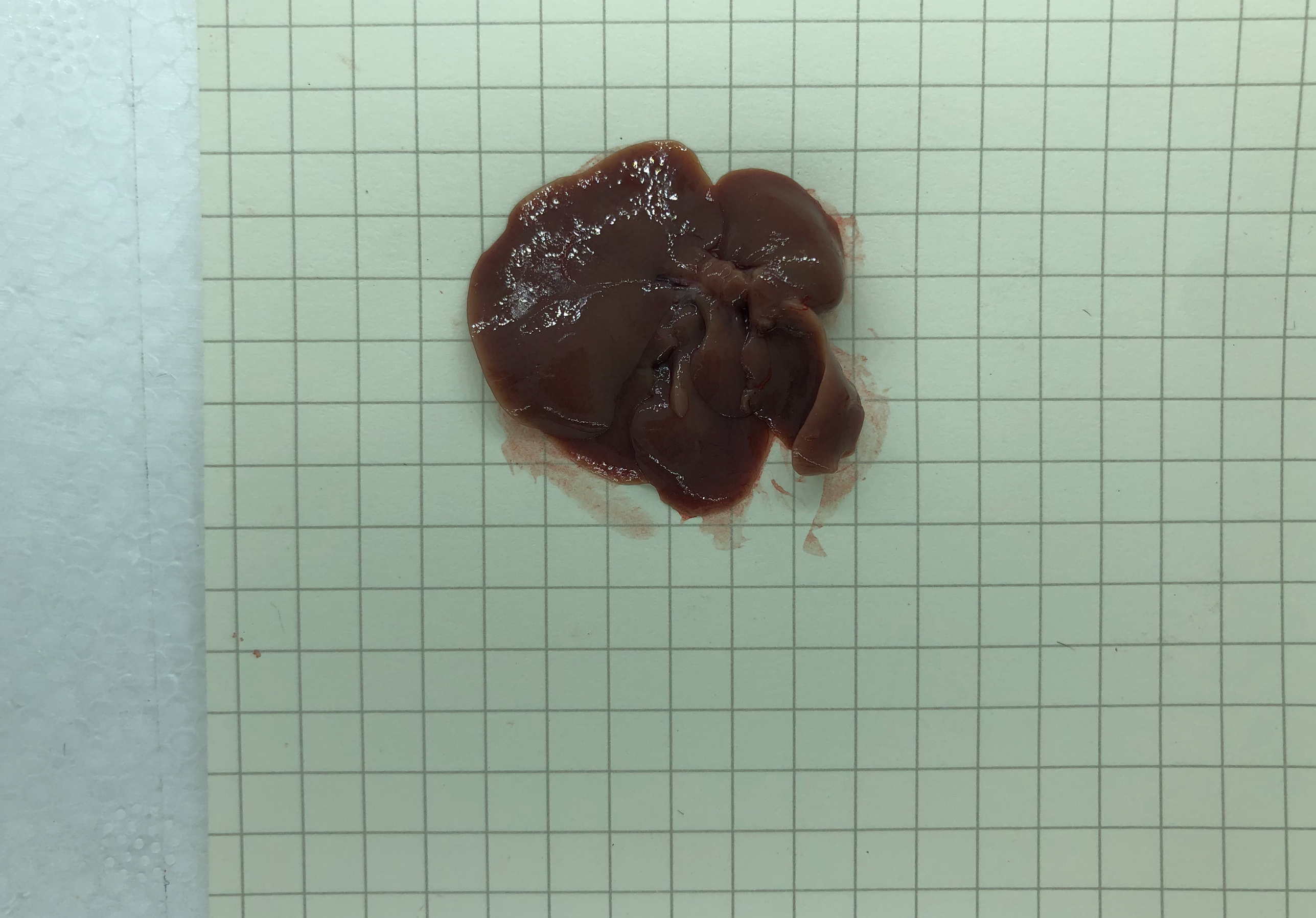

Supplement: Supplementary file 6 — Source Data for Figure 4 [file EMMM-15-e17230-s010.zip › Figure 4/4J/Liver image(DB-rAAV+Prednisone-12#).tif]

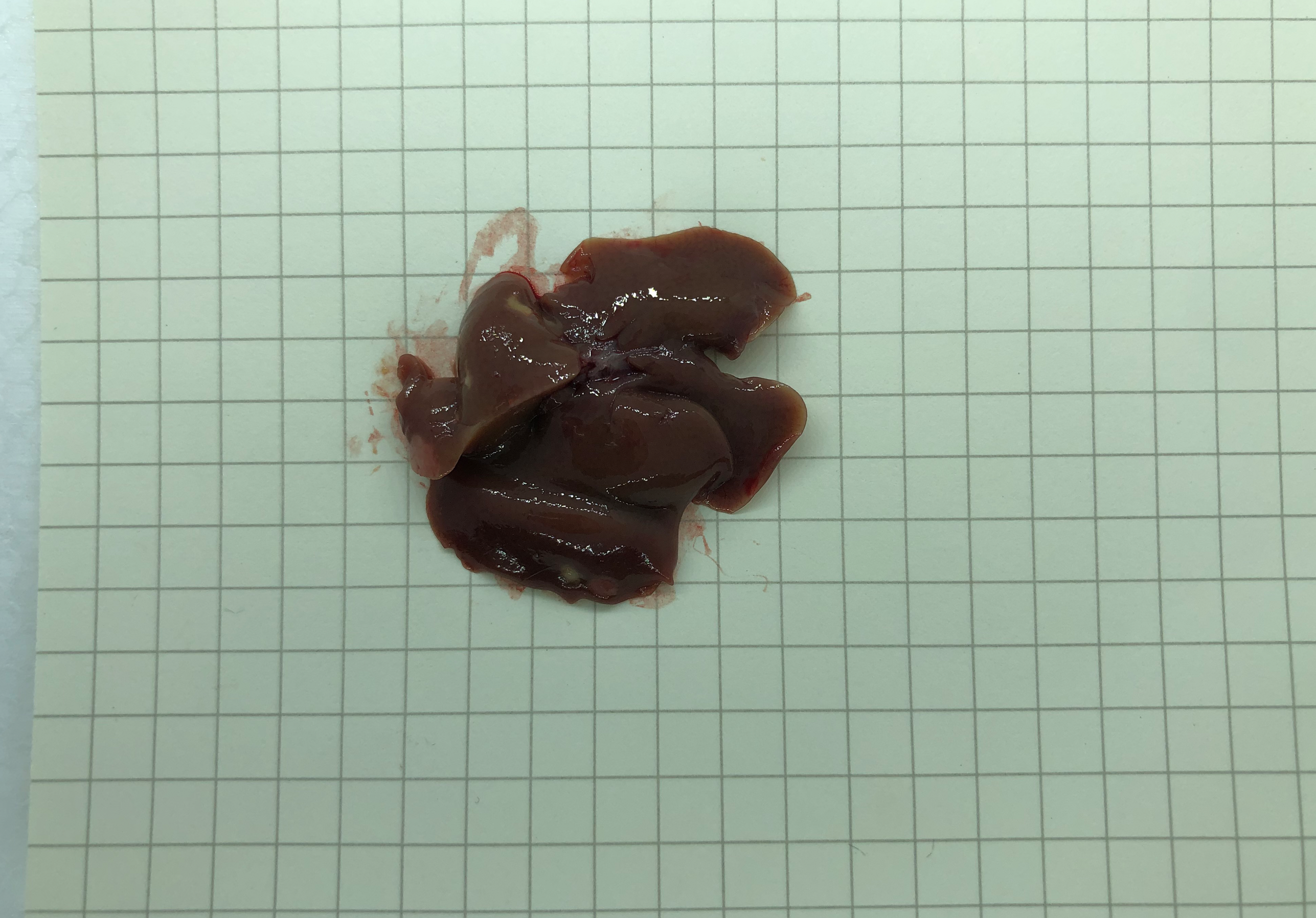

Supplement: Supplementary file 6 — Source Data for Figure 4 [file EMMM-15-e17230-s010.zip › Figure 4/4J/Liver image(DB-rAAV+Prednisone-2#).tif]

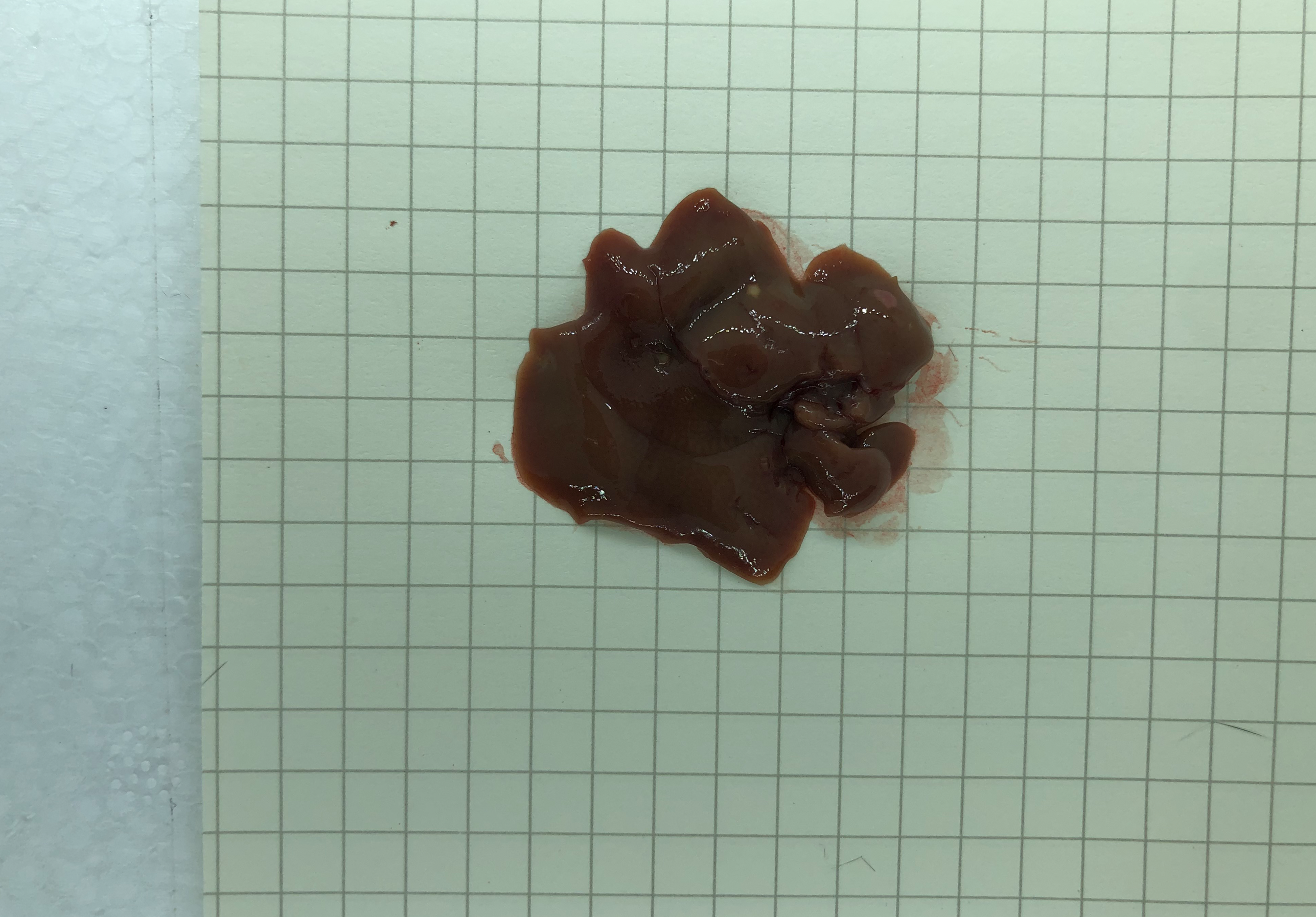

Supplement: Supplementary file 6 — Source Data for Figure 4 [file EMMM-15-e17230-s010.zip › Figure 4/4J/Liver image(DB-rAAV+Prednisone-3#).tif]

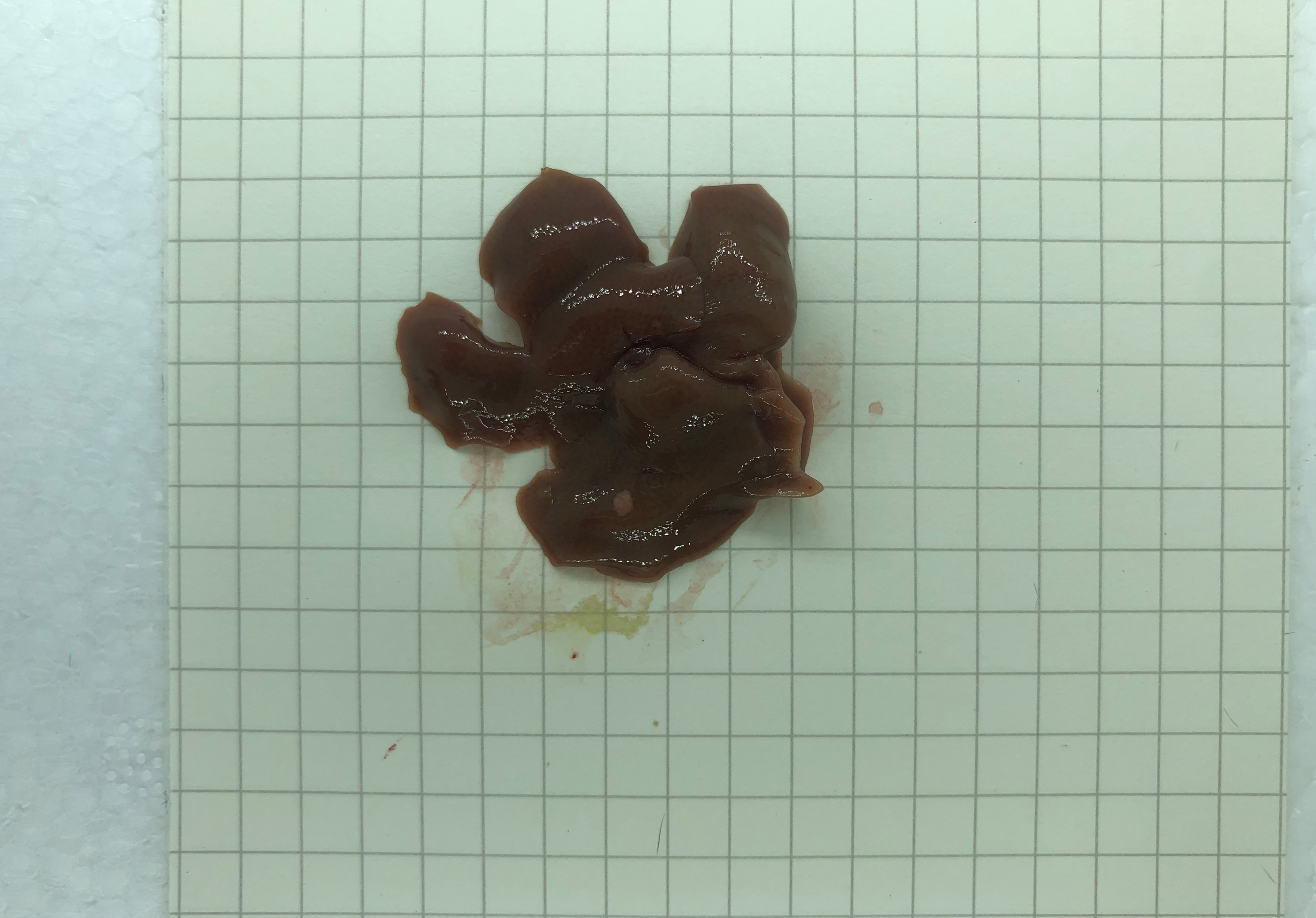

Supplement: Supplementary file 6 — Source Data for Figure 4 [file EMMM-15-e17230-s010.zip › Figure 4/4J/Liver image(DB-rAAV+Prednisone-4#).tif]

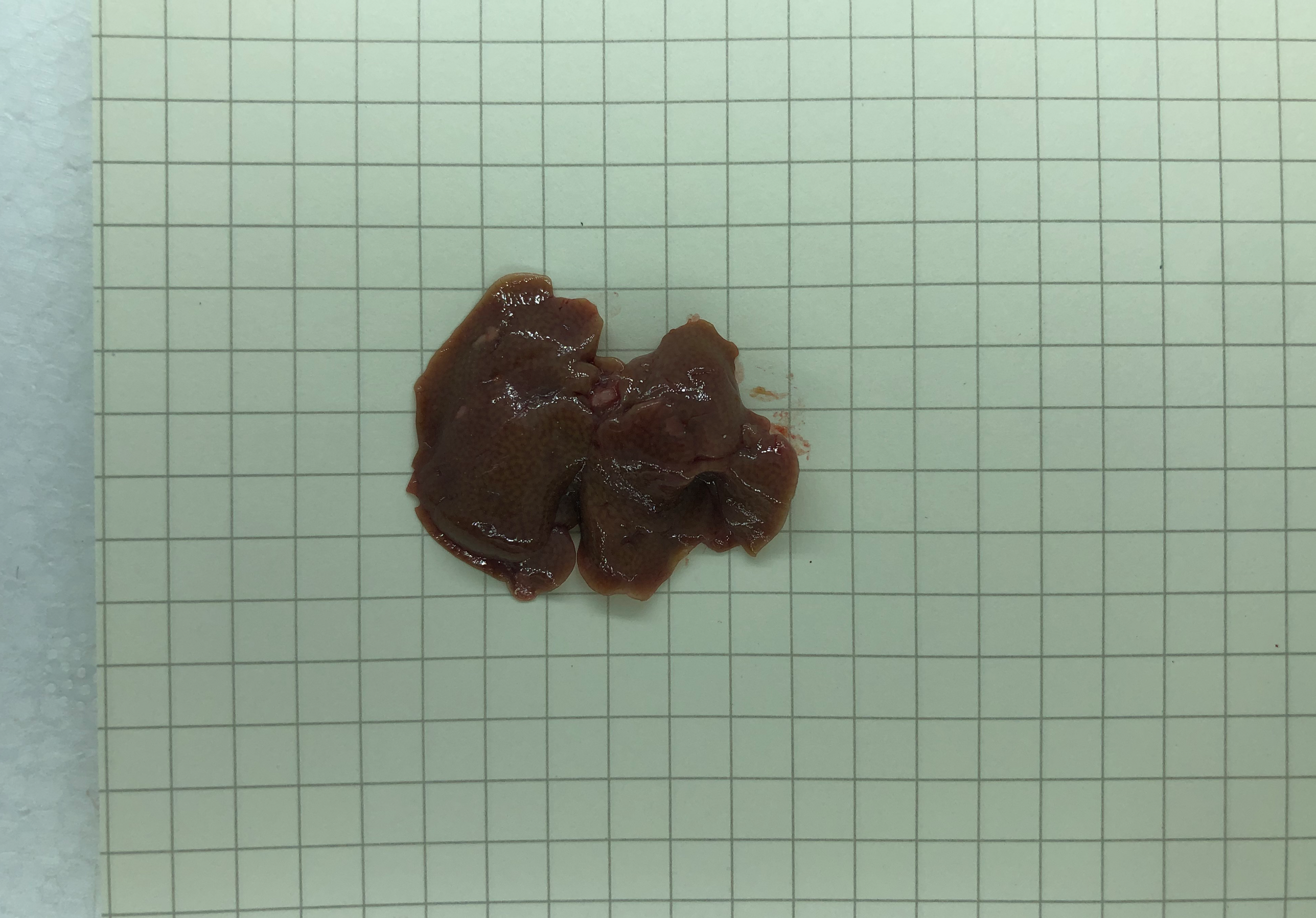

Supplement: Supplementary file 6 — Source Data for Figure 4 [file EMMM-15-e17230-s010.zip › Figure 4/4J/Liver image(DB-rAAV+Prednisone-5#).tif]

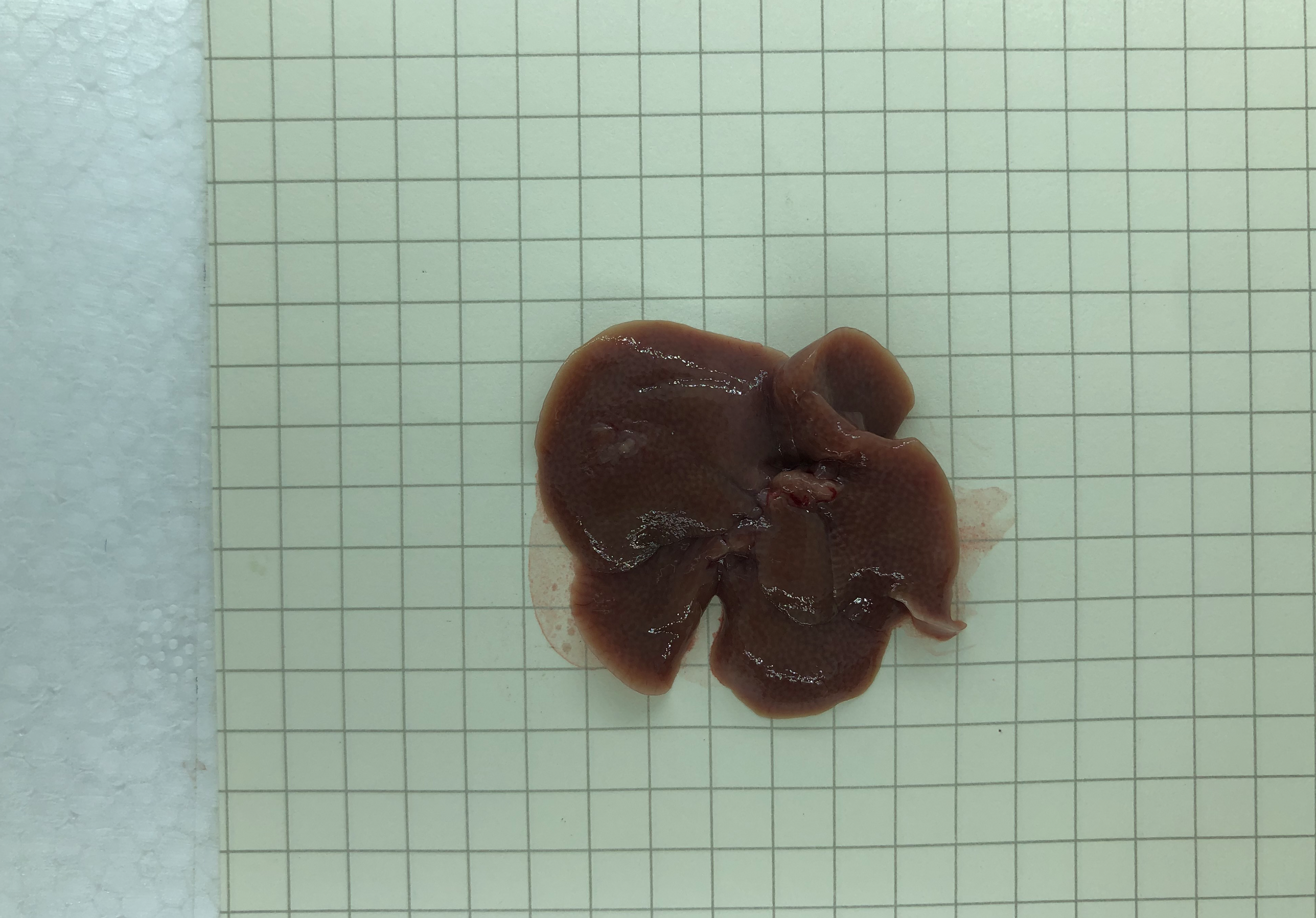

Supplement: Supplementary file 6 — Source Data for Figure 4 [file EMMM-15-e17230-s010.zip › Figure 4/4J/Liver image(DB-rAAV+Prednisone-6#).tif]

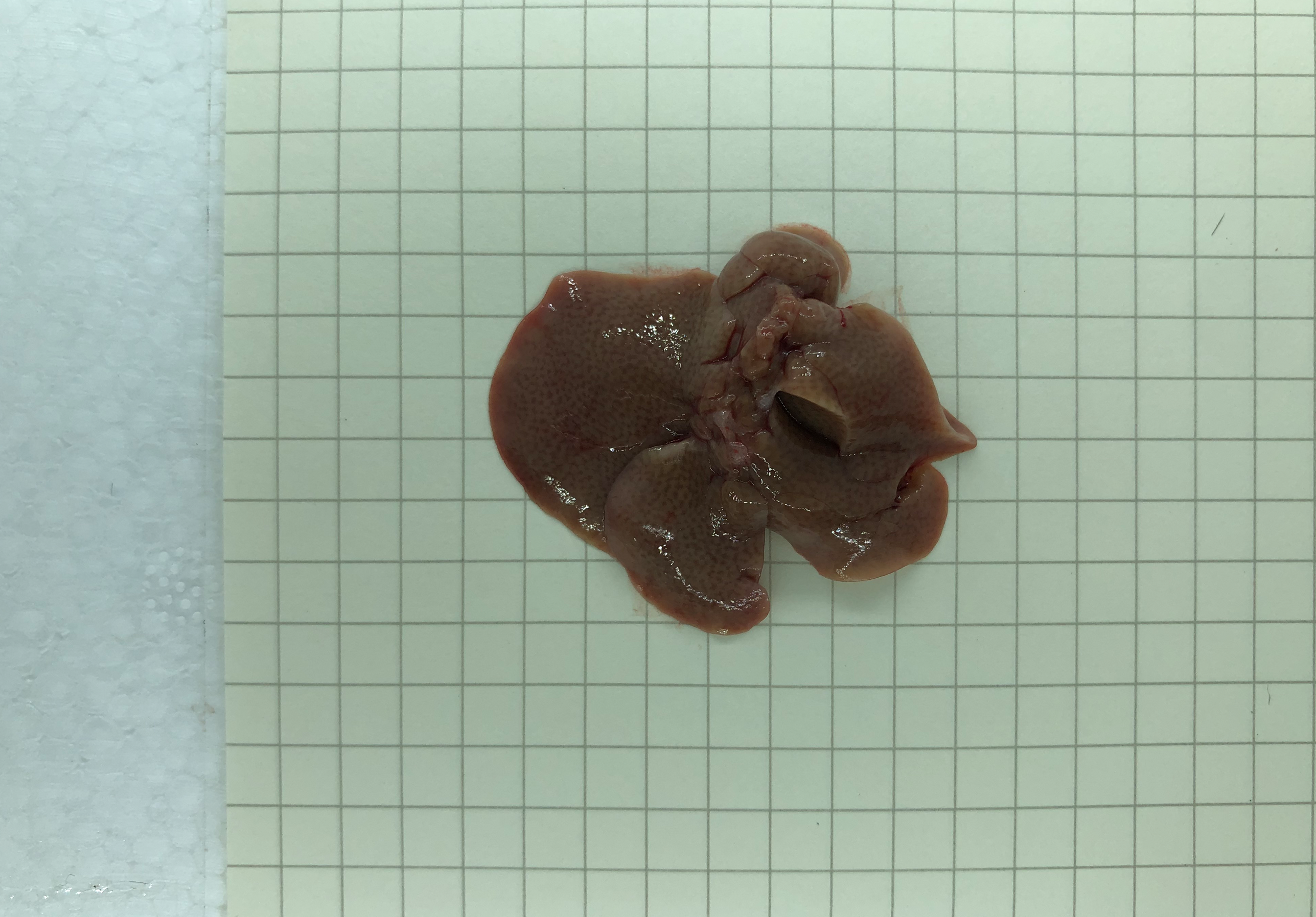

Supplement: Supplementary file 6 — Source Data for Figure 4 [file EMMM-15-e17230-s010.zip › Figure 4/4J/Liver image(DB-rAAV+Prednisone-7#).tif]

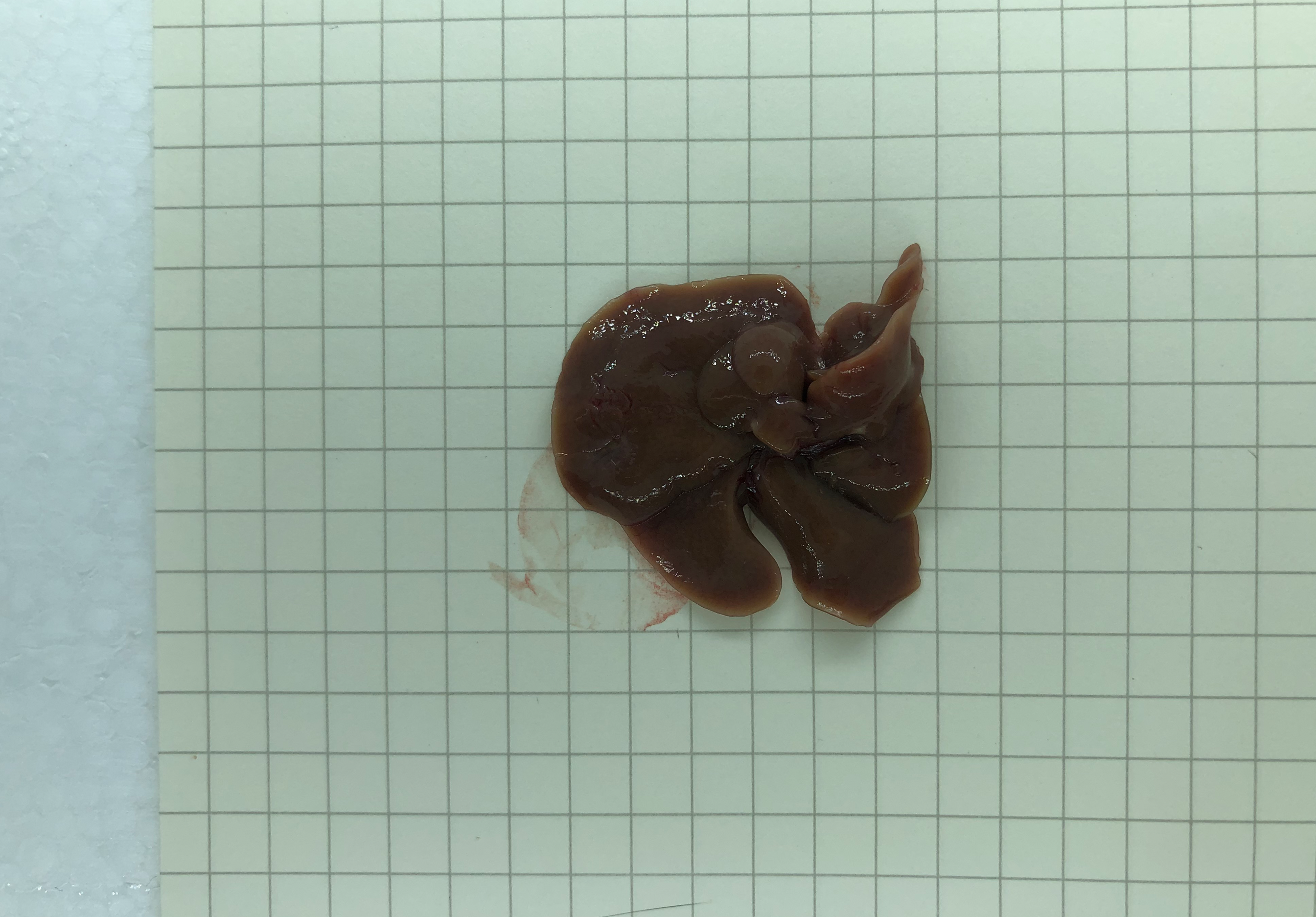

Supplement: Supplementary file 6 — Source Data for Figure 4 [file EMMM-15-e17230-s010.zip › Figure 4/4J/Liver image(DB-rAAV+Prednisone-8#).tif]

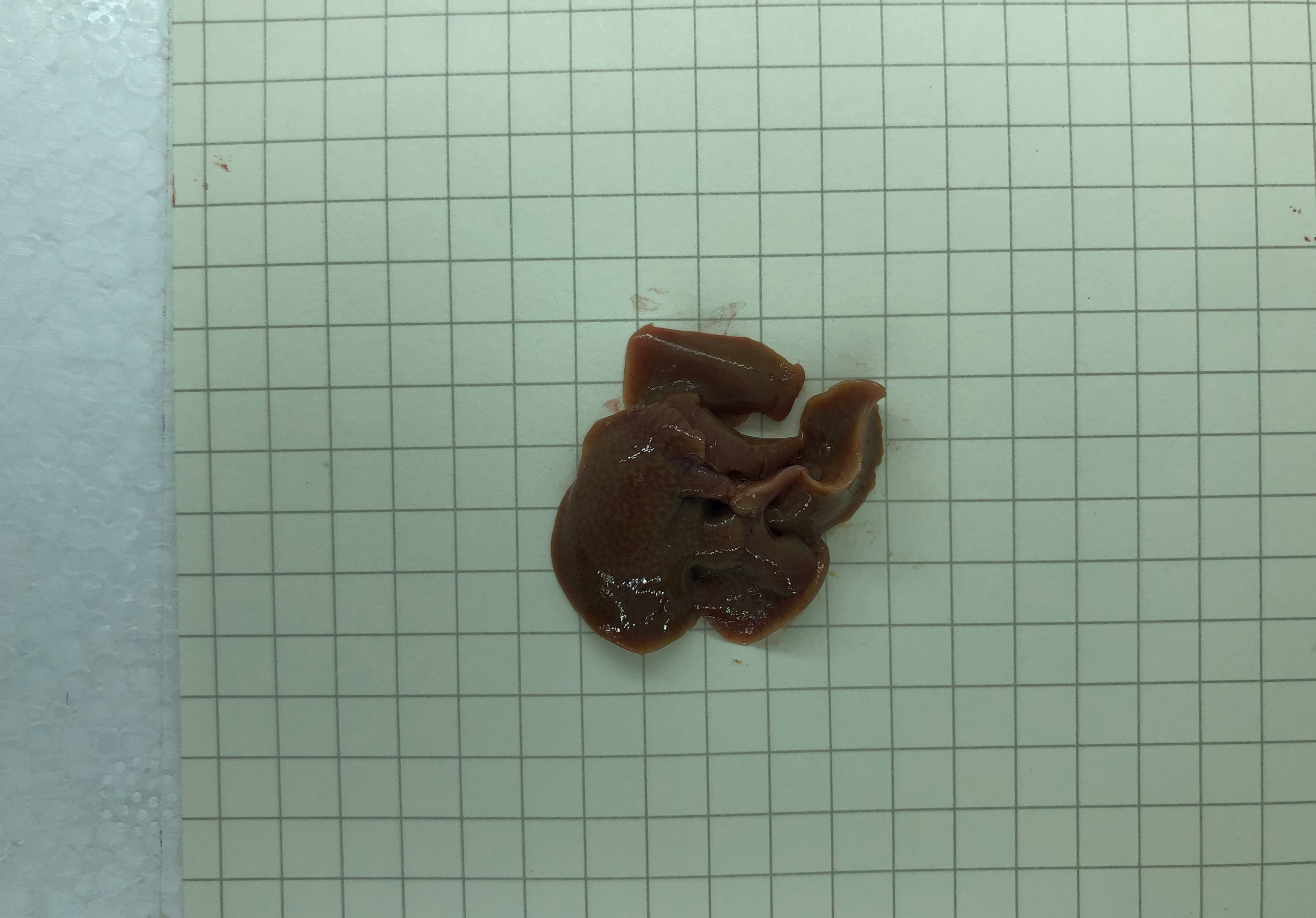

Supplement: Supplementary file 6 — Source Data for Figure 4 [file EMMM-15-e17230-s010.zip › Figure 4/4J/Liver image(DB-rAAV+Prednisone-9#).tif]

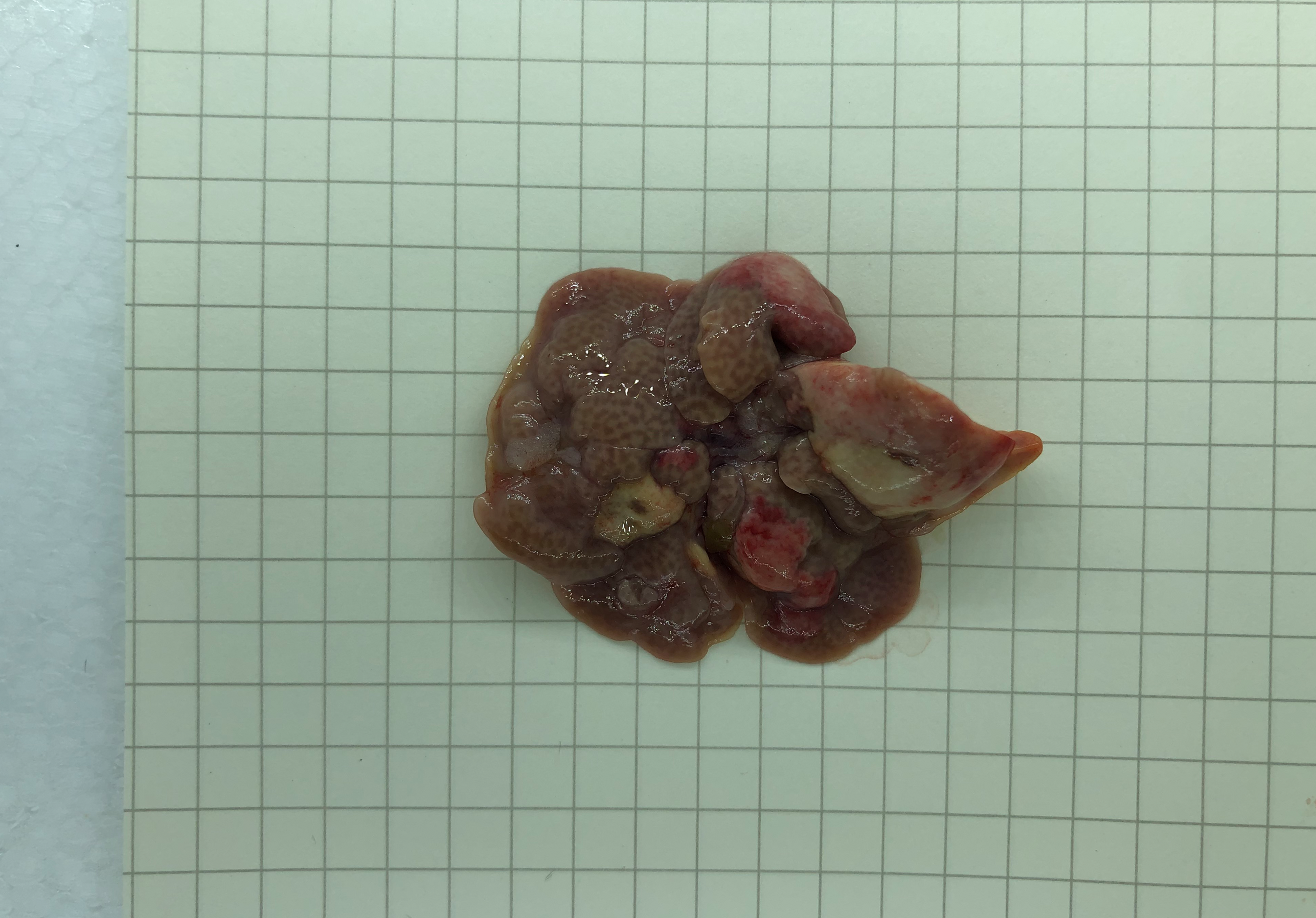

Supplement: Supplementary file 6 — Source Data for Figure 4 [file EMMM-15-e17230-s010.zip › Figure 4/4J/Liver image(DB-rAAV-1#).tif]

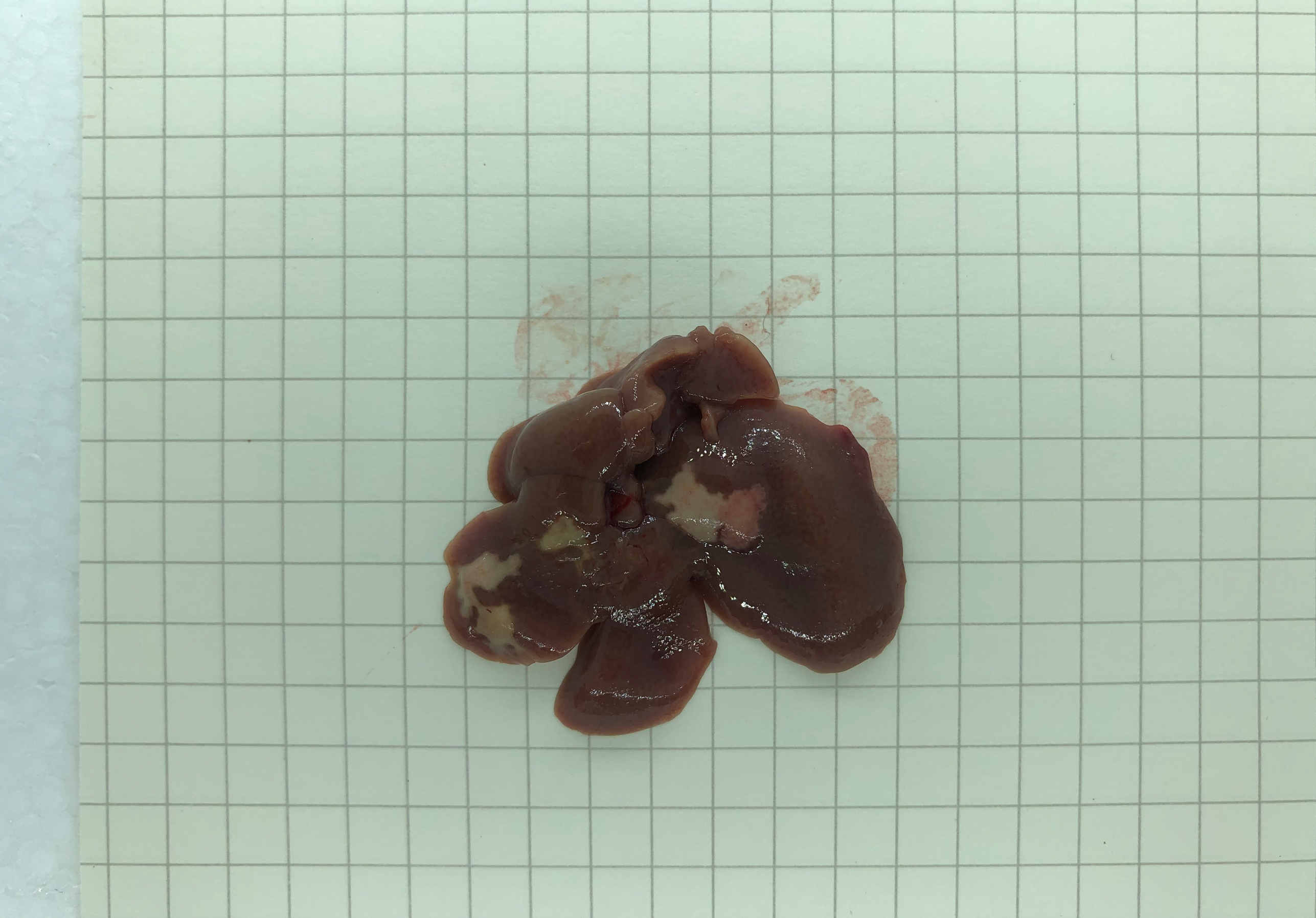

Supplement: Supplementary file 6 — Source Data for Figure 4 [file EMMM-15-e17230-s010.zip › Figure 4/4J/Liver image(DB-rAAV-10#).tif]

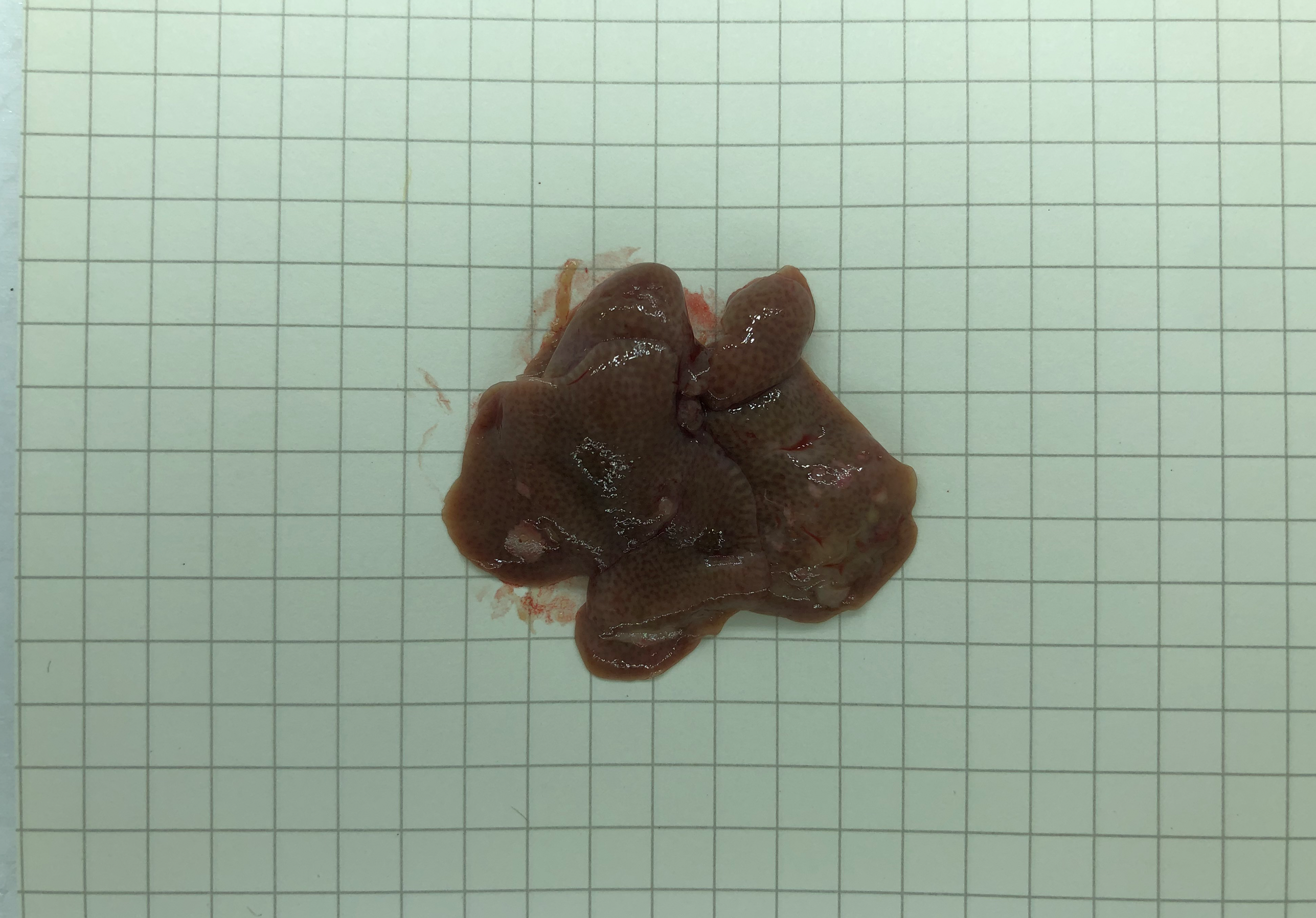

Supplement: Supplementary file 6 — Source Data for Figure 4 [file EMMM-15-e17230-s010.zip › Figure 4/4J/Liver image(DB-rAAV-11#).tif]

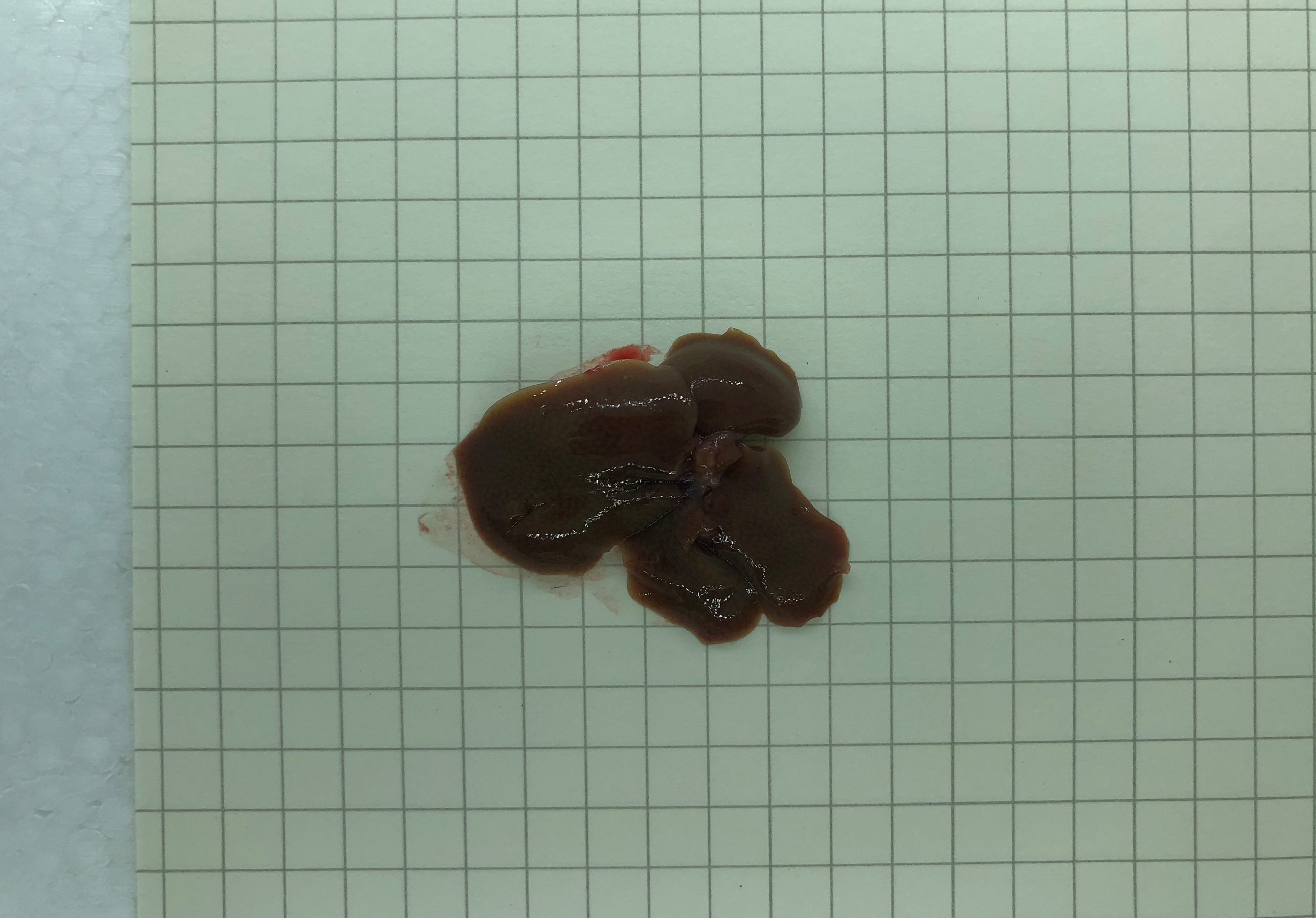

Supplement: Supplementary file 6 — Source Data for Figure 4 [file EMMM-15-e17230-s010.zip › Figure 4/4J/Liver image(DB-rAAV-12#).tif]

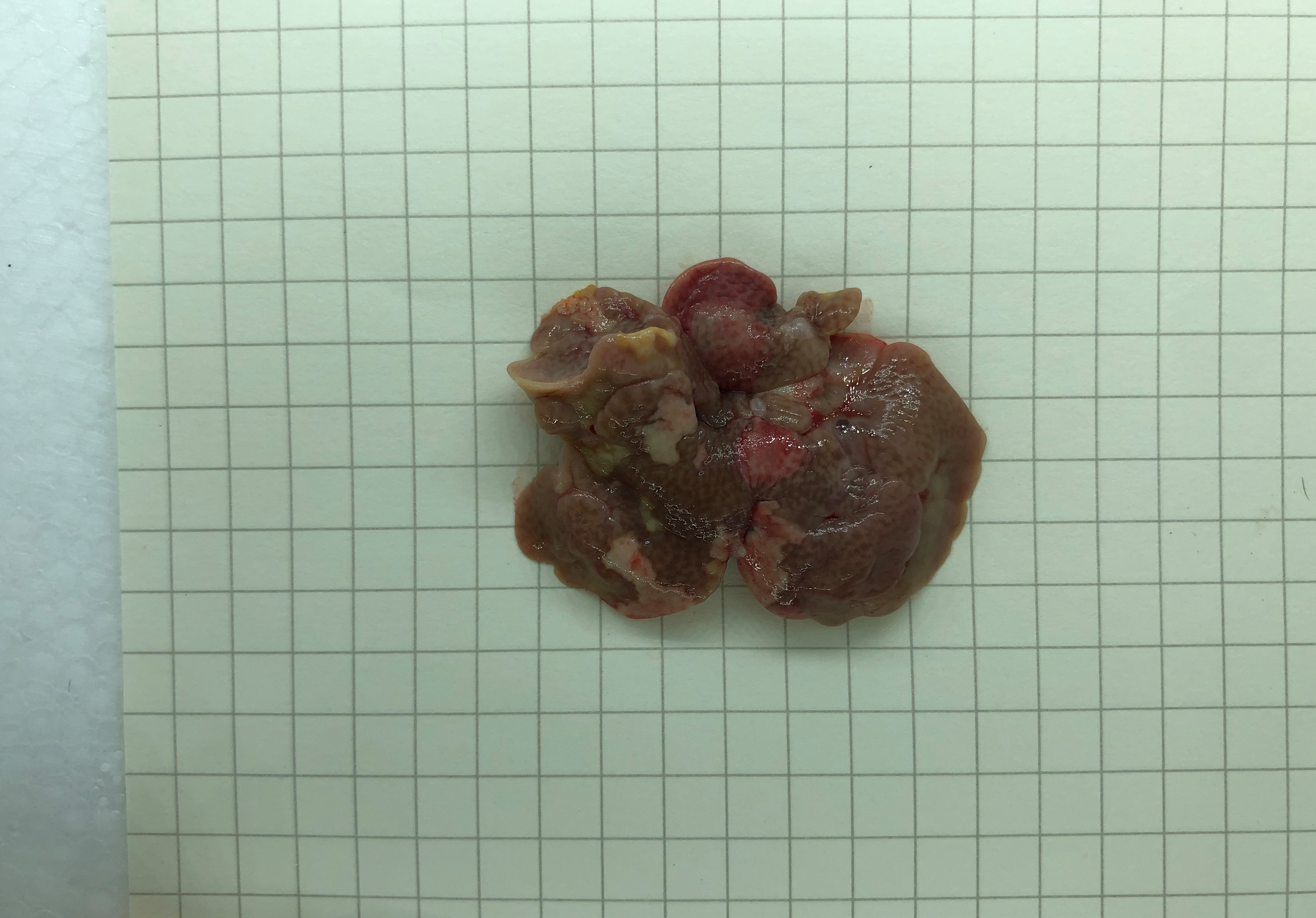

Supplement: Supplementary file 6 — Source Data for Figure 4 [file EMMM-15-e17230-s010.zip › Figure 4/4J/Liver image(DB-rAAV-2#).tif]

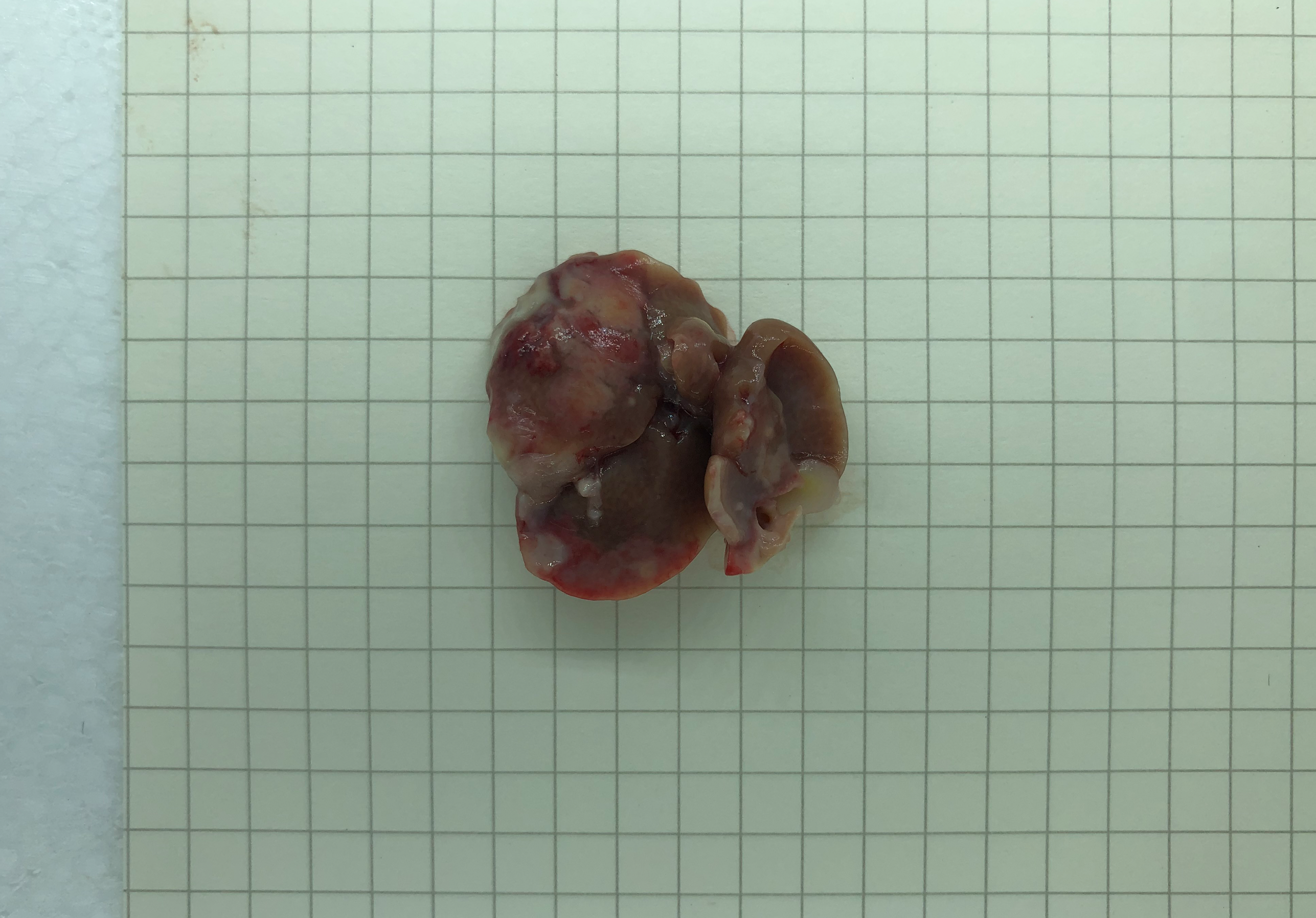

Supplement: Supplementary file 6 — Source Data for Figure 4 [file EMMM-15-e17230-s010.zip › Figure 4/4J/Liver image(DB-rAAV-3#).tif]

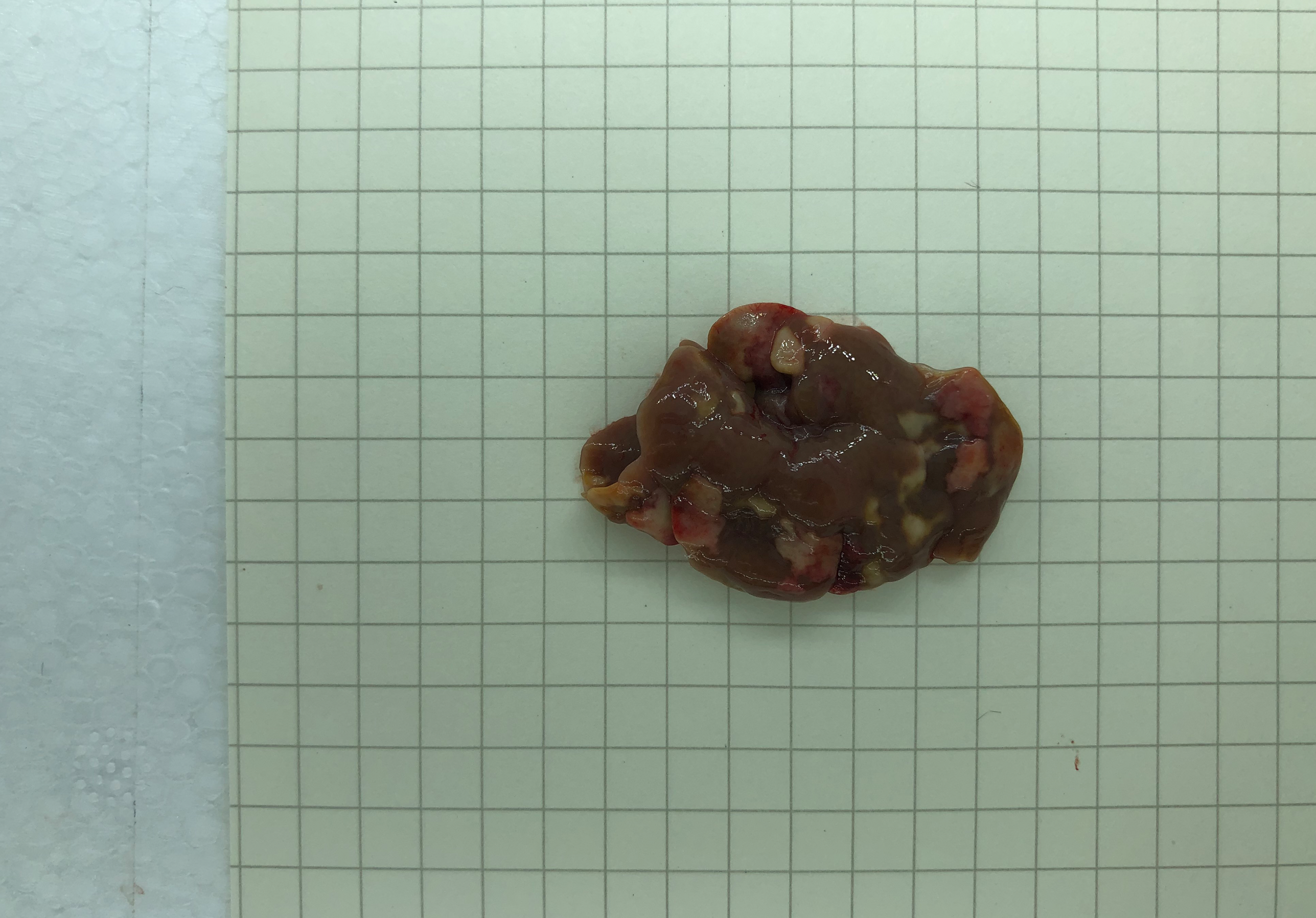

Supplement: Supplementary file 6 — Source Data for Figure 4 [file EMMM-15-e17230-s010.zip › Figure 4/4J/Liver image(DB-rAAV-4#).tif]

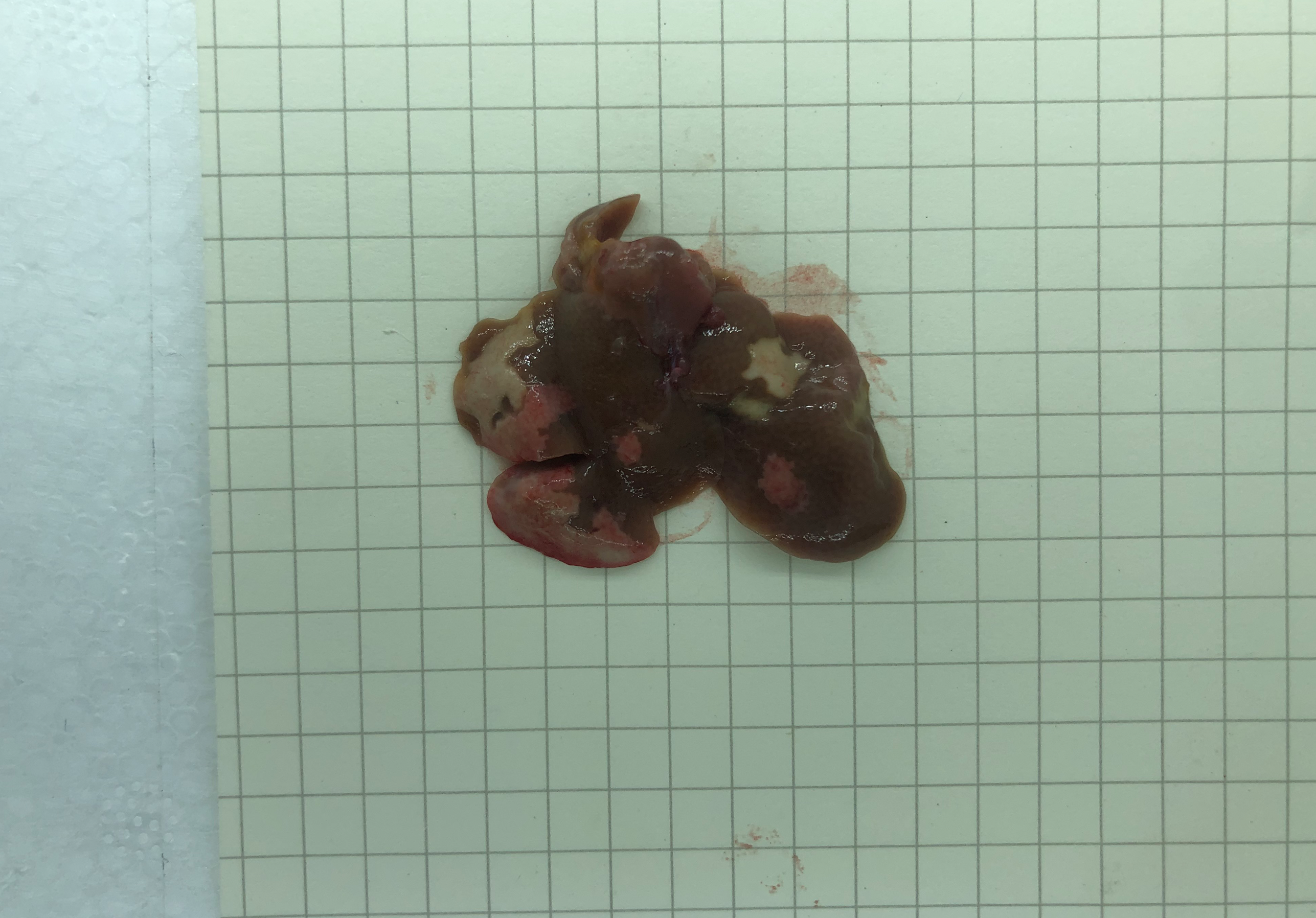

Supplement: Supplementary file 6 — Source Data for Figure 4 [file EMMM-15-e17230-s010.zip › Figure 4/4J/Liver image(DB-rAAV-5#).tif]

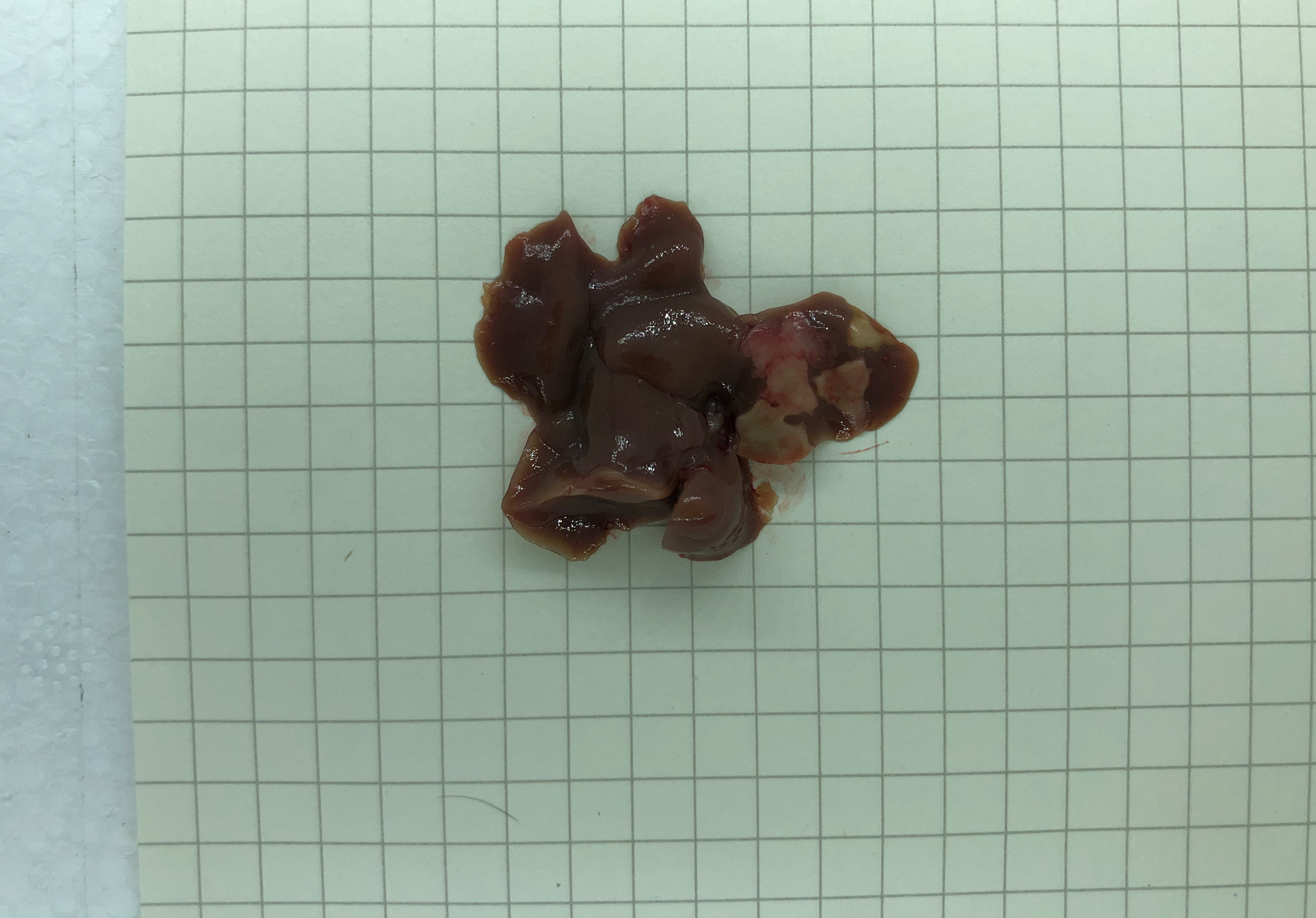

Supplement: Supplementary file 6 — Source Data for Figure 4 [file EMMM-15-e17230-s010.zip › Figure 4/4J/Liver image(DB-rAAV-6#).tif]

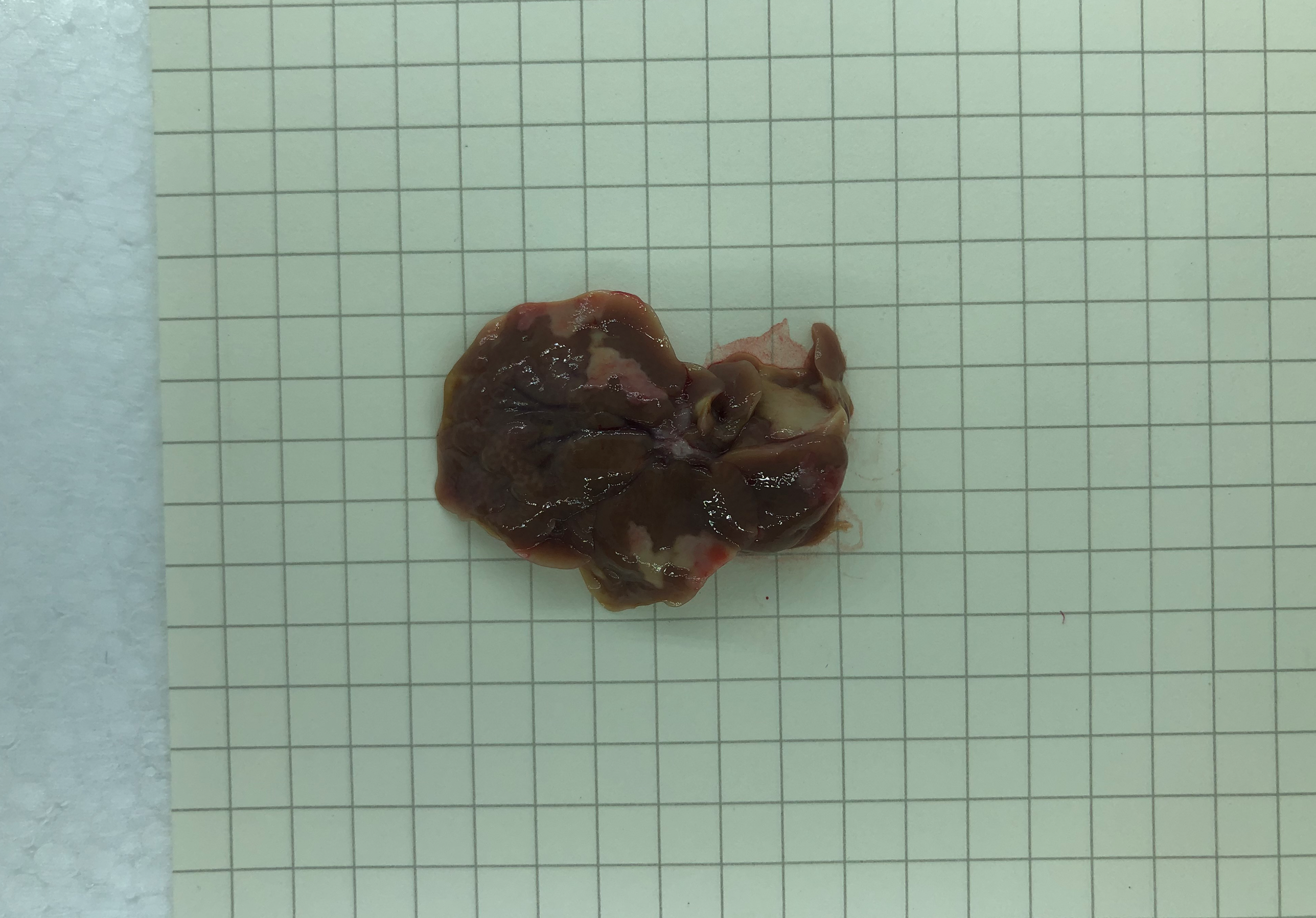

Supplement: Supplementary file 6 — Source Data for Figure 4 [file EMMM-15-e17230-s010.zip › Figure 4/4J/Liver image(DB-rAAV-7#).tif]

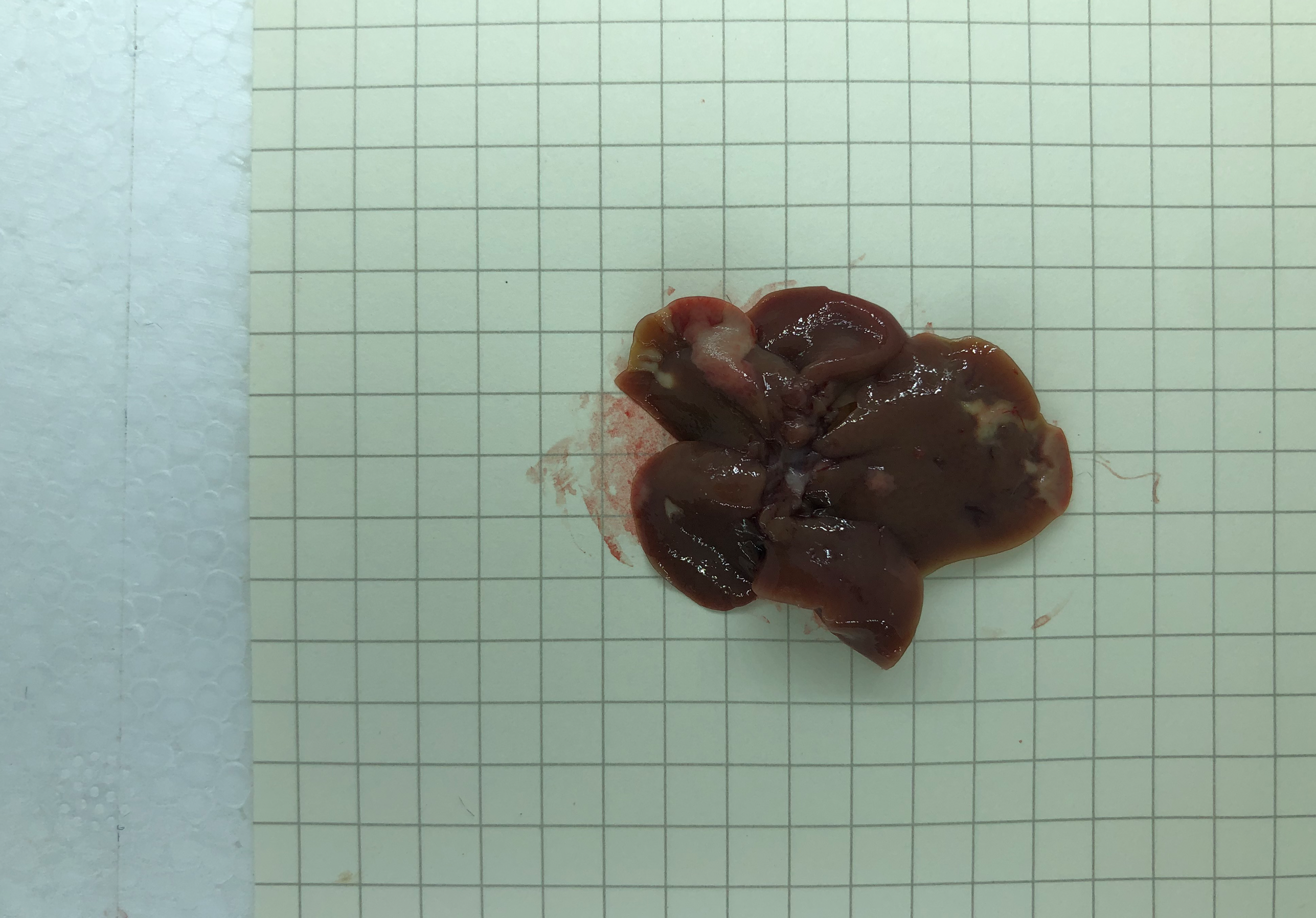

Supplement: Supplementary file 6 — Source Data for Figure 4 [file EMMM-15-e17230-s010.zip › Figure 4/4J/Liver image(DB-rAAV-8#).tif]

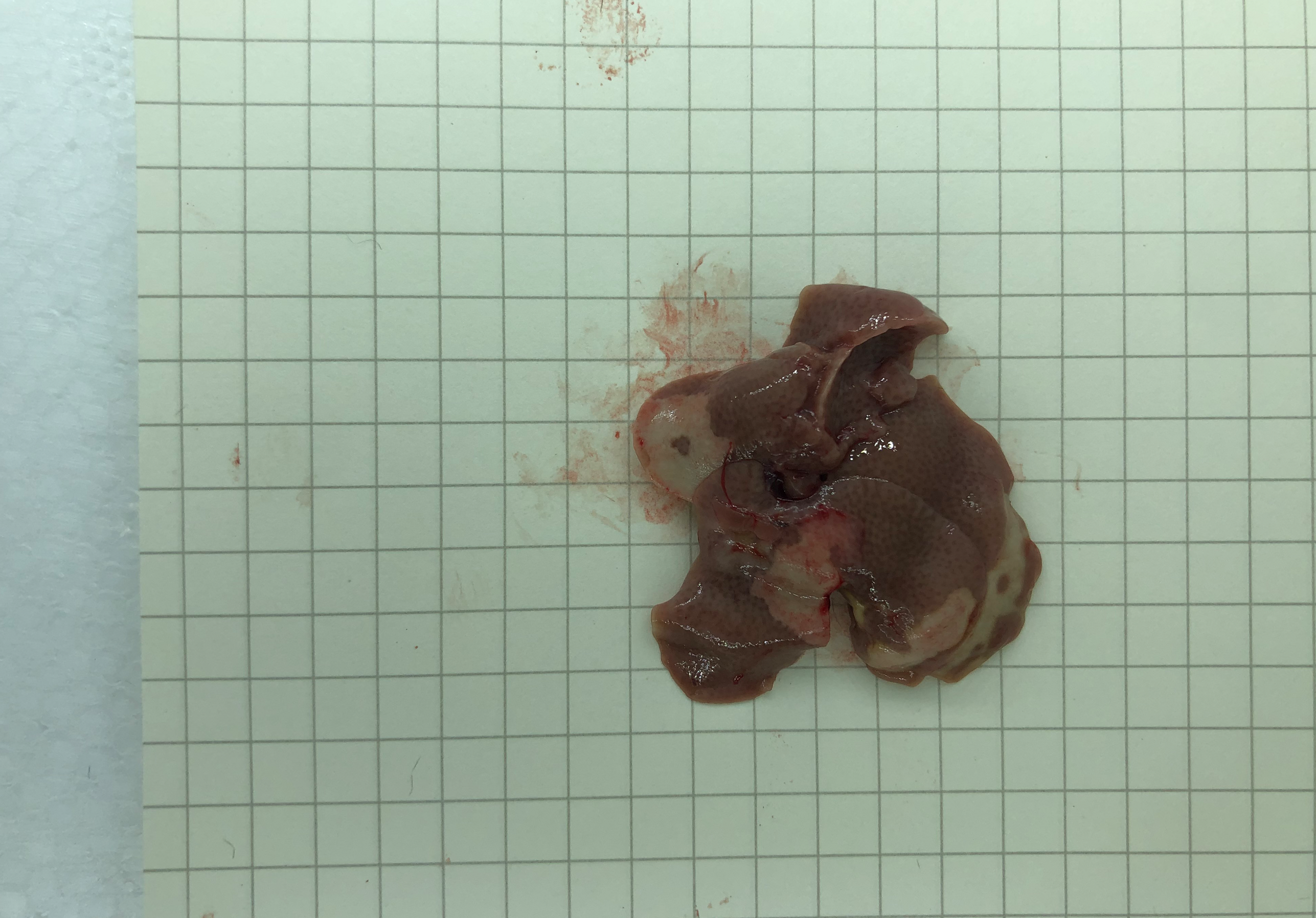

Supplement: Supplementary file 6 — Source Data for Figure 4 [file EMMM-15-e17230-s010.zip › Figure 4/4J/Liver image(DB-rAAV-9#).tif]

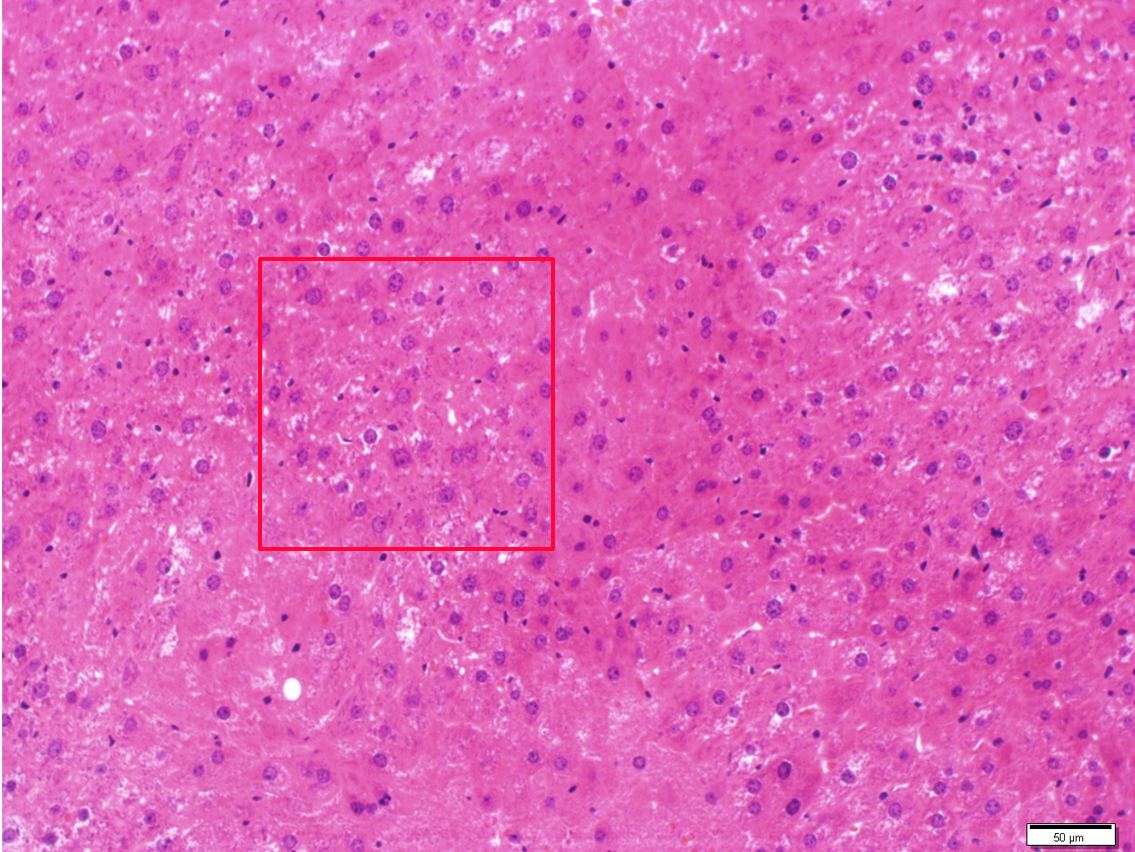

Supplement: Supplementary file 6 — Source Data for Figure 4 [file EMMM-15-e17230-s010.zip › Figure 4/4L/H&E for DB-rAAV+Prednisone-1-2M.tif]

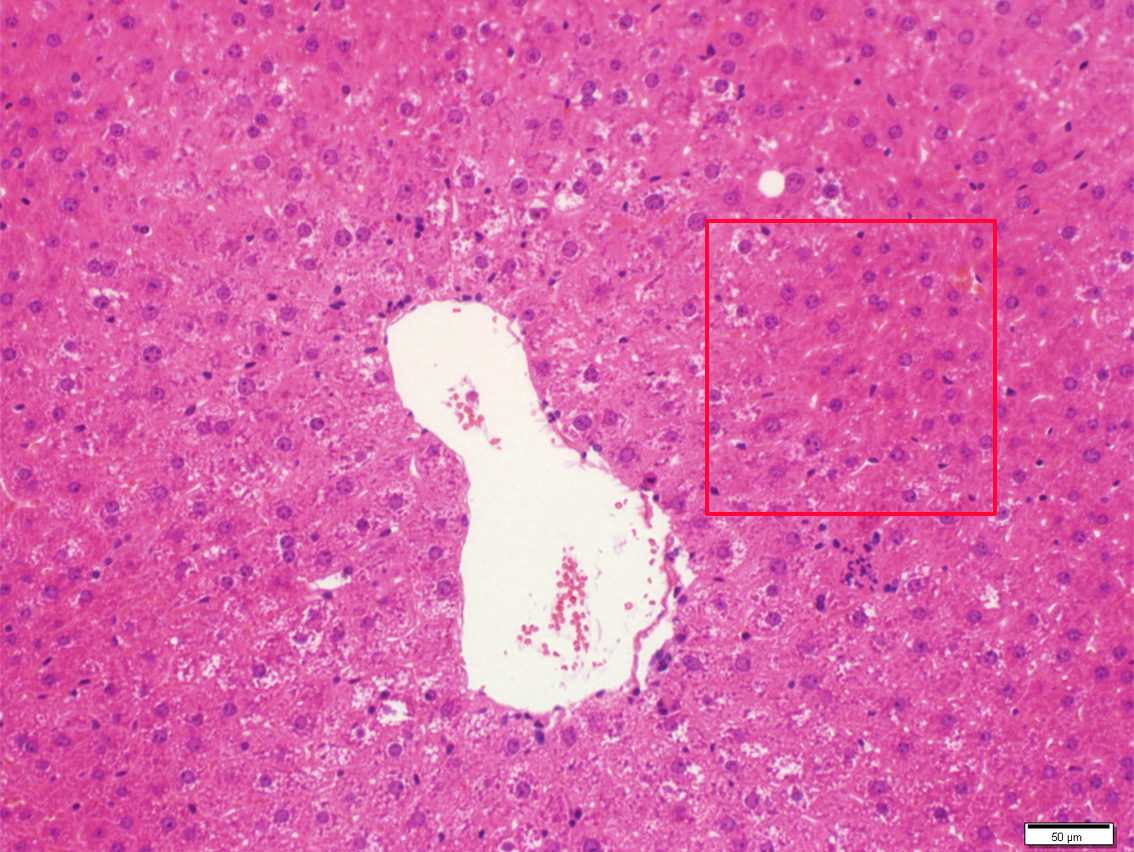

Supplement: Supplementary file 6 — Source Data for Figure 4 [file EMMM-15-e17230-s010.zip › Figure 4/4L/H&E for DB-rAAV+Prednisone-2-2M.tif]

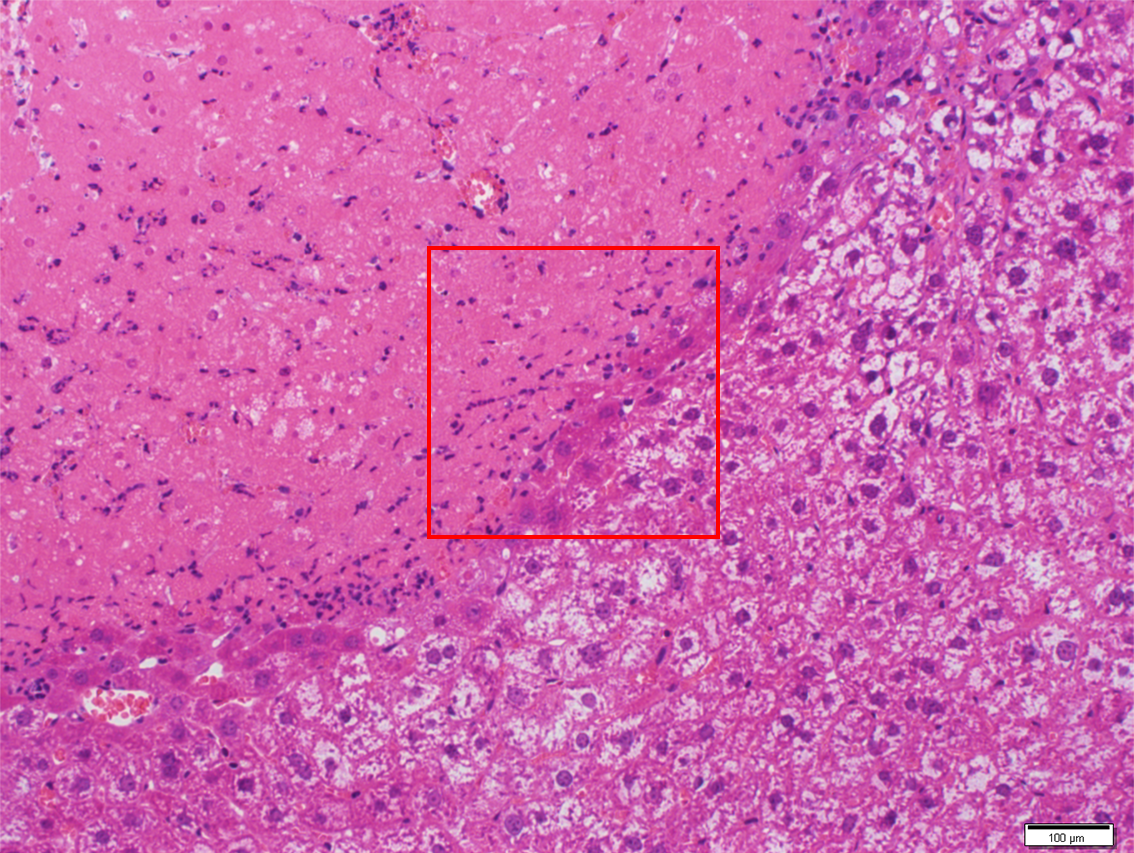

Supplement: Supplementary file 6 — Source Data for Figure 4 [file EMMM-15-e17230-s010.zip › Figure 4/4L/H&E for DB-rAAV-1-2M.tif]

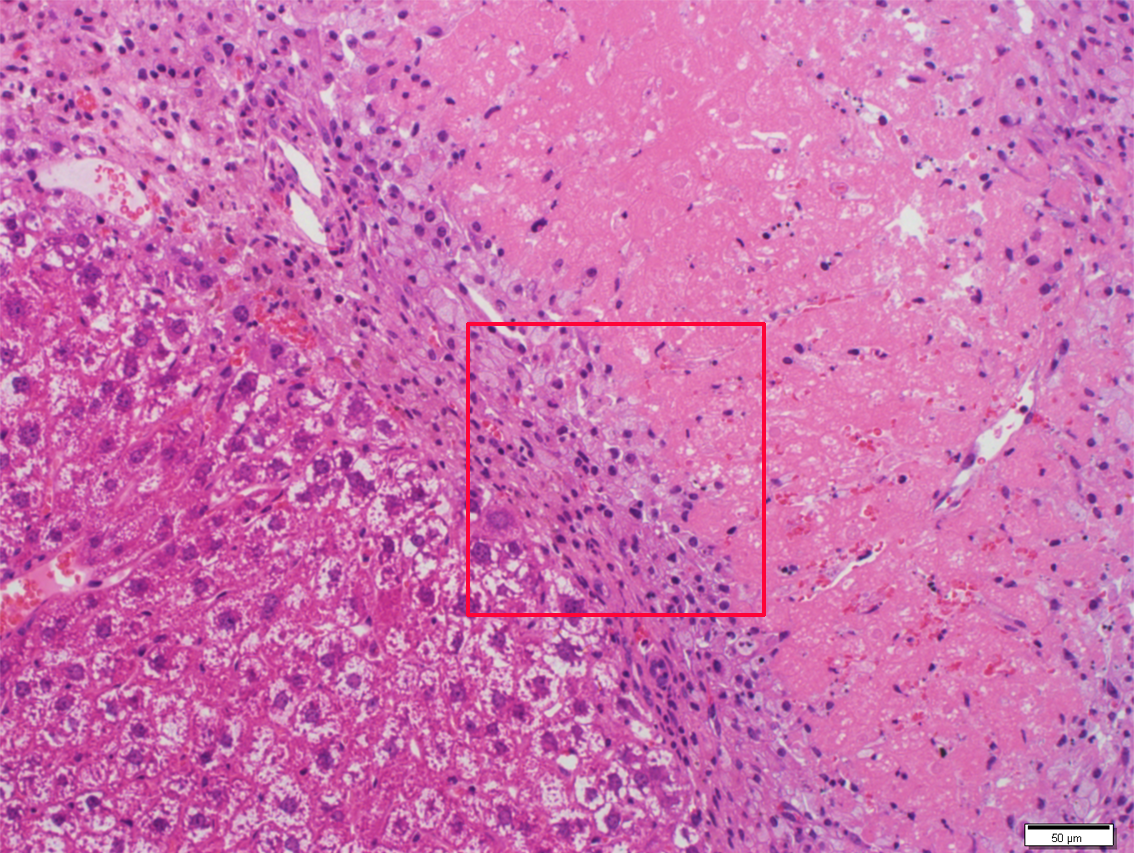

Supplement: Supplementary file 6 — Source Data for Figure 4 [file EMMM-15-e17230-s010.zip › Figure 4/4L/H&E for DB-rAAV-2-2M.tif]

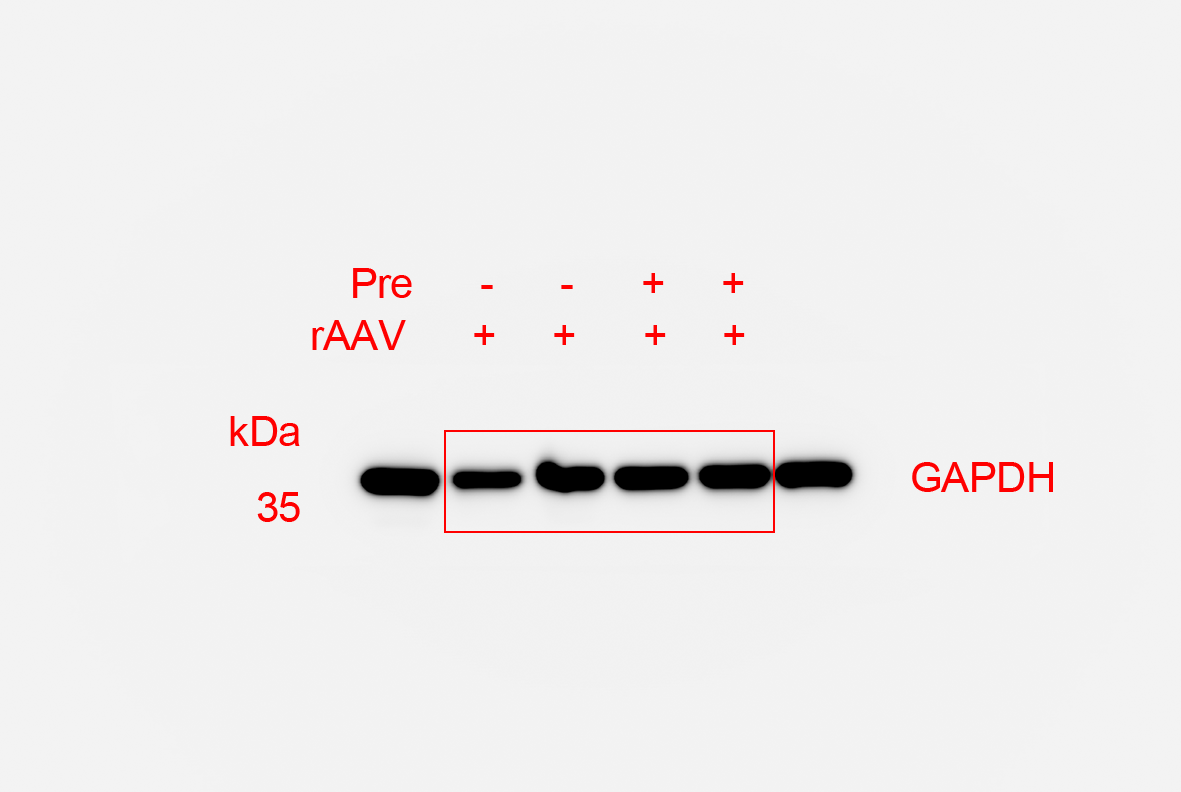

Supplement: Supplementary file 6 — Source Data for Figure 4 [file EMMM-15-e17230-s010.zip › Figure 4/4M/Western/GAPDH.tif]

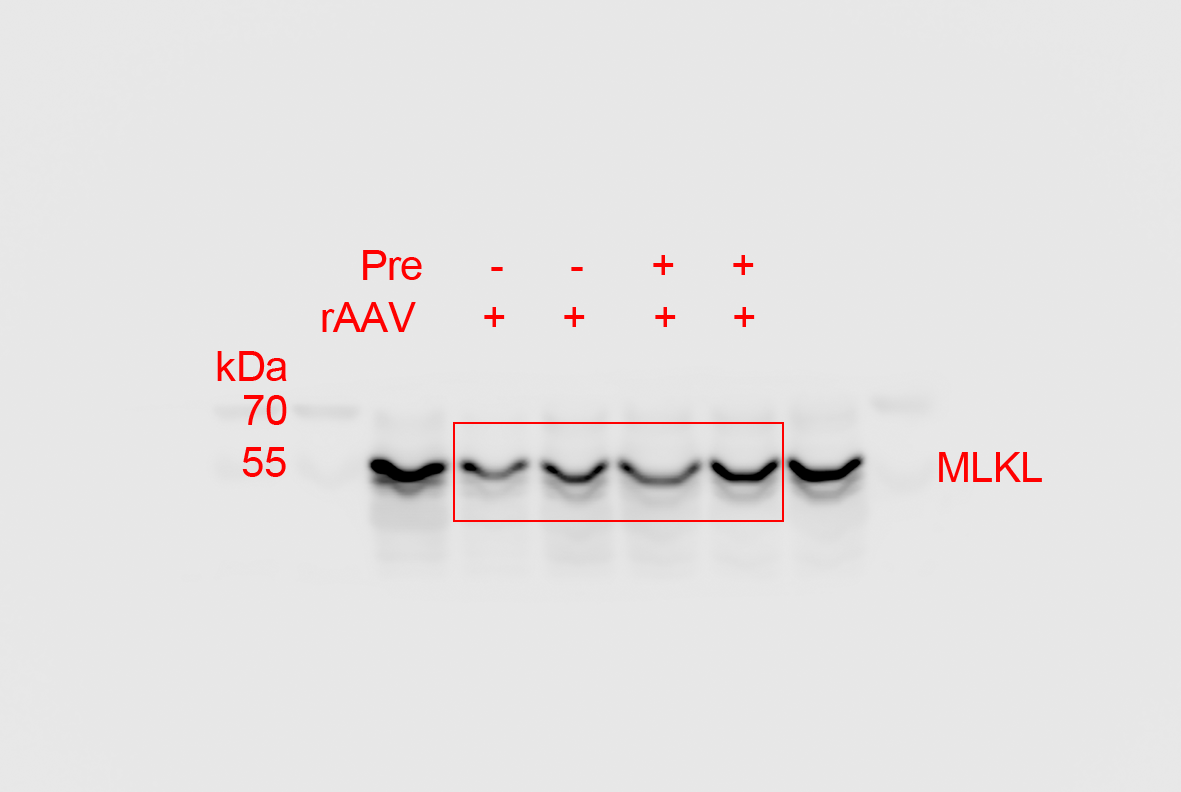

Supplement: Supplementary file 6 — Source Data for Figure 4 [file EMMM-15-e17230-s010.zip › Figure 4/4M/Western/MLKL.tif]

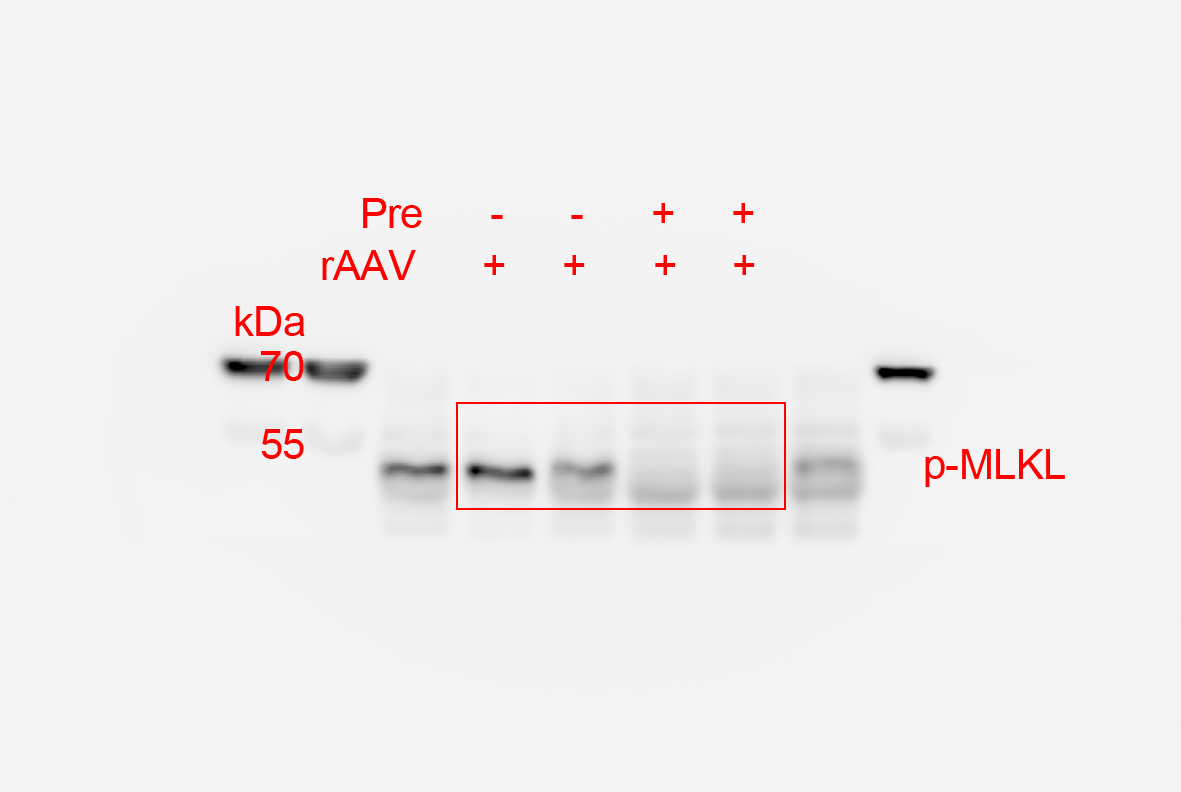

Supplement: Supplementary file 6 — Source Data for Figure 4 [file EMMM-15-e17230-s010.zip › Figure 4/4M/Western/p-MLKL.tif]

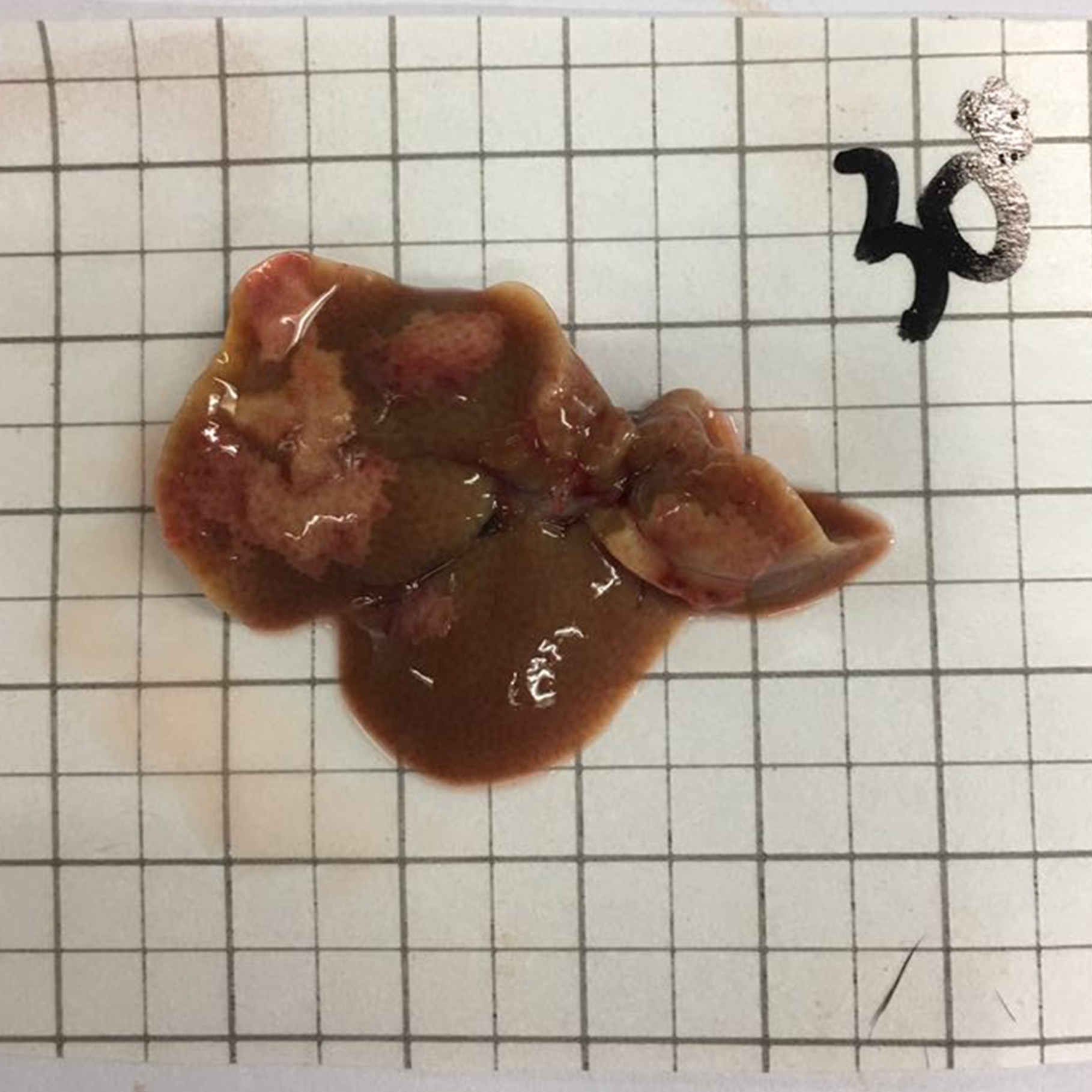

Supplement: Supplementary file 7 — Source Data for Figure 5 [file EMMM-15-e17230-s002.zip › Figure 5/5I/DB-rAAV-si-Cyp4a14-Liver.tif]

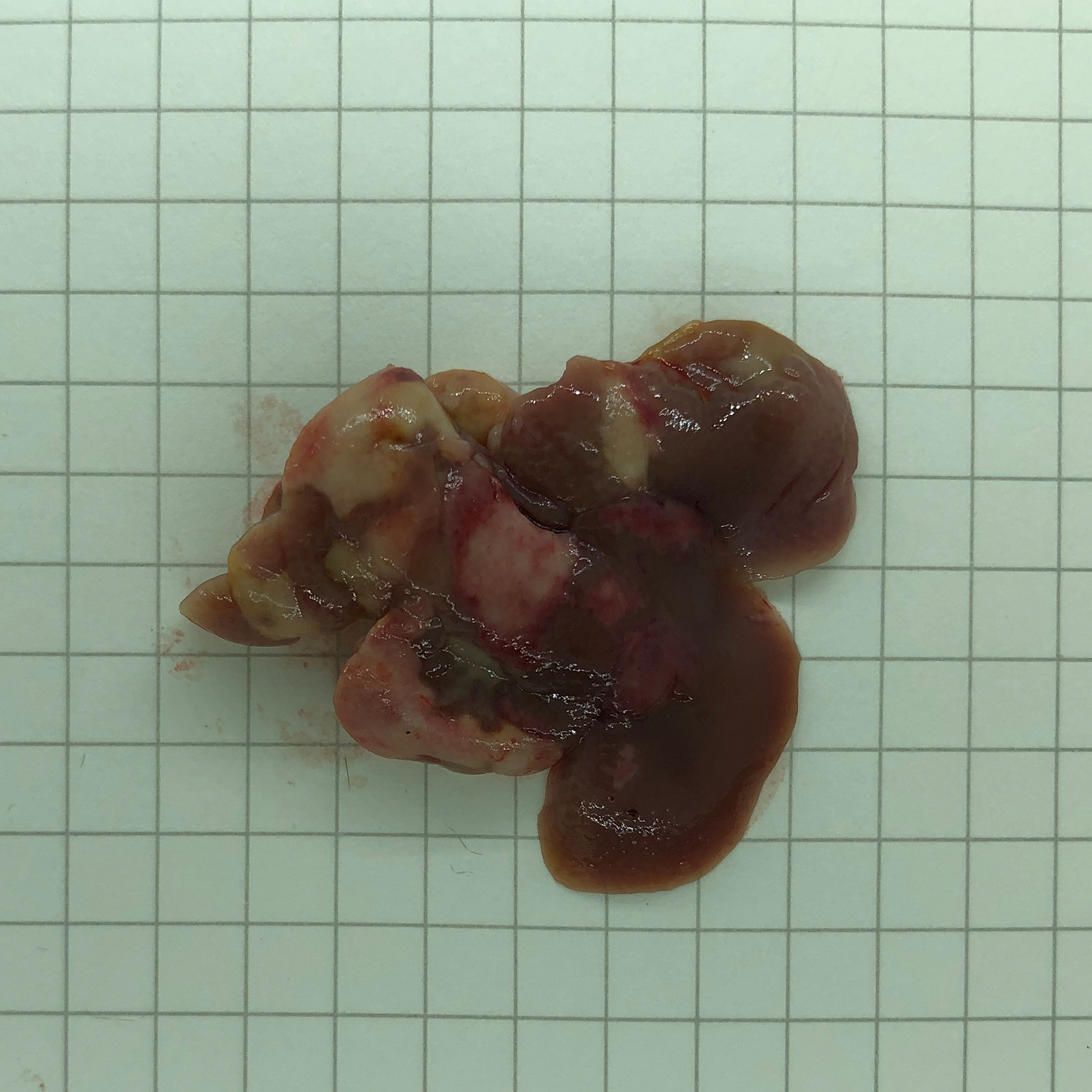

Supplement: Supplementary file 7 — Source Data for Figure 5 [file EMMM-15-e17230-s002.zip › Figure 5/5I/DB-rAAV-si-NC-Liver.tif]

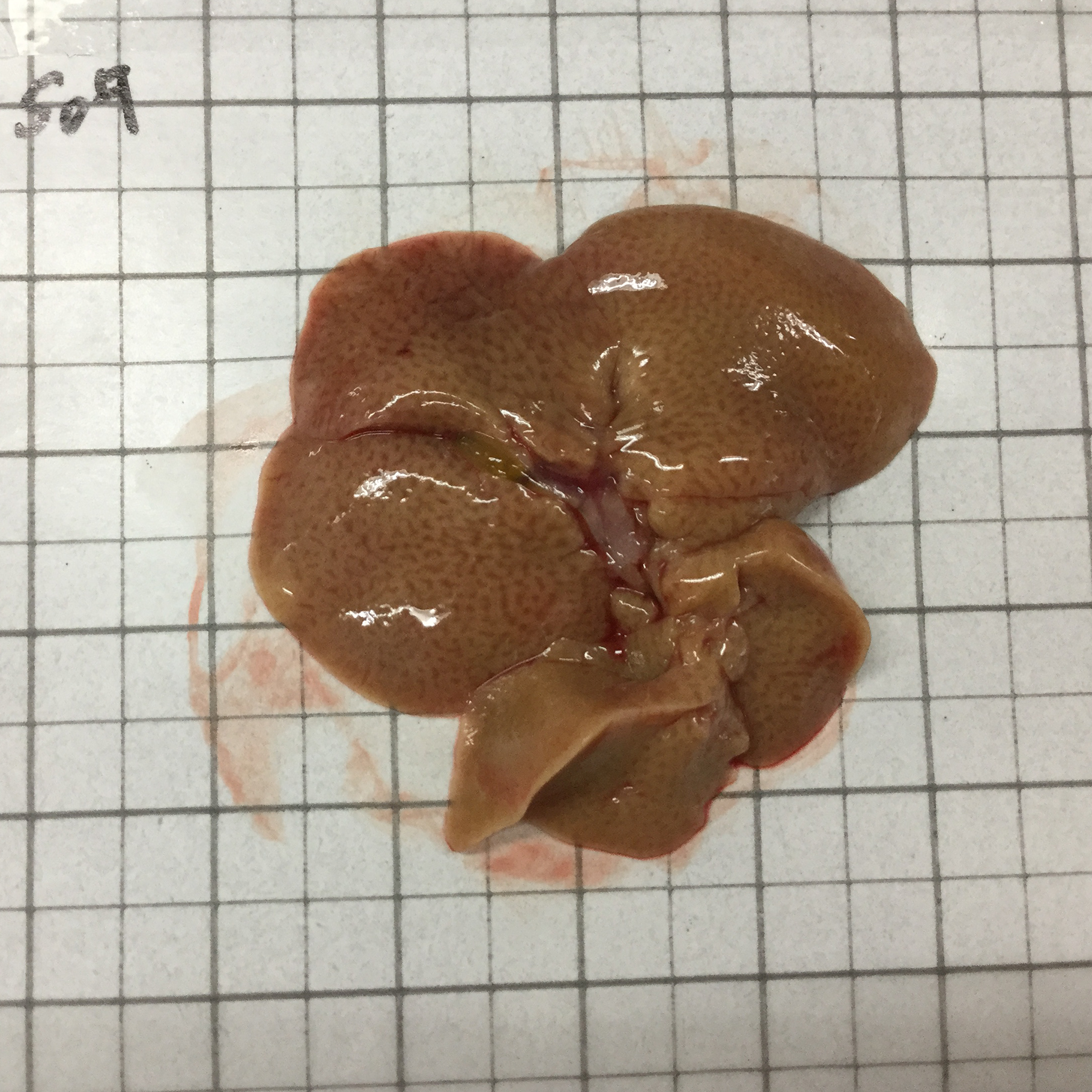

Supplement: Supplementary file 7 — Source Data for Figure 5 [file EMMM-15-e17230-s002.zip › Figure 5/5I/DB-rAAV-si-Pebp1-Liver.tif]

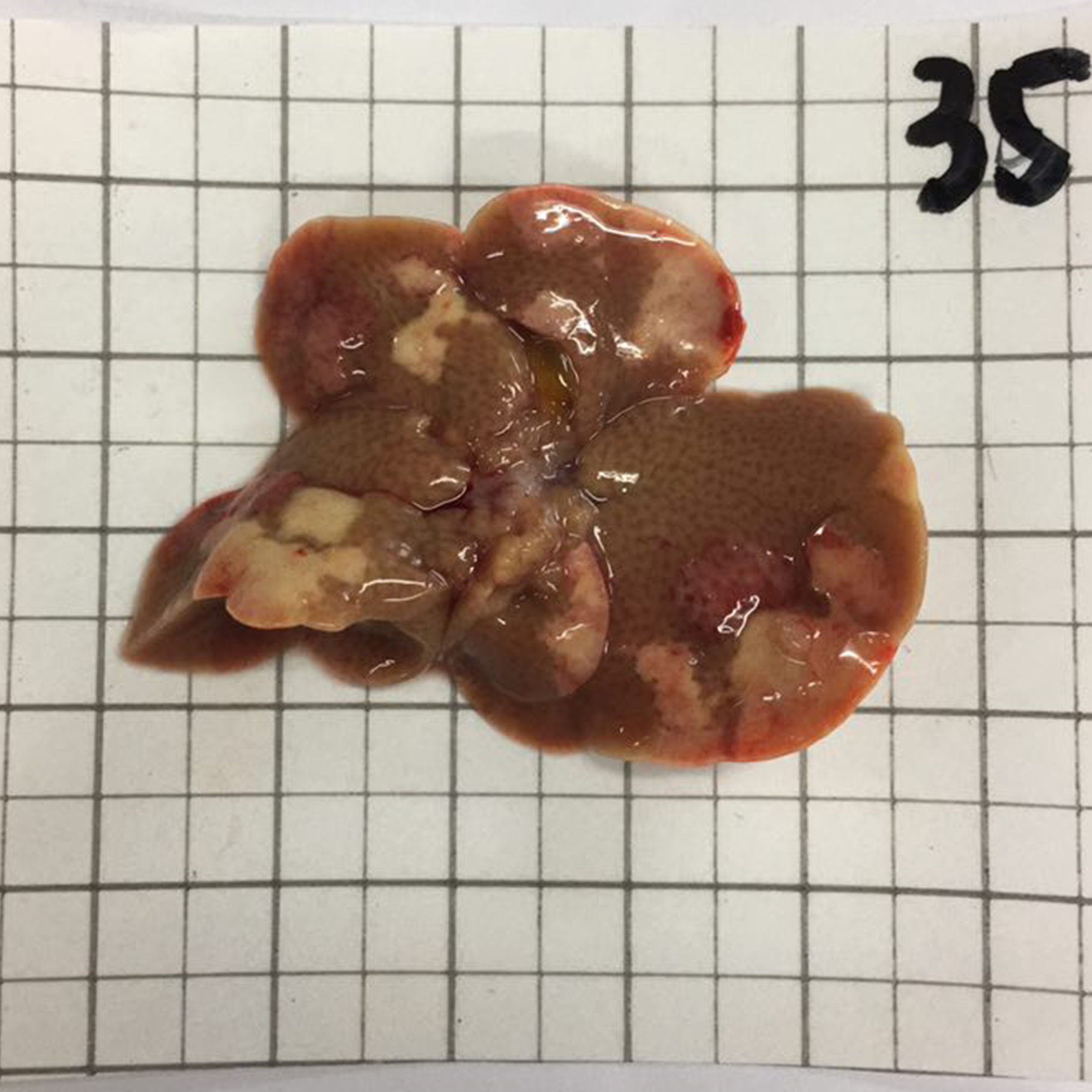

Supplement: Supplementary file 7 — Source Data for Figure 5 [file EMMM-15-e17230-s002.zip › Figure 5/5I/DB-rAAV-si-Tat-Liver.tif]

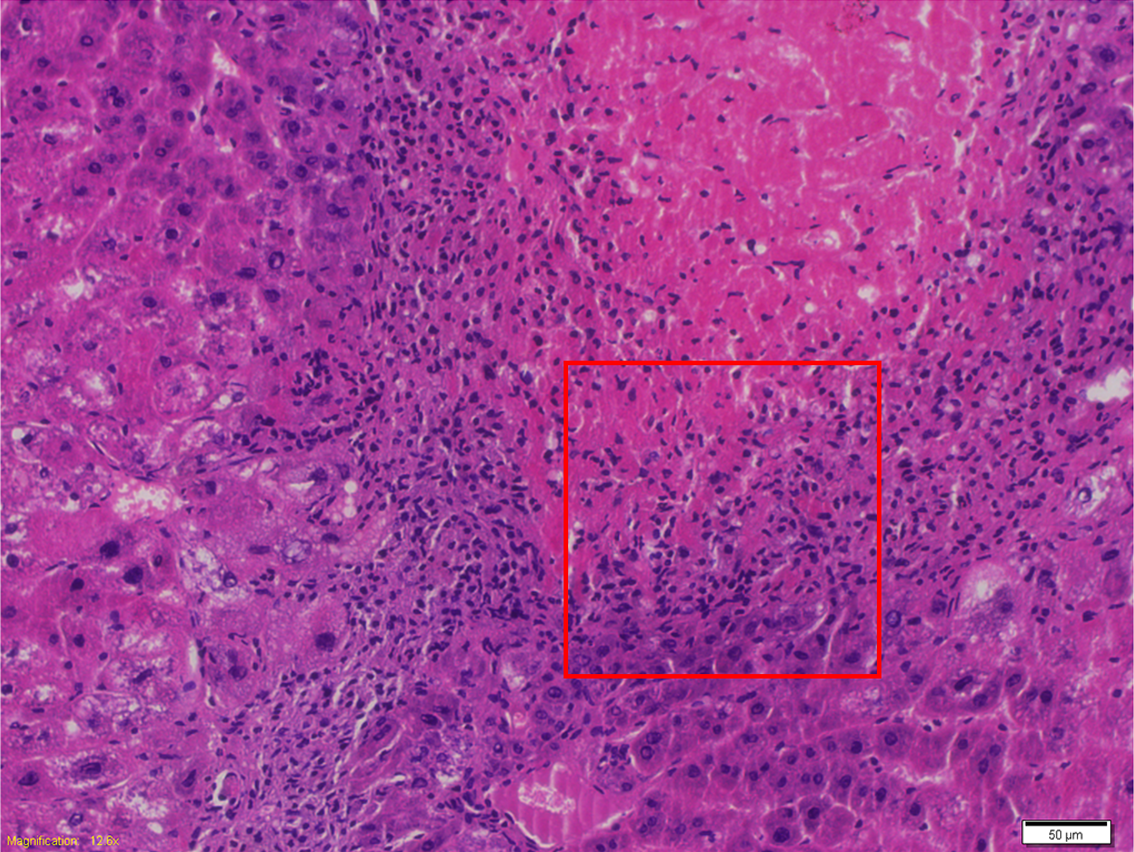

Supplement: Supplementary file 7 — Source Data for Figure 5 [file EMMM-15-e17230-s002.zip › Figure 5/5I/H&E for DB-rAAV-si-Cyp4a14.tif]

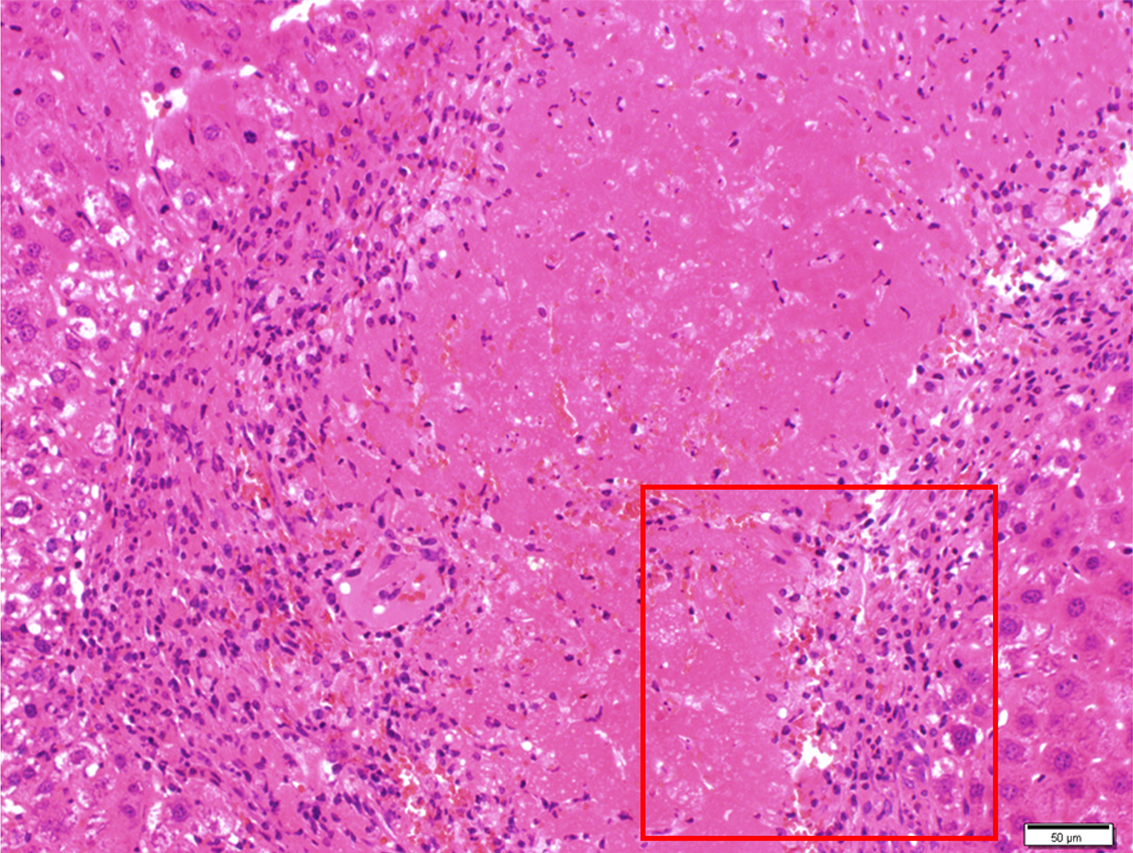

Supplement: Supplementary file 7 — Source Data for Figure 5 [file EMMM-15-e17230-s002.zip › Figure 5/5I/H&E for DB-rAAV-si-NC.tif]

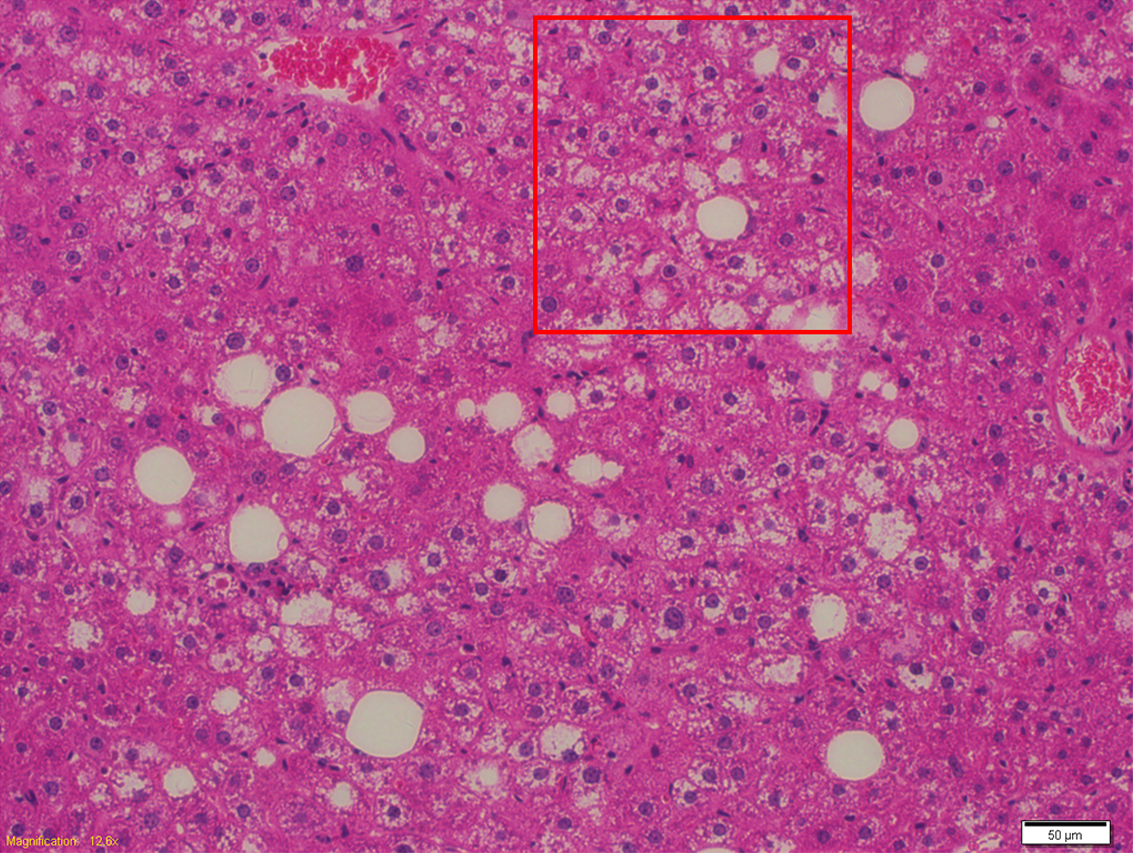

Supplement: Supplementary file 7 — Source Data for Figure 5 [file EMMM-15-e17230-s002.zip › Figure 5/5I/H&E for DB-rAAV-si-Pebp1.tif]

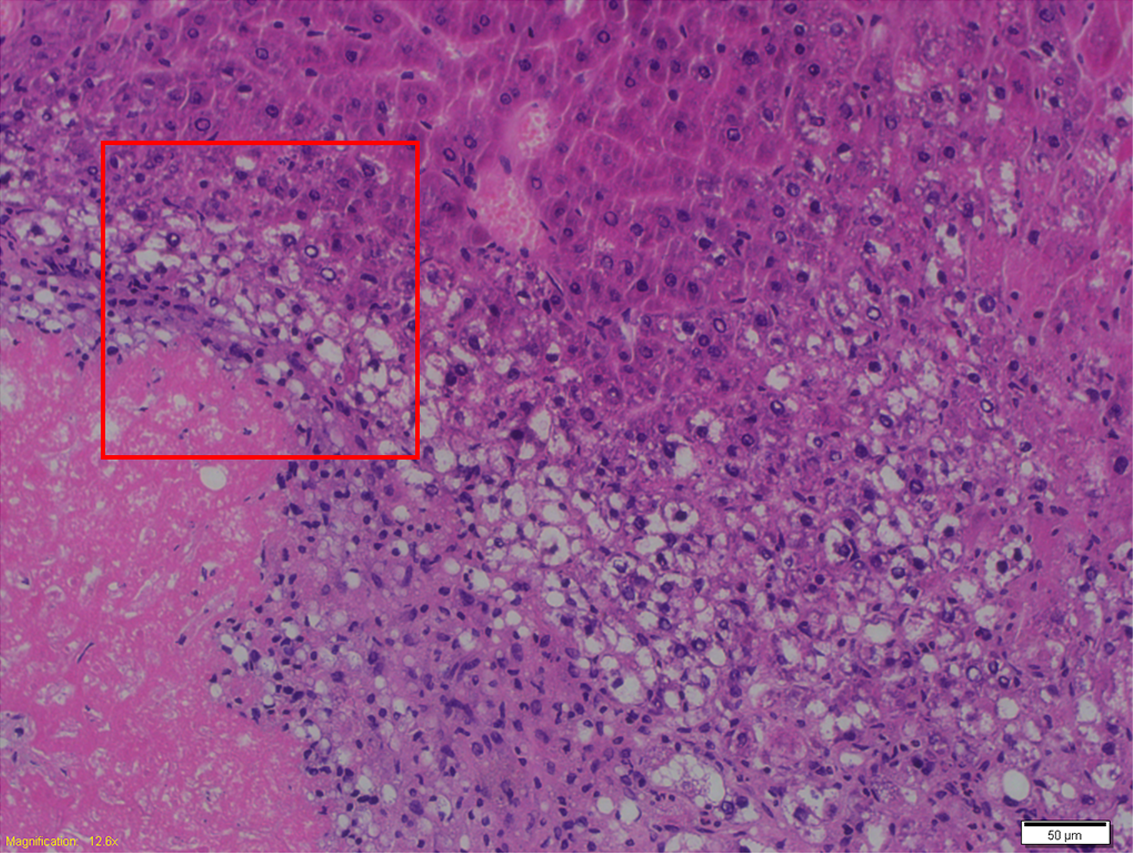

Supplement: Supplementary file 7 — Source Data for Figure 5 [file EMMM-15-e17230-s002.zip › Figure 5/5I/H&E for DB-rAAV-si-Tat.tif]

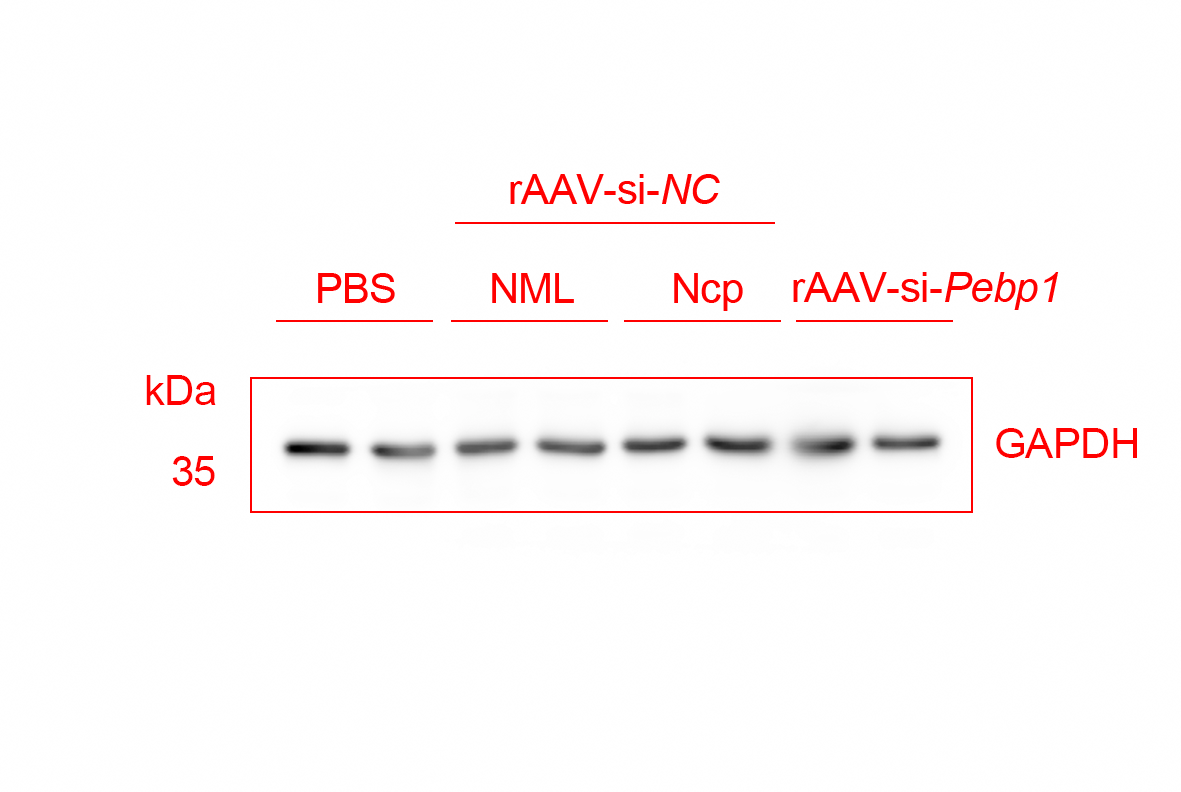

Supplement: Supplementary file 7 — Source Data for Figure 5 [file EMMM-15-e17230-s002.zip › Figure 5/5K/Western/GAPDH.tif]

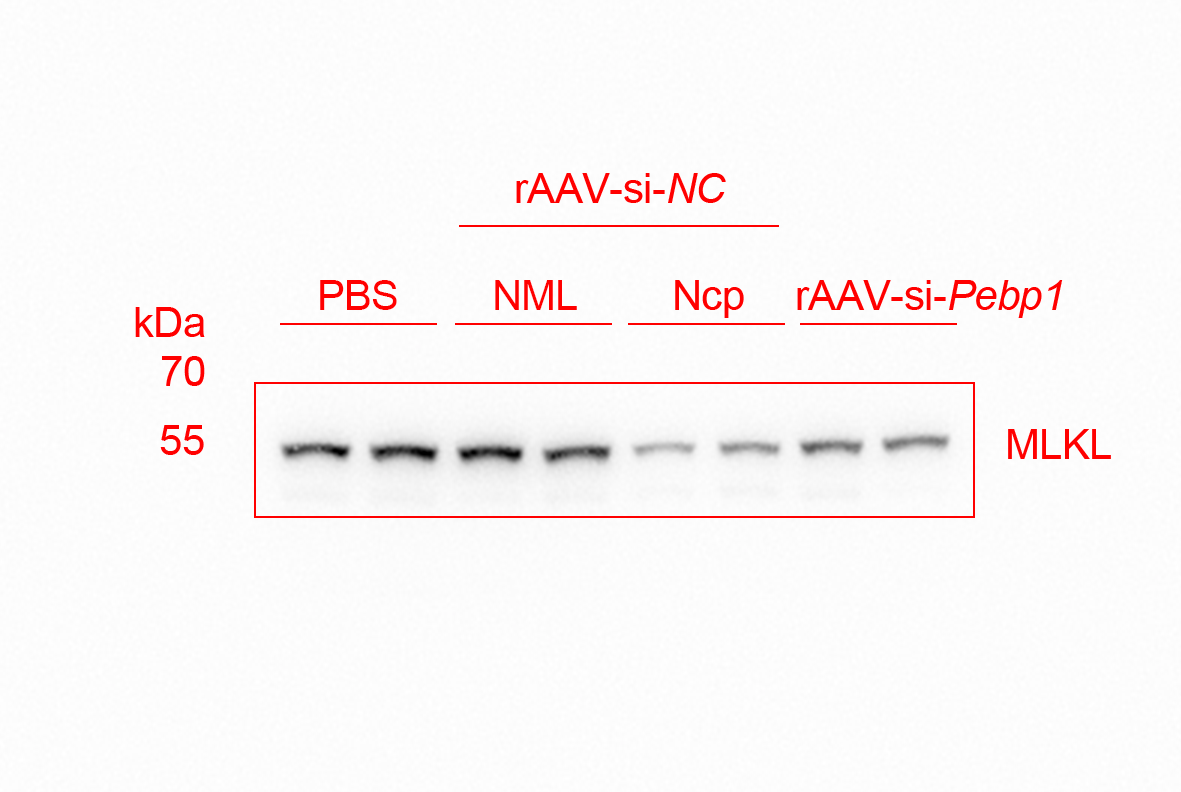

Supplement: Supplementary file 7 — Source Data for Figure 5 [file EMMM-15-e17230-s002.zip › Figure 5/5K/Western/MLKL.tif]

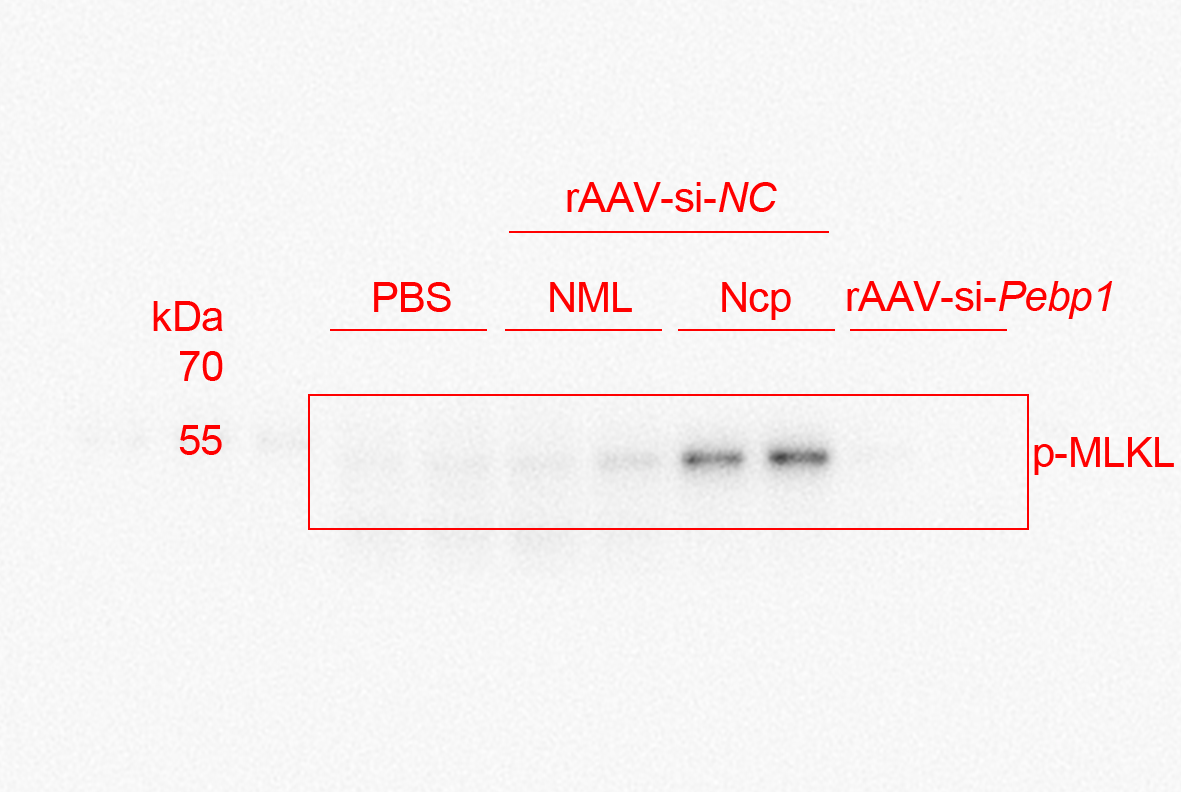

Supplement: Supplementary file 7 — Source Data for Figure 5 [file EMMM-15-e17230-s002.zip › Figure 5/5K/Western/p-MLKL.tif]

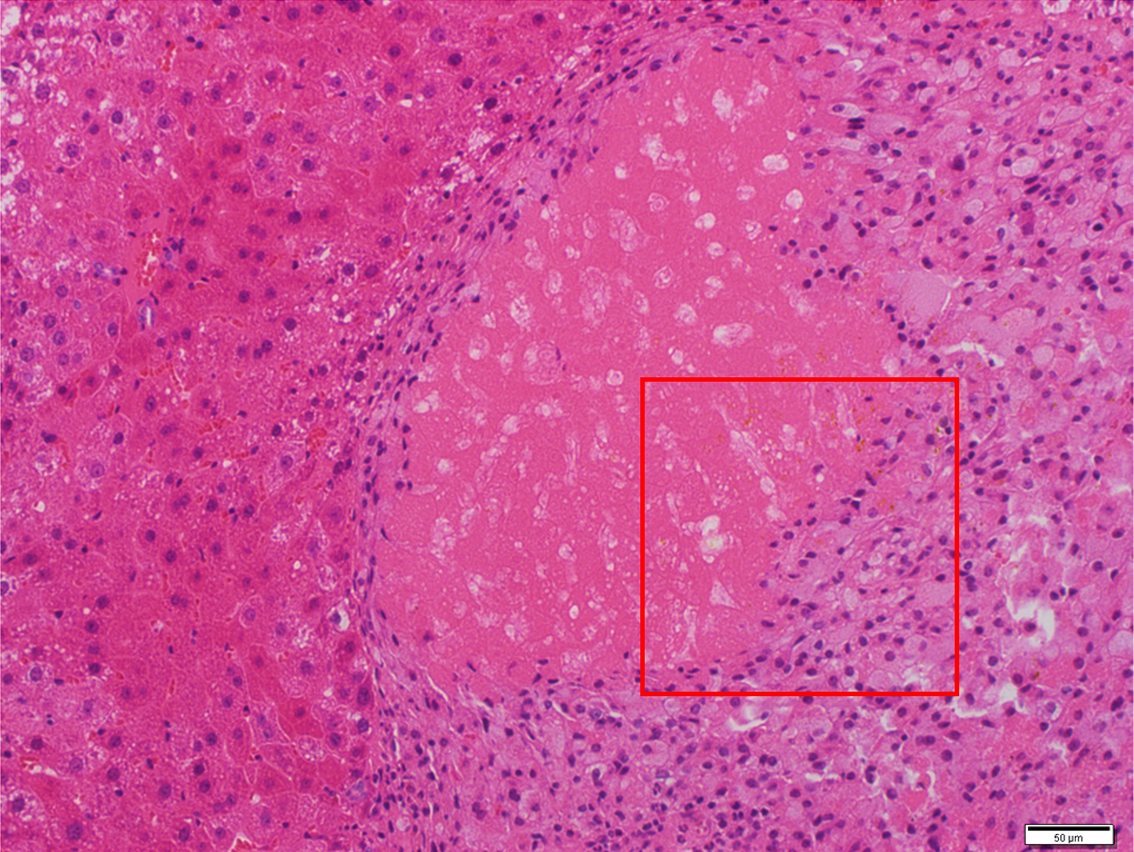

Supplement: Supplementary file 8 — Source Data for Figure 6 [file EMMM-15-e17230-s001.zip › Figure 6/6F/H&E for WT-HFD-rAAV-si-NC.tif]

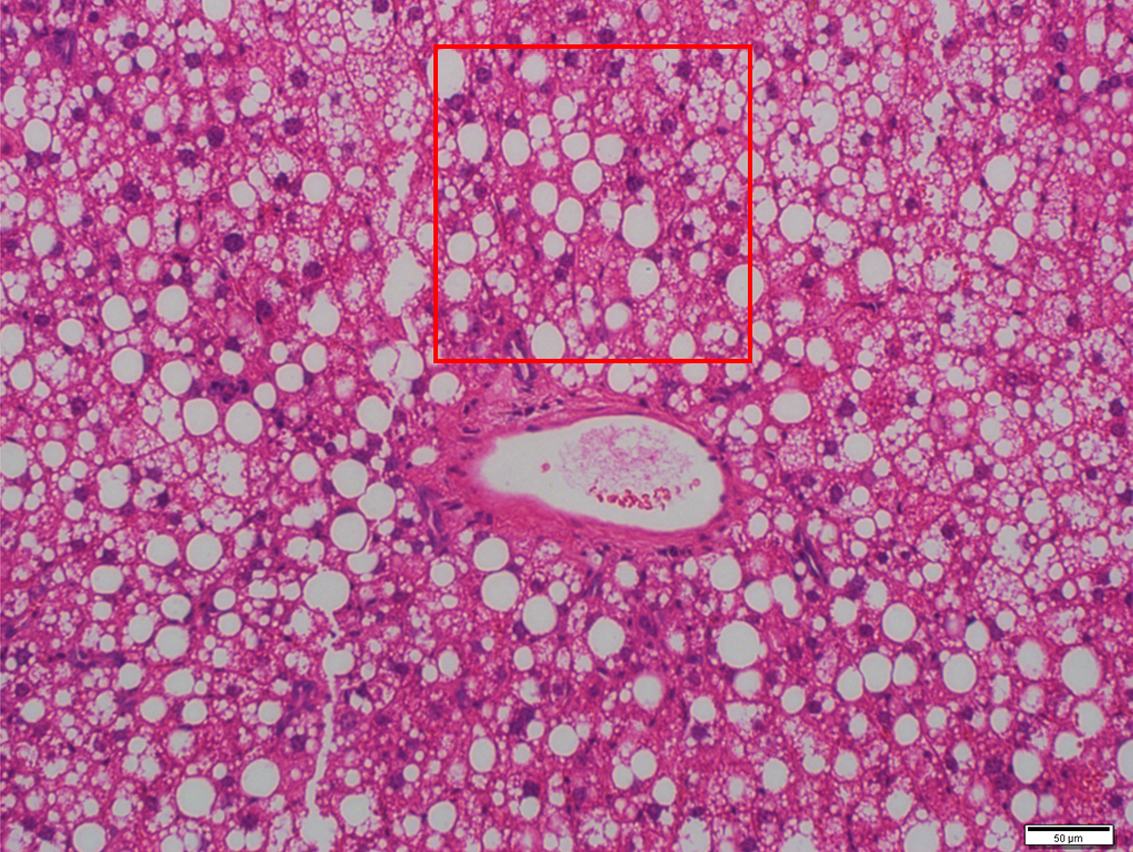

Supplement: Supplementary file 8 — Source Data for Figure 6 [file EMMM-15-e17230-s001.zip › Figure 6/6F/H&E for WT-HFD-rAAV-si-Pebp1.tif]
