# Supplementary figures and images for: AAV induces hepatic necroptosis and carcinoma in diabetic and obese mice dependent on Pebp1 pathway (part 2 of 2)
Source: EMBO Mol Med. 2023 Jun 5;15(7):e17230. doi: 10.15252/emmm.202217230 (PMC10331584; doi:10.15252/emmm.202217230)

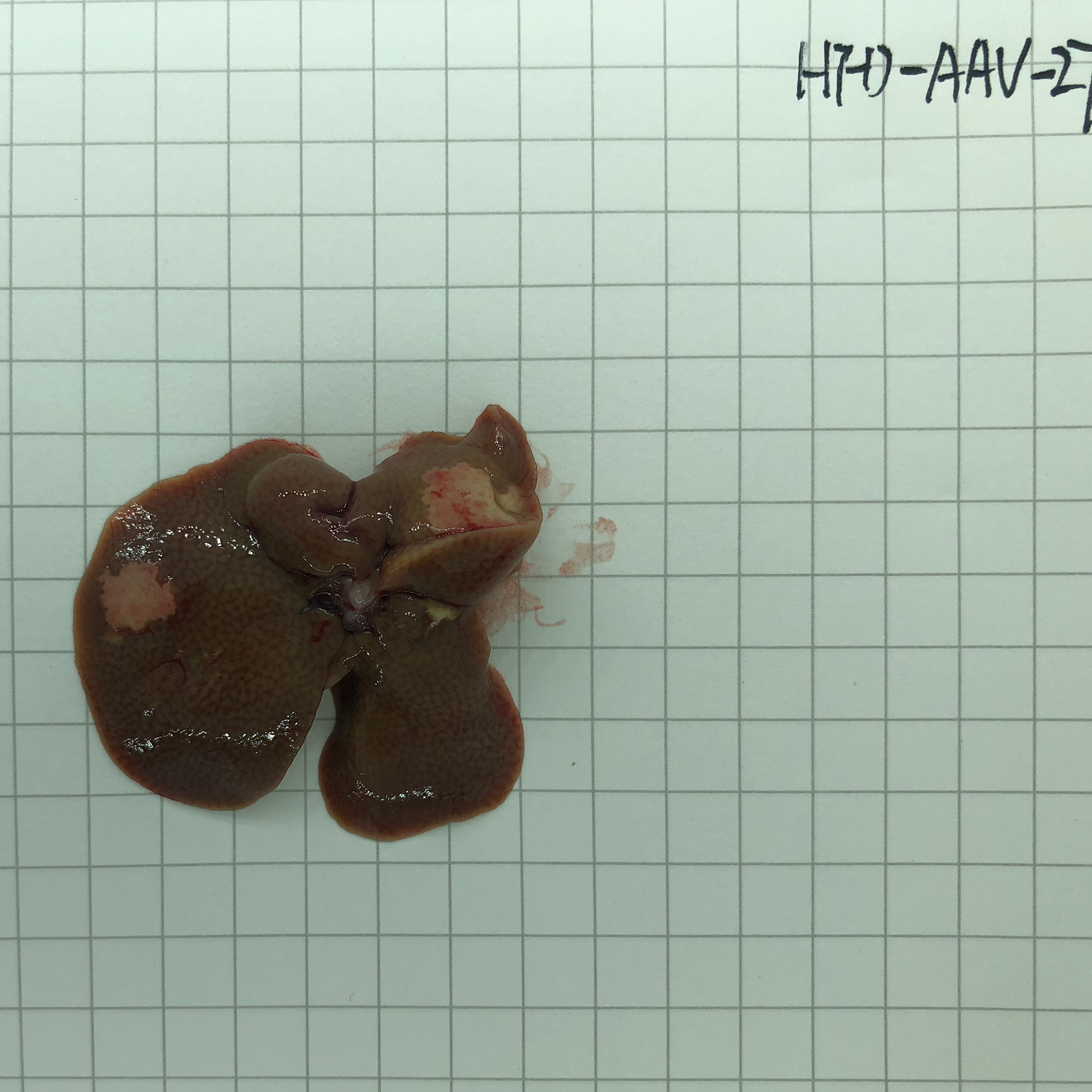

Supplement: Supplementary file 8 — Source Data for Figure 6 [file EMMM-15-e17230-s001.zip › Figure 6/6F/WT-HFD-rAAV-si-NC-Liver.tif]

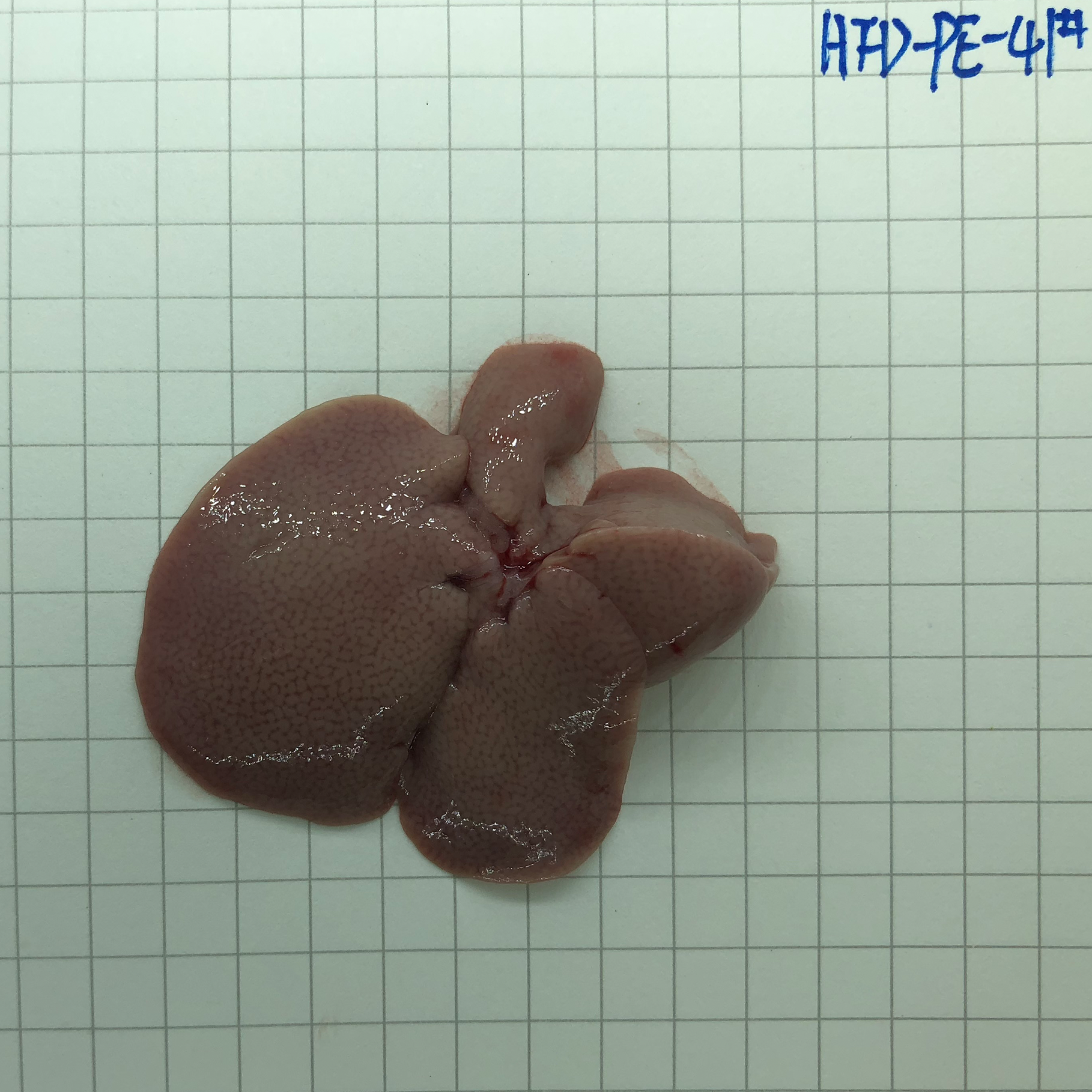

Supplement: Supplementary file 8 — Source Data for Figure 6 [file EMMM-15-e17230-s001.zip › Figure 6/6F/WT-HFD-rAAV-si-Pebp1-Liver.tif]

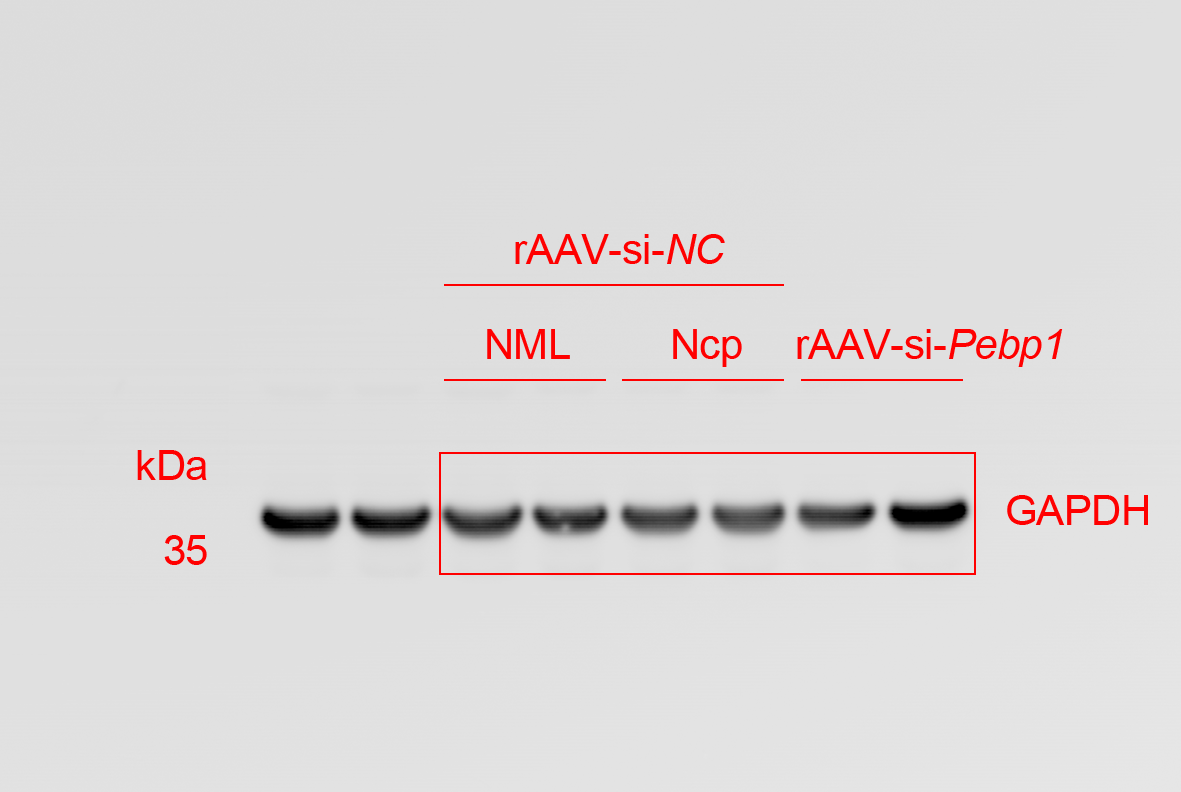

Supplement: Supplementary file 8 — Source Data for Figure 6 [file EMMM-15-e17230-s001.zip › Figure 6/6G/Western/GAPDH.tif]

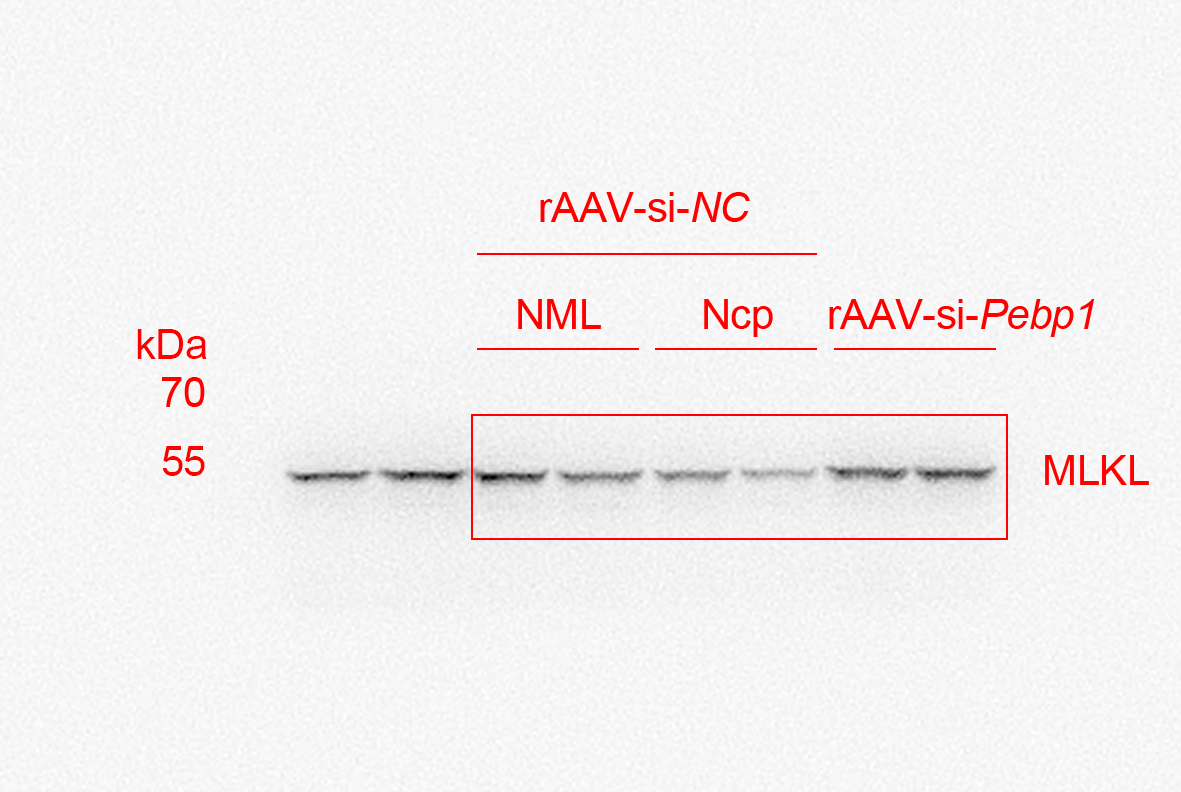

Supplement: Supplementary file 8 — Source Data for Figure 6 [file EMMM-15-e17230-s001.zip › Figure 6/6G/Western/MLKL.tif]

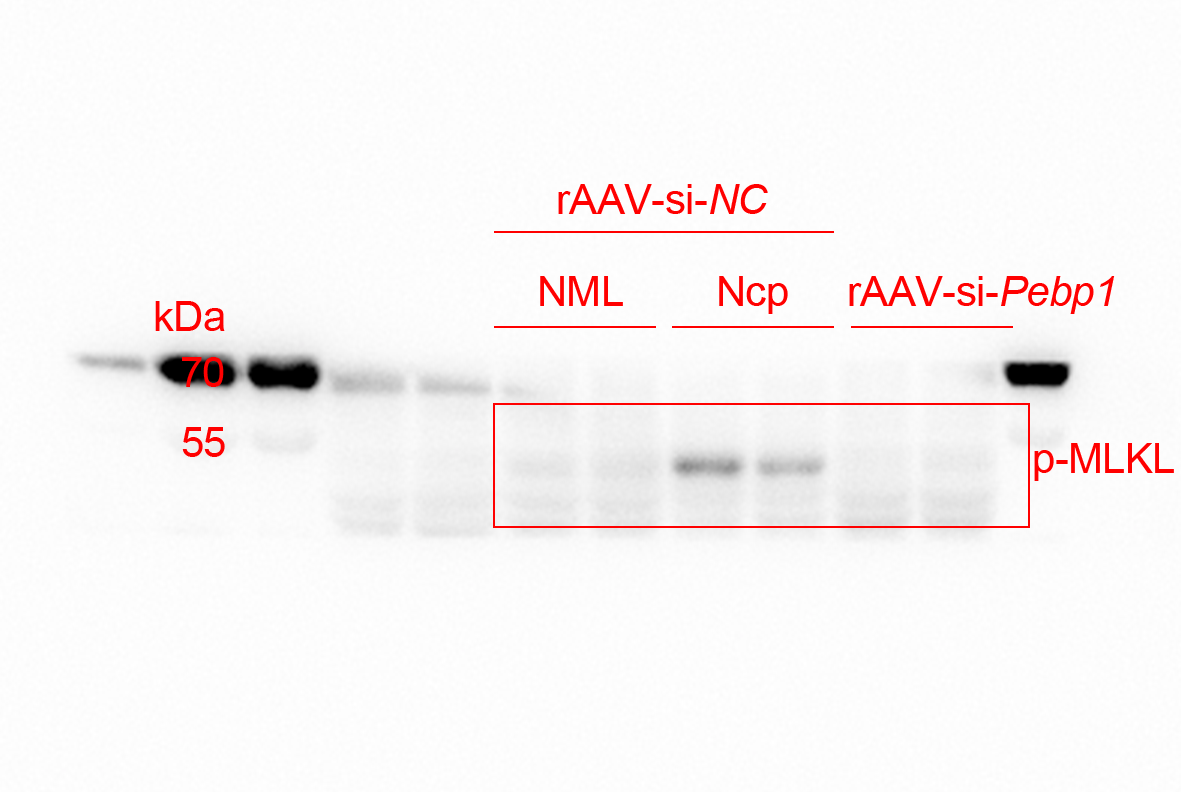

Supplement: Supplementary file 8 — Source Data for Figure 6 [file EMMM-15-e17230-s001.zip › Figure 6/6G/Western/p-MLKL.tif]

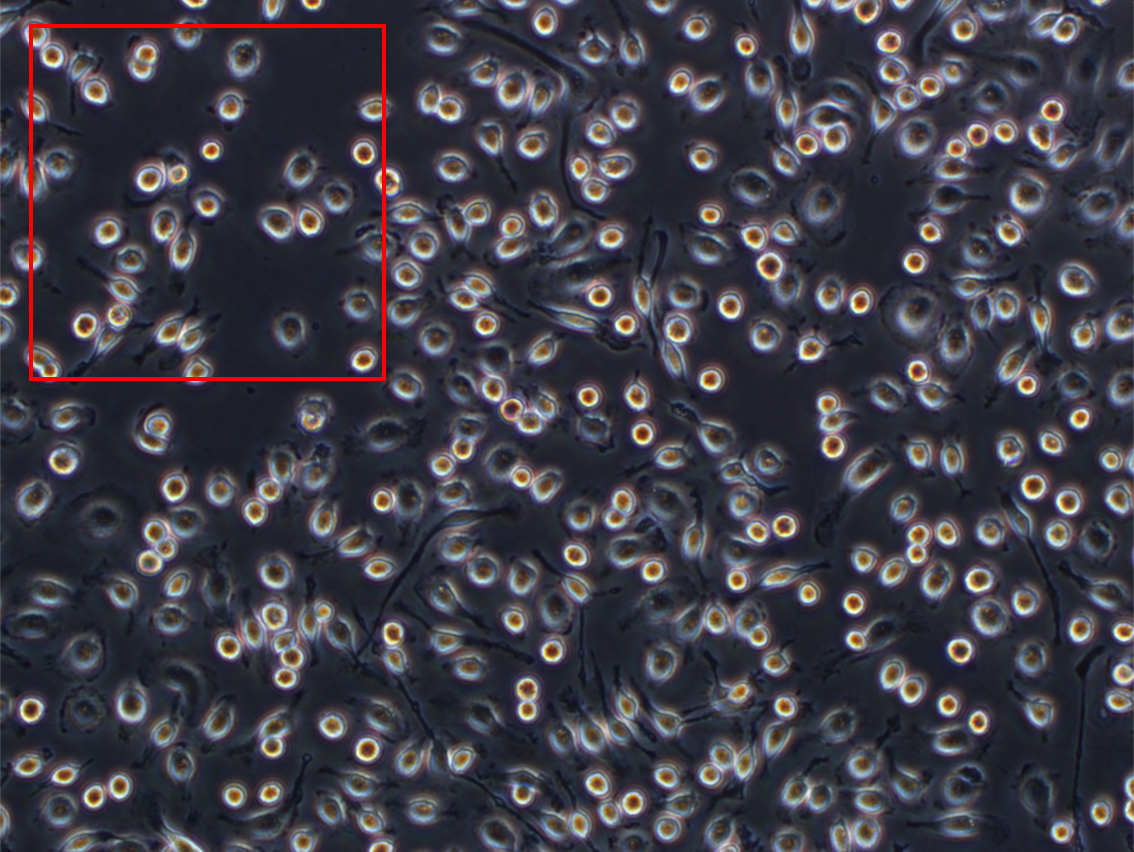

Supplement: Supplementary file 9 — Source Data for Figure 7 [file EMMM-15-e17230-s008.zip › Figure 7/7C/BF for si-NC-Vehicle.tif]

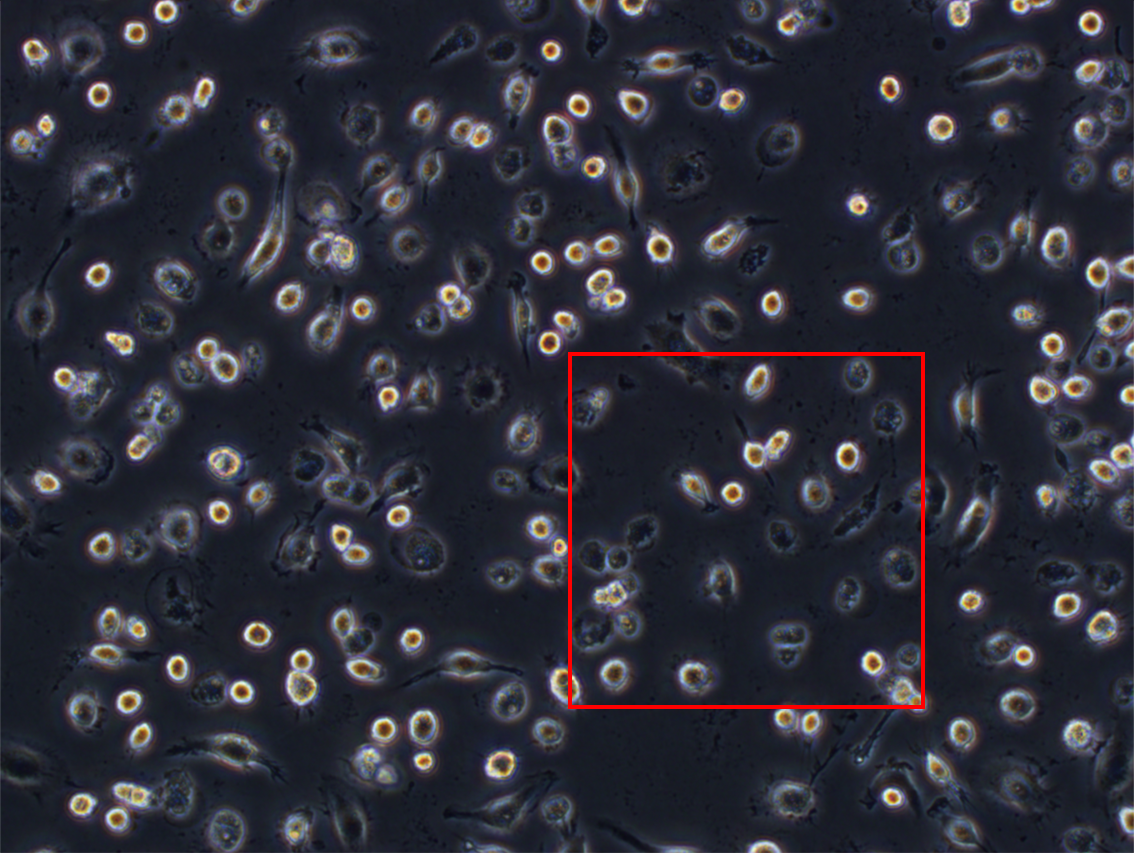

Supplement: Supplementary file 9 — Source Data for Figure 7 [file EMMM-15-e17230-s008.zip › Figure 7/7C/BF for si-NC-pIC+zVAD.tif]

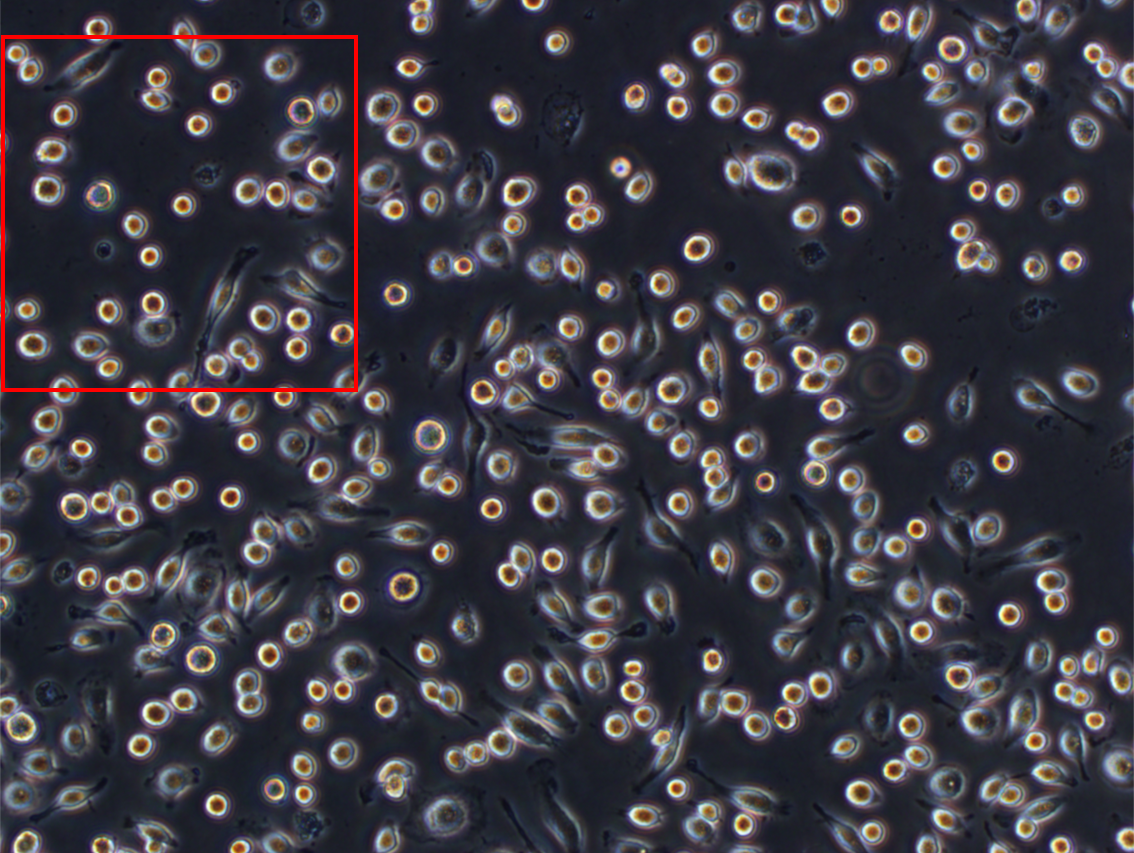

Supplement: Supplementary file 9 — Source Data for Figure 7 [file EMMM-15-e17230-s008.zip › Figure 7/7C/BF for si-Pebp1-Vehicle.tif]

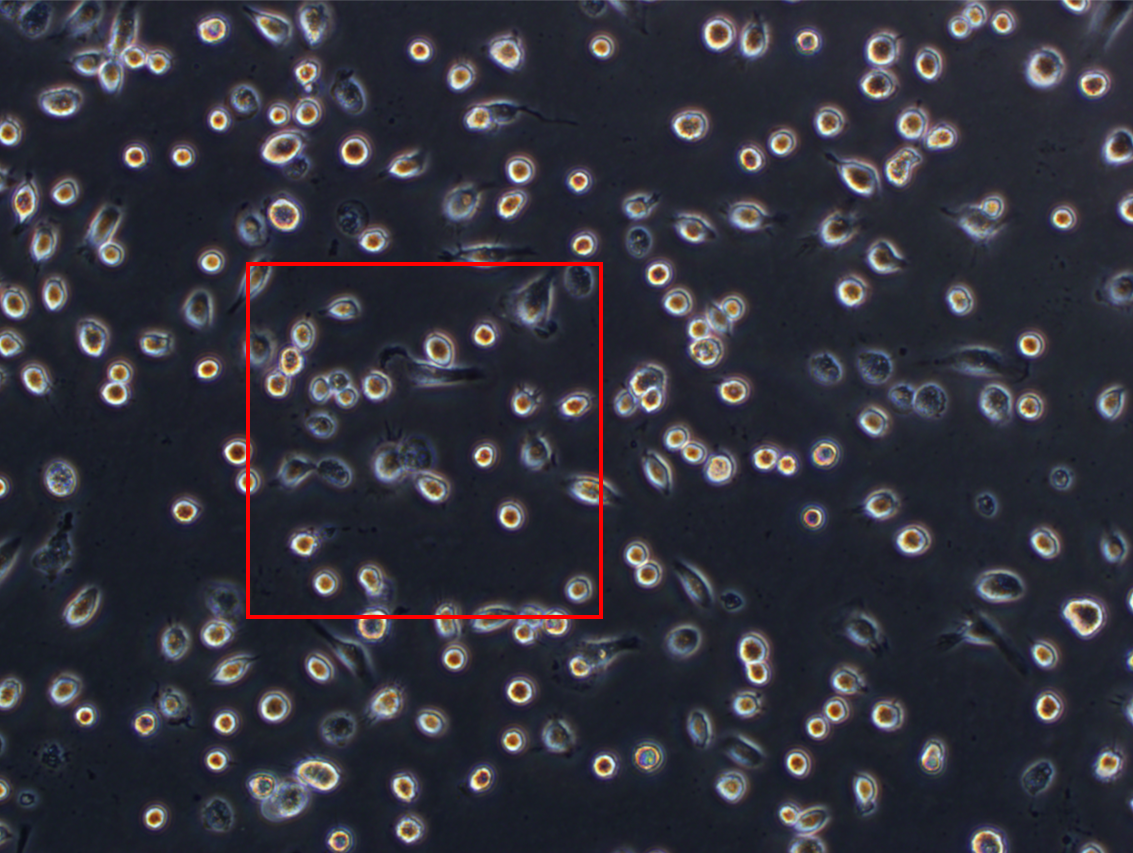

Supplement: Supplementary file 9 — Source Data for Figure 7 [file EMMM-15-e17230-s008.zip › Figure 7/7C/BF for si-Pebp1-pIC+zVAD.tif]

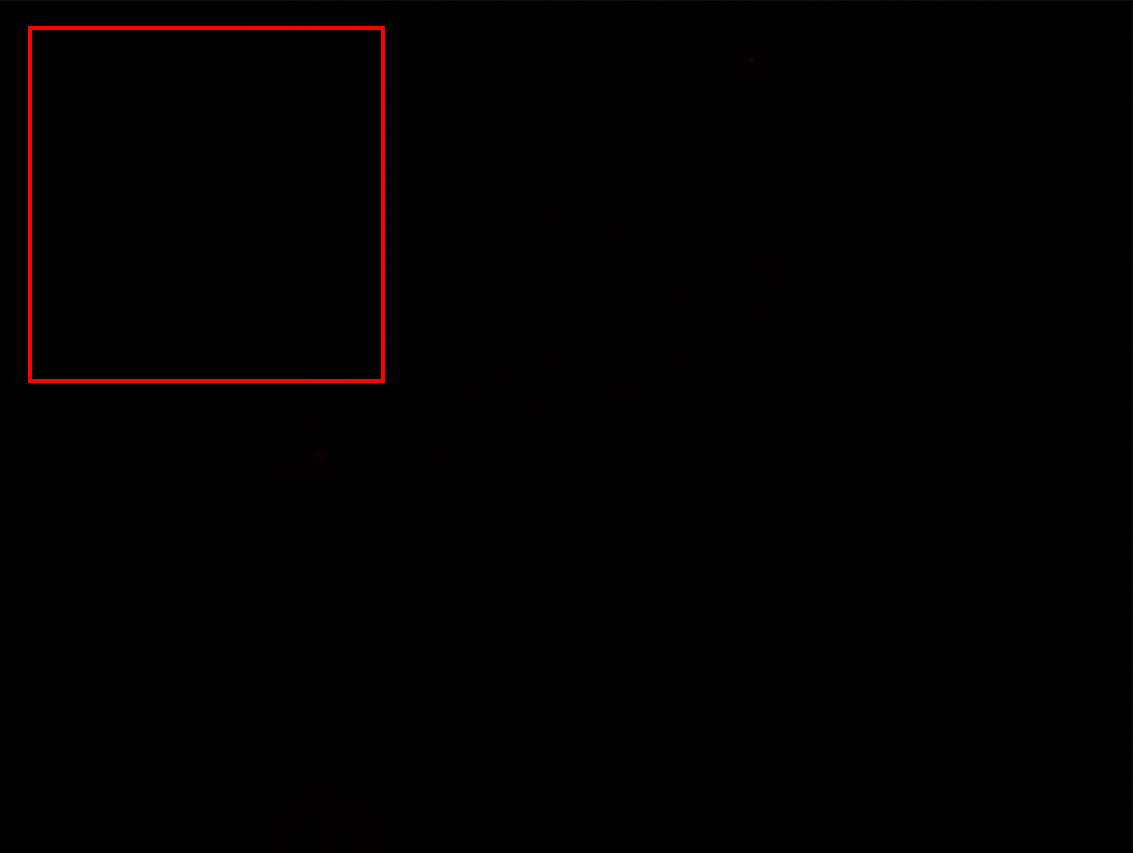

Supplement: Supplementary file 9 — Source Data for Figure 7 [file EMMM-15-e17230-s008.zip › Figure 7/7C/PI staining for si-NC-Vehicle.tif]

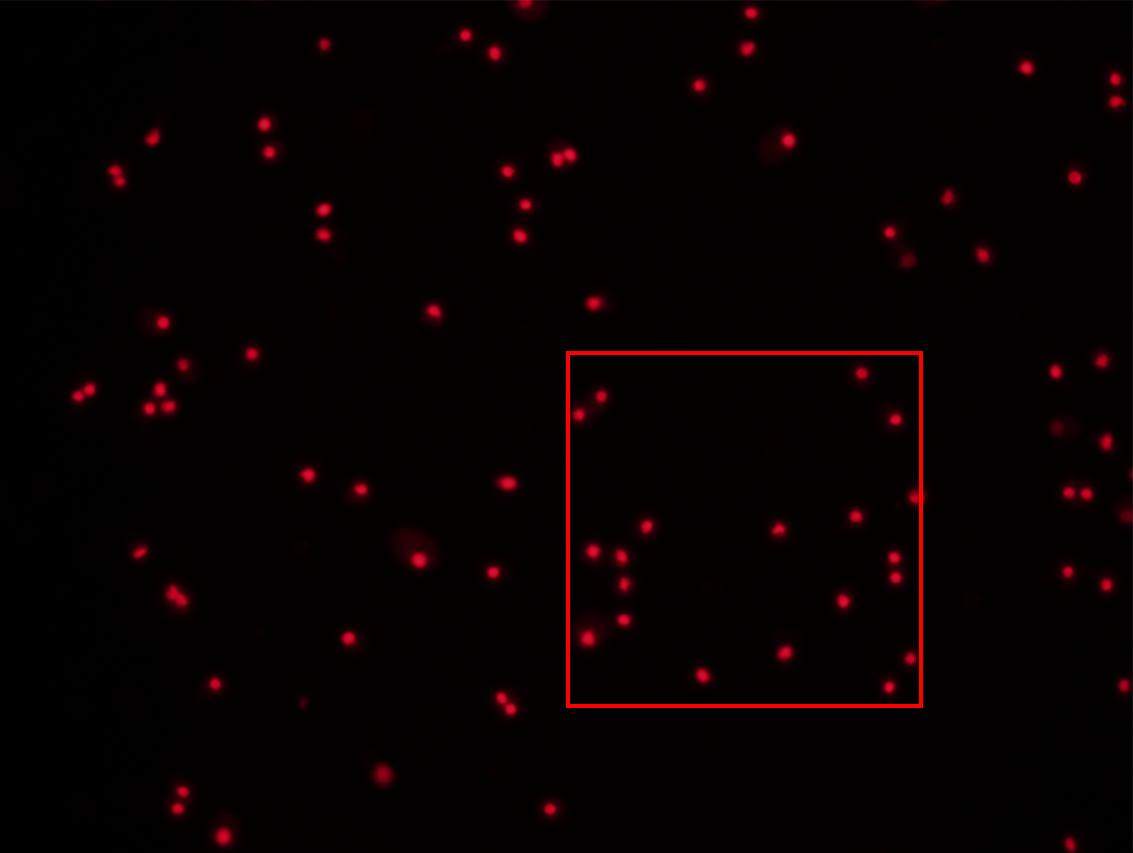

Supplement: Supplementary file 9 — Source Data for Figure 7 [file EMMM-15-e17230-s008.zip › Figure 7/7C/PI staining for si-NC-pIC+zVAD.tif]

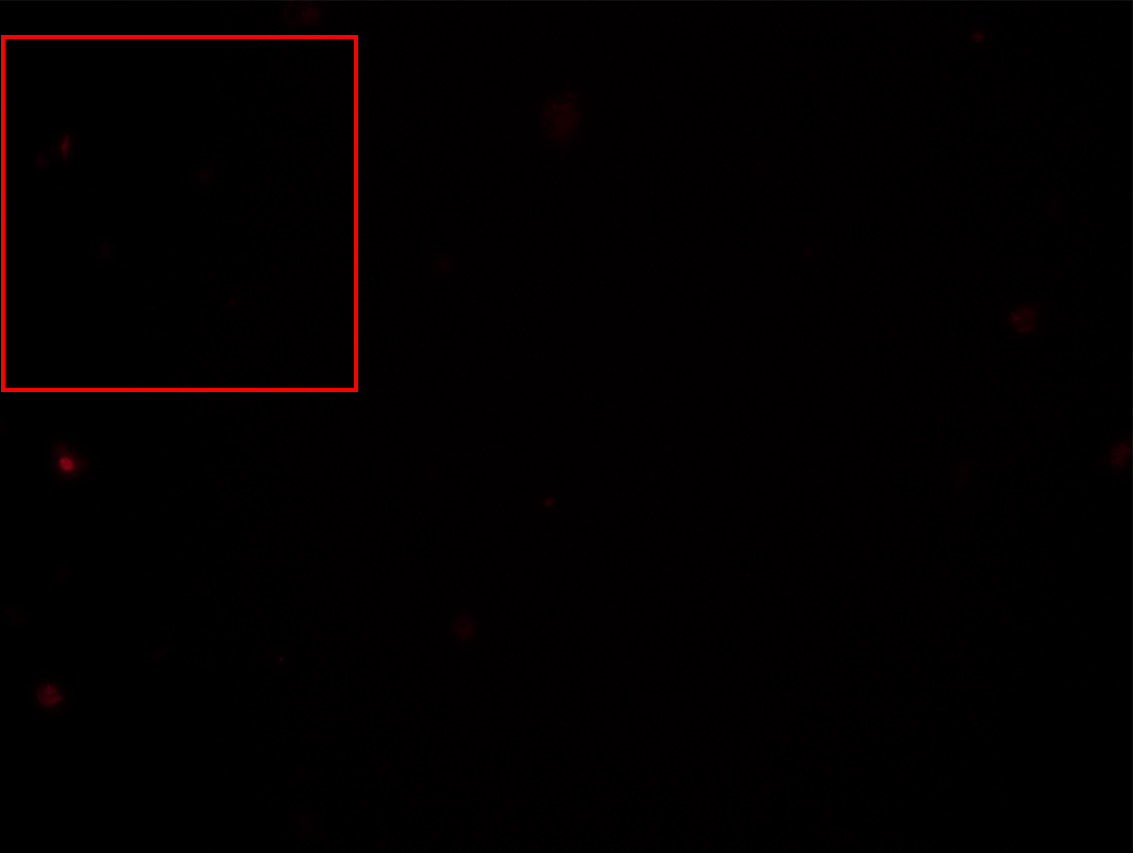

Supplement: Supplementary file 9 — Source Data for Figure 7 [file EMMM-15-e17230-s008.zip › Figure 7/7C/PI staining for si-Pebp1-Vehicle.tif]

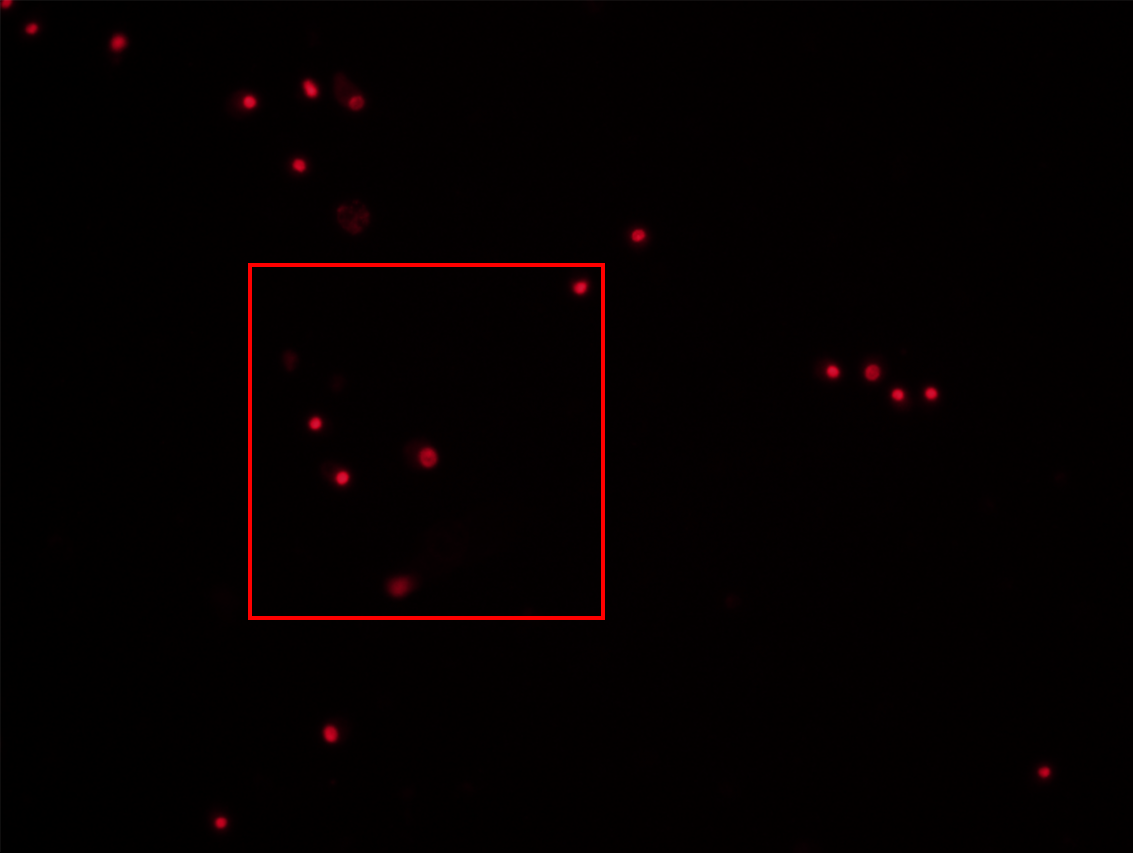

Supplement: Supplementary file 9 — Source Data for Figure 7 [file EMMM-15-e17230-s008.zip › Figure 7/7C/PI staining for si-Pebp1-pIC+zVAD.tif]

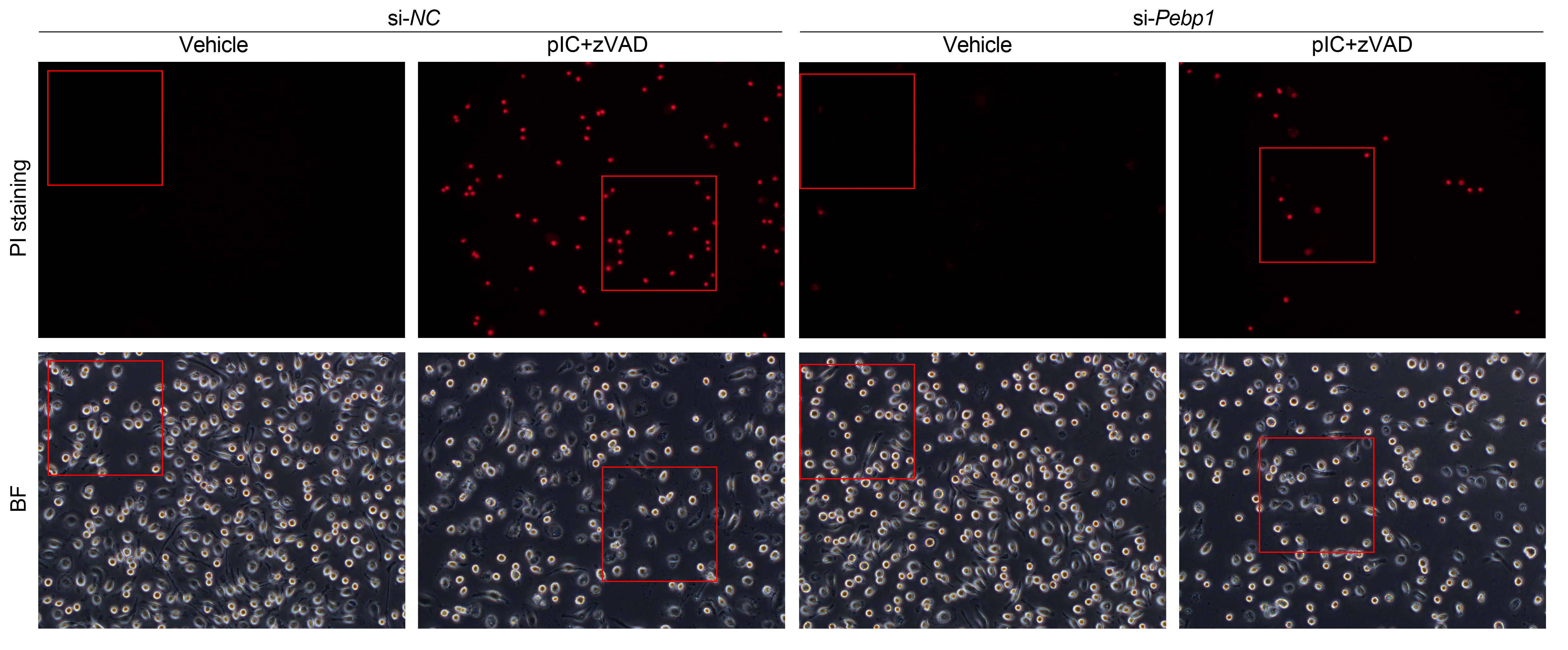

Supplement: Supplementary file 9 — Source Data for Figure 7 [file EMMM-15-e17230-s008.zip › Figure 7/7C/PI staining.tif]

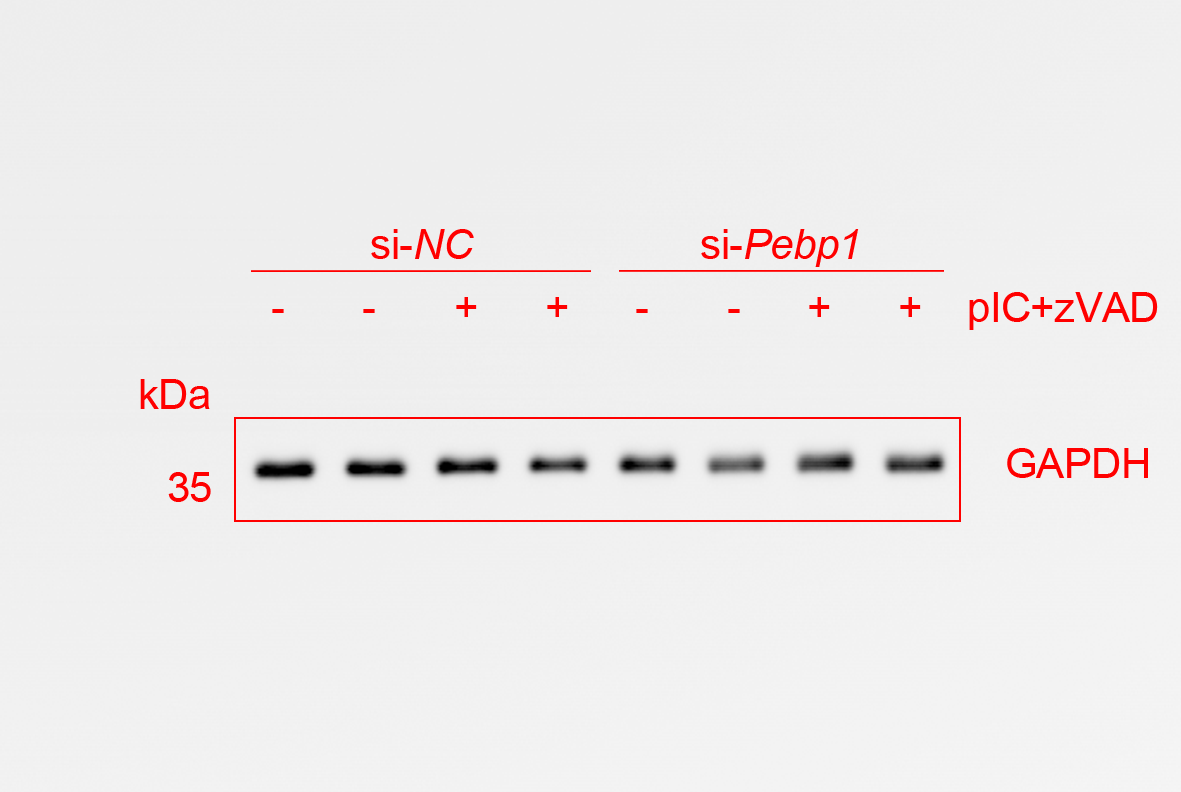

Supplement: Supplementary file 9 — Source Data for Figure 7 [file EMMM-15-e17230-s008.zip › Figure 7/7D/Western/GAPDH.tif]

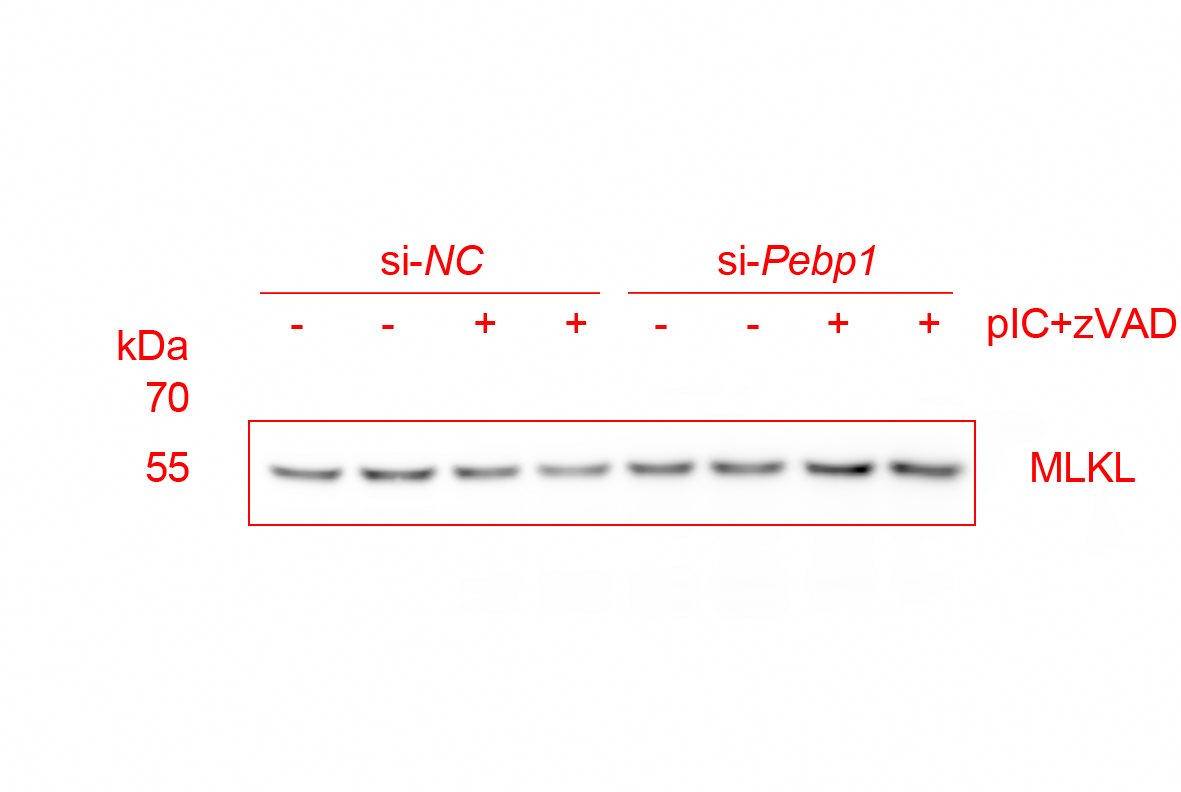

Supplement: Supplementary file 9 — Source Data for Figure 7 [file EMMM-15-e17230-s008.zip › Figure 7/7D/Western/MLKL.tif]

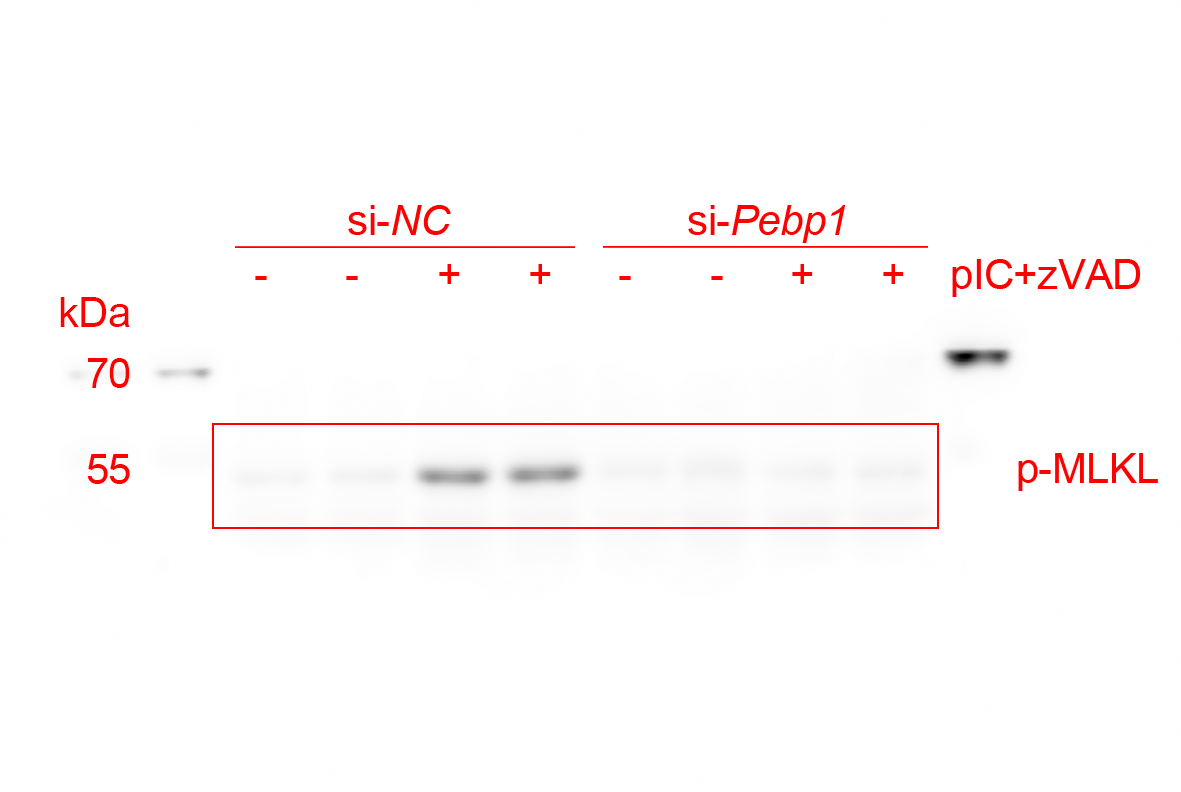

Supplement: Supplementary file 9 — Source Data for Figure 7 [file EMMM-15-e17230-s008.zip › Figure 7/7D/Western/p-MLKL.tif]

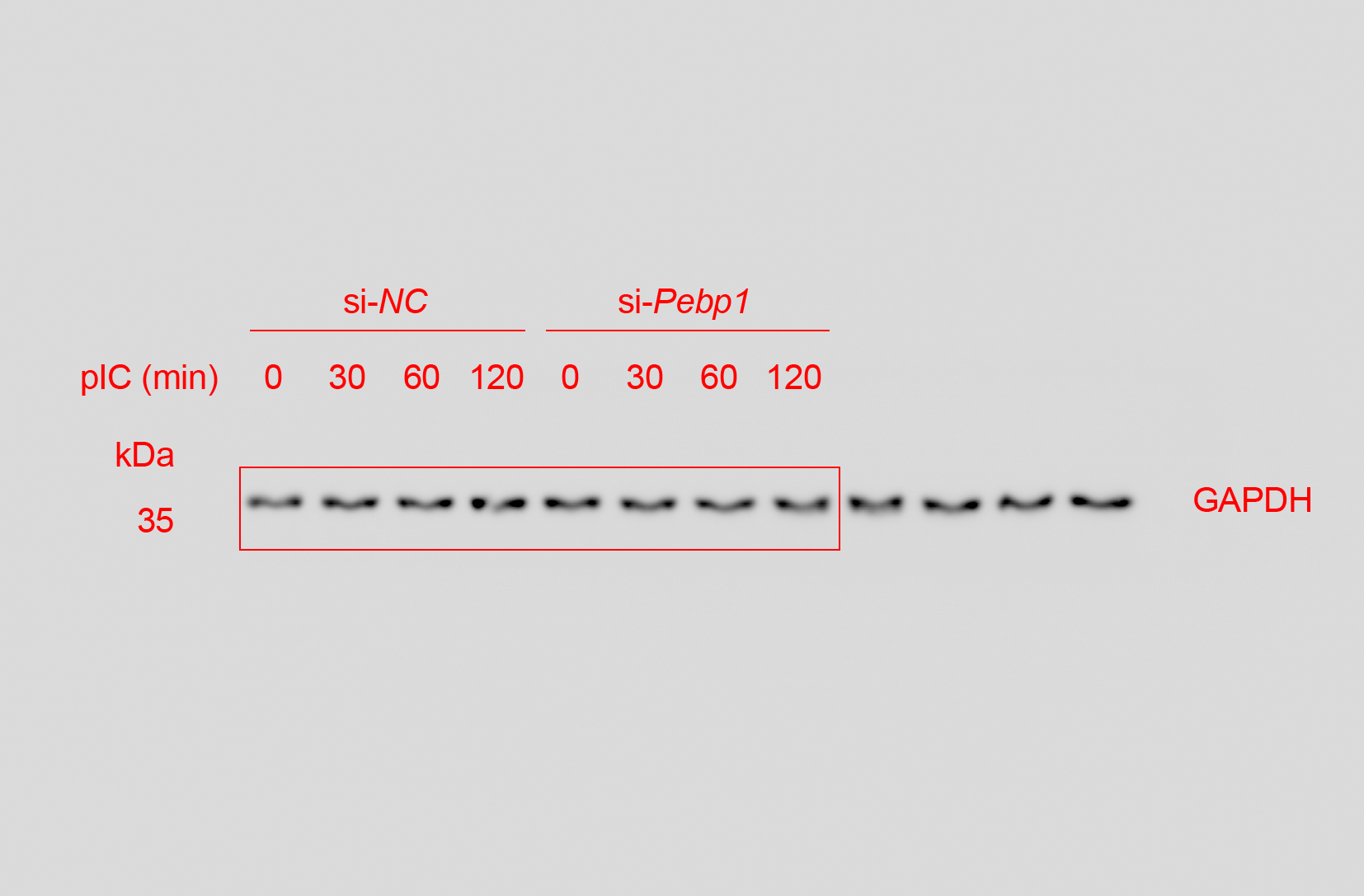

Supplement: Supplementary file 9 — Source Data for Figure 7 [file EMMM-15-e17230-s008.zip › Figure 7/7E/Western/GAPDH.tif]

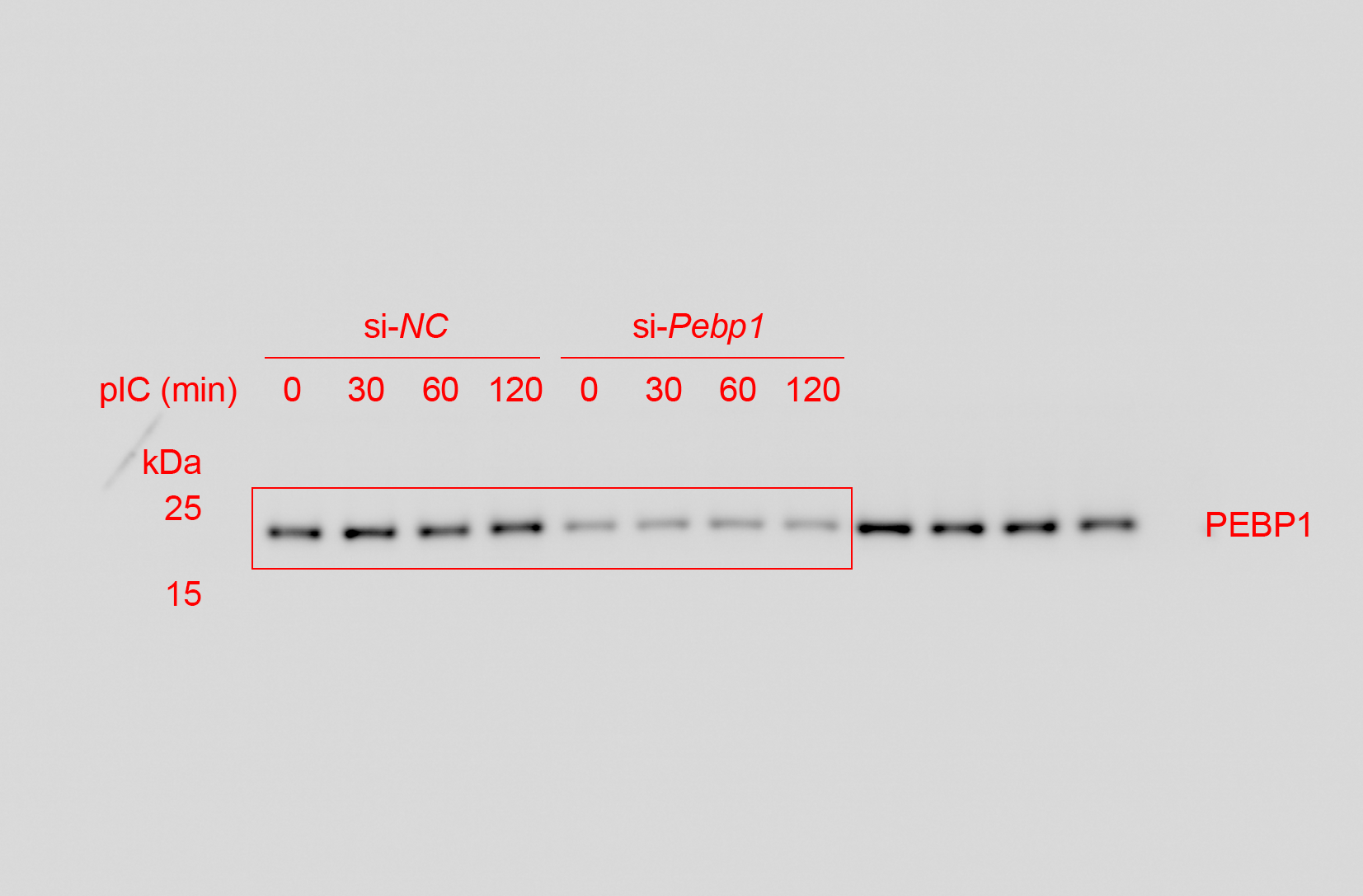

Supplement: Supplementary file 9 — Source Data for Figure 7 [file EMMM-15-e17230-s008.zip › Figure 7/7E/Western/PEBP1.tif]

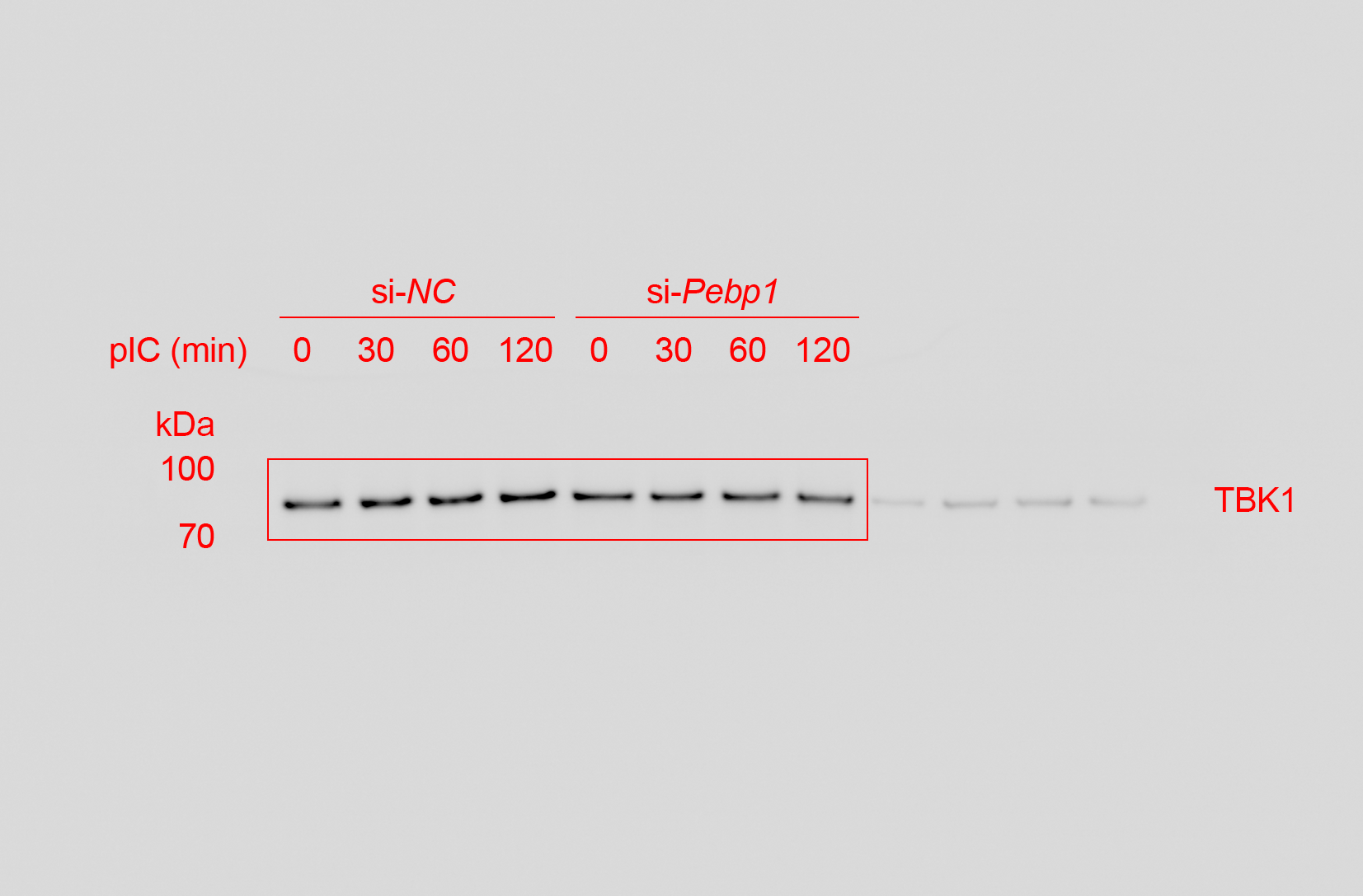

Supplement: Supplementary file 9 — Source Data for Figure 7 [file EMMM-15-e17230-s008.zip › Figure 7/7E/Western/TBK1.tif]

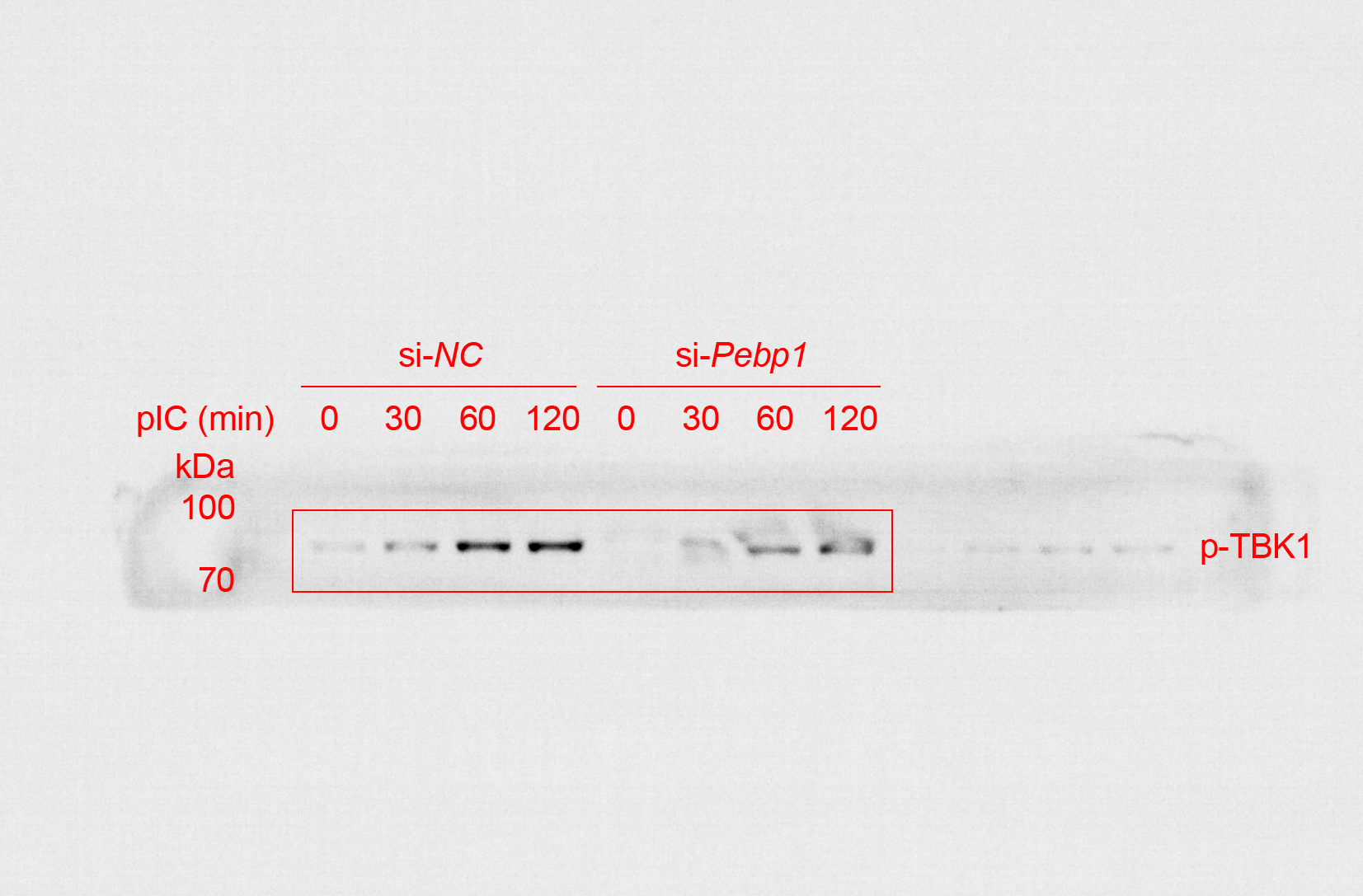

Supplement: Supplementary file 9 — Source Data for Figure 7 [file EMMM-15-e17230-s008.zip › Figure 7/7E/Western/p-TBK1.tif]

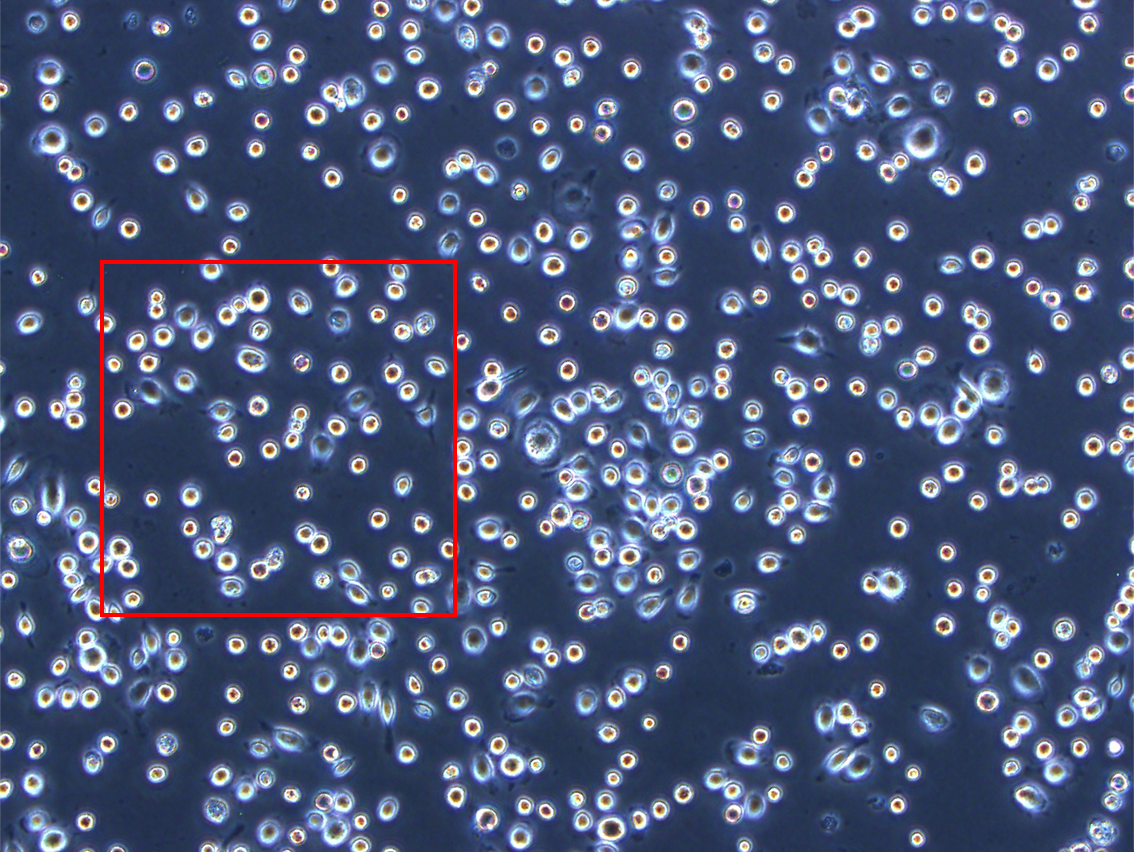

Supplement: Supplementary file 9 — Source Data for Figure 7 [file EMMM-15-e17230-s008.zip › Figure 7/7H/BF for si-NC-Vehicle.tif]

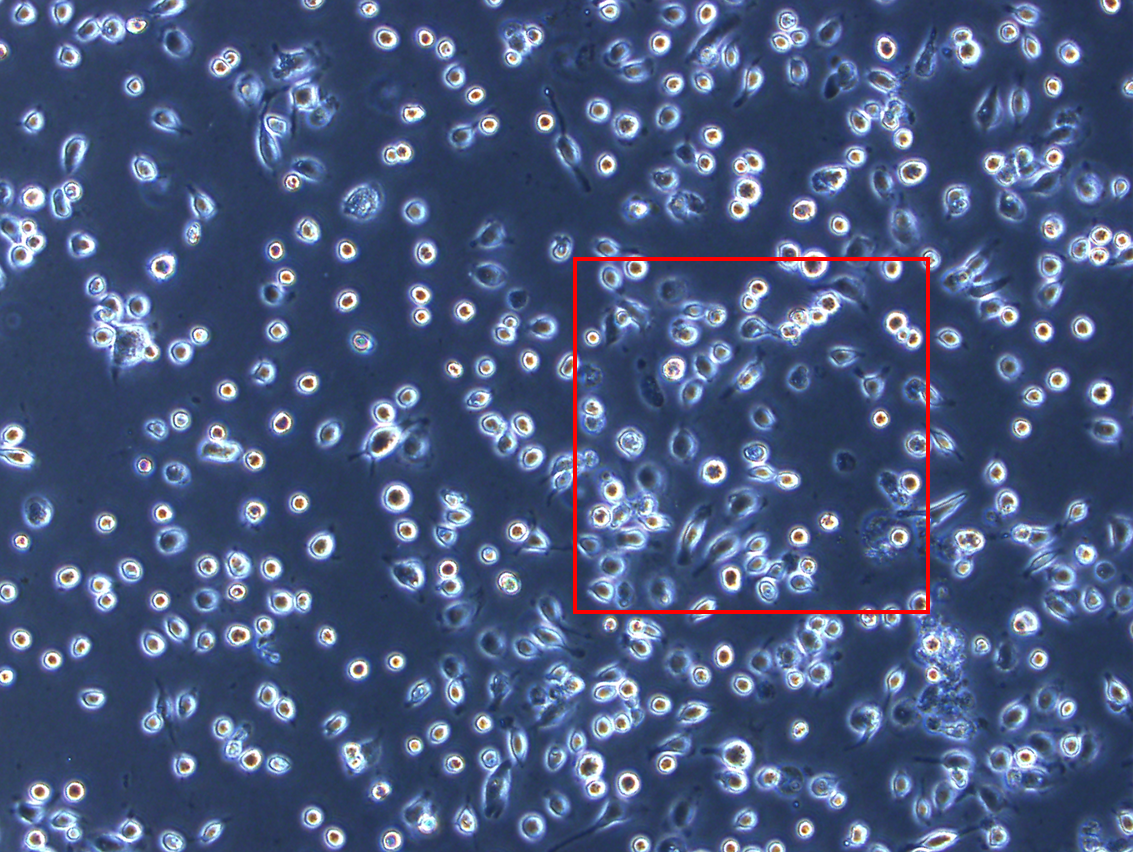

Supplement: Supplementary file 9 — Source Data for Figure 7 [file EMMM-15-e17230-s008.zip › Figure 7/7H/BF for si-NC-pIC+zVAD.tif]

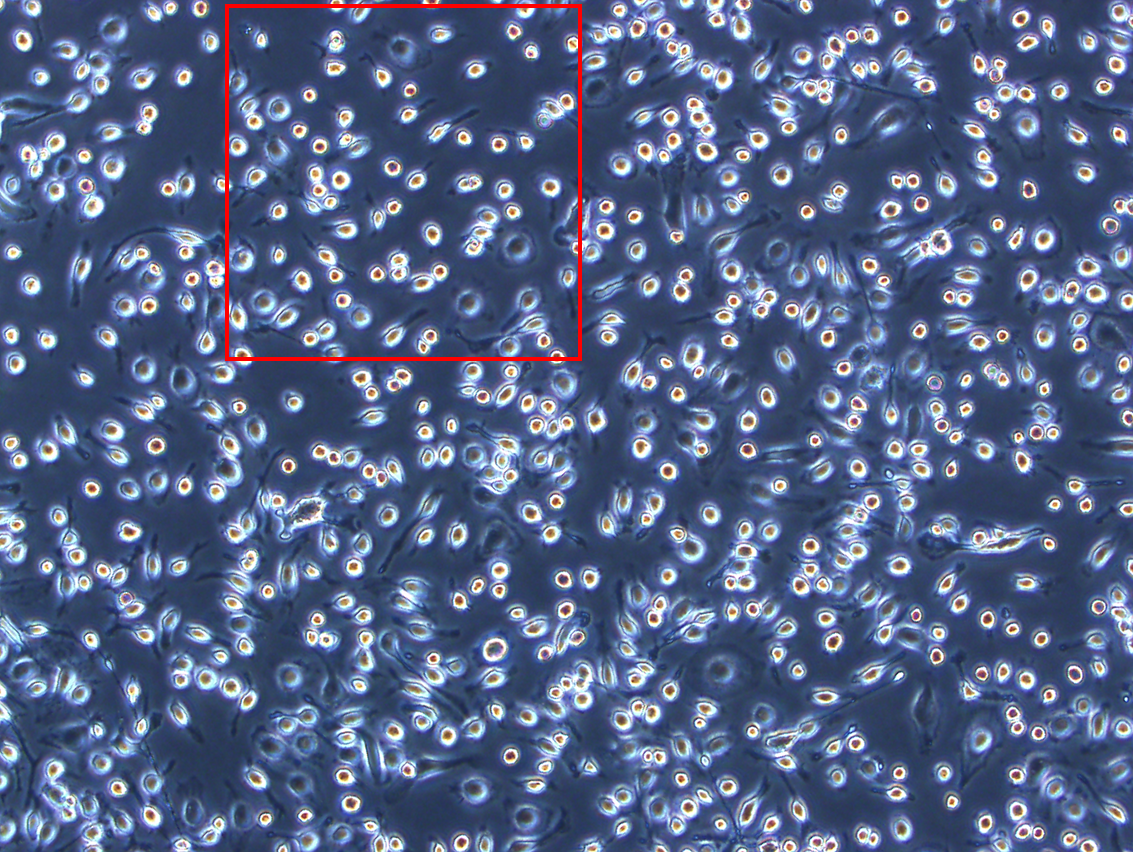

Supplement: Supplementary file 9 — Source Data for Figure 7 [file EMMM-15-e17230-s008.zip › Figure 7/7H/BF for si-Tbk1-Vehicle.tif]

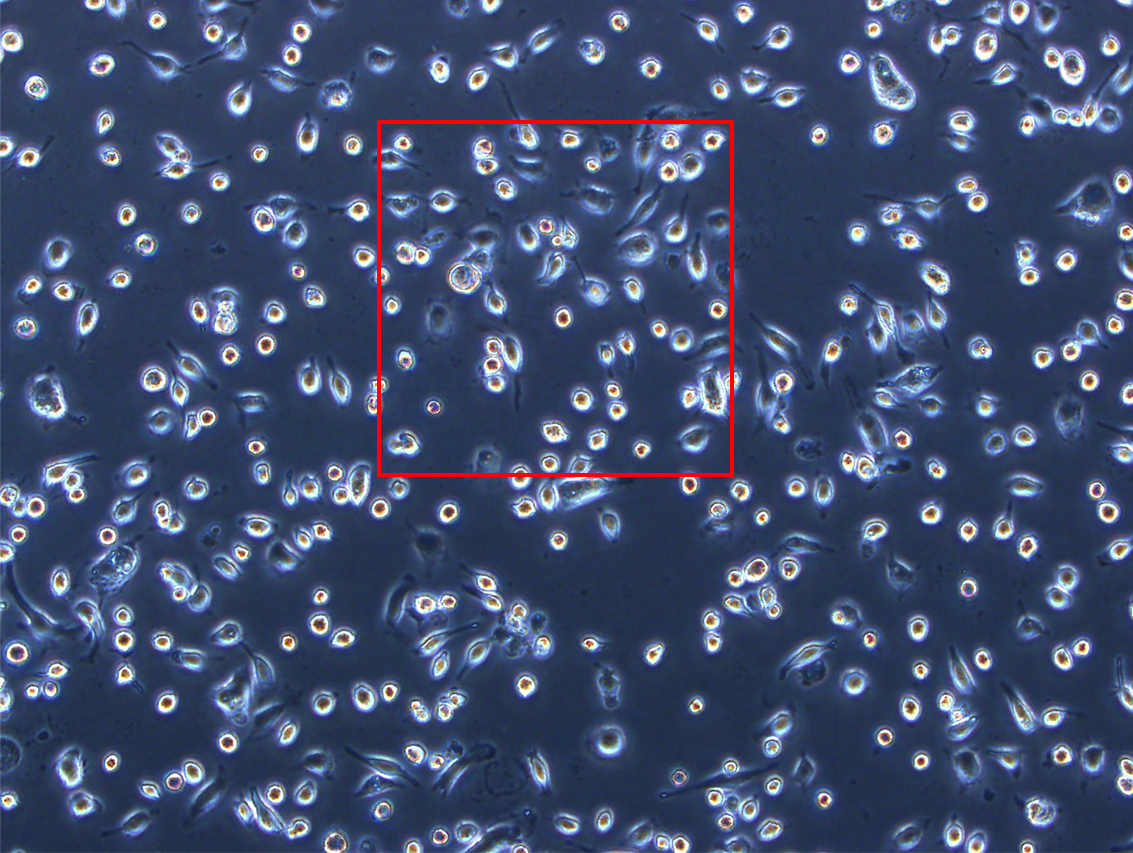

Supplement: Supplementary file 9 — Source Data for Figure 7 [file EMMM-15-e17230-s008.zip › Figure 7/7H/BF for si-Tbk1-pIC+zVAD.tif]

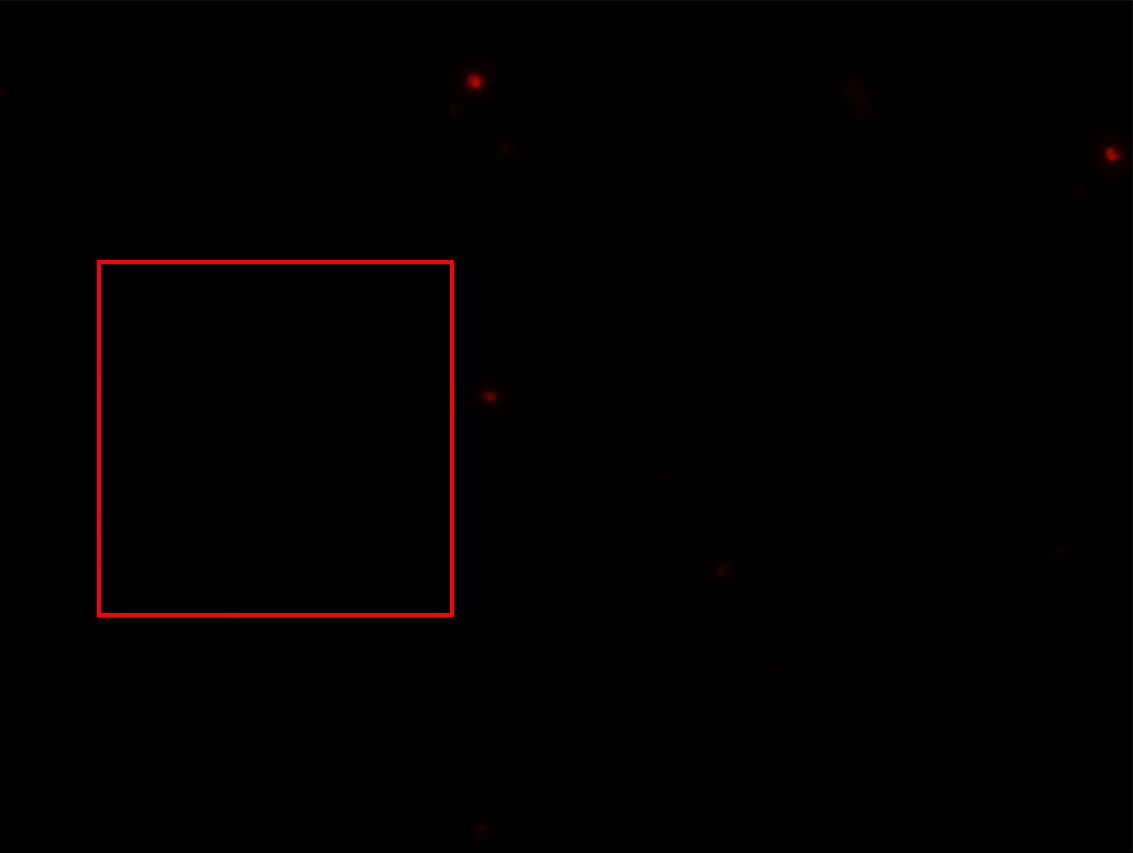

Supplement: Supplementary file 9 — Source Data for Figure 7 [file EMMM-15-e17230-s008.zip › Figure 7/7H/PI staining for si-NC-Vehicle.tif]

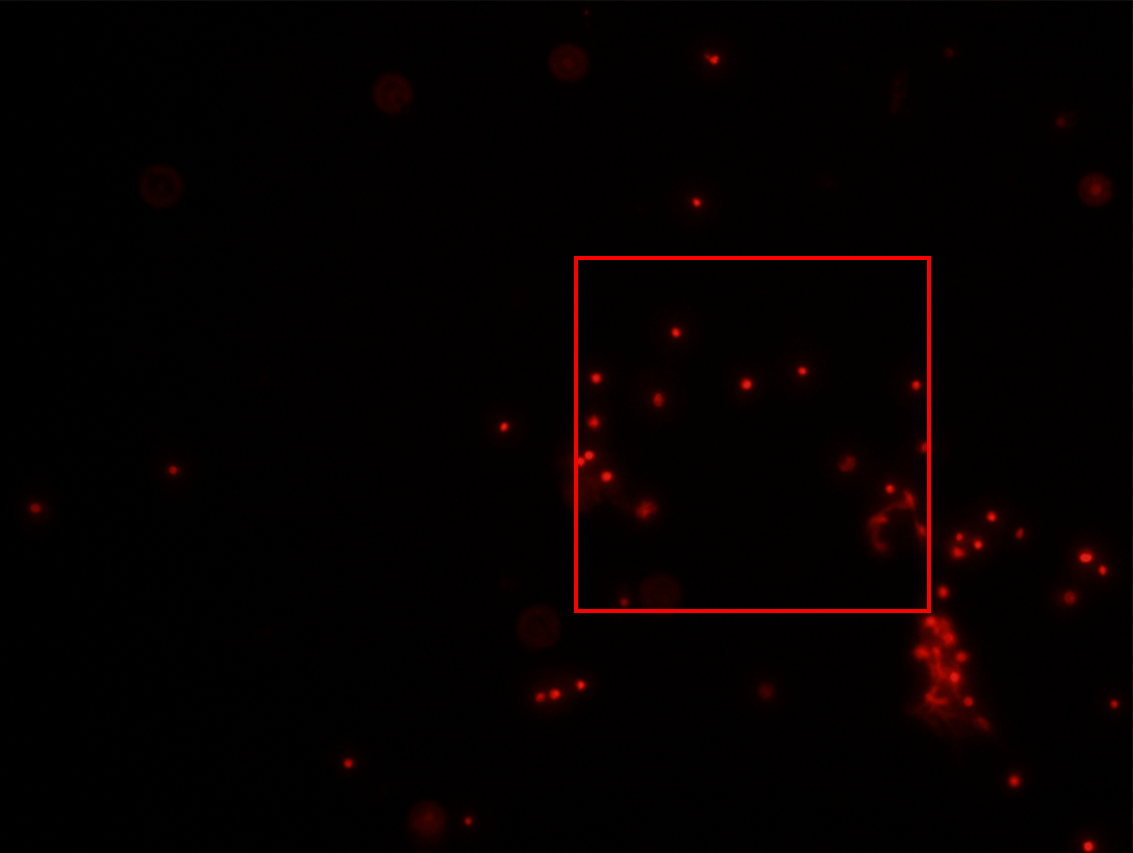

Supplement: Supplementary file 9 — Source Data for Figure 7 [file EMMM-15-e17230-s008.zip › Figure 7/7H/PI staining for si-NC-pIC+zVAD.tif]

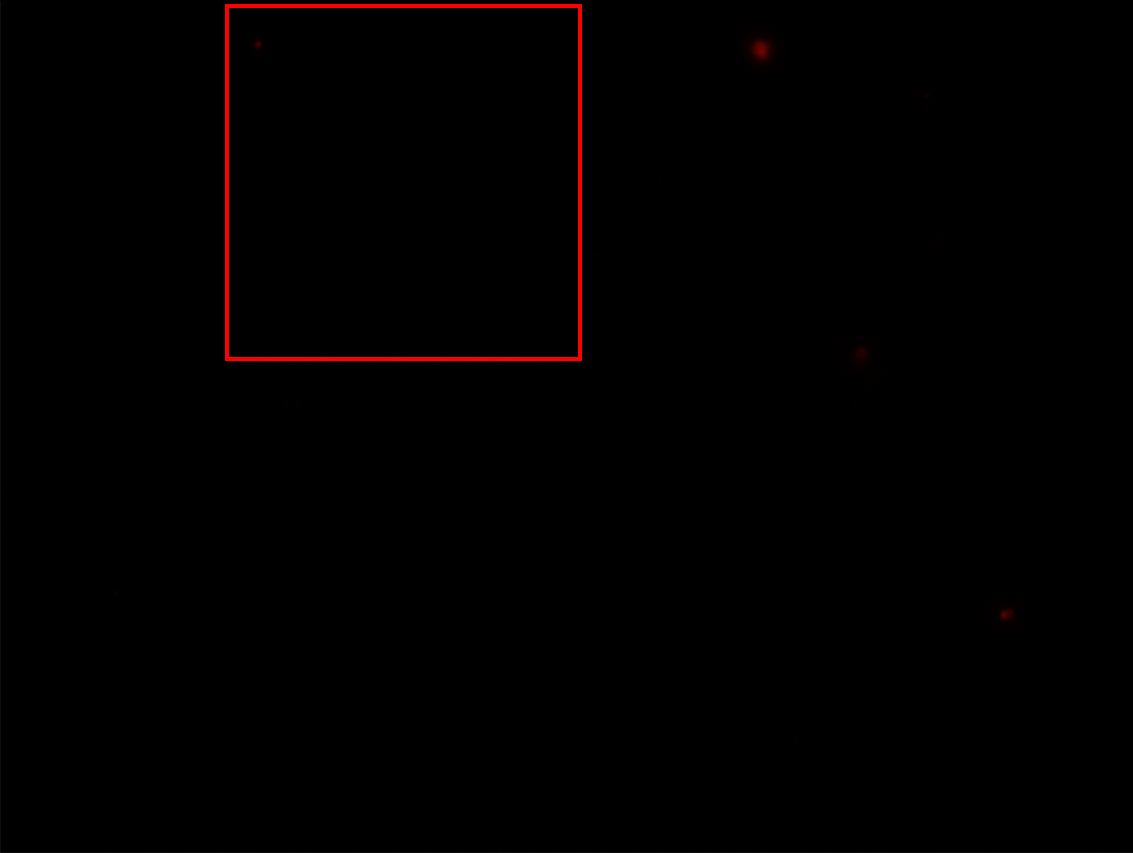

Supplement: Supplementary file 9 — Source Data for Figure 7 [file EMMM-15-e17230-s008.zip › Figure 7/7H/PI staining for si-Tbk1-Vehicle.tif]

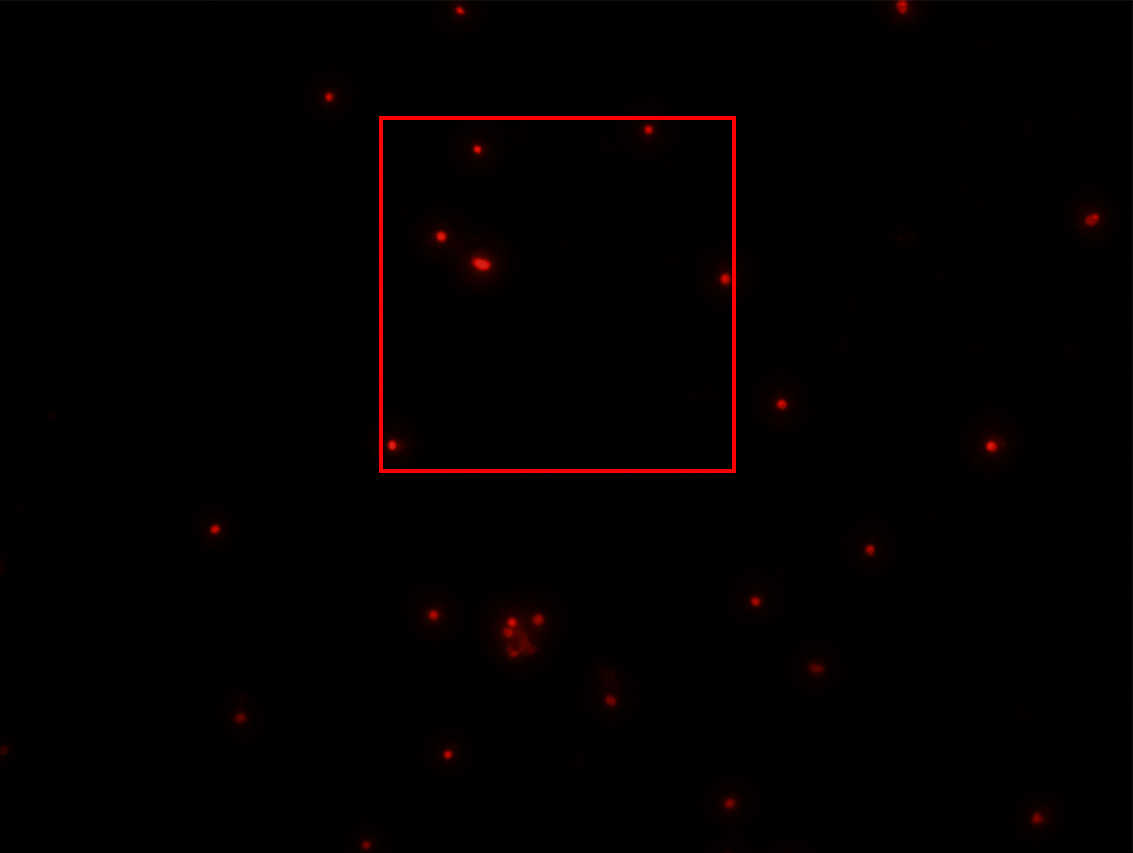

Supplement: Supplementary file 9 — Source Data for Figure 7 [file EMMM-15-e17230-s008.zip › Figure 7/7H/PI staining for si-Tbk1-pIC+zVAD.tif]

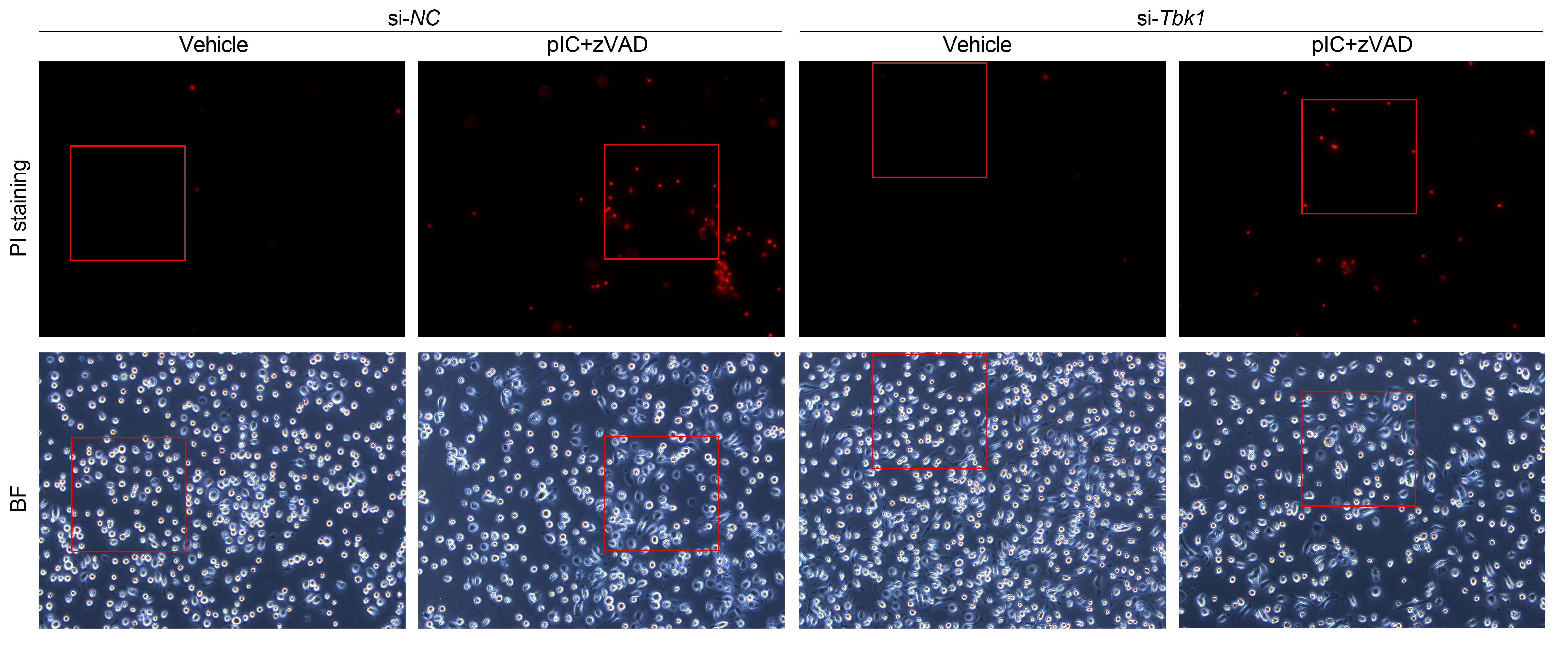

Supplement: Supplementary file 9 — Source Data for Figure 7 [file EMMM-15-e17230-s008.zip › Figure 7/7H/PI staining.tif]

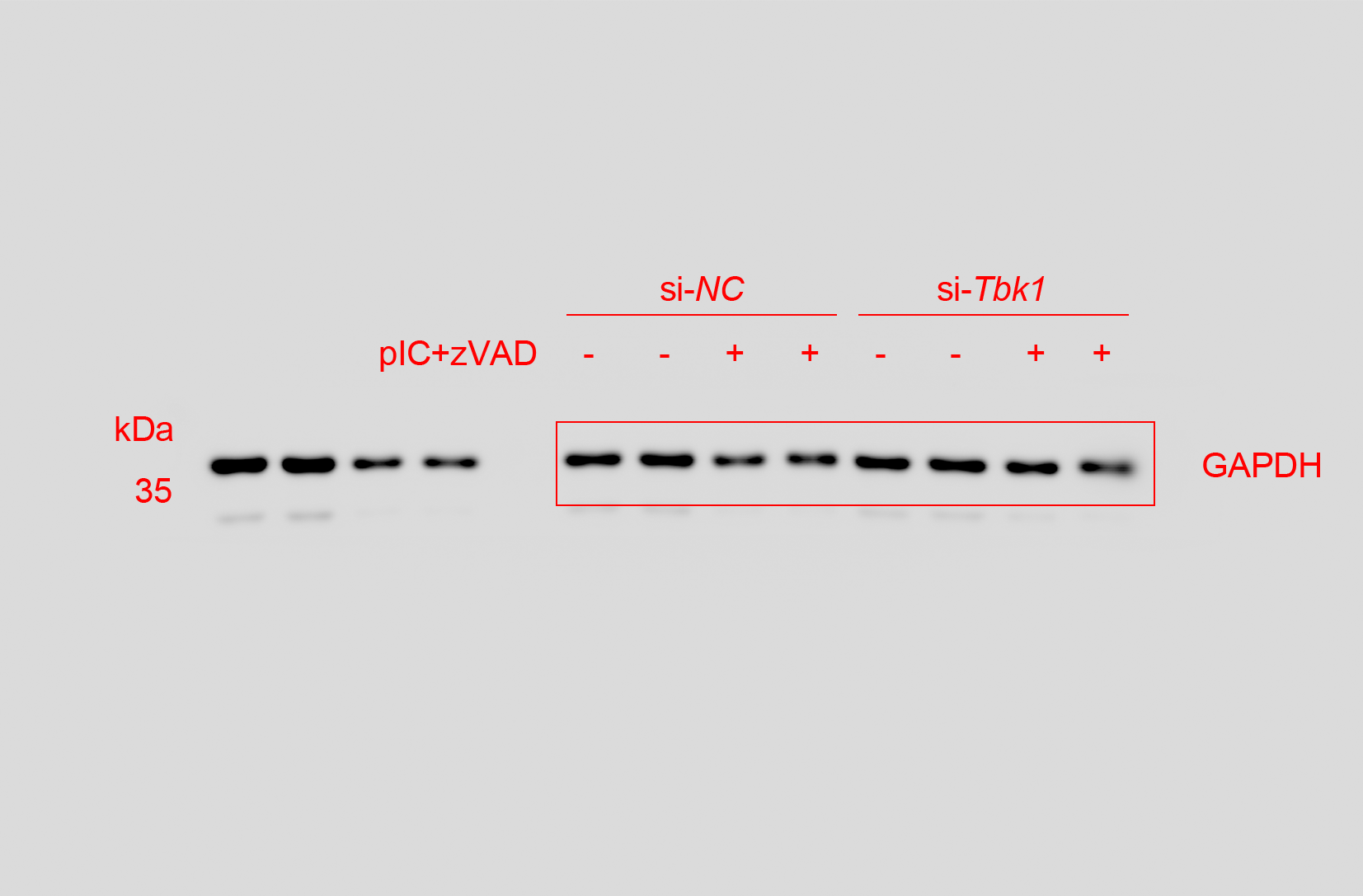

Supplement: Supplementary file 9 — Source Data for Figure 7 [file EMMM-15-e17230-s008.zip › Figure 7/7I/Western/GAPDH.tif]

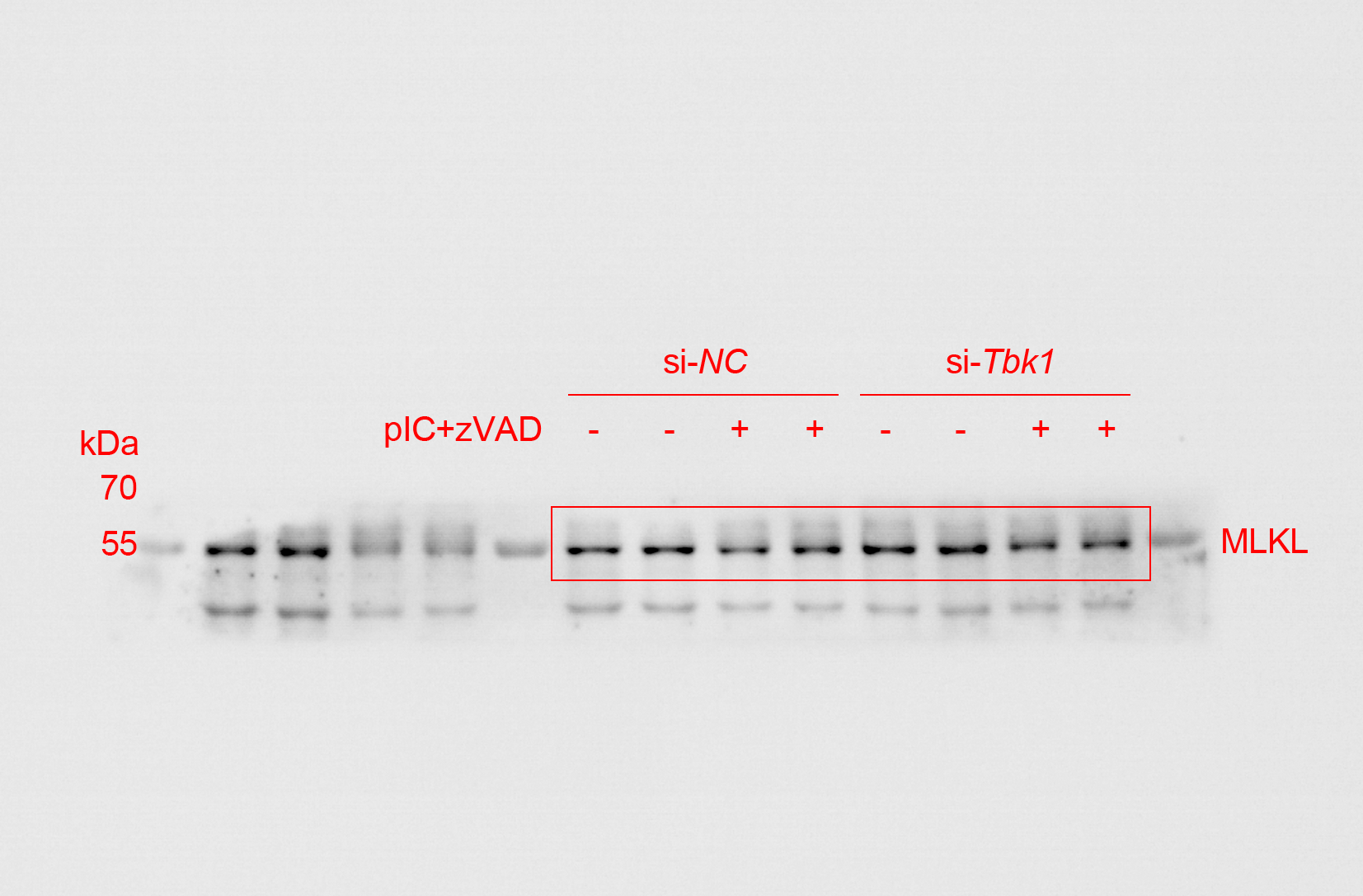

Supplement: Supplementary file 9 — Source Data for Figure 7 [file EMMM-15-e17230-s008.zip › Figure 7/7I/Western/MLKL.tif]

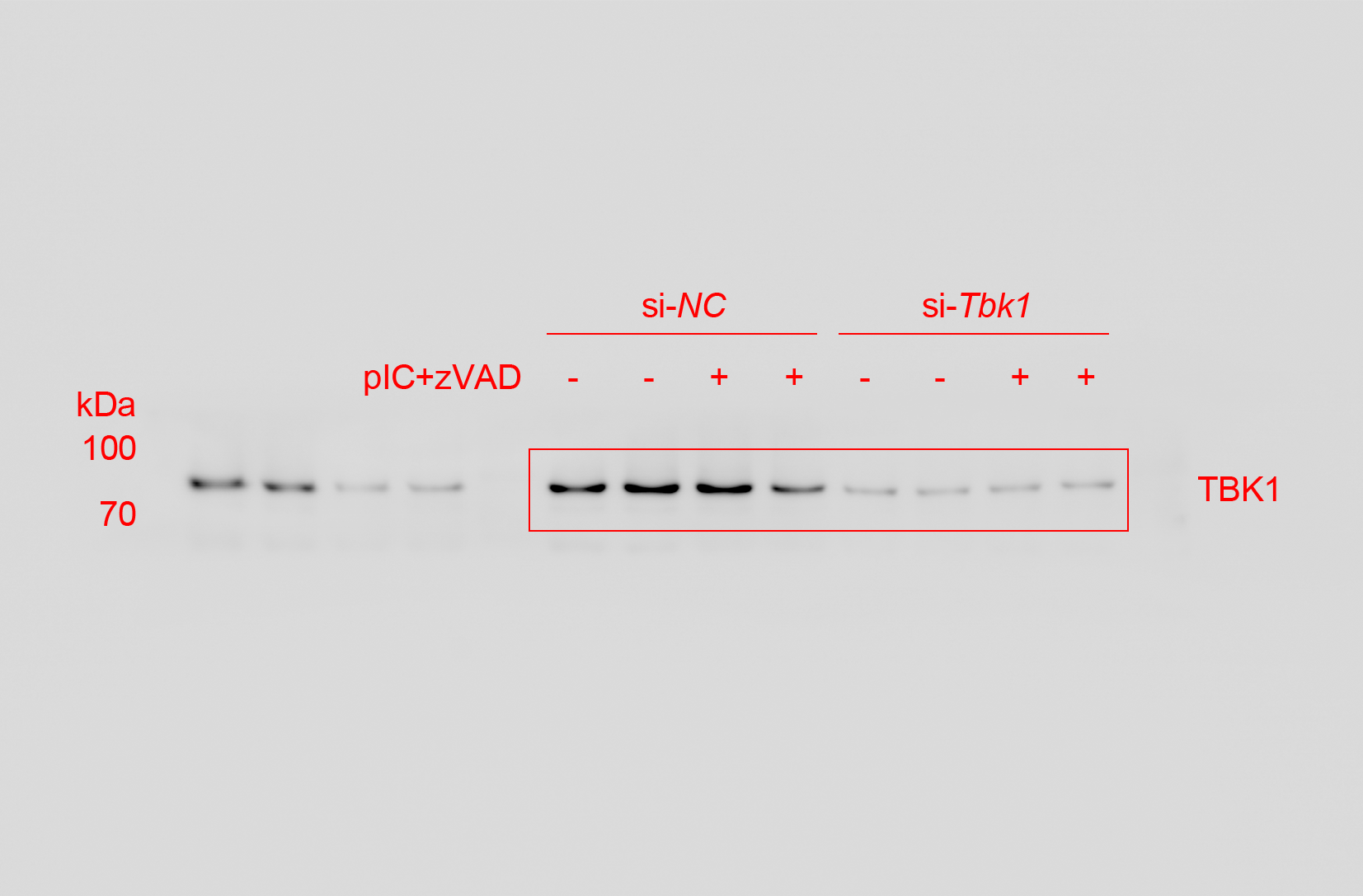

Supplement: Supplementary file 9 — Source Data for Figure 7 [file EMMM-15-e17230-s008.zip › Figure 7/7I/Western/TBK1.tif]

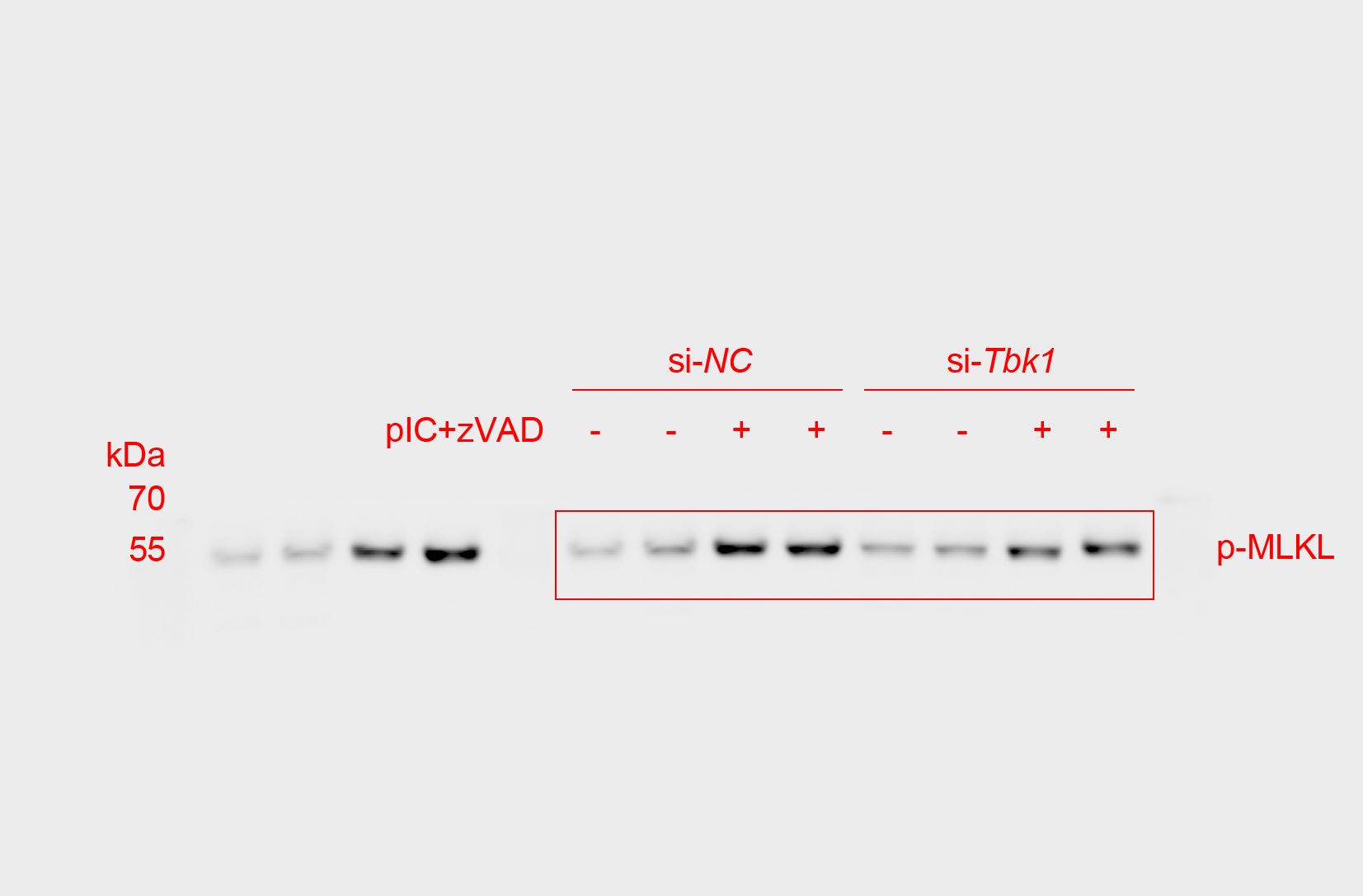

Supplement: Supplementary file 9 — Source Data for Figure 7 [file EMMM-15-e17230-s008.zip › Figure 7/7I/Western/p-MLKL.tif]

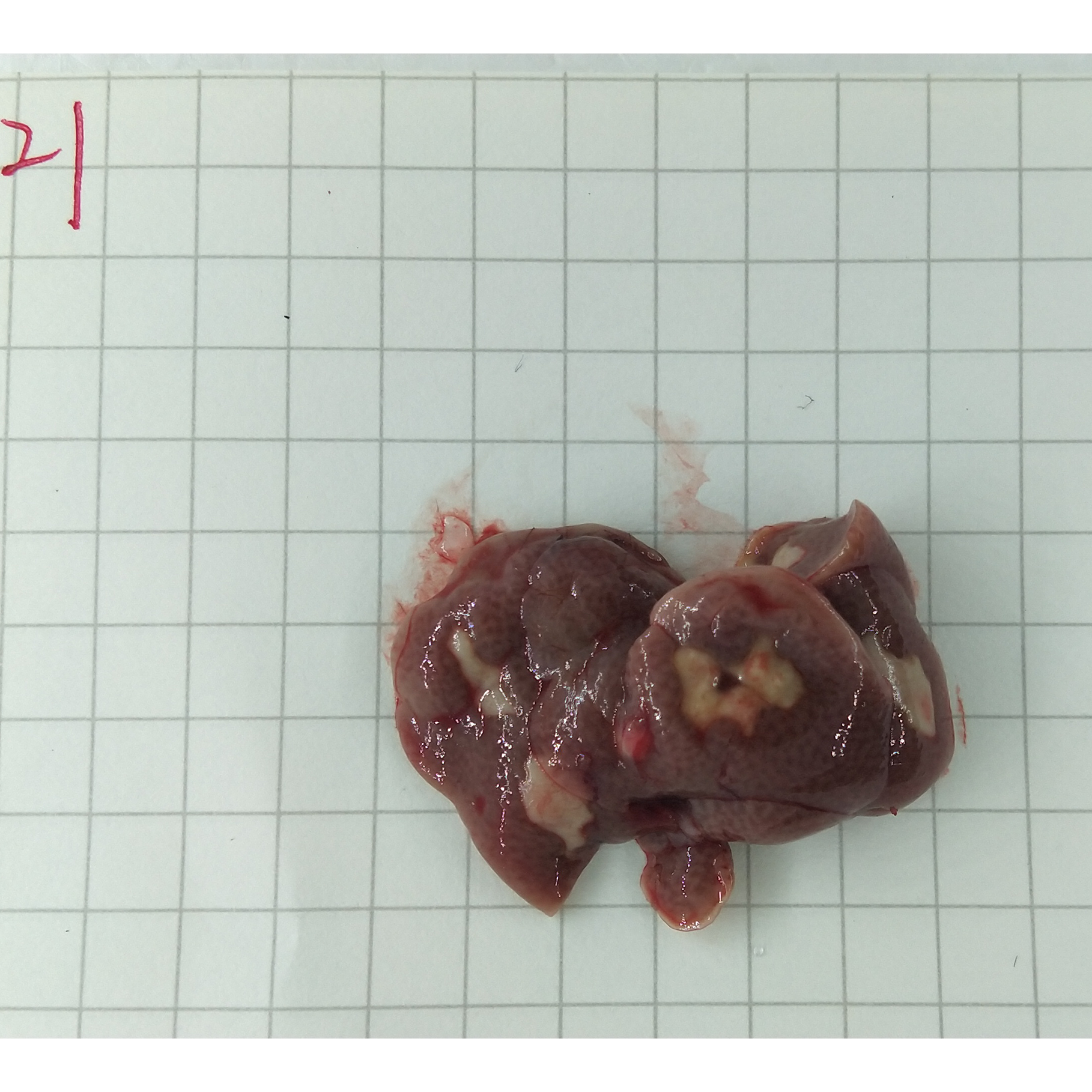

Supplement: Supplementary file 9 — Source Data for Figure 7 [file EMMM-15-e17230-s008.zip › Figure 7/7P/DB-rAAV-si-NC-Liver.tif]

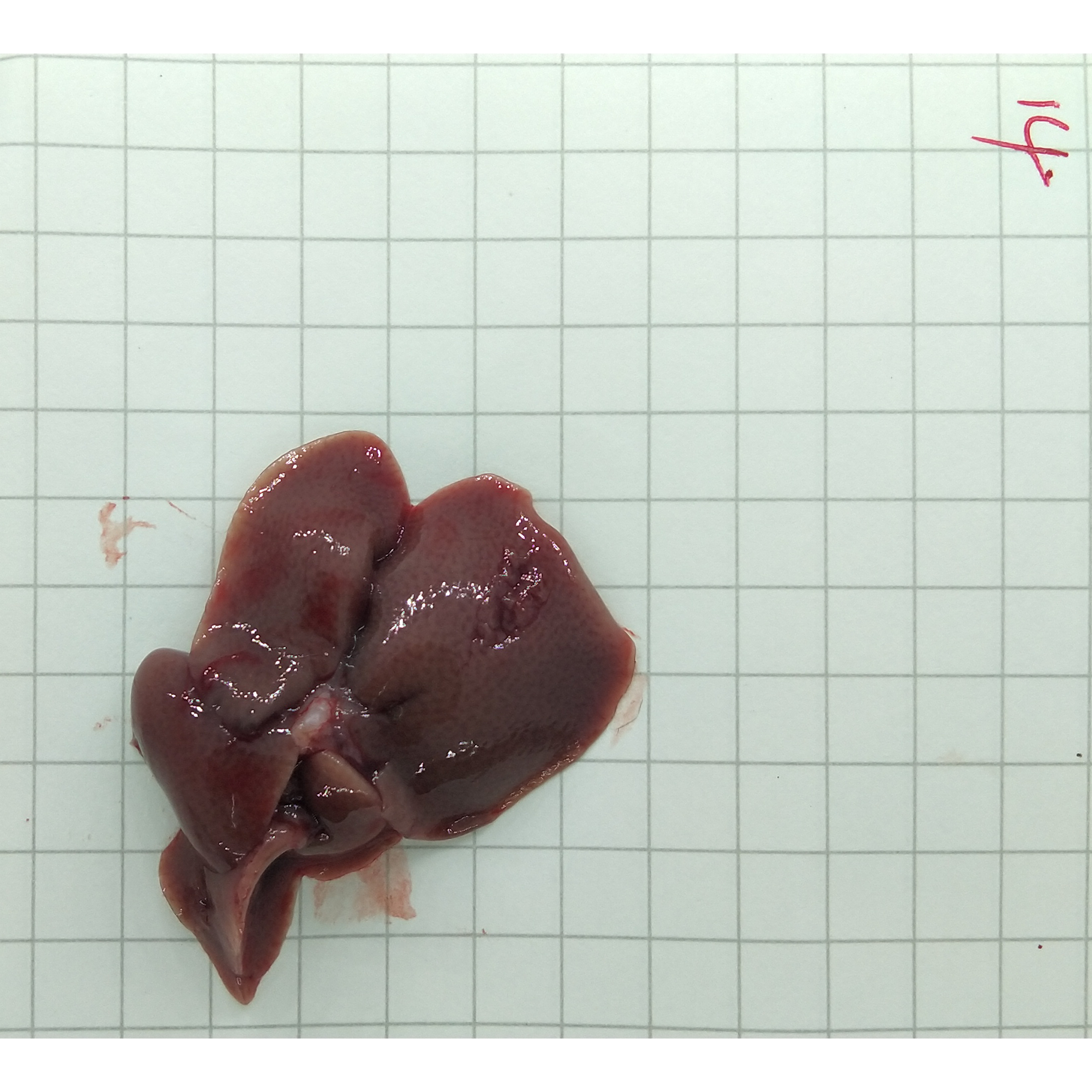

Supplement: Supplementary file 9 — Source Data for Figure 7 [file EMMM-15-e17230-s008.zip › Figure 7/7P/DB-rAAV-si-Tbk1-Liver.tif]

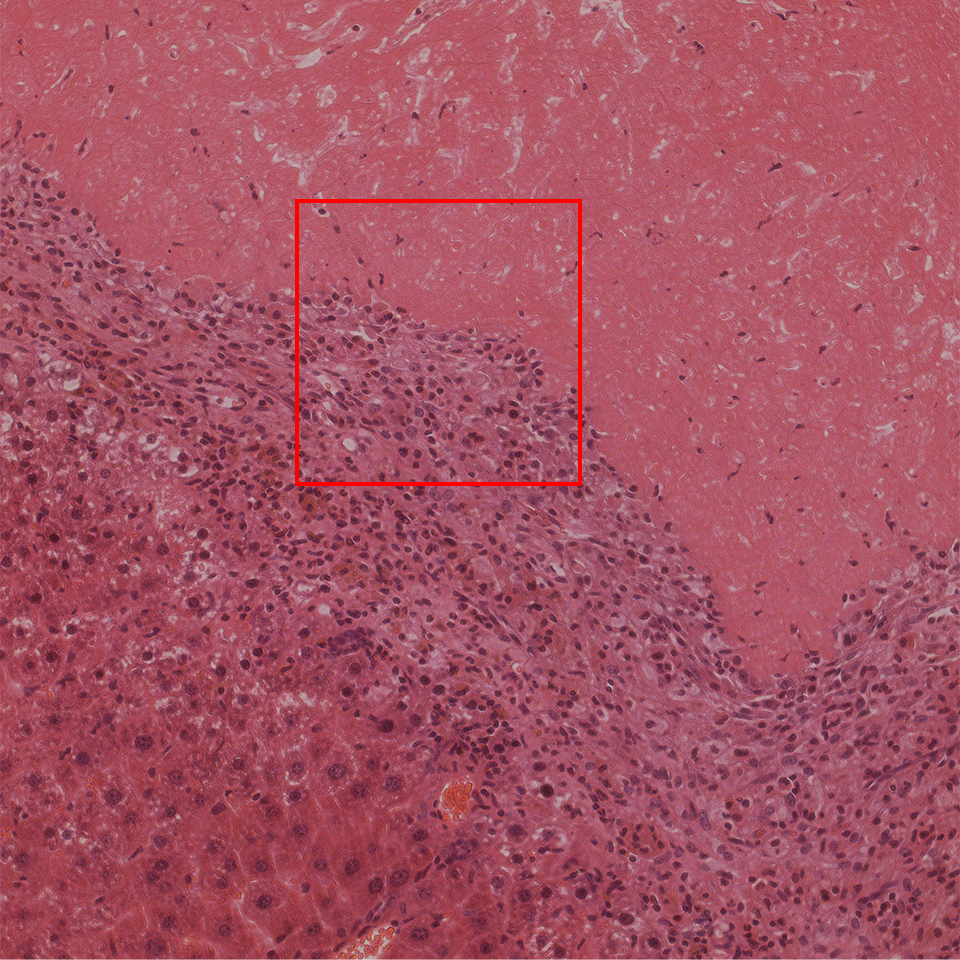

Supplement: Supplementary file 9 — Source Data for Figure 7 [file EMMM-15-e17230-s008.zip › Figure 7/7P/H&E for DB-rAAV-si-NC.tif]

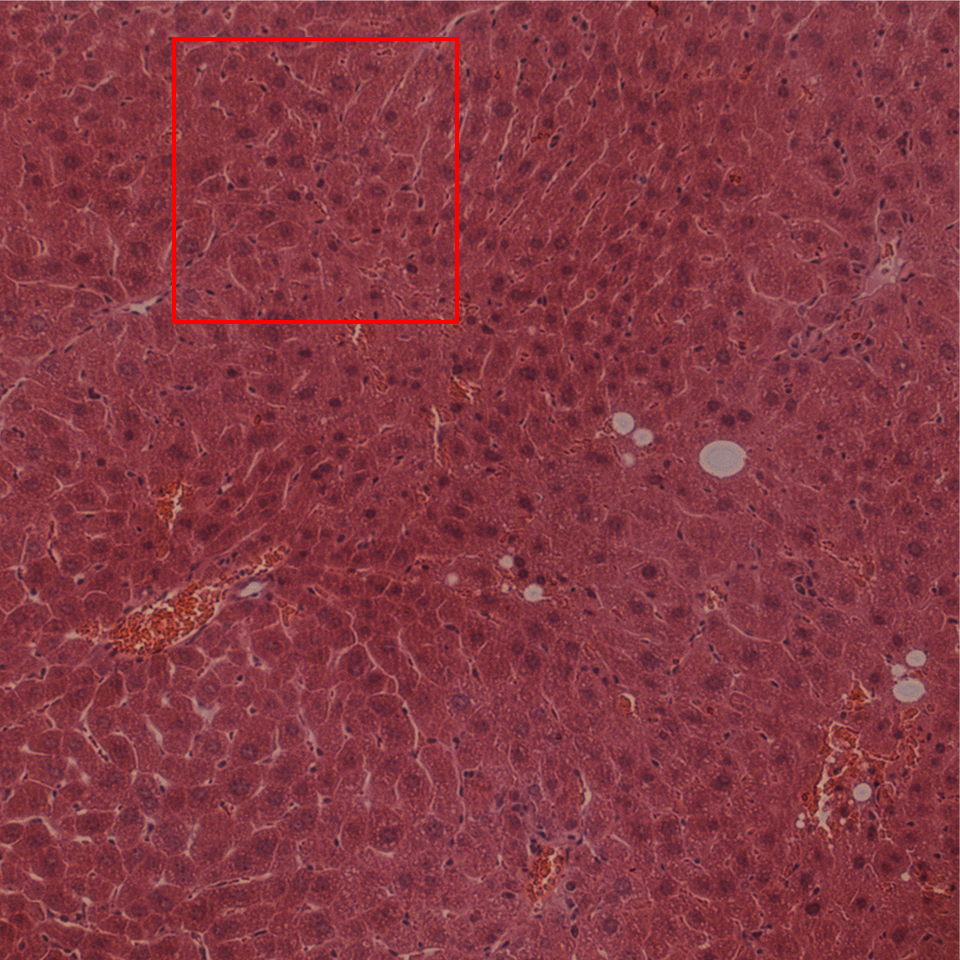

Supplement: Supplementary file 9 — Source Data for Figure 7 [file EMMM-15-e17230-s008.zip › Figure 7/7P/H&E for DB-rAAV-si-Tbk1.tif]

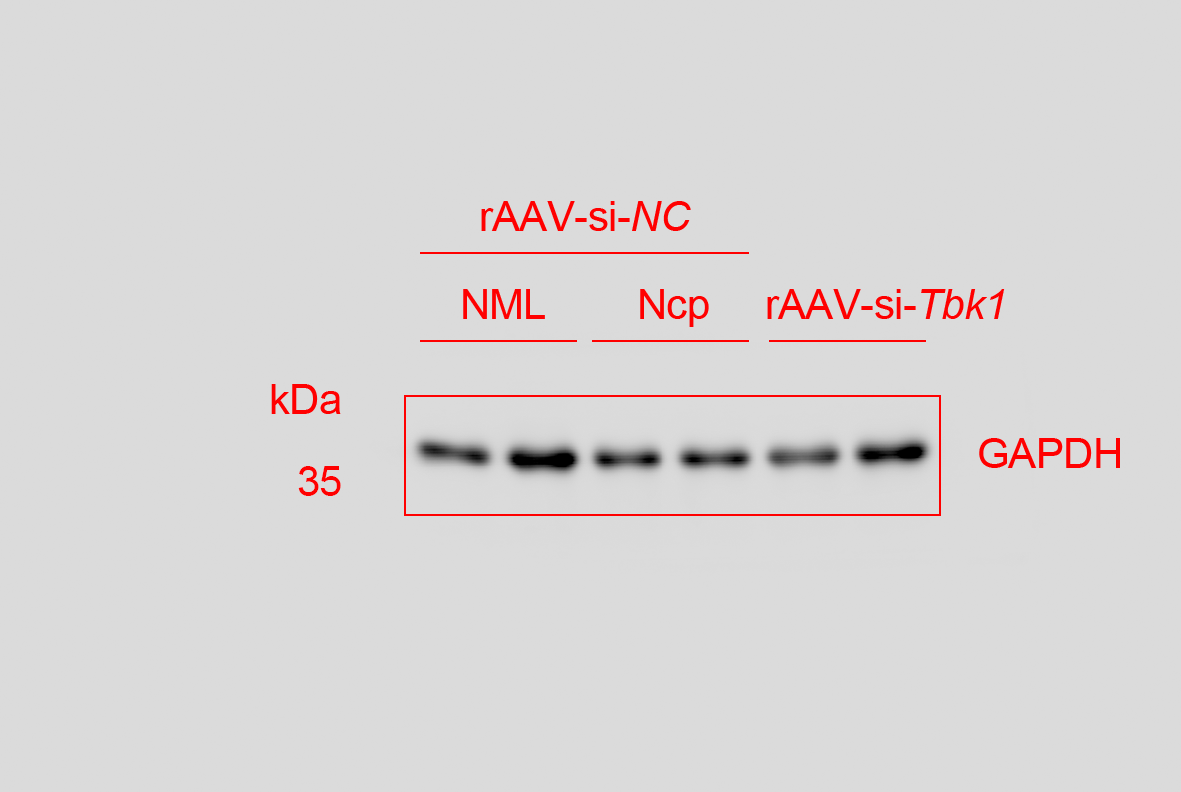

Supplement: Supplementary file 9 — Source Data for Figure 7 [file EMMM-15-e17230-s008.zip › Figure 7/7R/Western/GAPDH.tif]

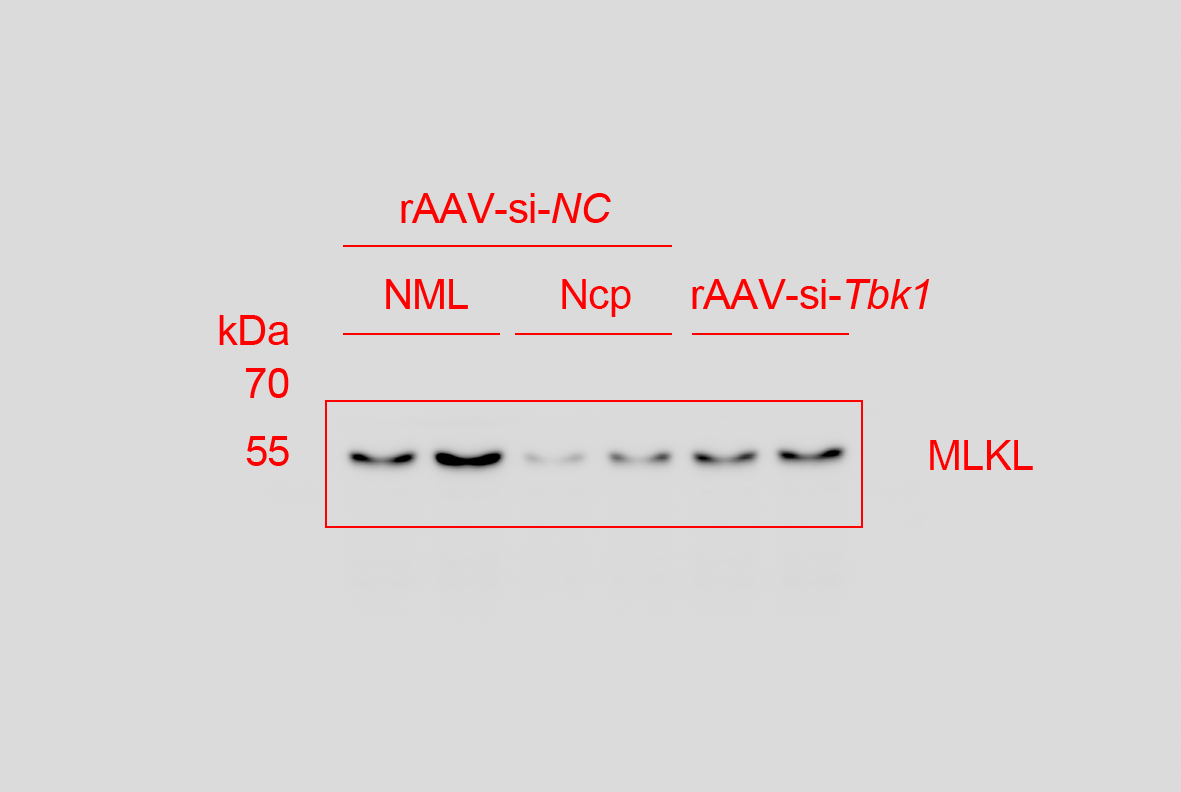

Supplement: Supplementary file 9 — Source Data for Figure 7 [file EMMM-15-e17230-s008.zip › Figure 7/7R/Western/MLKL.tif]

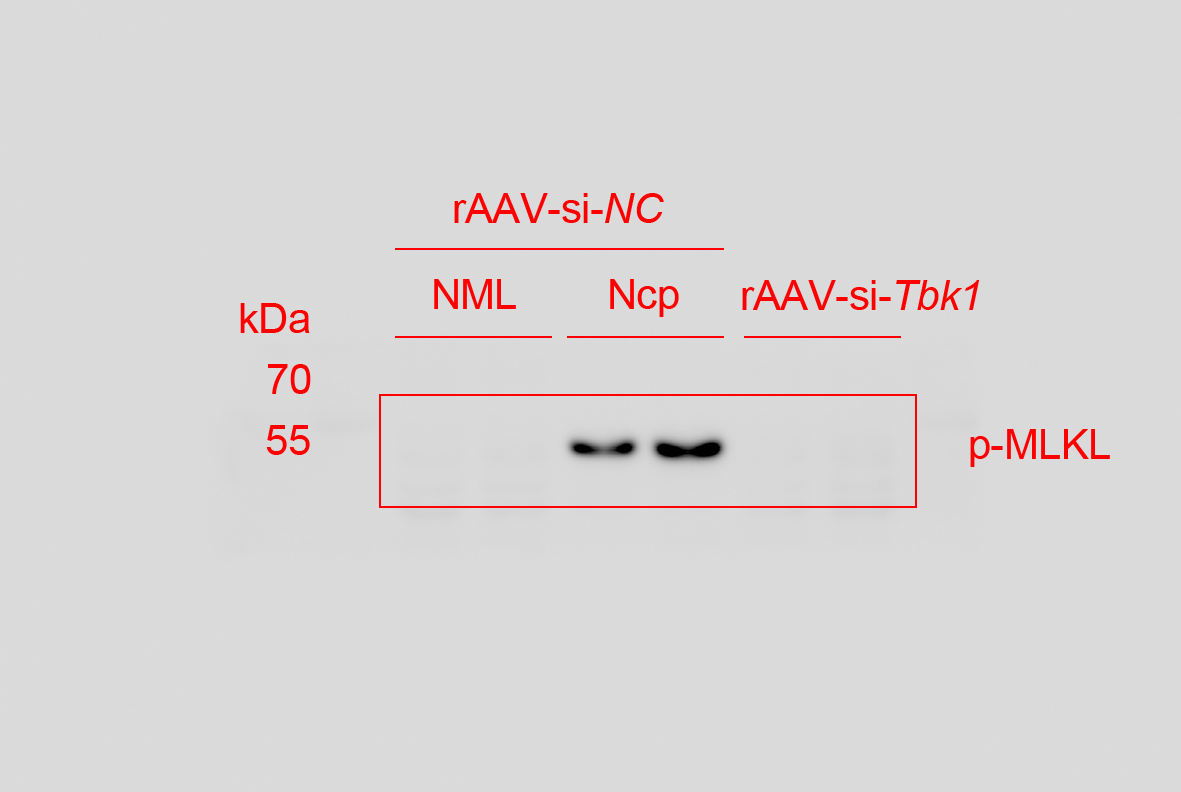

Supplement: Supplementary file 9 — Source Data for Figure 7 [file EMMM-15-e17230-s008.zip › Figure 7/7R/Western/p-MLKL.tif]
