# Supplementary material for: AAV induces hepatic necroptosis and carcinoma in diabetic and obese mice dependent on Pebp1 pathway
Source: EMBO Mol Med. 2023 Jun 5;15(7):e17230. doi: 10.15252/emmm.202217230 (PMC10331584; doi:10.15252/emmm.202217230)
Supplement: Supplementary file 10 [file EMMM-15-e17230-s006.pdf]

EMBO Molecular Medicine

AAV induces hepatic necroptosis and carcinoma in diabetic and obese mice dependent on Pebp1 pathway

Yalan Cheng, Zhentong Zhang , Peidong Gao, Hejin Lai, Wuling Zhong, Ning Feng, Yale Yang, Huimin Yu, Yali Zhang, Yumo Han, Jieya Dong, Zhishui He, Rui Huang and Qiwei Zhai
DOI: 10.15252/emmm.202217230

Corresponding author(s): Qiwei Zhai (qwzhai@sibs.ac.cn)

Review Timeline:

Submission Date:	30th Nov 22
Editorial Decision:	16th Jan 23
Revision Received:	23rd Apr 23
Editorial Decision:	5th May 23
Revision Received:	8th May 23
Accepted:	9th May 23

Editor: Lise Roth

Transaction Report:

(Note: With the exception of the correction of typographical or spelling errors that could be a source of ambiguity, letters and reports are not edited. Depending on transfer agreements, referee reports obtained elsewhere may or may not be included in this compilation. Referee reports are anonymous unless the Referee chooses to sign their reports.)

16th Jan 2023

Dear Prof. Zhai,

Thank you for the submission of your manuscript to EMBO Molecular Medicine, and please accept my apologies for the delay in getting back to you as we were waiting for one referee report. However, given that referee #1 has not yet gotten back to use despite several chasers, and that both referees #2 and #3 provide similar recommendations, we prefer to make a decision now in order to avoid further delay in the process. Should referee #1 provide a report, we will send it to you, with the understanding that we will not ask you extensive experiments in addition to the ones required in the enclosed reports from referee #2 and #3.

As you will see from the reports below, the referees acknowledge the interest of the study and are supporting publication of your work pending appropriate revisions.

Addressing the reviewers' concerns in full will be necessary for further considering the manuscript in our journal, and acceptance of the manuscript will entail a second round of review. EMBO Molecular Medicine encourages a single round of revision only and therefore, acceptance or rejection of the manuscript will depend on the completeness of your responses included in the next, final version of the manuscript. For this reason, and to save you from any frustrations in the end, I would strongly advise against returning an incomplete revision.

Revised manuscripts should be submitted within three months of a request for revision; they will otherwise be treated as new submissions, except under exceptional circumstances in which a short extension is obtained from the editor.

When submitting your revised manuscript, please carefully review the instructions that follow below. We perform an initial quality control of all revised manuscripts before re-review; failure to include requested items will delay the evaluation of your revision.

We require:

1) A .docx formatted version of the manuscript text (including legends for main figures, EV figures and tables). Please make sure that the changes are highlighted to be clearly visible.

2) Individual production quality figure files as .eps, .tif, .jpg (one file per figure). For guidance, download the 'Figure Guide PDF' (https://www.embopress.org/page/journal/17574684/authorguide#figureformat).

3) At EMBO Press we ask authors to provide source data for the main figures. Our source data coordinator will contact you to discuss which figure panels we would need source data for and will also provide you with helpful tips on how to upload and organize the files.

4) A .docx formatted letter INCLUDING the reviewers' reports and your detailed point-by-point responses to their comments. As part of the EMBO Press transparent editorial process, the point-by-point response is part of the Review Process File (RPF), which will be published alongside your paper.

5) A complete author checklist, which you can download from our author guidelines (https://www.embopress.org/page/journal/17574684/authorguide#submissionofrevisions). Please insert information in the checklist that is also reflected in the manuscript. The completed author checklist will also be part of the RPF.

6) Please note that all corresponding authors are required to supply an ORCID ID for their name upon submission of a revised manuscript.

7) It is mandatory to include a 'Data Availability' section after the Materials and Methods. Before submitting your revision, primary datasets produced in this study need to be deposited in an appropriate public database, and the accession numbers and database listed under 'Data Availability'. Please remember to provide a reviewer password if the datasets are not yet public (see https://www.embopress.org/page/journal/17574684/authorguide#dataavailability).

In case you have no data that requires deposition in a public database, please state so in this section. Note that the Data Availability Section is restricted to new primary data that are part of this study.

8) For data quantification: please specify the name of the statistical test used to generate error bars and P values, the number (n) of independent experiments (specify technical or biological replicates) underlying each data point and the test used to calculate p-values in each figure legend. The figure legends should contain a basic description of n, P and the test applied. Graphs must include a description of the bars and the error bars (s.d., s.e.m.). Please provide exact p values.

9) Our journal encourages inclusion of *data citations in the reference list* to directly cite datasets that were re-used and obtained from public databases. Data citations in the article text are distinct from normal bibliographical citations and should directly link to the database records from which the data can be accessed. In the main text, data citations are formatted as follows: "Data ref: Smith et al, 2001" or "Data ref: NCBI Sequence Read Archive PRJNA342805, 2017". In the Reference list, data citations must be labeled with "[DATASET]". A data reference must provide the database name, accession number/identifiers and a resolvable link to the landing page from which the data can be accessed at the end of the reference. Further instructions are available at .

10) We replaced Supplementary Information with Expanded View (EV) Figures and Tables that are collapsible/expandable online. A maximum of 5 EV Figures can be typeset. EV Figures should be cited as 'Figure EV1, Figure EV2" etc... in the text and their respective legends should be included in the main text after the legends of regular figures.
- For the figures that you do NOT wish to display as Expanded View figures, they should be bundled together with their legends in a single PDF file called *Appendix*, which should start with a short Table of Content. Appendix figures should be referred to in the main text as: "Appendix Figure S1, Appendix Figure S2" etc.
- Additional Tables/Datasets should be labeled and referred to as Table EV1, Dataset EV1, etc. Legends have to be provided in a separate tab in case of .xls files. Alternatively, the legend can be supplied as a separate text file (README) and zipped together with the Table/Dataset file.
See detailed instructions here:

11) The paper explained: EMBO Molecular Medicine articles are accompanied by a summary of the articles to emphasize the major findings in the paper and their medical implications for the non-specialist reader. Please provide a draft summary of your article highlighting
- the medical issue you are addressing,
- the results obtained and
- their clinical impact.
This may be edited to ensure that readers understand the significance and context of the research. Please refer to any of our published articles for an example.

12) For more information: There is space at the end of each article to list relevant web links for further consultation by our readers. Could you identify some relevant ones and provide such information as well? Some examples are patient associations, relevant databases, OMIM/proteins/genes links, author's websites, etc...

13) Author contributions: CRediT has replaced the traditional author contributions section because it offers a systematic machine readable author contributions format that allows for more effective research assessment. Please remove the Authors Contributions from the manuscript and use the free text boxes beneath each contributing author's name in our system to add specific details on the author's contribution. More information is available in our guide to authors.

14) Disclosure statement and competing interests: We updated our journal's competing interests policy in January 2022 and request authors to consider both actual and perceived competing interests. Please review the policy https://www.embopress.org/competing-interests and update your competing interests if necessary.

15) Every published paper now includes a 'Synopsis' to further enhance discoverability. Synopses are displayed on the journal webpage and are freely accessible to all readers. They include a short stand first (maximum of 300 characters, including space) as well as 2-5 one-sentences bullet points that summarizes the paper. Please write the bullet points to summarize the key NEW findings. They should be designed to be complementary to the abstract - i.e. not repeat the same text. We encourage inclusion of key acronyms and quantitative information (maximum of 30 words / bullet point). Please use the passive voice. Please attach these in a separate file or send them by email, we will incorporate them accordingly.

Please also suggest a striking image or visual abstract to illustrate your article as a PNG file 550 px wide x 300-600 px high.

16) As part of the EMBO Publications transparent editorial process initiative (see our Editorial at http://embomolmed.embopress.org/content/2/9/329), EMBO Molecular Medicine will publish online a Review Process File (RPF) to accompany accepted manuscripts.
In the event of acceptance, this file will be published in conjunction with your paper and will include the anonymous referee reports, your point-by-point response and all pertinent correspondence relating to the manuscript. Let us know whether you agree with the publication of the RPF and as here, if you want to remove or not any figures from it prior to publication.
Please note that the Authors checklist will be published at the end of the RPF.

EMBO Molecular Medicine has a "scooping protection" policy, whereby similar findings that are published by others during review or revision are not a criterion for rejection. Should you decide to submit a revised version, I do ask that you get in touch after three months if you have not completed it, to update us on the status.

I look forward to receiving your revised manuscript.

Yours sincerely,

Lise Roth

Lise Roth, PhD
Senior Editor
EMBO Molecular Medicine

Please note: When submitting your revision you will be prompted to enter your funding and payment information. This will allow Wiley to send you a quote for the article processing charge (APC) in case of acceptance. This quote takes into account any reduction or fee waivers that you may be eligible for. Authors do not need to pay any fees before their manuscript is accepted and transferred to the publisher.

EMBO Press participates in many Publish and Read agreements that allow authors to publish Open Access with reduced/no publication charges. Check your eligibility: https://authorservices.wiley.com/author-resources/Journal-Authors/open-access/affiliation-policies-payments/index.html

***** Reviewer's comments *****

Referee #2 (Comments on Novelty/Model System for Author):

The authors report that the recombinant AAV injection caused liver injury, hepatic necroptosis and HCC in db/db or high-fat diet-induced hyperglycemic and obese mice. Prednisone administration or knockdown of Pebp1 alleviated hepatic injury and necroptosis induced by recombinant AAV. Pebp1 acted through Tbk1 aactiivation in a setting simulating viral infection. Necroptosis and tumorigenesis did not ocur in diabetic only or obese only mice that received recombinant AAV particles. Authors concluded that their findings show that AAV infection is a critical risk factor for HCC in patients with diabetes and obesity, and AAV gene therapy for these patients should be carefully evaluated. They also propose that prednisone treatment or Pebp1 pathway targeting are promising strategies to alleviate inflammation and necroptosis occurred in AAV gene therapy or related diseases. The manuscript results are presented in a straight forward manner with well explained and well presented data. All key data was obtained in vivo in mice and some confirmatory data was obtained in vitro using primary cell culture. The English text of the manuscript would be much better after professional editing.

Referee #2 (Remarks for Author):

The manuscript results are presented in a straight forward manner with well explained and well presented data. All key data was obtained in vivo in mice and some confirmatory data was obtained in vitro using primary cell culture. The English text of the manuscript would be much better after professional editing.

Referee #3 (Comments on Novelty/Model System for Author):

The in vitro model could be improved by demonstrating that the same effects shown in Fig. 7A-I for mouse macrophages take place also in human macrophages.

Referee #3 (Remarks for Author):

The article is well written, experiments are well performed, and conclusions are mainly supported by the obtained results. Here are some critical comments:

Major comments:

1. In the text describing Fig. 1Q (section Results, 2.1.) it is written that "The db/db mice injected with rAAV showed decreased body weight" (as it is shown in the Fig. 1Q); however, in the legend to Fig. 1Q, it is written that "Six months after a single injection of rAAV, db/db mice showed similar body weight (Q)" - in contrast to the Fig. 1Q.
2. Injection of rAAV into hyperglycemic mice causes a 2-fold increase of liver weight (Fig. 2E), and histology of such liver (Fig.

2I) differs significantly from that of control (may be, glycogen deposition?). What causes these changes - does not discussed. Could high ALT and AST levels (Fig. 2G, H) be caused by other forms of hepatocyte death (and not by necroptosis)?
3. In the Results 2.5., it is described that three genes - Cyp4a14, Pebp1 and Tat - were chosen for validation by RT-PCR. However, it is not explained - how these genes were selected among the top 15 genes shown in Fig. 5A.
4. In the legend to Fig. 5L it is mentioned that "hepatic mRNA levels of mice in (D)"; probably, there should be (K) instead of (D)?
5. In the Fig. 6D,E it can be seen that the control group is divided to two subgroups: one, having an increased ALT & AST levels (are there the same mice having high ALT & AST?), and another one, having the same ALT & AST levels as mice injected with rAAV-si-Pebp1. However, this is not discussed. But then it is not clear - which mice are shown in the Fig. 6F, G & H: whether mice from both control subgroups behave similarly in the tests shown in the Fig. 6F, G & H? Whether the endogenous Pebp1 level was similar between these two subgroups of control mice?
6. All experiments of the section 2.7 of "Results" (shown in Fig. 7A-I) were performed in mouse primary macrophages. Because the authors of the study make recommendations for treatment of patients, it seems reasonable to demonstrate that the same effects take place also in human macrophages.

Minor comments:
1. Typos: p.9, lane 9 (Results, 2.4): "... we first investigated" ("d" is omitted); p.10, lane 18: "While..." is unnecessary, should be removed; p.12, lane 3: after "inflammation" there are should be "." instead of ",".

Point-by-point Responses to Referees

***** Reviewer's comments *****

Referee #2 (Comments on Novelty/Model System for Author):

The authors report that the recombinant AAV injection caused liver injury, hepatic necroptosis and HCC in db/db or high-fat diet-induced hyperglycemic and obese mice. Prednisone administration or knockdown of Pebp1 alleviated hepatic injury and necroptosis induced by recombinant AAV. Pebp1 acted through Tbk1 aactiivation in a setting simulating viral infection. Necroptosis and tumorigenesis did not ocur in diabetic only or obese only mice that received recombinant AAV particles. Authors concluded that their findings show that AAV infection is a critical risk factor for HCC in patients with diabetes and obesity, and AAV gene therapy for these patients should be carefully evaluated. They also propose that prednisone treatment or Pebp1 pathway targeting are promising strategies to alleviate inflammation and necroptosis occurred in AAV gene therapy or related diseases. The manuscript results are presented in a straight forward manner with well explained and well presented data. All key data was obtained in vivo in mice and some confirmatory data was obtained in vitro using primary cell culture. The English text of the manuscript would be much better after professional editing.

Response:

We really appreciate Referee #2's suggestion for the professional editing of manuscript. According to Referee #2's suggestion, we have carefully checked and edited the English text in the revised manuscript.

Referee #2 (Remarks for Author):

The manuscript results are presented in a straight forward manner with well explained and well presented data. All key data was obtained in vivo in mice and some confirmatory data was obtained in vitro using primary cell culture. The English text of the manuscript would be much better after professional editing.

Response:

According to Referee #2's suggestion, we have carefully checked and edited the English text in the revised manuscript as following.

1. "A prospective study showed that persons with anti-HCV positivity and anti-HBV negativity **had** a 20-fold increased risk of developing HCC" was replaced by "A prospective study showed that persons with anti-HCV positivity and anti-HBV negativity **have** a 20-fold increased risk of developing HCC" at line 18 on page 2 in Introduction.

2. "Adeno-associated virus (AAV) infection is about 30%–80% in human **population**" was replaced by "Adeno-associated virus (AAV) infection is about 30%–80% in human **populations**" at line 2 on page 3 in Introduction.

3. "Pebp1 was screened **to investigate** its role in inflammation and necroptosis in vivo and in vitro" Was replaced by "Pebp1 was screened**, and we investigated** its role in inflammation and necroptosis in vivo and in vitro" at line 8 on page 4 in Introduction.

4. "We next analyzed gene expression profiles of normal and necroptotic **area** from livers of *db/db* mice after a single injection of PBS or rAAV for 2 months." was replaced by "We next analyzed gene expression profiles of normal and necroptotic **areas** from livers of *db/db* mice after a single injection of PBS or rAAV for 2 months." at line 18-19 on page 5 in Result.

5. "Taken together, **either** hyperglycemia **or** obesity is **not sufficient** for rAAV-induced hepatic necroptosis in mice." was replaced by "Taken together, **neither** obesity **nor** hyperglycemia alone is **sufficient** for rAAV-induced hepatic necroptosis in mice." at line 3-4 on page 8 in Result.

6. “To investigate whether prednisone can also attenuate liver injury, hepatic necroptosis and carcinoma induced by rAAV injection in mice with hyperglycemia and obesity, we first **investigate** whether prednisone can alleviate inflammation and necroptosis in vitro.” was replaced by “To investigate whether prednisone can also attenuate liver injury, hepatic necroptosis and carcinoma induced by rAAV injection in mice with hyperglycemia and obesity, we first **investigated** whether prednisone can alleviate inflammation and necroptosis in vitro.” at line 11-13 on page 9 in Result.

7. “**While serum** ALT and AST activities were significantly decreased in *db/db* mice injected with rAAV-*si-Pebp1* compared with those injected with rAAV-*si-NC*, rAAV-*si-Cyp4a14* or rAAV-*si-Tat*” was replaced by “**Serum** ALT and AST activities were significantly decreased in *db/db* mice injected with rAAV-*si-Pebp1* compared with those injected with rAAV-*si-NC*, rAAV-*si-Cyp4a14* or rAAV-*si-Tat*” at line 7-9 on page 11 in Result.

8. “To investigate the role of Pebp1 in necroptosis pathway, we first analyzed the role of Pebp1 in inflammation, Poly(I:C) was used to mimic virus infection in cell model to induce inflammation as described previously” was replaced by “To investigate the role of Pebp1 in necroptosis pathway, we first analyzed the role of Pebp1 in inflammation. Poly(I:C) was used to mimic virus infection in cell model to induce inflammation as described previously” at line 14-15 on page 12 in Result.

9. “These evidences demonstrate that inhibition of Pebp1 pathway can also attenuate inflammation and necroptosis in vitro and in vivo” was replaced by “These evidences demonstrate that inhibition of Pebp1 pathway can also attenuate inflammation and necroptosis **both** in vitro and in vivo” at line 17-18 on page 13 in Result.

10. “Mouse peritoneal **macrophage** isolation, culture and treatment” was replaced by “Mouse peritoneal **macrophages** isolation, culture and treatment” at line 15 on Page 21 in Materials and Methods.

11. “(Q-R) Six months after a single injection of rAAV, *db/db* mice showed **similar** body weight (Q) and blood glucose levels (R) **with** the mice injected with PBS” was replaced by “(Q-R) Six months after a single injection of rAAV, *db/db* mice showed **decreased** body weight (Q) and **similar** blood glucose levels (R) **compared to** the mice injected with PBS.” at line 3-5 on page 30 in Figure legends.

Referee #3 (Comments on Novelty/Model System for Author):

The in vitro model could be improved by demonstrating that the same effects shown in Fig. 7A-I for mouse macrophages take place also in human macrophages.

Response:

We really appreciate Referee #3's constructive suggestion to show the effects of PEBP1 and TBK1 in human macrophages. We examined the roles of PEBP1 and TBK1 in THP-1 human macrophages, showed the data in Expanded View Figure 5, and added the corresponding description in Materials and Methods, Results and Figure Legends.

Referee #3 (Remarks for Author):

The article is well written, experiments are well performed, and conclusions are mainly supported by the obtained results. Here are some critical comments:

Major comments:

1. In the text describing Fig. 1Q (section Results, 2.1.) it is written that "The db/db mice injected with rAAV showed decreased body weight" (as it is shown in the Fig. 1Q); however, in the legend to Fig. 1Q, it is written that "Six months after a single injection of rAAV, db/db mice showed similar body weight (Q)" - in contrast to the Fig. 1Q.

Response:

We really appreciate Referee #3's comments for the mistake we made in the figure legend for Fig. 1Q. We replaced "Six months after a single injection of rAAV, *db/db* mice showed **similar** body weight (Q) and blood glucose levels (R) **with** the mice injected with PBS" with "Six months after a single injection of rAAV, *db/db* mice showed **decreased** body weight (Q) and **similar** blood glucose levels (R) **compared to** the mice injected with PBS" at line 3-5 on page 30 in Figure legends.

2. Injection of rAAV into hyperglycemic mice causes a 2-fold increase of liver weight (Fig. 2E), and histology of such liver (Fig. 2I) differs significantly from that of control (may be, glycogen deposition?). What causes these changes - does not discussed.

Could high ALT and AST levels (Fig. 2G, H) be caused by other forms of hepatocyte death (and not by necroptosis)?

Response:

We really appreciate Referee #3's comments for what caused the changes of liver weight and histology of liver in streptozotocin-induced hyperglycemic mice injected with rAAV. According to Referee #3's suggestions, we measured glycogen content in liver. As shown in Figure EV4A, two months after a single injection of rAAV, hyperglycemic mice induced by streptozotocin showed similar glycogen deposition compared with mice injected with PBS. We added the corresponding description in Materials and Methods, Results and Figure legends.

It has been reported that the oncogenic transcriptional coactivator Yes-associated protein (YAP), a nuclear effector of the Hippo signaling pathway, plays critical roles in organ size control, including liver (Li, Wu et al., 2022, Yu, Zhao et al., 2015). [redacted] We are intended to further investigate the detailed mechanism for the 2-fold increase of liver weight in streptozotocin-induced hyperglycemic mice injected with rAAV. Therefore, we prefer to present the following data in our next paper but not in this manuscript.

Figure for referees not shown.

To further investigate whether the high ALT and AST levels as shown in Figure 2G and 2H were caused by other forms of hepatocyte death, we examined the cleavage of

Caspase-3 and Caspase-8 as the markers of apoptosis, and found that both the cleaved Caspase-3 and Caspase-8 were dramatically increased in streptozotocin-induced hyperglycemic mice injected with rAAV compared to those injected with PBS (Figure EV4B). It has been reported that apoptosis may lead to the secondary necrosis, and caused the elevated ALT and AST levels (Rogers, Fernandes-Alnemri et al., 2017, Vanden Berghe, Vanlangenakker et al., 2010). We added the corresponding description in Materials and Methods, Results and Figure legends.

3. In the Results 2.5., it is described that three genes - Cyp4a14, Pebp1 and Tat - were chosen for validation by RT-PCR. However, it is not explained - how these genes were selected among the top 15 genes shown in Fig. 5A.

Response:

We completely agree with Referee #3's comments to explain how these genes were selected among the top 15 genes shown in Fig. 5A. Among the top 15 genes shown in Fig. 5A, Cyp4a14 plays an important role in in the development and progression of NAFLD (Zhang, Li et al., 2017), Pebp1 is an inflammatory and immune system modulator (Gabriela-Freitas, Pinheiro et al., 2019, Lai, Gu et al., 2017), and Tat plays an important suppressive role in the development and progression of HCC (Fu, Dong et al., 2010). However, the effects of these three genes involved in different signaling pathways on diabetes or necroptosis are still largely unknown. Therefore, we selected these three genes for further investigation.

"Among the top 15 genes upregulated in *db/db* mice, *Cyp4a14* plays an important role in in the development and progression of NAFLD (Zhang et al., 2017), Pebp1 is an inflammatory and immune system modulator (Gabriela-Freitas et al., 2019, Lai et al., 2017), and Tat plays an important suppressive role in the development and progression of HCC (Fu et al., 2010). However, the effects of these three genes involved in different signaling pathways on diabetes or necroptosis are still largely unknown." was inserted at line 18-21 on page 10 and line 1-2 on page 11.

4. In the legend to Fig. 5L it is mentioned that "hepatic mRNA levels of mice in (D)"; probably, there should be (K) instead of (D)?

Response:

We agree to Referee #3's comments for the correction of the legend to Fig. 5L. We mentioned that "The indicated hepatic mRNA levels of mice in (D)", because that the

samples used in 5L are from rAAV-si-*NC* and rAAV-si-*Pebp1* groups in 5D. Meanwhile we completely agree to Referee #3's suggestion for the correction, and the description that "The indicated hepatic mRNA levels of mice in (K)" is clearer than "The indicated hepatic mRNA levels of mice in (D)".

According to Referee #3's comments, we replaced "The indicated hepatic mRNA levels of mice in **(D)**" with "The indicated hepatic mRNA levels of mice in **(K)**" at line 14 on page 34.

5. In the Fig. 6D,E it can be seen that the control group is divided to two subgroups: one, having an increased ALT & AST levels (are there the same mice having high ALT & AST?), and another one, having the same ALT & AST levels as mice injected with rAAV-si-Pebp1. However, this is not discussed. But then it is not clear - which mice are shown in the Fig. 6F, G & H: whether mice from both control subgroups behave similarly in the tests shown in the Fig. 6F, G & H? Whether the endogenous Pebp1 level was similar between these two subgroups of control mice?

Response:

According to Referee #3's comments on Fig. 6D and Fig.6E, we carefully analyzed the data again, and found that the same mice having high ALT activity had high AST activity. The observation that the control group is divided to two subgroups is due to the incidence of liver injury and necroptosis induced by rAAV injection. As shown in Fig. 6D and 6E, 5 of 10 mice in control group were with high ALT and AST activities. As shown in Fig. 6F, 4 of the 5 mice in control group with high ALT and AST activities had hepatic necroptosis. To clearly show the same mice have high ALT & AST, we used colored dots to indicate the mice with high ALT or AST activity, and the same color dot in (D) and (E) indicates the same mouse (Fig. 6D and E). All the mice showed in Fig. 6B-E are shown in the Fig. 6F, G & H, and we indicated the number of mice used in the figure legend of revised manuscript at line 2-3 on page 35.

According to Referee #3's comments, we examined the endogenous Pebp1 level in the two subgroups of control mice. As shown in Figure EV4C and D, both Pebp1 mRNA and protein levels were similar in the high ALT & AST subgroup and low ALT & AST subgroup. In addition, we added the corresponding description in Results on page 12 and figure legends in Fig. 6 on page 35 and Figure EV4C and D on page 38.

6. All experiments of the section 2.7 of "Results" (shown in Fig. 7A-I) were performed in mouse primary macrophages. Because the authors of the study make recommendations for treatment of patients, it seems reasonable to demonstrate that the same effects take place also in human macrophages.

Response:

We really appreciate Referee #3's constructive suggestion to show that the effects of PEBP1 and TBK1 take place also in human macrophages. THP-1 monocyte is an immortalized human AML-derived cell line, which can differentiate to macrophages when treated with phorbol-12-myristate 13-acetate (PMA) (Shi & Kehrl, 2019). Macrophages differentiated from THP-1 human monocytes are frequently used as *in vitro* models (Chanput, Mes et al., 2014, Shi & Kehrl, 2019). Here we used THP-1 human macrophages to investigate the effects of PEBP1 and TBK1. As shown in Figure EV5A and B, downregulation of PEBP1 and TBK1 in THP-1 human macrophages by siRNAs were confirmed by qPCR, and knockdown of Pebp1 and Tbk1 markedly inhibited the increase of some inflammatory factors induced by poly(I:C). As expected, necroptosis of macrophages monitored by propidium iodide staining and p-MLKL level was also significantly alleviated by downregulation of PEBP1 and TBK1 (Figure EV5C-E). We added the corresponding description in Materials and Methods on page 22, Results on page 13 and Figure legends on page 38-39.

Minor comments:

1. Typos: p.9, lane 9 (Results, 2.4): "... we first investigated" ("d" is omitted); p.10, lane 18: "While..." is unnecessary, should be removed; p.12, lane 3: after "inflammation" there are should be "." instead of ",".

Response:

According to Referee #3's comments, we replaced "we first **investigate** whether prednisone can alleviate inflammation and necroptosis *in v itro*" with "we first **investigated** whether prednisone can alleviate inflammation and necroptosis *in vitro*" at line 13 on page 9 (Results, 2.4).

According to Referee #3's comments, we replaced "**While serum** ALT and AST activities were significantly decreased..." with "**Serum** ALT and AST activities were significantly…" at line 7 on page 11.

According to Referee #3's comments, we replaced "To investigate the role of Pebp1 in necroptosis pathway, we first analyzed the role of Pebp1 in inflammation, Poly(I:C) was used to mimic virus infection in cell model to induce inflammation as described previously" with "To investigate the role of Pebp1 in necroptosis pathway, we first analyzed the role of Pebp1 in inflammation. Poly(I:C) was used to mimic virus infection in cell model to induce inflammation as described previously" at line 14-15 on page 12.

Reference:

Chanput W, Mes JJ, Wichers HJ (2014) THP-1 cell line: an in vitro cell model for immune modulation approach. Int Immunopharmacol 23: 37-45

Fu L, Dong SS, Xie YW, Tai LS, Chen L, Kong KL, Man K, Xie D, Li Y, Cheng Y, Tao Q, Guan XY (2010) Down-Regulation of Tyrosine Aminotransferase at a Frequently Deleted Region 16q22 Contributes to the Pathogenesis of Hepatocellular Carcinoma. Hepatology 51: 1624-1634

Gabriela-Freitas M, Pinheiro J, Raquel-Cunha A, Cardoso-Carneiro D, Martinho O (2019) RKIP as an Inflammatory and Immune System Modulator: Implications in Cancer. Biomolecules 9

Lai R, Gu M, Jiang W, Lin W, Xu P, Liu Z, Huang H, An H, Wang X (2017) Raf Kinase Inhibitor Protein Preferentially Promotes TLR3-Triggered Signaling and Inflammation. J Immunol 198: 4086-4095

Li H, Wu BK, Kanchwala M, Cai J, Wang L, Xing C, Zheng Y, Pan D (2022) YAP/TAZ drives cell proliferation and tumour growth via a polyamine-eIF5A hypusination-LSD1 axis. Nat Cell Biol 24: 373-383

Rogers C, Fernandes-Alnemri T, Mayes L, Alnemri D, Cingolani G, Alnemri ES (2017) Cleavage of DFNA5 by caspase-3 during apoptosis mediates progression to secondary necrotic/pyroptotic cell death. Nat Commun 8: 14128

Shi CS, Kehrl JH (2019) Bcl-2 regulates pyroptosis and necroptosis by targeting BH3-like domains in GSDMD and MLKL. Cell Death Discov 5: 151

Vanden Berghe T, Vanlangenakker N, Parthoens E, Deckers W, Devos M, Festjens N, Guerin CJ, Brunk UT, Declercq W, Vandenabeele P (2010) Necroptosis, necrosis and secondary necrosis converge on similar cellular disintegration features. Cell Death Differ 17: 922-30

Yu FX, Zhao B, Guan KL (2015) Hippo Pathway in Organ Size Control, Tissue Homeostasis, and Cancer. Cell 163: 811-28

Zhang X, Li S, Zhou Y, Su W, Ruan X, Wang B, Zheng F, Warner M, Gustafsson JA, Guan Y (2017) Ablation of cytochrome P450 omega-hydroxylase 4A14 gene attenuates hepatic steatosis and fibrosis. Proc Natl Acad Sci U S A 114: 3181-3185

5th May 2023

Dear Prof. Zhai,

Thank you for the submission of your revised manuscript to EMBO Molecular Medicine. We have now received the enclosed report from the referee who re-reviewed your study. As you will see, this referee is supportive of publication, and we will therefore be able to accept your manuscript once the following editorial points will be addressed:

1/ Main manuscript text:
- Please accept all changes and only keep in track changes mode any new modification.
- Thank you for providing 5 keywords, please add them to the manuscript on the title page.
- Please carefully check the manuscript for grammar and language.
- Materials and methods:
o Animals: please provide the housing and husbandry conditions.
o Cell culture: please indicate the origin of the cells, and whether they were tested for mycoplasma contamination (kindly also provide this information in the authors checklist)
o Statistics: please include a sentence about randomization, blinding and inclusion/exclusion criteria. Please adjust the checklist accordingly.

2/ Checklist:
- Please remove the entry in "Animal observed in or captured from the field" as it doesn't apply to your study.
- Please complete the cells (mycoplasma) and statistics (blinding, inclusion/exclusion criteria) sections.
- Please indicate whether relevant guidelines or checklists have been followed or provided.

3/ Thank you for providing the Paper Explained. I added minor modifications, please check and amend as you see fit:
Problem: Obesity and diabetes are important risk factors for tumorigenesis, including liver cancer. Adeno-associated virus (AAV), widely used for gene therapy, is found in 30-80% of liver cancer patients. However, whether environmental or therapeutical AAV infection is a critical risk factor for liver cancer in individuals with obesity and diabetes is yet to be elucidated.
Results: rAAV injection leads to liver injury, hepatic necroptosis and liver cancer in hyperglycemic and obese mice, but not in hyperglycemic and slim mice or euglycemic and obese mice. Prednisone administration markedly alleviated liver injury and hepatic necroptosis in hyperglycemic and obese mice. Inhibition of Pebp1/Tbk1 signaling also attenuated liver injury, hepatic necroptosis and subsequent liver cancer caused by rAAV injection in hyperglycemic and obese mice.
Impact: Environmental AAV infection or AAV gene therapy for individuals with hyperglycemia and obesity should be carefully evaluated. Both prednisone treatment and targeting Pebp1/Tbk1 signaling are promising strategies to prevent or treat AAV-induced liver injury, hepatic necroptosis and related diseases including liver cancer.

4/ Thank you for providing a synopsis text. I added minor modification, let me know if you agree or amend as you see fit:
Hepatic necroptosis and HCC were caused by recombinant AAV injection in hyperglycemic and obese mice, which could be alleviated by prednisone administration or Pebp1/Tbk1 signaling inhibition.
- Injection of rAAV induced hepatic necroptosis and carcinoma in hyperglycemic and obese mice, but not in hyperglycemic and slim mice or euglycemic and obese mice.
- Oral administration of prednisone significantly alleviated rAAV-induced hepatic necroptosis in hyperglycemic and obese mice.
- Pebp1/Tbk1 mediated rAAV-induced hepatic necroptosis in hyperglycemic and obese mice.

5/ As part of the EMBO Publications transparent editorial process initiative (see our Editorial at http://embomolmed.embopress.org/content/2/9/329), EMBO Molecular Medicine will publish online a Review Process File (RPF) to accompany accepted manuscripts. We note that you would like the figures removed from your point-by-point letter.
Please note that the Authors checklist will be published at the end of the RPF.

I look forward to receiving your revised manuscript.

Yours sincerely,

Lise Roth

Lise Roth, PhD
Senior Editor
EMBO Molecular Medicine

The system will prompt you to fill in your funding and payment information. This will allow Wiley to send you a quote for the article processing charge (APC) in case of acceptance. This quote takes into account any reduction or fee waivers that you may be eligible for. Authors do not need to pay any fees before their manuscript is accepted and transferred to our publisher.

***** Reviewer's comments *****

Referee #3 (Comments on Novelty/Model System for Author):

The authors have solved all the problems mentioned by the reviewers: corrected mistakes, improved language of the article, explained issues that were not clear, performed additional experiments and added the appropriate data to the manuscript.

Referee #3 (Remarks for Author):

No additional remarks to the authors.

The authors have addressed all minor editorial requests.

9th May 2023

Dear Prof. Zhai,

Thank you for providing the revised files. We are pleased to inform you that your manuscript is accepted for publication and is now being sent to our publisher to be included in the next available issue of EMBO Molecular Medicine.

Please note that I slightly modified the abstract, please let me know if it is fine as is or if you would like to amend it:

Abstract:
Obesity and diabetes are risk factors for hepatocellular carcinoma (HCC); however, the underlying mechanisms are yet to be elucidated. Adeno-associated virus (AAV) frequently infects humans and has been widely used in gene therapy, but the risk of AAV-mediated infection such as HCC should be further evaluated. Here, we show that recombinant AAV injection caused liver injury, hepatic necroptosis and HCC in db/db or high-fat diet-induced hyperglycemic and obese mice, but not in mice with only hyperglycemia or obesity. Prednisone administration or knockdown of Pebp1, highly expressed in db/db mice, alleviated hepatic injury and necroptosis induced by recombinant AAV in mice with diabetes and obesity. Inhibition of Pebp1 pathway also attenuated inflammation and necroptosis in vitro. Our findings show that AAV infection is a critical risk factor for HCC in patients with diabetes and obesity, and AAV gene therapy for these patients should be carefully evaluated. Both prednisone treatment and targeting Pebp1 pathway are promising strategies to alleviate inflammation and necroptosis that occurred in AAV gene therapy or related diseases.

In the Paper Explained, note that I have changed "people" for "of the population" (see below). Please confirm that this is correct.

Problem: Obesity and diabetes are important risk factors for tumorigenesis, including liver cancer. Adeno-associated virus (AAV), widely used for gene therapy, is found in about 30-80% of the population. However, whether environmental or therapeutical AAV infection is a critical risk factor for liver cancer in individuals with obesity and diabetes is yet to be elucidated.

Results: rAAV injection leads to liver injury, hepatic necroptosis and liver cancer in hyperglycemic and obese mice, but not in hyperglycemic and slim mice or euglycemic and obese mice. Prednisone administration markedly alleviated liver injury and hepatic necroptosis in hyperglycemic and obese mice. Inhibition of Pebp1/Tbk1 signaling also attenuated liver injury, hepatic necroptosis and subsequent liver cancer caused by rAAV injection in hyperglycemic and obese mice.

Impact: Environmental AAV infection or AAV gene therapy for individuals with hyperglycemia and obesity should be carefully evaluated. Both prednisone treatment and targeting Pebp1/Tbk1 signaling are promising strategies to prevent or treat AAV-induced liver injury, hepatic necroptosis and related diseases including liver cancer.

Please read below for additional IMPORTANT information regarding your article, its publication and the production process.

Congratulations on your interesting work!

With kind regard,

Lise Roth

Lise Roth, Ph.D
Senior Editor
EMBO Molecular Medicine

*** *** *** IMPORTANT INFORMATION *** *** ***

SPEED OF PUBLICATION
The journal aims for rapid publication of papers, using using the advance online publication "Early View" to expedite the process: A properly copy-edited and formatted version will be published as "Early View" after the proofs have been corrected. Please help the Editors and publisher avoid delays by providing e-mail address(es), telephone and fax numbers at which

author(s) can be contacted.

Should you be planning a Press Release on your article, please get in contact with embomolmed@wiley.com as early as possible, in order to coordinate publication and release dates.

LICENSE AND PAYMENT:

All articles published in EMBO Molecular Medicine are fully open access: immediately and freely available to read, download and share.

EMBO Molecular Medicine charges an article processing charge (APC) to cover the publication costs. You, as the corresponding author for this manuscript, should have already received a quote with the article processing fee separately. Please let us know in case this quote has not been received.

Once your article is at Wiley for editorial production you will receive an email from Wiley's Author Services system, which will ask you to log in and will present you with the publication license form for completion. Within the same system the publication fee can be paid by credit card, an invoice, pro forma invoice or purchase order can be requested.

Payment of the publication charge and the signed Open Access Agreement form must be received before the article can be published online.

PROOFS

You will receive the proofs by e-mail approximately 2 weeks after all relevant files have been sent o our Production Office. Please return them within 48 hours and if there should be any problems, please contact the production office at embopressproduction@wiley.com.

Please inform us if there is likely to be any difficulty in reaching you at the above address at that time. Failure to meet our deadlines may result in a delay of publication.

All further communications concerning your paper proofs should quote reference number EMM-2022-17230-V3 and be directed to the production office at embopressproduction@wiley.com.

EMBO Press Author Checklist

Corresponding Author Name: Qiwei Zhai
Journal Submitted to: EMBO Molecular Medicine
Manuscript Number: EMM-2022-17230

USEFUL LINKS FOR COMPLETING THIS FORM
The EMBO Journal - Author Guidelines
EMBO Reports - Author Guidelines
Molecular Systems Biology - Author Guidelines
EMBO Molecular Medicine - Author Guidelines

Reporting Checklist for Life Science Articles (updated January
This checklist is adapted from Materials Design Analysis Reporting (MDAR) Checklist for Authors. MDAR establishes a minimum set of requirements in transparent reporting in the life sciences (see Statement of Task: 10.31222/osf.io/9sm4x). Please follow the journal's guidelines in preparing your
Please note that a copy of this checklist will be published alongside your article.

Abridged guidelines for figures
1. Data
The data shown in figures should satisfy the following conditions:
- the data were obtained and processed according to the field's best practice and are presented to reflect the results of the experiments in an accurate and unbiased manner.
- ideally, figure panels should include only measurements that are directly comparable to each other and obtained with the same assay.
- plots include clearly labeled error bars for independent experiments and sample sizes. Unless justified, error bars should not be shown for technical
- if n<5, the individual data points from each experiment should be plotted. Any statistical test employed should be justified.
- Source Data should be included to report the data underlying figures according to the guidelines set out in the authorship guidelines on Data

2. Captions
Each figure caption should contain the following information, for each panel where they are relevant:
- a specification of the experimental system investigated (eg cell line, species name).
- the assay(s) and method(s) used to carry out the reported observations and measurements.
- an explicit mention of the biological and chemical entity(ies) that are being measured.
- an explicit mention of the biological and chemical entity(ies) that are altered/varied/perturbed in a controlled manner.
- the exact sample size (n) for each experimental group/condition, given as a number, not a range;
- a description of the sample collection allowing the reader to understand whether the samples represent technical or biological replicates (including how many animals, litters, cultures, etc.).
- a statement of how many times the experiment shown was independently replicated in the laboratory.
- definitions of statistical methods and measures:
  - common tests, such as t-test (please specify whether paired vs. unpaired), simple χ2 tests, Wilcoxon and Mann-Whitney tests, can be unambiguously identified by name only, but more complex techniques should be described in the methods section;
  - are tests one-sided or two-sided?
  - are there adjustments for multiple comparisons?
  - exact statistical test results, e.g., P values = x but not P values < x;
  - definition of 'center values' as median or average;
  - definition of error bars as s.d. or s.e.m.

Please complete ALL of the questions below.
Select "Not Applicable" only when the requested information is not relevant for your study.

Materials

Newly Created Materials	Information included in the manuscript?	In which section is the information available? (Reagents and Tools Table, Materials and Methods, Figures, Data Availability Section)
New materials and reagents need to be available; do any restrictions apply?	Not Applicable	

Antibodies	Information included in the manuscript?	In which section is the information available? (Reagents and Tools Table, Materials and Methods, Figures, Data Availability Section)
For **antibodies** provide the following information:<br>- Commercial antibodies: RRID (if possible) or supplier name, catalogue number and or/clone number<br>- Non-commercial: RRID or citation	Yes	Materials and Methods

DNA and RNA sequences	Information included in the manuscript?	In which section is the information available? (Reagents and Tools Table, Materials and Methods, Figures, Data Availability Section)
**Short novel DNA or RNA including primers, probes**: provide the sequences.	Yes	Materials and Methods

Cell materials	Information included in the manuscript?	In which section is the information available? (Reagents and Tools Table, Materials and Methods, Figures, Data Availability Section)
**Cell lines:** Provide species information, strain. Provide accession number in repository **OR** supplier name, catalog number, clone number, and/**OR** RRID.	Yes	Materials and Methods
**Primary cultures:** Provide species, strain, sex of origin, genetic modification status.	Yes	Materials and Methods
Report if the cell lines were recently **authenticated** (e.g., by STR profiling) and tested for mycoplasma contamination.	Yes	Materials and Methods

Experimental animals	Information included in the manuscript?	In which section is the information available? (Reagents and Tools Table, Materials and Methods, Figures, Data Availability Section)
**Laboratory animals or Model organisms:** Provide species, strain, sex, age, genetic modification status. Provide accession number in repository **OR** supplier name, catalog number, clone number, **OR** RRID.	Yes	Materials and Methods
**Animal observed in or captured from the field:** Provide species, sex, and age where possible.	Not Applicable	
Please detail **housing and husbandry conditions**.	Yes	Materials and Methods

Plants and microbes	Information included in the manuscript?	In which section is the information available? (Reagents and Tools Table, Materials and Methods, Figures, Data Availability Section)
**Plants:** provide species and strain, ecotype and cultivar where relevant, unique accession number if available, and source (including location for collected wild specimens).	Not Applicable	
**Microbes:** provide species and strain, unique accession number if available, and source.	Not Applicable	

Human research participants	Information included in the manuscript?	In which section is the information available? (Reagents and Tools Table, Materials and Methods, Figures, Data Availability Section)
If collected and within the bounds of privacy constraints report on age, sex and gender or ethnicity for all study participants.	Not Applicable	

Core facilities	Information included in the manuscript?	In which section is the information available? (Reagents and Tools Table, Materials and Methods, Figures, Data Availability Section)
If your work benefited from core facilities, was their service mentioned in the acknowledgments section?	Yes	Acknowledgements

Design

Study protocol	Information included in the manuscript?	In which section is the information available? (Reagents and Tools Table, Materials and Methods, Figures, Data Availability Section)

If study protocol has been **pre-registered, provide DOI in the manuscript**. For clinical trials, provide the trial registration number **OR** cite DOI.	Not Applicable	
Report the **clinical trial registration number** (at ClinicalTrials.gov or equivalent), where applicable.	Not Applicable	

Laboratory protocol	Information included in the manuscript?	In which section is the information available? (Reagents and Tools Table, Materials and Methods, Figures, Data Availability Section)
Provide DOI OR other citation details if **external detailed step-by-step protocols** are available.	Not Applicable	

Experimental study design and statistics	Information included in the manuscript?	In which section is the information available? (Reagents and Tools Table, Materials and Methods, Figures, Data Availability Section)
Include a statement about **sample size** estimate even if no statistical methods were used.	Yes	Figure Legends
Were any steps taken to minimize the effects of subjective bias when allocating animals/samples to treatment (e.g. **randomization procedure**)? If yes, have they been described?	Yes	Materials and Methods
Include a statement about **blinding** even if no blinding was done.	Yes	Materials and Methods
Describe **inclusion/exclusion criteria** if samples or animals were excluded from the analysis. Were the criteria pre-established?<br><br>If sample or data points were omitted from analysis, report if this was due to attrition or intentional exclusion and provide justification.	Yes	Materials and Methods
For every figure, are **statistical tests** justified as appropriate? Do the data meet the assumptions of the tests (e.g., normal distribution)? Describe any methods used to assess it. Is there an estimate of variation within each group of data? Is the variance similar between the groups that are being statistically compared?	Yes	Materials and Methods

Sample definition and in-laboratory replication	Information included in the manuscript?	In which section is the information available? (Reagents and Tools Table, Materials and Methods, Figures, Data Availability Section)
In the figure legends: state number of times the experiment was **replicated** in laboratory.	Yes	Figure Legends
In the figure legends: define whether data describe **technical or biological replicates**.	Yes	Figure Legends

Ethics

Ethics	Information included in the manuscript?	In which section is the information available? (Reagents and Tools Table, Materials and Methods, Figures, Data Availability Section)
Studies involving **human participants**: State details of **authority granting ethics approval** (IRB or equivalent committee(s), provide reference number for approval.	Not Applicable	
Studies involving **human participants**: Include a statement confirming that **informed consent** was obtained from all subjects and that the experiments conformed to the principles set out in the WMA Declaration of Helsinki and the Department of Health and Human Services Belmont Report.	Not Applicable	
Studies involving **human participants:** For publication of **patient photos**, include a statement confirming that consent to publish was obtained.	Not Applicable	
Studies involving experimental **animals**: State details of **authority granting ethics approval** (IRB or equivalent committee(s), provide reference number for approval. Include a statement of compliance with ethical regulations.	Yes	Meterials and Methods
Studies involving **specimen and field samples:** State if relevant **permits** obtained, provide details of authority approving study; if none were required, explain why.	Not Applicable	

Dual Use Research of Concern (DURC)	Information included in the manuscript?	In which section is the information available? (Reagents and Tools Table, Materials and Methods, Figures, Data Availability Section)
Could your study fall under dual use research restrictions? Please check biosecurity documents and list of **select agents and toxins** (CDC): https://www.selectagents.gov/sat/list.htm	Not Applicable	
If you used a select agent, is the security level of the lab appropriate and reported in the manuscript?	Not Applicable	
If a study is subject to dual use research of concern regulations, is the name of the **authority granting approval and reference number** for the regulatory approval provided in the manuscript?	Not Applicable	

Reporting

The MDAR framework recommends adoption of discipline-specific guidelines, established and endorsed through community initiatives. Journals have their own policy about requiring specific guidelines and recommendations to complement MDAR.

Adherence to community standards	Information included in the manuscript?	In which section is the information available? (Reagents and Tools Table, Materials and Methods, Figures, Data Availability Section)
State if relevant guidelines or checklists (e.g., **ICMJE, MIBBI, ARRIVE, PRISMA**) have been followed or provided.	Not Applicable	
For **tumor marker prognostic studies,** we recommend that you follow the **REMARK** reporting guidelines (see link list at top right). See author guidelines, under 'Reporting Guidelines'. Please confirm you have followed these guidelines.	Not Applicable	
For **phase II and III randomized controlled trials**, please refer to the **CONSORT** flow diagram (see link list at top right) and submit the CONSORT checklist (see link list at top right) with your submission. See author guidelines, under 'Reporting Guidelines'. Please confirm you have submitted this list.	Not Applicable	

Data Availability

Data availability	Information included in the manuscript?	In which section is the information available? (Reagents and Tools Table, Materials and Methods, Figures, Data Availability Section)
Have **primary datasets** been deposited according to the journal's guidelines (see 'Data Deposition' section) and the respective accession numbers provided in the Data Availability Section?	Yes	Data Availability
Were **human clinical and genomic datasets** deposited in a public access-controlled repository in accordance to ethical obligations to the patients and to the applicable consent agreement?	Not Applicable	
Are **computational models** that are central and integral to a study available without restrictions in a machine-readable form? Were the relevant accession numbers or links provided?	Not Applicable	
If publicly available data were reused, provide the respective **data citations in the reference list**.	Not Applicable	
